# PhICl_2_-Mediated Regioselective and Electrophilic Oxythio/Selenocyanation of *o*-(1-Alkynyl)benzoates: Access to Biologically Active S/SeCN-Containing Isocoumarins

**DOI:** 10.3389/fchem.2022.859995

**Published:** 2022-05-18

**Authors:** Shanqing Tao, Aiwen Huo, Yan Gao, Xiangyang Zhang, Jingyue Yang, Yunfei Du

**Affiliations:** ^1^ Tianjin Key Laboratory for Modern Drug Delivery and High-Efficiency, School of Pharmaceutical Science and Technology, Tianjin University, Tianjin, China; ^2^ Hebei Key Laboratory of State Key Laboratory of Metastable Materials Science and Technology, Yanshan University, Qinhuangdao, China

**Keywords:** PhICl_2_, oxythiocyanation, oxyselenocyanation, *o*-(1-Alkynyl)benzoate, isocoumarin

## Abstract

The application of PhICl_2_/NH_4_SCN and PhICl_2_/KSeCN reagent systems to the synthesis of the biologically active S/SeCN-containing isocoumarins *via* a process involving thio/selenocyanation, enabled by thio/selenocyanogen chloride generated *in situ*, followed with an intramolecular lactonization was realized. Gram-scale synthesis, further derivatization to access C4 thio/selenocyanated Xyridin A and anti-tumor activities of the obtained products highlight the potential use of this method.

## Introduction

**FIGURE 1:**
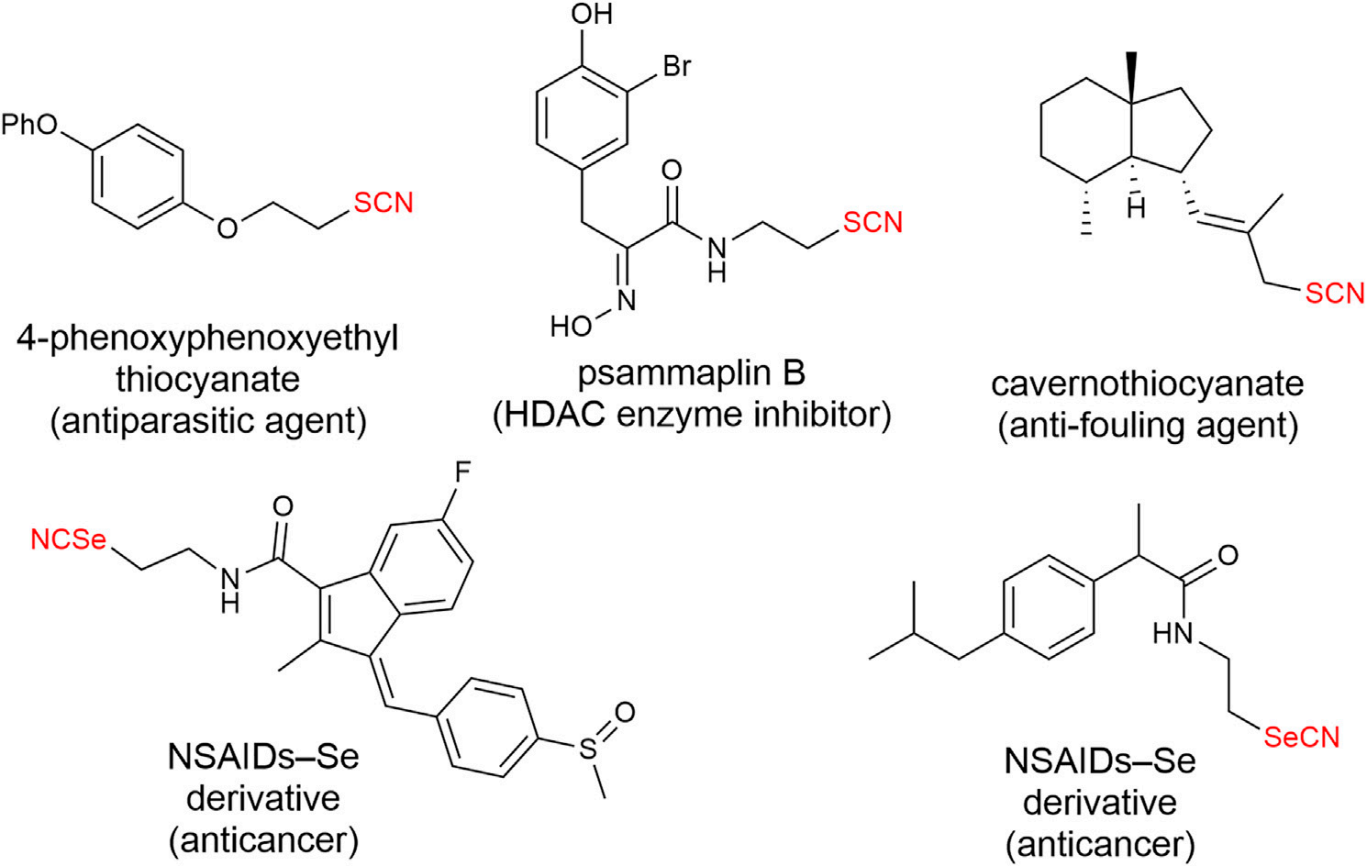
Representative examples of biologically interesting thio/selenocyanated compounds.

Organosulfur and selenium compounds have been widely used in organic and biological chemistry (Parnham and Graf, 1991; Ip and Ganther, 1992; Ji et al., 1999; Mugesh et al., 2001; Yao and Larock, 2003; Borges et al., 2005; Mehta et al., 2009; Santos et al., 2009; Sperança et al., 2011; Wilkins et al., 2016; Glenadel et al., 2018; Lin et al., 2020; Mampuys et al., 2020). Among them, organic thiocyanated compounds and their selenylated analogs have attracted continuous attention of organic and medicinal chemists. They can be used as versatile building blocks to achieve various useful synthetic transformations since their thiocyanato and selenocyanato moieties can be readily converted to other sulfur and selenium-containing functional groups (Castanheiro et al., 2016; Zhang et al., 2018; Wu et al., 2021; Yuan et al., 2021). In the meanwhile, naturally occurring or pharmaceutically interesting organothio/selenocyanates have been reported to show a broad spectrum of bioactivities (Chen et al., 2007). For instances, the SCN-containing 4-phenoxyphenoxyethyl thiocyanate (Elhalem et al., 2002), psammaplin B (Piña et al., 2003), and cavernothiocyanate (Hirota et al., 1996) have been evaluated as antiparasitic agents, HDAC enzyme inhibitor and antifouling agents, respectively (Figure 1). Furthermore, it is reported that some NSAID selenocyanated derivatives exert promising activities in reducing the viability of certain type of cancer cell lines (Plano et al., 2016; Liu et al., 2018) (Figure 1). In these regards, direct or late-stage introduction of thio/selenocyanato functional groups into bioactive compounds is of great significance in organic and medicinal chemistry.

The chemistry that describes the preparation of 4-chalcogen isocoumain through the cyclization of 2-alkynylaryl esters promoted by electrophilic chalcogen species has been well documented (Yao and Larock, 2003; Mehta et al., 2009; Sperança et al., 2011; Wilkins et al., 2016; Glenadel et al., 2018; Lin et al., 2020). On the other aspect, thio/selenocyanation of alkenes (Yang et al., 2015; Zhang et al., 2018; Ye et al., 2019; Meng et al., 2020; Nadiveedhi et al., 2020) and hetero/aromatics (Feng et al., 2018; Rezayati and Ramazani, 2020) has also been extensively investigated. However, the thio/selenocyantion of alkynes, a straightforward and versatile route to construct C_vinyl_–S(e) CN bond with the concomitant incorporation of a second functionality into the substrate, has remained less exploited. For examples, only dithio/selenocyanation (Prakash et al., 2001; Lu et al., 2018), hydrothiocyanation (Jiang et al., 2017; Wu et al., 2018), and iodothiocyanation (Zeng and Chen, 2018) of alkynes have been established so far. The thiocyanation and especially selenocyanation of alkynes coupled with an intramolecular functionalization, which could afford structurally diverse heterocycles remain unexplored. Our literature survey showed that the existing approaches include a metal-free synthesis of thiocyanato-containing azaspirotrienediones *via* photocatalytic carbothiocyanation of *N*-phenylpropynamides (Chen et al., 2019), a visible light-promoted carbon nitride-catalyzed thiocyanation of methylthiolated alkynones with NH_4_SCN affording the corresponding thiocyanated thioflavone products (Zeng et al., 2021), and a TCCA/NH_4_SCN-mediated cyclization/thiocyanation of alkynyl aryl ketones enabling the synthsis of 3-thiocyanated chromones (Xiao et al., 2021). Most recently, Zhou and co-workers reported the synthesis of isoquinolylsenocyanates and quinolylsenlenocyanates *via* electrophilic selenocyanogen cyclization induced by pseudohalogen (SeCN)_2_ generated *in situ* (Wang et al., 2022). Each of the above methods has its merits in preparing the corresponding S/SeCN-containing heterocycles. However, owing to the importance of the SCN/SeCN-containing heterocycles, it is still highly desirable to develop alternative and innovative approaches to realize the assemblage of versatile SCN/SeCN-containing heterocyclic framework.

**SCHEME 1:**
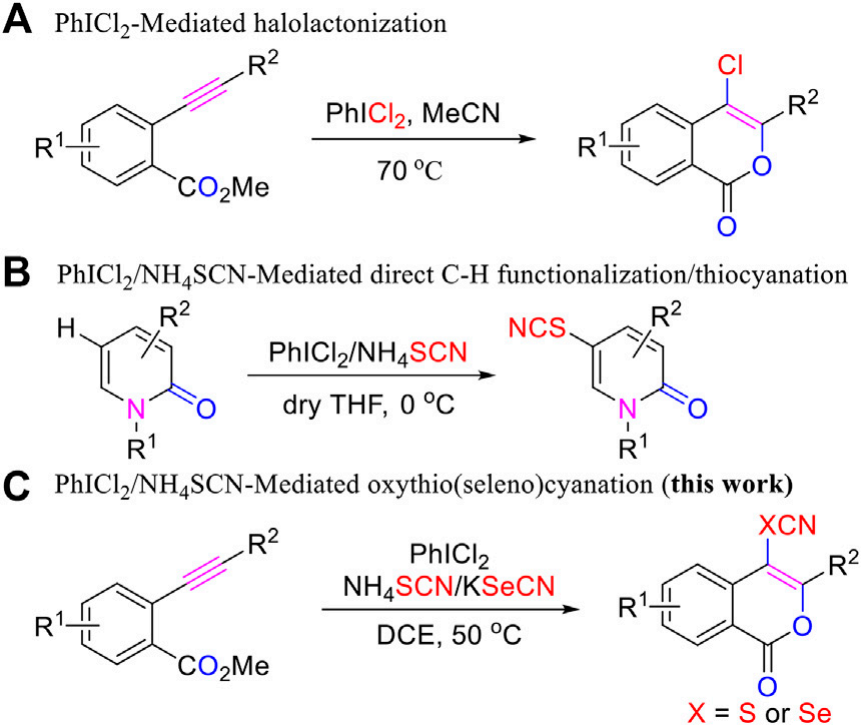
PhICl_2_-Mediated Synthesis of Functionalized Heterocycles.

PhICl_2_, the first hypervalent iodine reagent discovered in 1886 (Willgerodt, 1886), has found wide application in various organic transformations (Stang and Zhdankin, 1996). In 2019, we reported that PhICl_2_ could enable halolactonization of *ortho*-alkynylbenzoates, resulting in the formation of a series of functionalized 4-chloroisocoumarins under metal free conditions (Scheme 1A) (Xing et al., 2019b). By using PhICl_2_/NH_4_SCN system, we also realized the synthesis of the C5 thiocyanated 2-pyridones from pyridin-2(1*H*)-ones, demonstrating the efficiency of the reagents system in direct C-H functionalization/thiocyanation (Scheme 1B) (Tao et al., 2021). However, to the best of our knowledge, this oxidative PhICl_2_/NH_4_SCN system has never been used to the synthesis of thiocyanated heterocycles *via* intramolecular oxidative cyclization/oxythiocyanation of alkyne compounds, a strategy that is different from the above direct C–H functionalization/thiocyanation approach. In current work, we describe that by adopting PhICl_2_/NH_4_SCN reagents system, the biologically interesting C4-thiocyanated isocoumarins could be regioselectively achieved *via* oxythiocyanation of *o*-alkynylbenzoates. Furthermore, the protocol could be extended to the synthesis of C4-selenocyanated isocoumarins by using PhICl_2_/KSeCN, which is applied in organic synthesis for the first time (Scheme 1C).

## Results and Discussion

### Optimization of Reaction Conditions

At the outset of our studies, we were interested to investigate whether the PhICl_2_/NH_4_SCN reagent system could be applied to the intramolecular cyclization as well as oxythio/selenocyanation of *o*-alkynylbenzoates, in hope of achieving the biologically interesting C4-thio/selenocyanated isocoumarins. Our study on condition optimization was commenced with the simplest *o*-alkynylbenzoate **1a**. To our delight, the desired C4-thiocyanated isocoumarin **2a** could be obtained in 86% yield from the reaction of **1a** with 2 equivalents of PhICl_2_ and 2 equivalents of NH_4_SCN in DCM for 12 h at room temperature (Table 1, entry 1). The solvents screening showed DCE to be superior to other commonly used solvents, including DCM, MeOH, EtOAc, toluene, and MeCN (Table 1, entries 2–6). Neither increasing nor decreasing the dosage of PhICl_2_/NH_4_SCN were beneficial for improving the yield of the product (Table 1, entries 7–8). The other oxidants including PIDA, PIFA, PhIO, I_2_, and NBS were also applied to take the place of PhICl_2_, however, lower yield, no reaction or none desired product were observed in these cases (Table 1, entries 9–13). The other SCN-containing inorganic salts including NaSCN, KSCN, AgSCN and CuSCN have also been investigated, but none of them provided better outcome than NH_4_SCN (SI, Supplementary Table S1, entries 14–17). We have also found that the reaction temperature had an obvious influence on the outcome of the reaction. Performing the reaction of **1a** (0.20 mmol), PhICl_2_ (0.4 mmol) and NH_4_SCN (0.4 mmol) in DCE at 50°C not only improved the reaction yield to 96%, but also shorten the reaction time to 2 h (Table 1, entry 14). However, when the reaction temperature was further elevated to 60°C, it was found that product **2a** was obtained in relatively lower yield, with the starting substrate **1a** recovered in a yield of 5% (SI, Supplementary Table S1, entry 19). Furthermore, the reaction does not need a N_2_ atmosphere (Table 1, entry 14 vs 15). Based on the outcomes of the above screening experiments, the best yield of product **2a** (96%) could be obtained by subjecting NH_4_SCN (2 equiv), PhICl_2_ (2 equiv) to DCE at 50°C for 2 h (Table 1, entry 14).

**TABLE 1:** Optimization on the reaction conditions^a^.

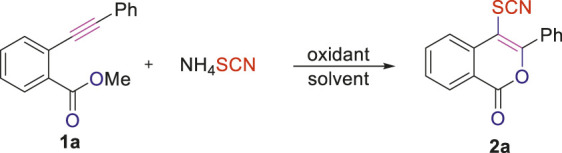				
Entry	Oxidant (equiv)	[SCN] (equiv)	Solvent	Yield (%)^b^ ^)^
1	PhICl_2_ (2)	NH_4_SCN (2)	DCM	80
2	PhICl_2_ (2)	NH_4_SCN (2)	DCE	92
3	PhICl_2_ (2)	NH_4_SCN (2)	MeOH	trace
4	PhICl_2_ (2)	NH_4_SCN (2)	EtOAc	trace
5	PhICl_2_ (2)	NH_4_SCN (2)	toluene	trace
6	PhICl_2_ (2)	NH_4_SCN (2)	MeCN	82
7	PhICl_2_ (1)	NH_4_SCN (1)	DCE	65
8	PhICl_2_ (3)	NH_4_SCN (3)	DCE	90
9	PIDA (2)	NH_4_SCN (2)	DCE	20
10	PIFA (2)	NH_4_SCN (2)	DCE	25
11	PhIO (2)	NH_4_SCN (2)	DCE	20
12	I_2_ (2)	NH_4_SCN (2)	DCE	NR^c^
13	NBS (2)	NH_4_SCN (2)	DCE	ND^d^
14^e^	PhICl_2_ (2)	NH_4_SCN (2)	DCE	96
15^f^	PhICl_2_ (2)	NH_4_SCN (2)	DCE	95

a Reaction coditions: A mixture of oxidant and NH_4_SCN, in solvent (5 ml) was stirred at rt for 0.5 h, then **1a** (0.20 mmol) was added, stirred at rt for 12 h.

b Yield of isolated products.

c NR = no reaction.

d ND = no desired product.

e **1a** (0.20 mmol) was added, stirred at 50°C for 2 h.

f **1a** (0.20 mmol) was added under N_2_ atmosphere, stirred at 50°C for 2 h.

### Scope Exploration of Substrates

With the optimized conditions in hand, we came to investigate the substrate scope of this reaction by subjecting various *o*-alkynylbenzoates to the standard condition (Table 2). First, the electronic effect of R^1^ substituent on the phenyl ring of *o*-alkynylbenzoates was explored. Substrates with either electron-donating or -withdrawing groups on the phenyl ring were found to well participate the cyclization/thiocyanation reaction and the desired C4 thiocyanated ioscoumarins **2b-f** were obtained in good to excellent yields. Moreover, it is worth noting that substrates bearing electron-donating groups (**1a-c**) exhibited better performance than those bearing electron-withdrawing substituents (**1d-f**). For instances, when R^1^ is a strong electron-withdrawing -NO_2_ group, the reaction smoothly afforded the corresponding isocoumarin **2d** in 56% yield. Replacement of the -NO_2_ group with -Cl or -F groups provided better results, with corresponding products **2e-f** obtained in 87 and 85%, respectively. The electronic effect of substituents on the alkyne motif was carefully tested next. To our satisfaction, this transformation was applicable to alkynoates bearing diverse substituents ranging from phenyl, naphthyl, thienyl to alkyl, with the corresponding C4 thiocyanated products **2g-t** accomplished in good to excellent yields. Most strikingly, TMS functionality in the substrate was also well tolerated under the reaction conditions and the target product **2r** was obtained in 80% yield. The method was also applicable to terminal alkyne, which could afford the corresponding C3-unsubstituted isocoumarin **2s** in 84% yield.

**TABLE 2:** Electrophilic thio/selenocyanation of *o*-alkynylbenzoate^a^.

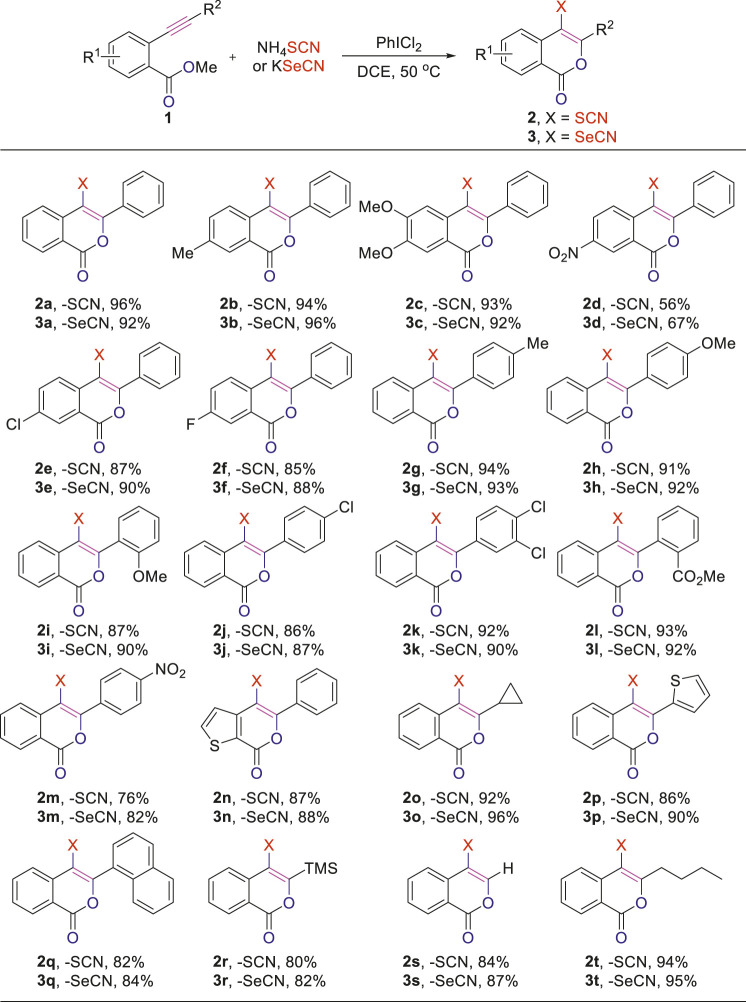

a A mixture of PhICl_2_ (0.4 mmol) and NH_4_SCN (0.4 mmol) or KSeCN (0.4 mmol) in DCE (5 ml) was stirred at rt for 0.5 h, then **1** (0.20 mmol) was added, stirred at 50°C for 2 h, isolated yields.

Encouraged by the feasibility of oxythiocyanation of *o*-alkynylbenzoates, we further explored the synthesis of C4 selenocyanated isocoumarins, a closely related analogue of thiocyanated isocoumarins. Since the NH_4_SeCN was not commercially available, we had to resort to other selenocyanate sources (SI, Supplementary Table S2). To our delight, when KSeCN was applied, the reaction worked equally well as the oxythiocyanation and the corresponding selenocyanated product **3a** was obtained in 92% yield. Under the adjusted conditions, a series of *o*-alkynylbenzoates were converted to the corresponding selenocyanated analogs **3b-m** in high to excellent yields, regardless of the electronic effect of substituents on the phenyl ring. Notably, functional groups such as -F, -Cl, -NO_2_, and -CO_2_Me remained intact during the transformation, thereby facilitating late-stage functionalization of the obtained products. Furthermore, heterocycle-fused substrate **1n** also reacted smoothly under the newly optimized reaction conditions, affording the corresponding product **3n** in good yield. Finally, good to excellent yields were observed for substrates with R^2^ being substituents ranging from aliphatic, hetero/aromatic ring to TMS functionality (**3o-t**). The structure of **2h** (CCDC: 2126012) and **3h** (CCDC: 2126018) were unambiguously confirmed through X-ray crystallographic analysis, for details, see supporting information.

### Synthetic Applications

**SCHEME 2:**
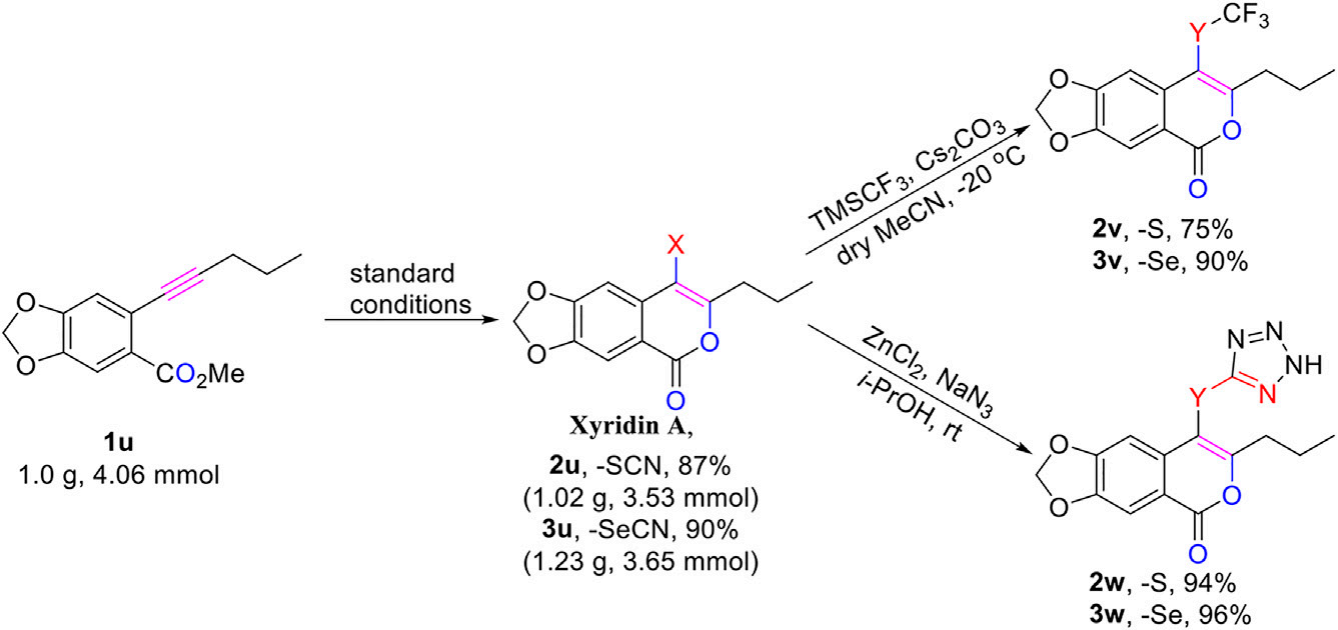
Gram-scale Synthesis and Product Derivatization.

The isocoumarin skeleton commonly exists in many naturally-occurring compounds and potent pharmaceutical agents displaying anti-tumor, antifungal, and anti-inflammatory properties (Pal and Pal, 2019). For instance, Xyridin A (Ruangrungsi et al., 1995) was isolated in 1995 from *Xyris indica*, and was found to possess antibacterial activity against various bacteria (Saeed, 2003). To further demonstrate the synthetic utility of our method, a gram-scale reaction was performed with substrate **1u** under standard conditions and the corresponding C4 thio/selenocyanated Xyridin A derivatives **2u** and **3u** were obtained in 87 and 90%, respectively (Scheme 2). Ultimately, compounds **2u** and **3u** could be readily transformed into SCF_3_- (Liang et al., 2015) and SeCF_3_-containing Xyridin A derivatives **2v** and **3v** by treatment with TMSCF_3_ and Cs_2_CO_3_ in acetonitrile. Moreover, [3 + 2] cycloaddition of compounds **2u**/**3u** with sodium azide was also performed to afford Xyridin A bearing thiotetrazole moiety **2w**/**3w** in 94 and 96% yields (Liang et al., 2015), respectively.

### Investigation of Mechanism

**SCHEME 3:**
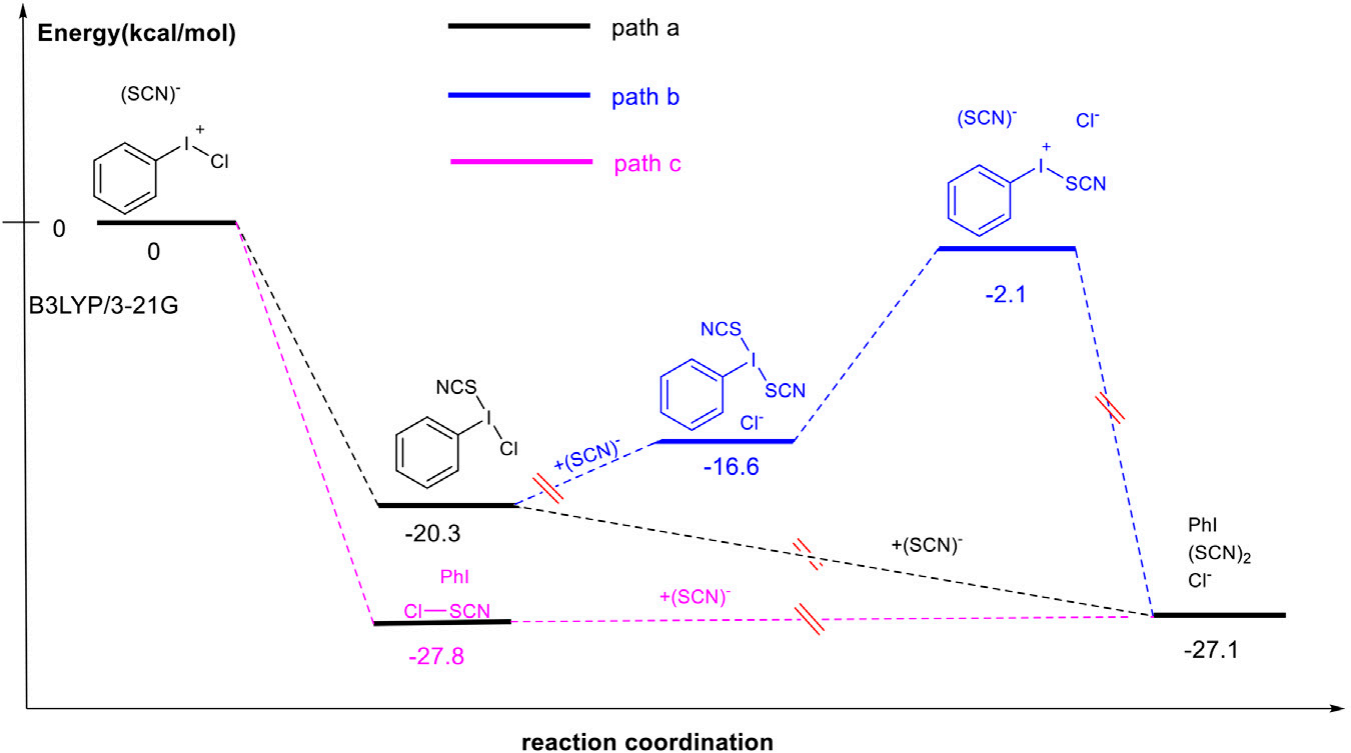
DFT Computation of Possible Reaction Pathways to (SCN)_2_.

We have previously postulated that the reaction of PhICl_2_ with NH_4_SCN could generate PhI(SCN)_2_ as a reactive hypervalent iodine (III) species *via* a ligand-exchange process (Scheme 3). In order to further corroborate whether PhI(SCN)_2_ species was indeed formed, the reaction of TolICl_2_ with NH_4_SCN in CDCl_3_ was carried out and ^1^H NMR analysis was implemented. The outcome revealed that only the peak of 2.46 ppm (s), which can be attributed to the methyl group of TolICl_2_, and the peak of 2.29 ppm(s), which can be attributed to the methyl group of *p*-iodotoluene, were observed throughout the whole reaction process (see SI for details). No signal from PhI(SCN)_2_ was detected, though. In order to further understand the most appropriate pathway adopted by the reaction between PhICl_2_ with NH_4_SCN, a computational study was carried out and the result is shown in Scheme 3. The reaction pathway b involving formation of PhI(SCN)_2_ obviously requires more energy than other pathways. Even though pathway a is also theoretically possible, the ^1^H NMR experiment result did not support the formation of PhICl(SCN). It is not the reaction pathway with the lowest energy, either. Pathway c with the formation of ClSCN is not only consistent with the ^13^C NMR experiment (Tao et al., 2021), but also preferred thermodynamically (see SI for details).

**SCHEME 4:**
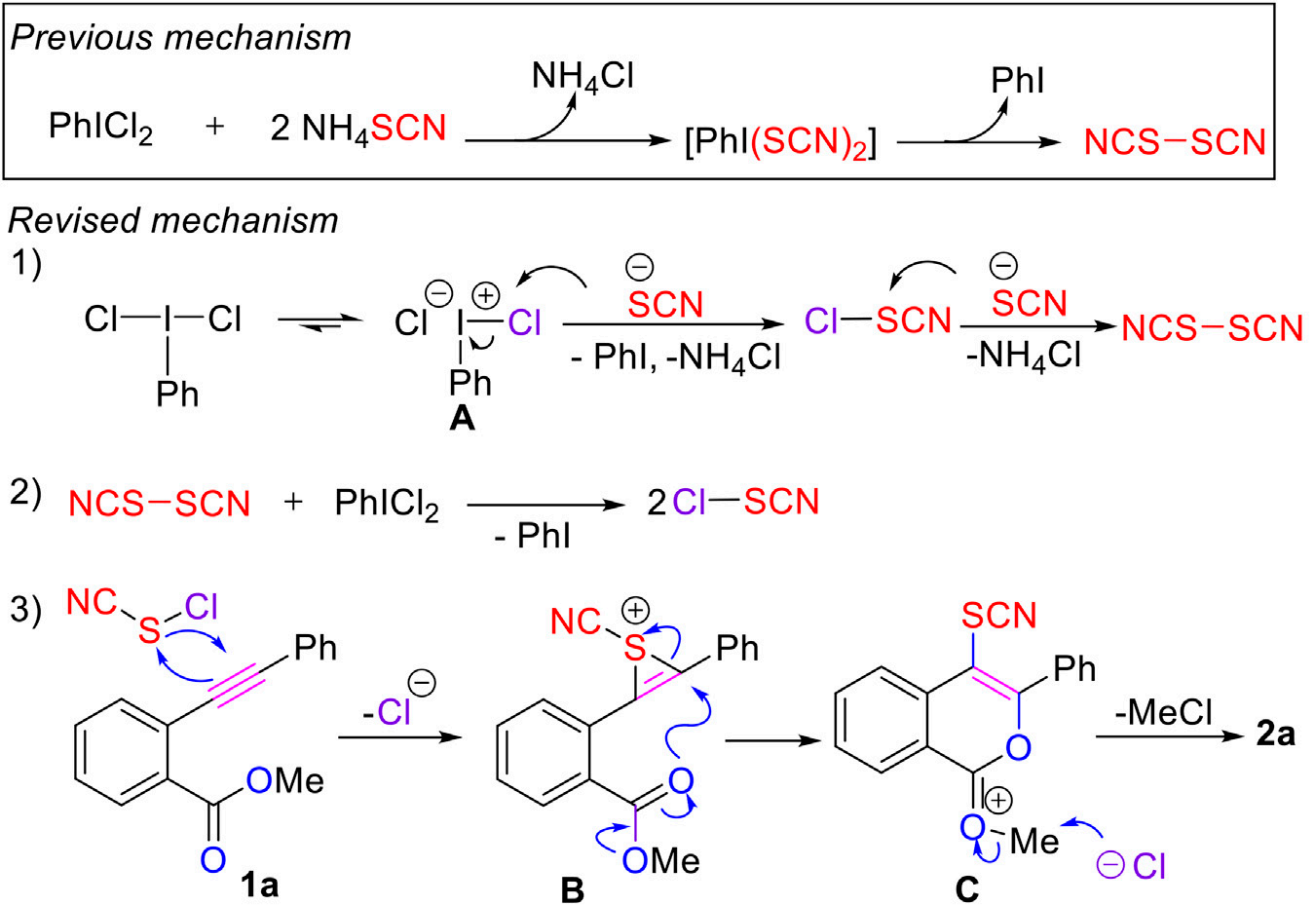
Proposed Mechanistic Pathway.

Based on these newly gained experimental (control experiments see ESI, Supplementary Scheme S1, SupplementaryFigure S1, S2 and computational calculation) and computational results as well as previous literature reports (Kita et al., 1997; Woon et al., 2006; Ito et al., 2019; Xing et al., 2019a; An et al., 2020), a possible mechanistic pathway for the formation of the C4 thiocyanated isocumarins was proposed (Scheme 4). Differing from the previous mechanism (Kita et al., 1997), we tentatively proposed that intermediate **A**, an ionic form of PhICl_2_, reacted with thiocyanate directly to give the reactive thiocyanogen chloride, which could be supported by the observation of peak of 109.1 ppm in its ^13^C NMR analysis (Tao et al., 2021). Then the reaction of thiocyanate with thiocyanogen chloride provides (SCN)_2_, which further reacts with the oxidative PhICl_2_ to give thiocyanogen chloride (Tao et al., 2021). Next, electrophilic addition between the reactive thiocyanogen chloride with substrate **1a** gave rise to intermediate **B** (Xing et al., 2019a; An et al., 2020; Hellwig et al., 2020; Lynch and Scanlan, 2020; Sun et al., 2020; Jurinic et al., 2021; Slivka and Onysko, 2021). Due to the presence of the adjacent electron-withdrawing methoxycarbonyl group which makes the C (sp^2^) connecting with the Ph substituent more electron-deficient, a favored intramolecular *6-exo* cyclization occurred in intermediate **B**, leading to formation of the cyclic intermediate **C**. Finally, removal of the methyl group by the nucleophilic attack of chloride ion gave the title product **2a** (Scheme 4).

### CCK-8 Assay

**FIGURE 2:**
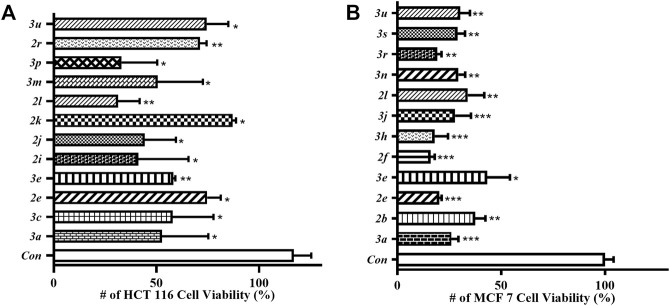
Antitumor activity of synthesized 4-thio/selenocyanated isocoumarins (10 μM) against HCT 116 **(A)** and MCF 7 **(B)** cell lines, determined by a CCK-8 assay.

Finally, those synthesized 4-thio/selenocyanated isocoumarins were screened *in vitro* for antitumor activity test using CCK-8 assay against HCT 116 and MCF 7 cell lines (for details, see the ESI, Supplementary Figure S5). The results in Figure 2 indicated that these compounds showed moderate activity against HCT 116 after 48 h exposure. Compound **2l** was found to be the most potent candidate of the series, which exerted 69% inhibition at 10 μM concentration against HCT 116. Moreover, the screening results revealed that those compounds were active against MCF 7 cell line. Especially, compound **2f**, **3h**, and **3r** seem to be equally potent with 84, 82, and 81% antiproliferative inhibition at 10 μM concentration against MCF 7 cell line, respectively.

## Conclusion

In summary, we have realized an alternative synthesis of C4 thio/selenocyanated isocoumarins in a highly regioselective manner with good to excellent yields. Compared with the previous direct C–H functionalization/thiocyanation of a heterocyclic skeleton, this method realized the construction of the functionalized isocoumarin framework *via* a hypervalent iodine-mediated electrophilic thio/selenocyanation approach. In addition to the features of metal-free conditions, mild reaction conditions, high-yielding of products and broad tolerance of functional groups, the obtained thio/selenocyanated isocoumarins were found to possess anti-tumor activities and have been proven to be useful building blocks, as the functionalized Xyridin A could be converted to other pharmaceutically interesting Xyridin A derivatives.

## Materials and Methods

Reagents and solvents were purchased as reagent grade and were used without further purification. PhICl_2_ (Zhao and Zhang, 2007) were prepared according to literature methods. All reactions were performed in standard glassware, heated at 70°C for 3 h before use. Flash column chromatography was performed over silica gel (200–300°mesh) using a mixture of ethyl acetate (EtOAc), and petroleum ether (PE).

### Experimental Details General Procedure for tbl2

To an oven-dried 25 ml round-bottom flask were added NH_4_SCN or KSeCN (0.4 mmol), PhICl_2_ (0.4 mmol) and DCE (5 ml). The mixture was stirred at rt for 30 min. Then, substrate **1** (0.2 mmol) in DCE (5 ml) was added to the reaction mixture in one portion. The reaction was heated to 50°C in an aluminum heating block and stirred for another 2 h, poured into the saturated brine solution (20 ml). The product was extracted with DCM (20 ml), dried with Na_2_SO_4_ and concentrated. The crude product was purified using silica gel column chromatography.Table 2

## Data Availability

The original contributions presented in the study are included in the article/Supplementary Material, further inquiries can be directed to the corresponding authors.
