# Supplementary material for: PhICl2-Mediated Regioselective and Electrophilic Oxythio/Selenocyanation of o-(1-Alkynyl)benzoates: Access to Biologically Active S/SeCN-Containing Isocoumarins
Source: Front Chem. 2022 May 18;10:859995. doi: 10.3389/fchem.2022.859995 (PMC9158338; doi:10.3389/fchem.2022.859995)
Supplement: Supplementary file 1 [file DataSheet1.docx]

***Supplementary Material***

**Contents**

| I | Optimization Study for Formation of **2a** and **3a** | S1 |
| --- | --- | --- |
| II | Experiment Data of **2** and **3** | S2 |
| III | Control Experiments | S30 |
| IV | Biological Experiments | S41 |
| V | Reference | S42 |
| VI | NMR Spectra of Products | S43 |
| VII | X-Ray Structure and Data | S162 |

**I. Optimization Study for Formation of 2a and 3a**

Table S1. Optimization on the formation of **2a***^a^*

| Entry | Oxidant (equiv) | [SCN] (equiv) | Solvent | Yield(%)*^b^* |
| --- | --- | --- | --- | --- |
| 1 | PhICl_2_ (2) | NH_4_SCN (2) | DCM | 80 |
| 2 | PhICl_2_ (2) | NH_4_SCN (2) | DCE | 92 |
| 3 | PhICl_2_ (2) | NH_4_SCN (2) | MeOH | trace |
| 4 | PhICl_2_ (2) | NH_4_SCN (2) | EtOAc | trace |
| 5 | PhICl_2_ (2) | NH_4_SCN (2) | toluene | trace |
| 6 | PhICl_2_ (2) | NH_4_SCN (2) | MeCN | 82 |
| 7 | PhICl_2_ (1) | NH_4_SCN (1) | DCE | 65 |
| 8 | PhICl_2_ (3) | NH_4_SCN (3) | DCE | 90 |
| 9 | PIDA (2) | NH_4_SCN (2) | DCE | 20 |
| 10 | PIFA (2) | NH_4_SCN (2) | DCE | 25 |
| 11 | PhIO (2) | NH_4_SCN (2) | DCE | 20 |
| 12 | I_2_ (2) | NH_4_SCN (2) | DCE | NR*^c^* |
| 13 | NBS (2) | NH_4_SCN (2) | DCE | ND*^d^* |
| 14 | PhICl_2_ (2) | NaSCN (2) | DCE | 87 |
| 15 | PhICl_2_ (2) | KSCN (2) | DCE | 82 |
| 16 | PhICl_2_ (2) | AgSCN (2) | DCE | 90 |
| 17 | PhICl_2_ (2) | CuSCN (2) | DCE | 65 |
| **18*^e^*** | **PhICl_2_ (2)** | **NH_4_SCN (2)** | **DCE** | **96** |
| 19*^f^* | PhICl_2_ (2) | NH_4_SCN (2) | DCE | 90 |
| 20*^g^* | PhICl_2_ (2) | NH_4_SCN (2) | DCE | 87 |
| 21*^h^* | PhICl_2_ (2) | NH_4_SCN (2) | DCE | 95 |

*^a^*Reaction coditions: A mixture of oxidant and [SCN] in solvent (5 mL) was stirred at rt for 0.5 h, then **1a** (0.20 mmol) was added, stirred at rt for 12 h. *^b^*Yield of isolated products. *^c^*NR = no reaction. *^d^*ND = no desired product. *^e^***1a** (0.20 mmol) was added, stirred at 50 ^o^C for 2 h. *^f^***1a** (0.20 mmol) was added, stirred at 60 ^o^C for 2 h. *^g^***1a** (0.20 mmol) was added, stirred at 70 ^o^C for 2 h. *^h^***1a** (0.20 mmol) was added under N_2_ atmosphere, stirred at 50 ^o^C for 2 h.

Table S2. Optimization on the formation of **3a***^a^*

| Entry | [SeCN] | Yield(%)*^b^* |
| --- | --- | --- |
| 1 | KSeCN | 92 |
| 2 | AgSeCN | 90 |

*^a^*Reaction coditions: A mixture of PhICl_2_ (0.4 mmol)and [SeCN] (0.4 mmol) in DCE (5 mL) was stirred at rt for 0.5 h, then **1a** (0.20 mmol) was added, stirred at 50 ^o^C for 2 h. *^b^*Yield of isolated products.

# II Experimental Data of 2 and 3

**General information.** ^1^H and ^13^C NMR spectra were recorded on 600 MHz and 400 MHz spectrometers at 25 ^o^C. Chemical shifts values are given in ppm. Data are reported as follows: chemical shift, multiplicity (s = singlet, d =doublet, t = triplet, q = quartet, br = broad, m = multiplet). The coupling constants *J* are reported in Hertz (Hz). High resolution mass spectrometry (HRMS) was obtained on a Q-TOF micro spectrometer. Melting points were determined by a Micro melting point apparatus. TLC plates were visualized by exposure to ultraviolet light.

Reagents and solvents were purchased as reagent grade and were used withoutfurther purification. All reactions were performed in standard glassware, heated at 70 ^o^C for 3 h before use. Flash column chromatography was performed over silica gel (200-300 mesh) using a mixture of ethyl acetate (EtOAc), and petroleum ether (PE).

**General procedure for the synthesis of 2 and 3**

To an oven-dried 25 mL round-bottom flask were added NH_4_SCN or KSeCN (0.4 mmol), PhICl_2_ (0.4 mmol) and DCE (5 mL). The mixture was stirred at rt for 30 min. Then, substrate **1** (0.2 mmol) in DCE (5 mL) was added to the reaction mixture in one portion. The reaction was heated to 50 ^o^C in an aluminum heating block and stirred for another 2 h, poured into the saturated brine solution (20 mL). The product was extracted with DCM (20 mL), dried with Na_2_SO_4_ and concentrated. The crude product was purified using silica gel column chromatography.

**3-Phenyl-4-thiocyanato-1*H*-isochromen-1-one (2a)**

The reaction was accomplished according to the general experimental procedure described above. Product **2a** was purified by flash column chromatography (silica gel, EtOAc/PE = 10/90). Yield: 53.6 mg, 96%, a white solid, mp. 189 – 190 ^o^C. ^1^H NMR (400 MHz, CDCl_3_) δ 8.40 (dd, *J* = 7.8, 1.4 Hz, 1H), 8.11 (dd, *J* = 8.1, 1.2 Hz, 1H), 7.97 (ddd, *J* = 8.3, 7.3, 1.4 Hz, 1H), 7.78 – 7.71 (m, 2H), 7.69 (td, *J* = 7.6, 1.1 Hz, 1H), 7.62 – 7.50 (m, 3H). ^13^C NMR (101 MHz, CDCl_3_) δ 161.5, 160.1, 136.1, 135.8, 131.6, 131.3, 130.5, 129.7, 129.7, 128.6, 124.9, 120.6, 110.0, 100.1. HRMS (ESI) m/z: [M + Na]^+^ Calcd for C_16_H_9_NO_2_SNa 302.0246; Found 302.0252.

**3-Phenyl-4-selenocyanato-1*H*-isochromen-1-one (3a)**

The reaction was accomplished according to the general experimental procedure described above. Product **3a** was purified by flash column chromatography (silica gel, EtOAc/PE = 20/80). Yield: 60.0 mg, 92%, a white solid, mp. 224 – 225 ^o^C. ^1^H NMR (400 MHz, DMSO-*d*_6_) δ 8.32 – 8.28 (m, 1H), 8.17 – 8.07 (m, 2H), 7.80 – 7.75 (m, 3H), 7.63 – 7.54 (m, 3H). ^13^C NMR (101 MHz, DMSO-*d*_6_) δ 160.7, 159.9, 137.3, 136.8, 133.9, 131.1, 130.2, 130.2, 130.0, 128.7, 127.9, 120.5, 105.3, 103.4. ^77^Se NMR (76 MHz, CDCl_3_) δ 232.1. HRMS (ESI) m/z: [M + Na]^+^ Calcd for C_16_H_9_NO_2_SeNa 349.9691; Found 349.9697.

**7-Methyl-3-phenyl-4-thiocyanato-1*H*-isochromen-1-one (2b)**

The reaction was accomplished according to the general experimental procedure described above. Product **2b** was purified by flash column chromatography (silica gel, EtOAc/PE = 10/90). Yield: 55.1 mg, 94%, a white solid, mp. 189 – 190 ^o^C. ^1^H NMR (400 MHz, CDCl_3_) δ 8.24 – 8.17 (m, 1H), 7.99 (d, *J* = 8.2 Hz, 1H), 7.85 – 7.69 (m, 3H), 7.62 – 7.47 (m, 3H), 2.55 (s, 3H). ^13^C NMR (101 MHz, CDCl_3_) δ 160.5, 160.3, 140.4, 137.2, 133.3, 131.6, 131.2, 130.2, 129.7, 128.5, 124.9, 120.5, 110.1, 100.1, 21.4. HRMS (ESI) m/z: [M + Na]^+^ Calcd for C_17_H_11_NO_2_SNa 316.0403; Found 316.0413.

**7-Methyl-3-phenyl-4-selenocyanato-1*H*-isochromen-1-one (3b)**

The reaction was accomplished according to the general experimental procedure described above. Product **3b** was purified by flash column chromatography (silica gel, EtOAc/PE = 20/80). Yield: 65.3 mg, 96%, a white solid, mp. 208 – 209 ^o^C. ^1^H NMR (400 MHz, CDCl_3_) δ 8.25 – 8.14 (m, 1H), 7.96 (d, *J* = 8.2 Hz, 1H), 7.75 (dd, *J* = 8.4, 1.9 Hz, 1H), 7.69 – 7.60 (m, 2H), 7.57 – 7.48 (m, 3H), 2.54 (s, 3H). ^13^C NMR (101 MHz, CDCl_3_) δ 160.7, 160.0, 140.3, 137.2, 134.1, 132.9, 131.0, 130.1, 129.8, 128.4, 127.2, 120.5, 100.2, 99.9, 21.3. ^77^Se NMR (76 MHz, CDCl_3_) δ 231.9. HRMS (ESI) m/z: [M + Na]^+^ Calcd for C_17_H_11_NO_2_SeNa 363.9847; Found 363.9852.

**6,7-Dimethoxy-3-phenyl-4-thiocyanato-1*H*-isochromen-1-one (2c)**

The reaction was accomplished according to the general experimental procedure described above. Product **2c** was purified by flash column chromatography (silica gel, EtOAc/PE = 10/90). Yield: 63.1 mg, 93%, a white solid, mp. 170 – 172 ^o^C. ^1^H NMR (600 MHz, CDCl_3_) δ 7.74 (s, 1H), 7.74 – 7.70 (m, 2H), 7.58 – 7.52 (m, 3H), 7.44 (s, 1H), 4.13 (s, 3H), 4.04 (s, 3H). ^13^C NMR (151 MHz, CDCl_3_) δ 160.4, 160.1, 156.0, 150.8, 131.7, 131.4, 131.1, 129.7, 128.5, 113.7, 110.3, 109.9, 105.9, 99.6, 56.6, 56.6. HRMS (ESI) m/z: [M + Na]^+^ Calcd for C_18_H_13_NO_4_SNa 362.0457; Found 362.0462.

**6,7-Dimethoxy-3-phenyl-4-selenocyanato-1*H*-isochromen-1-one (3c)**

The reaction was accomplished according to the general experimental procedure described above. Product **3c** was purified by flash column chromatography (silica gel, EtOAc/PE = 20/80). Yield: 71.1 mg, 92%, an orange solid, mp. 180 – 181 ^o^C. ^1^H NMR (400 MHz, CDCl_3_) δ 7.75 (s, 1H), 7.64 (dd, *J* = 7.7, 2.0 Hz, 2H), 7.58 – 7.48 (m, 3H), 7.43 (s, 1H), 4.12 (s, 3H), 4.04 (s, 3H). ^13^C NMR (101 MHz, CDCl_3_) δ 160.5, 159.8, 155.9, 150.7, 133.0, 132.2, 131.0, 129.8, 128.4, 113.7, 110.2, 108.4, 100.1, 99.5, 56.6, 56.6. ^77^Se NMR (76 MHz, CDCl_3_) δ 233.9. HRMS (ESI) m/z: [M + Na]^+^ Calcd for C_18_H_13_NO_4_SeNa 409.9902; Found 409.9905.

**7-Nitro-3-phenyl-4-thiocyanato-1*H*-isochromen-1-one (2d)**

The reaction was accomplished according to the general experimental procedure described above. Product **2d** was purified by flash column chromatography (silica gel, EtOAc/PE = 10/90). Yield: 36.3 mg, 56%, a yellow solid, mp. 188 – 190 ^o^C. ^1^H NMR (600 MHz, CDCl_3_) δ 9.22 (s, 1H), 8.75 (dd, *J* = 8.8, 2.4 Hz, 1H), 8.29 (d, *J* = 8.8 Hz, 1H), 7.83 – 7.72 (m, 2H), 7.64 (dd, *J* = 8.4, 6.4 Hz, 1H), 7.59 (t, *J* = 7.5 Hz, 2H). ^13^C NMR (151 MHz, CDCl_3_) δ 164.7, 158.2, 147.9, 140.8, 132.2, 130.7, 130.0, 129.7, 128.8, 126.7, 126.1, 121.3, 109.0, 99.5. HRMS (ESI) m/z: [M + Na]^+^ Calcd for C_16_H_8_N_2_O_4_SNa 347.0097; Found 347.0103.

**7-Nitro-3-phenyl-4-selenocyanato-1*H*-isochromen-1-one (3d)**

The reaction was accomplished according to the general experimental procedure described above. Product **3d** was purified by flash column chromatography (silica gel, EtOAc/PE = 20/80). Yield: 49.7 mg, 67%, a yellow solid, mp. 204 – 205 ^o^C. ^1^H NMR (400 MHz, CDCl_3_) δ 9.22 (d, *J* = 2.4 Hz, 1H), 8.73 (dd, *J* = 8.8, 2.4 Hz, 1H), 8.27 (d, *J* = 8.9 Hz, 1H), 7.72 – 7.67 (m, 2H), 7.63 – 7.54 (m, 3H). ^13^C NMR (101 MHz, CDCl_3_) δ 164.2, 158.6, 147.8, 141.5, 132.1, 132.0, 130.0, 129.8, 129.1, 128.7, 126.1, 121.3, 99.3, 98.9. ^77^Se NMR (76 MHz, CDCl_3_) δ 242.8. HRMS (ESI) m/z: [M + Na]^+^ Calcd for C_16_H_8_N_2_O_4_SeNa 394.9541; Found 394.9545.

**7-Chloro-3-phenyl-4-thiocyanato-1H-isochromen-1-one (2e)**

The reaction was accomplished according to the general experimental procedure described above. Product **2e** was purified by flash column chromatography (silica gel, EtOAc/PE = 10/90). Yield: 54.6 mg, 87%, a white solid, mp. 194 – 196 ^o^C. ^1^H NMR (400 MHz, CDCl_3_) δ 8.37 (t, *J* = 1.6 Hz, 1H), 8.06 (d, *J* = 8.6 Hz, 1H), 7.91 (ddd, *J* = 8.6, 2.3, 0.8 Hz, 1H), 7.76 – 7.71 (m, 2H), 7.60 – 7.53 (m, 3H). ^13^C NMR (151 MHz, CDCl_3_) δ 161.7, 158.9, 136.3, 136.0, 134.3, 131.5, 131.2, 129.9, 129.7, 128.6, 126.6, 121.8, 109.5, 99.7. HRMS (ESI) m/z: [M + Na]^+^ Calcd for C_16_H_8_ClNO_2_SNa 335.9856; Found 335.9867.

**7-Chloro-3-phenyl-4-selenocyanato-1*H*-isochromen-1-one (3e)**

The reaction was accomplished according to the general experimental procedure described above. Product **3e** was purified by flash column chromatography (silica gel, EtOAc/PE = 20/80). Yield: 64.9 mg, 90%, a white solid, mp. 218 – 220 ^o^C. ^1^H NMR (600 MHz, CDCl_3_) δ 8.36 (d, *J* = 2.3 Hz, 1H), 8.03 (d, *J* = 8.6 Hz, 1H), 7.88 (dd, *J* = 8.6, 2.3 Hz, 1H), 7.65 (dt, *J* = 6.8, 1.5 Hz, 2H), 7.59 – 7.52 (m, 3H). ^13^C NMR (151 MHz, CDCl_3_) δ 161.2, 159.3, 136.2, 135.9, 135.1, 132.6, 131.3, 129.7, 128.9, 128.5, 121.9, 99.6, 99.2. ^77^Se NMR (76 MHz, CDCl_3_) δ 235.9. HRMS (ESI) m/z: [M + Na]^+^ Calcd for C_16_H_8_ClNO_2_SeNa 383.9301; Found 383.9307.

**7-Fluoro-3-phenyl-4-thiocyanato-1*H*-isochromen-1-one (2f)**

The reaction was accomplished according to the general experimental procedure described above. Product **2f** was purified by flash column chromatography (silica gel, EtOAc/PE = 10/90). Yield: 50.5 mg, 85%, a yellow solid, mp. 196 – 198 ^o^C. ^1^H NMR (600 MHz, CDCl_3_) δ 8.12 (dd, *J* = 8.8, 4.7 Hz, 1H), 8.04 (d, *J* = 7.9 Hz, 1H), 7.72 (d, *J* = 7.3 Hz, 2H), 7.68 (d, *J* = 8.7 Hz, 1H), 7.56 (dd, *J* = 12.8, 7.0 Hz, 3H). ^13^C NMR (151 MHz, CDCl_3_) δ 162.7 (d, *J* = 253.6 Hz), 160.9 (d, *J* = 2.5 Hz), 159.2 (d, *J* = 3.3 Hz), 132.3 (d, *J* = 2.8 Hz), 131.4, 131.3, 129.7, 128.6, 127.7 (d, *J* = 7.8 Hz), 124.2 (d, *J* = 23.0 Hz), 122.5 (d, *J* = 8.3 Hz), 116.2 (d, *J* = 23.7 Hz), 109.6, 99.6. HRMS (ESI) m/z: [M + Na]^+^ Calcd for C_16_H_8_FNO_2_SNa 320.0152; Found 320.0157.

**7-Fluoro-3-phenyl-4-selenocyanato-1*H*-isochromen-1-one (3f)**

The reaction was accomplished according to the general experimental procedure described above. Product **3f** was purified by flash column chromatography (silica gel, EtOAc/PE = 20/80). Yield: 60.6 mg, 88%, a white solid, mp. 216 – 218 ^o^C. ^1^H NMR (400 MHz, CDCl_3_) δ 8.10 (dd, *J* = 8.9, 4.7 Hz, 1H), 8.06 (dd, *J* = 8.0, 2.8 Hz, 1H), 7.70 – 7.63 (m, 3H), 7.60 – 7.50 (m, 3H). ^13^C NMR (101 MHz, CDCl_3_) δ 162.7 (d, *J* = 253.2 Hz), 160.3 (d, *J* = 2.7 Hz), 159.6 (d, *J* = 3.3 Hz), 133.1 (d, *J* = 2.8 Hz), 132.5, 131.3, 130.0 (d, *J* = 8.0 Hz), 129.8, 128.5, 124.2 (d, *J* = 22.9 Hz), 122.4 (d, *J* = 8.4 Hz), 116.1 (d, *J* = 23.6 Hz), 99.9, 99.2. ^77^Se NMR (76 MHz, CDCl_3_) δ 237.1. HRMS (ESI) m/z: [M + Na]^+^ Calcd for C_16_H_8_FNO_2_SeNa 367.9596; Found 367.9601.

**4-Thiocyanato-3-(*p*-tolyl)-1*H*-isochromen-1-one (2g)**

The reaction was accomplished according to the general experimental procedure described above. Product **2g** was purified by flash column chromatography (silica gel, EtOAc/PE = 10/90). Yield: 55.1 mg, 94%, a white solid, mp. 241 – 242 ^o^C. ^1^H NMR (600 MHz, DMSO-*d*_6_) δ 8.3 – 8.3 (m, 1H), 8.1 – 8.1 (m, 2H), 7.8 (ddd, *J* = 8.1, 5.9, 2.5 Hz, 1H), 7.7 – 7.7 (m, 2H), 7.4 (d, *J* = 7.9 Hz, 2H), 2.4 (s, 3H). ^13^C NMR (151 MHz, DMSO-*d*_6_) δ = 160.6, 159.8, 141.0, 136.2, 135.7, 129.7, 129.6, 129.5, 129.0, 129.0, 124.7, 120.2, 111.3, 100.8, 21.1. HRMS (ESI) m/z: [M + Na]^+^ Calcd for C_17_H_11_NO_2_SNa 316.0403; Found 316.0409.

**4-Selenocyanato-3-(*p*-tolyl)-1*H*-isochromen-1-one (3g)**

The reaction was accomplished according to the general experimental procedure described above. Product **3g** was purified by flash column chromatography (silica gel, EtOAc/PE = 20/80). Yield: 63.2 mg, 93%, a white solid, mp. 247 – 248 ^o^C. ^1^H NMR (600 MHz, CDCl_3_) δ 8.4 (dd, *J* = 7.9, 1.4 Hz, 1H), 8.1 – 8.1 (m, 1H), 8.0 (td, *J* = 7.7, 1.4 Hz, 1H), 7.7 – 7.7 (m, 1H), 7.7 – 7.6 (m, 2H), 7.4 (d, *J* = 7.9 Hz, 2H), 2.5 (s, 3H). ^13^C NMR (101 MHz, CDCl_3_) δ 161.7, 160.2, 141.9, 136.0, 136.0, 130.4, 129.7, 129.6, 129.2, 128.7, 124.8, 120.5, 110.1, 99.6, 21.6. ^77^Se NMR (76 MHz, DMSO-*d*_6_) δ 234.6. HRMS (ESI) m/z: [M + Na]^+^ Calcd for C_17_H_11_NO_2_SeNa 363.9847; Found 363.9852.

**3-(4-Methoxyphenyl)-4-thiocyanato-1*H*-isochromen-1-one (2h)**

The reaction was accomplished according to the general experimental procedure described above. Product **2h** was purified by flash column chromatography (silica gel, EtOAc/PE = 10/90). Yield: 56.3 mg, 91%, a white solid, mp. 188 – 190 ^o^C. ^1^H NMR (400 MHz, DMSO-*d*_6_) δ 8.3 (dt, *J* = 8.0, 1.0 Hz, 1H), 8.2 – 8.1 (m, 2H), 7.8 – 7.7 (m, 3H), 7.2 – 7.1 (m, 2H), 3.9 (s, 3H). ^13^C NMR (101 MHz, DMSO-*d*_6_) δ 161.7, 160.9, 160.4, 136.7, 136.3, 131.9, 130.1, 130.0, 125.2, 124.4, 120.5, 114.4, 112.0, 100.7, 56.0. HRMS (ESI) m/z: [M + Na]^+^ Calcd for C_17_H_11_NO_3_SNa 332.0352; Found 332.0353.

**3-(4-Methoxyphenyl)-4-selenocyanato-1*H*-isochromen-1-one (3h)**

The reaction was accomplished according to the general experimental procedure described above. Product **3h** was purified by flash column chromatography (silica gel, EtOAc/PE = 20/80). Yield: 65.5 mg, 92%, a white solid, mp. 224– 226 ^o^C. ^1^H NMR (600 MHz, CDCl_3_) δ 8.4 (d, *J* = 7.8 Hz, 1H), 8.1 (d, *J* = 8.1 Hz, 1H), 7.9 – 7.9 (m, 1H), 7.7 – 7.6 (m, 3H), 7.0 (d, *J* = 8.7 Hz, 2H), 3.9 (s, 3H). ^13^C NMR (151 MHz, CDCl_3_) δ 161.8, 160.9, 160.6, 136.9, 135.9, 131.6, 130.3, 129.3, 127.1, 125.1, 120.6, 113.8, 100.1, 99.0, 55.5. ^77^Se NMR (76 MHz, CDCl_3_) δ 235.0. HRMS (ESI) m/z: [M + Na]^+^ Calcd for C_17_H_11_NO_3_SeNa 379.9796; Found 379.9803.

**3-(2-Methoxyphenyl)-4-thiocyanato-1*H*-isochromen-1-one (2i)**

The reaction was accomplished according to the general experimental procedure described above. Product **2i** was purified by flash column chromatography (silica gel, EtOAc/PE = 10/90). Yield: 53.8 mg, 87%, a white solid, mp. 174 – 176 ^o^C. ^1^H NMR (400 MHz, CDCl_3_) δ 8.4 (dd, *J* = 8.0, 1.4 Hz, 1H), 8.1 (d, *J* = 8.1 Hz, 1H), 7.9 (ddd, *J* = 8.1, 7.3, 1.4 Hz, 1H), 7.7 – 7.6 (m, 1H), 7.6 – 7.5 (m, 2H), 7.1 (td, *J* = 7.6, 1.0 Hz, 1H), 7.0 (d, *J* = 8.3 Hz, 1H), 3.9 (s, 3H). ^13^C NMR (101 MHz, CDCl_3_) δ 160.7, 157.9, 156.7, 135.9, 135.5, 132.8, 130.7, 130.4, 129.5, 124.6, 120.9, 120.8, 120.8, 111.2, 110.4, 103.0, 55.7. HRMS (ESI) m/z: [M + Na]^+^ Calcd for C_17_H_11_NO_3_SNa 332.0352; Found 332.0358.

**3-(2-Methoxyphenyl)-4-selenocyanato-1*H*-isochromen-1-one (3i)**

The reaction was accomplished according to the general experimental procedure described above. Product **3i** was purified by flash column chromatography (silica gel, EtOAc/PE = 20/80). Yield: 64.1 mg, 90%, a white solid, mp. 196 – 198 ^o^C. ^1^H NMR (400 MHz, CDCl_3_) δ 8.4 (dt, *J* = 7.9, 1.3 Hz, 1H), 8.1 – 8.0 (m, 1H), 7.9 (ddt, *J* = 8.2, 7.2, 1.3 Hz, 1H), 7.7 – 7.6 (m, 1H), 7.6 (dt, *J* = 7.7, 1.4 Hz, 1H), 7.5 (ddt, *J* = 8.6, 7.4, 1.4 Hz, 1H), 7.2 (tt, *J* = 7.6, 1.1 Hz, 1H), 7.0 (dd, *J* = 8.5, 1.1 Hz, 1H), 3.9 (s, 3H). ^13^C NMR (101 MHz, CDCl_3_) δ 161.2, 156.4, 155.4, 136.2, 135.8, 132.8, 130.8, 130.3, 129.4, 127.0, 122.0, 121.3, 120.8, 111.0, 103.3, 101.6, 55.8. ^77^Se NMR (76 MHz, CDCl_3_) δ 234.3. HRMS (ESI) m/z: [M + Na]^+^ Calcd for C_17_H_11_NO_3_SeNa 379.9796; Found 379.9803.

**3-(4-Chlorophenyl)-4-thiocyanato-1*H*-isochromen-1-one (2j)**

The reaction was accomplished according to the general experimental procedure described above. Product **2j** was purified by flash column chromatography (silica gel, EtOAc/PE = 10/90). Yield: 53.9 mg, 86%, a white solid, mp. 222 – 224 ^o^C. ^1^H NMR (600 MHz, CDCl_3_) δ 8.4 (dd, *J* = 7.9, 1.4 Hz, 1H), 8.1 – 8.1 (m, 1H), 8.0 (td, *J* = 7.8, 1.4 Hz, 1H), 7.7 – 7.7 (m, 3H), 7.6 – 7.5 (m, 2H). ^13^C NMR (101 MHz, CDCl_3_) δ 160.3, 159.9, 137.7, 136.2, 135.6, 131.0, 130.5, 130.0, 129.9, 129.0, 125.0, 120.6, 109.7, 100.5. HRMS (ESI) m/z: [M + Na]^+^ Calcd for C_16_H_8_ClNO_2_SNa 335.9856; Found 335.9859.

**3-(4-Chlorophenyl)-4-selenocyanato-1*H*-isochromen-1-one (3j)**

The reaction was accomplished according to the general experimental procedure described above. Product **3j** was purified by flash column chromatography (silica gel, EtOAc/PE = 20/80). Yield: 62.7 mg, 87%, a white solid, mp. 250 – 251 ^o^C. ^1^H NMR (600 MHz, DMSO-*d*_6_) δ 8.3 – 8.3 (m, 1H), 8.1 – 8.1 (m, 2H), 7.8 – 7.8 (m, 2H), 7.8 (ddd, *J* = 8.2, 7.0, 1.5 Hz, 1H), 7.7 – 7.7 (m, 2H). ^13^C NMR (151 MHz, DMSO-*d*_6_) δ 160.1, 158.1, 136.7, 136.3, 135.4, 132.2, 131.6, 129.8, 129.5, 128.3, 127.5, 120.1, 104.6, 103.4. ^77^Se NMR (76 MHz, DMSO-*d*_6_) δ 232.7. HRMS (ESI) m/z: [M + Na]^+^ Calcd for C_16_H_8_ClNO_2_SeNa 383.9301; Found 383.9305.

**3-(3,4-dichlorophenyl)-4-thiocyanato-1*H*-isochromen-1-one (2k)**

The reaction was accomplished according to the general experimental procedure described above. Product **2k** was purified by flash column chromatography (silica gel, EtOAc/PE = 10/90). Yield: 64.1 mg, 92%, a white solid, mp. 178 – 180 ^o^C. ^1^H NMR (400 MHz, CDCl_3_) δ 8.4 (ddd, *J* = 7.9, 1.4, 0.6 Hz, 1H), 8.1 (dt, *J* = 8.0, 1.0 Hz, 1H), 8.0 (ddd, *J* = 8.1, 7.3, 1.4 Hz, 1H), 7.9 (dd, *J* = 2.0, 0.5 Hz, 1H), 7.7 (ddd, *J* = 8.4, 7.3, 1.1 Hz, 1H), 7.7 – 7.6 (m, 2H). ^13^C NMR (151 MHz, CDCl_3_) δ 159.5, 158.9, 136.2, 136.0, 135.3, 133.3, 131.5, 131.2, 130.7, 130.6, 130.2, 128.8, 125.1, 120.7, 109.2, 101.2. HRMS (ESI) m/z: [M + Na]^+^ Calcd for C_16_H_7_Cl_2_NO_2_SNa 369.9467; Found 369.9474.

**3-(3,4-Dichlorophenyl)-4-selenocyanato-1*H*-isochromen-1-one (3k)**

The reaction was accomplished according to the general experimental procedure described above. Product **3k** was purified by flash column chromatography (silica gel, EtOAc/PE = 20/80). Yield: 71.1 mg, 90%, a white solid, mp. 229 – 230 ^o^C. ^1^H NMR (600 MHz, CDCl_3_) δ 8.4 (dd, *J* = 7.9, 1.4 Hz, 1H), 8.1 – 8.1 (m, 1H), 8.0 (ddd, *J* = 8.2, 7.4, 1.4 Hz, 1H), 7.8 (d, *J* = 2.1 Hz, 1H), 7.7 (ddd, *J* = 8.3, 7.4, 1.1 Hz, 1H), 7.6 (d, *J* = 8.3 Hz, 1H), 7.5 (dd, *J* = 8.3, 2.1 Hz, 1H). ^13^C NMR (151 MHz, CDCl_3_) δ 159.8, 158.5, 136.2, 136.1, 135.8, 133.1, 132.5, 131.6, 130.5, 130.5, 130.1, 129.0, 127.4, 120.8, 100.8, 99.3. ^77^Se NMR (76 MHz, CDCl_3_) δ 230.5. HRMS (ESI) m/z: [M + Na]^+^ Calcd for C_16_H_7_Cl_2_NO_2_SeNa 417.8911; Found 417.8915.

**Methyl 2-(1-oxo-4-thiocyanato-1*H*-isochromen-3-yl)benzoate (2l)**

The reaction was accomplished according to the general experimental procedure described above. Product **2l** was purified by flash column chromatography (silica gel, EtOAc/PE = 10/90). Yield: 62.7 mg, 93%, a white solid, mp. 172 – 174 ^o^C. ^1^H NMR (600 MHz, CDCl_3_) δ 8.4 – 8.4 (m, 1H), 8.2 (dd, *J* = 7.9, 1.4 Hz, 1H), 8.1 – 8.0 (m, 1H), 8.0 – 7.9 (m, 1H), 7.7 (td, *J* = 7.6, 1.4 Hz, 1H), 7.7 (dtd, *J* = 12.0, 7.6, 1.2 Hz, 2H), 7.6 (dd, *J* = 7.6, 1.3 Hz, 1H), 3.8 (s, 3H). ^13^C NMR (151 MHz, CDCl_3_) δ = 165.5, 162.1, 160.1, 135.9, 135.5, 133.1, 132.7, 131.1, 130.9, 130.6, 130.6, 129.6, 129.5, 124.5, 120.7, 109.4, 100.8, 52.7. HRMS (ESI) m/z: [M + Na]^+^ Calcd for C_18_H_11_NO_4_SNa 360.0301; Found 360.0305.

**Methyl 2-(1-oxo-4-selenocyanato-1*H*-isochromen-3-yl)benzoate (3l)**

The reaction was accomplished according to the general experimental procedure described above. Product **3l** was purified by flash column chromatography (silica gel, EtOAc/PE = 20/80). Yield: 70.8 mg, 92%, a white solid, mp. 174 – 176 ^o^C. ^1^H NMR (600 MHz, CDCl_3_) δ 8.4 (dd, *J* = 8.0, 1.4 Hz, 1H), 8.1 (dd, *J* = 7.9, 1.3 Hz, 1H), 8.1 – 8.0 (m, 1H), 7.9 (td, *J* = 7.8, 1.4 Hz, 1H), 7.7 (td, *J* = 7.6, 1.4 Hz, 1H), 7.7 (dtd, *J* = 15.4, 7.9, 1.2 Hz, 2H), 7.6 (dd, *J* = 7.6, 1.3 Hz, 1H), 3.8 (s, 3H). ^13^C NMR (151 MHz, CDCl_3_) δ 166.0, 160.6, 160.5, 136.3, 136.0, 134.4, 132.7, 130.8, 130.8, 130.7, 130.5, 129.5, 129.4, 126.8, 120.8, 101.4, 100.2, 52.7. ^77^Se NMR (76 MHz, CDCl_3_) δ 217.9. HRMS (ESI) m/z: [M + Na]^+^ Calcd for C_18_H_11_NO_4_SeNa 407.9746; Found 407.9750.

**3-(4-Nitrophenyl)-4-thiocyanato-1*H*-isochromen-1-one (2m)**

The reaction was accomplished according to the general experimental procedure described above. Product **2m** was purified by flash column chromatography (silica gel, EtOAc/PE = 10/90). Yield: 49.3 mg, 76%, a yellow solid, mp. 212 – 213 ^o^C. ^1^H NMR (400 MHz, CDCl_3_) δ 8.5 – 8.4 (m, 3H), 8.1 (d, *J* = 8.1 Hz, 1H), 8.0 (td, *J* = 7.8, 1.4 Hz, 1H), 8.0 – 7.9 (m, 2H), 7.8 (t, *J* = 7.6 Hz, 1H). ^13^C NMR (101 MHz, CDCl_3_) δ 159.3, 158.8, 149.1, 137.3, 136.4, 135.1, 130.9, 130.7, 130.6, 125.2, 123.8, 120.8, 109.1, 102.1. HRMS (ESI) m/z: [M + Na]^+^ Calcd for C_16_H_8_N_2_O_4_SNa 347.0097; Found 347.0101.

**3-(4-Nitrophenyl)-4-selenocyanato-1*H*-isochromen-1-one (3m)**

The reaction was accomplished according to the general experimental procedure described above. Product **3m** was purified by flash column chromatography (silica gel, EtOAc/PE = 20/80). Yield: 60.8 mg, 82%, a yellow solid, mp. 196 – 198 ^o^C. ^1^H NMR (400 MHz, CDCl_3_) δ 8.4 – 8.3 (m, 3H), 8.1 (d, *J* = 8.1 Hz, 1H), 8.0 (t, *J* = 7.8 Hz, 1H), 7.9 (d, *J* = 8.4 Hz, 2H), 7.7 (t, *J* = 7.6 Hz, 1H). ^13^C NMR (101 MHz, CDCl_3_) δ 159.7, 158.5, 149.1, 138.6, 136.4, 135.9, 131.0, 130.6, 130.5, 127.5, 123.7, 120.8, 101.5, 99.3. ^77^Se NMR (76 MHz, CDCl_3_) δ 229.3. HRMS (ESI) m/z: [M + Na]^+^ Calcd for C_16_H_8_N_2_O_4_SeNa 394.9541; Found 394.9545.

**6-Phenyl-7-thiocyanato-4*H*-thieno[3,2-c]pyran-4-one (2n)**

The reaction was accomplished according to the general experimental procedure described above. Product **2n** was purified by flash column chromatography (silica gel, EtOAc/PE = 10/90). Yield: 49.6 mg, 87%, a yellow solid, mp. 184 – 186 ^o^C. ^1^H NMR (600 MHz, CDCl_3_) δ 8.0 (d, *J* = 5.1 Hz, 1H), 7.7 (dt, *J* = 6.6, 1.6 Hz, 2H), 7.6 (d, *J* = 5.1 Hz, 1H), 7.6 – 7.5 (m, 3H). ^13^C NMR (151 MHz, CDCl_3_) δ 162.6, 156.0, 147.1, 138.1, 131.4, 130.7, 129.7, 128.6, 124.8, 123.2, 109.4, 97.9. HRMS (ESI) m/z: [M + Na]^+^ Calcd for C_14_H_7_NO_2_S_2_Na 307.9810; Found 307.9814.

**6-Phenyl-7-selenocyanato-4*H*-thieno[3,2-c]pyran-4-one (3n)**

The reaction was accomplished according to the general experimental procedure described above. Product **3n** was purified by flash column chromatography (silica gel, EtOAc/PE = 20/80). Yield: 58.5 mg, 88%, a white solid, mp. 209 – 210 ^o^C. ^1^H NMR (600 MHz, CDCl_3_) δ 8.0 (d, *J* = 5.2 Hz, 1H), 7.7 – 7.6 (m, 2H), 7.6 (d, *J* = 5.2 Hz, 1H), 7.5 (ddd, *J* = 14.4, 7.8, 6.2 Hz, 3H). ^13^C NMR (101 MHz, CDCl_3_) δ 162.1, 156.5, 148.0, 137.6, 131.8, 131.3, 129.7, 128.6, 126.5, 123.0, 99.8, 95.8. ^77^Se NMR (76 MHz, CDCl_3_) δ 262.9. HRMS (ESI) m/z: [M + Na]^+^ Calcd for C_14_H_7_NO_2_SSeNa 355.9255; Found 355.9259.

**3-Cyclopropyl-4-thiocyanato-1*H*-isochromen-1-one (2o)**

The reaction was accomplished according to the general experimental procedure described above. Product **2o** was purified by flash column chromatography (silica gel, EtOAc/PE = 10/90). Yield: 44.7 mg, 92%, a white solid, mp. 161 – 162 ^o^C. ^1^H NMR (600 MHz, CDCl_3_) δ 8.3 (dd, *J* = 7.9, 1.4 Hz, 1H), 8.0 (dd, *J* = 8.1, 1.1 Hz, 1H), 7.9 (td, *J* = 8.2, 7.7, 1.4 Hz, 1H), 7.6 (td, *J* = 7.6, 1.1 Hz, 1H), 2.7 (tt, *J* = 8.2, 4.9 Hz, 1H), 1.4 – 1.4 (m, 2H), 1.2 – 1.2 (m, 2H). ^13^C NMR (151 MHz, CDCl_3_) δ 165.4, 159.8, 136.1, 135.9, 130.3, 128.4, 123.6, 119.9, 109.6, 97.6, 13.4, 9.5. HRMS (ESI) m/z: [M + Na]^+^ Calcd for C_13_H_9_NO_2_SNa 266.0246; Found 266.0250.

**3-Cyclopropyl-4-selenocyanato-1*H*-isochromen-1-one (3o)**

The reaction was accomplished according to the general experimental procedure described above. Product **3o** was purified by flash column chromatography (silica gel, EtOAc/PE = 20/80). Yield: 55.7 mg, 96%, a white solid, mp. 180 – 182 ^o^C. ^1^H NMR (600 MHz, CDCl_3_) δ 8.3 (dd, *J* = 7.9, 1.4 Hz, 1H), 7.9 (dd, *J* = 8.2, 1.1 Hz, 1H), 7.8 (ddd, *J* = 8.2, 7.3, 1.4 Hz, 1H), 7.6 – 7.5 (m, 1H), 2.7 (tt, *J* = 8.2, 4.9 Hz, 1H), 1.4 – 1.3 (m, 2H), 1.2 – 1.1 (m, 2H). ^13^C NMR (151 MHz, CDCl_3_) δ 164.4, 160.1, 137.0, 135.9, 130.2, 128.4, 125.8, 120.0, 99.6, 97.7, 15.3, 9.5. ^77^Se NMR (76 MHz, CDCl_3_) δ 187.4. HRMS (ESI) m/z: [M + Na]^+^ Calcd for C_13_H_9_NO_2_SeNa 313.9691; Found 313.9697.

**4-Thiocyanato-3-(thiophen-2-yl)-1*H*-isochromen-1-one (2p**)

The reaction was accomplished according to the general experimental procedure described above. Product **2p** was purified by flash column chromatography (silica gel, EtOAc/PE = 10/90). Yield: 49.0 mg, 86%, a white solid, mp. 170 – 171 ^o^C. ^1^H NMR (400 MHz, CDCl_3_) δ 8.4 (dd, *J* = 7.9, 1.4 Hz, 1H), 8.2 – 8.1 (m, 2H), 7.9 (ddd, *J* = 8.4, 7.4, 1.4 Hz, 1H), 7.7 (dd, *J* = 5.0, 1.3 Hz, 1H), 7.6 (td, *J* = 7.6, 1.1 Hz, 1H), 7.2 (dd, *J* = 5.1, 3.9 Hz, 1H). ^13^C NMR (101 MHz, CDCl_3_) δ 159.4, 155.3, 136.4, 136.1, 133.4, 132.8, 132.6, 130.5, 129.3, 127.8, 124.9, 120.2, 109.1, 96.3. HRMS (ESI) m/z: [M + Na]^+^ Calcd for C_14_H_7_NO_2_S_2_Na 307.9810; Found 307.9816.

**4-Selenocyanato-3-(thiophen-2-yl)-1*H*-isochromen-1-one (3p)**

The reaction was accomplished according to the general experimental procedure described above. Product **3p** was purified by flash column chromatography (silica gel, EtOAc/PE = 20/80). Yield: 59.8 mg, 90%, a yellow solid, mp. 183 – 184 ^o^C. ^1^H NMR (600 MHz, CDCl_3_) δ 8.3 (dd, *J* = 7.9, 1.4 Hz, 1H), 8.1 (d, *J* = 8.3 Hz, 1H), 8.0 (dd, *J* = 3.8, 1.2 Hz, 1H), 7.9 (ddd, *J* = 8.3, 7.3, 1.4 Hz, 1H), 7.7 (dd, *J* = 5.1, 1.3 Hz, 1H), 7.6 (td, *J* = 7.6, 1.1 Hz, 1H), 7.2 (dd, *J* = 5.1, 3.8 Hz, 1H). ^13^C NMR (151 MHz, CDCl_3_) δ 159.7, 154.7, 137.1, 136.0, 133.7, 133.3, 131.9, 130.3, 129.3, 127.6, 127.4, 120.4, 99.4, 97.3, 77.2, 77.0, 76.8. ^77^Se NMR (76 MHz, CDCl_3_) δ 217.2. HRMS (ESI) m/z: [M + Na]^+^ Calcd for C_14_H_7_NO_2_SSeNa 355.9255; Found 355.9260.

**3-(Naphthalen-1-yl)-4-thiocyanato-1*H*-isochromen-1-one (2q)**

The reaction was accomplished according to the general experimental procedure described above. Product **2q** was purified by flash column chromatography (silica gel, EtOAc/PE = 10/90). Yield: 54.0 mg, 82%, a white solid, mp. 138 – 140 ^o^C. ^1^H NMR (400 MHz, CDCl_3_) δ 8.5 (dd, *J* = 8.0, 1.3 Hz, 1H), 8.1 (dd, *J* = 8.1, 1.1 Hz, 1H), 8.1 (dt, *J* = 8.3, 1.1 Hz, 1H), 8.0 – 8.0 (m, 2H), 7.8 – 7.7 (m, 2H), 7.7 (dd, *J* = 7.2, 1.3 Hz, 1H), 7.6 – 7.5 (m, 3H). ^13^C NMR (101 MHz, CDCl_3_) δ 161.2, 160.3, 136.2, 135.3, 133.5, 131.5, 130.8, 130.7, 130.0, 129.2, 128.9, 128.5, 127.9, 126.9, 125.0, 124.9, 124.4, 120.9, 109.7, 103.4. HRMS (ESI) m/z: [M + Na]^+^ Calcd for C_20_H_11_NO_2_SNa 352.0403; Found 352.0406.

**3-(Naphthalen-1-yl)-4-selenocyanato-1*H*-isochromen-1-one (3q)**

The reaction was accomplished according to the general experimental procedure described above. Product **2q** was purified by flash column chromatography (silica gel, EtOAc/PE = 20/80). Yield: 63.2 mg, 84%, a white solid, mp. 169 – 170 ^o^C. ^1^H NMR (400 MHz, DMSO-*d*_6_) δ 8.3 (dt, *J* = 7.9, 1.1 Hz, 1H), 8.2 (dt, *J* = 8.3, 1.1 Hz, 1H), 8.2 – 8.1 (m, 2H), 8.1 – 8.1 (m, 1H), 7.9 (dq, *J* = 8.1, 0.9 Hz, 1H), 7.9 – 7.8 (m, 2H), 7.7 (dd, *J* = 8.3, 7.0 Hz, 1H), 7.6 (dddd, *J* = 19.6, 8.3, 6.9, 1.4 Hz, 2H). ^13^C NMR (101 MHz, DMSO-*d*_6_) δ 160.9, 159.5, 137.0, 136.7, 133.4, 131.4, 131.1, 130.9, 130.3, 130.1, 129.4, 128.9, 127.8, 127.8, 127.1, 125.8, 125.6, 121.1, 106.0, 105.0. ^77^Se NMR (76 MHz, CDCl_3_) δ 227.7. HRMS (ESI) m/z: [M + Na]^+^ Calcd for C_20_H_11_NO_2_SeNa 399.9847; Found 399.9853.

**4-Thiocyanato-3-(trimethylsilyl)-1*H*-isochromen-1-one (2r)**

The reaction was accomplished according to the general experimental procedure described above. Product **2r** was purified by flash column chromatography (silica gel, EtOAc/PE = 10/90). Yield: 44.0 mg, 80%, a white solid, mp. 103 – 104 ^o^C. ^1^H NMR (600 MHz, CDCl_3_) δ 8.3 (ddd, *J* = 7.9, 1.3, 0.7 Hz, 1H), 8.0 – 7.9 (m, 2H), 7.7 – 7.6 (m, 1H), 0.5 (s, 9H). ^13^C NMR (151 MHz, CDCl_3_) δ 174.0, 162.1, 136.6, 135.6, 131.2, 131.1, 124.9, 122.6, 113.1, 110.7, 0.0. HRMS (ESI) m/z: [M + Na]^+^ Calcd for C_13_H_13_NO_2_SSiNa 298.0328; Found 298.0334.

**4-Selenocyanato-3-(trimethylsilyl)-1*H*-isochromen-1-one (3r)**

The reaction was accomplished according to the general experimental procedure described above. Product **3r** was purified by flash column chromatography (silica gel, EtOAc/PE = 20/80). Yield: 52.8 mg, 82%, a white solid, mp. 130 – 131 ^o^C. ^1^H NMR (400 MHz, CDCl_3_) δ 8.3 (dt, *J* = 7.7, 1.0 Hz, 1H), 7.9 – 7.9 (m, 2H), 7.7 (ddd, *J* = 8.3, 6.7, 1.8 Hz, 1H), 0.5 (s, 9H). ^13^C NMR (101 MHz, CDCl_3_) δ 172.6, 161.4, 135.7, 135.4, 130.2, 130.1, 126.1, 121.9, 111.8, 99.9, -0.5. ^77^Se NMR (76 MHz, CDCl_3_) δ 205.9. HRMS (ESI) m/z: [M + Na]^+^ Calcd for C_13_H_13_NO_2_SeSiNa 345.9773; Found 345.9779.

**4-Thiocyanato-1*H*-isochromen-1-one (2s)**

The reaction was accomplished according to the general experimental procedure described above. Product **2s** was purified by flash column chromatography (silica gel, EtOAc/PE = 10/90). Yield: 34.1 mg, 84%, a white solid, mp. 136 – 138 ^o^C. ^1^H NMR (400 MHz, CDCl_3_) δ 8.3 (dd, *J* = 7.9, 0.9 Hz, 1H), 8.0 (dt, *J* = 7.7, 1.0 Hz, 1H), 7.9 (td, *J* = 7.7, 1.2 Hz, 1H), 7.7 (td, *J* = 7.5, 0.9 Hz, 1H), 6.4 (s, 1H). ^13^C NMR (151 MHz, CDCl_3_) δ 164.7, 153.8, 136.0, 135.3, 132.4, 126.3, 126.3, 125.3, 108.6, 91.8. HRMS (ESI) m/z: [M + Na]^+^ Calcd for C_10_H_5_NO_2_SNa 225.9933; Found 225.9939.

**4-Selenocyanato-1*H*-isochromen-1-one (3s)**

The reaction was accomplished according to the general experimental procedure described above. Product **3s** was purified by flash column chromatography (silica gel, EtOAc/PE = 20/80). Yield: 43.5 mg, 87%, a white solid, mp. 140 – 141 ^o^C. ^1^H NMR (600 MHz, CDCl_3_) δ 8.4 (d, *J* = 7.9 Hz, 1H), 8.0 (d, *J* = 7.7 Hz, 1H), 7.9 – 7.8 (m, 1H), 7.7 (t, *J* = 7.5 Hz, 1H), 6.6 (s, 1H). ^13^C NMR (151 MHz, CDCl_3_) δ 164.8, 153.6, 136.5, 135.1, 132.2, 126.7, 126.3, 125.4, 98.6, 88.1. ^77^Se NMR (76 MHz, CDCl_3_) δ 204.8. HRMS (ESI) m/z: [M + Na]^+^ Calcd for C_10_H_5_NO_2_SeNa 273.9378; Found 273.9384.

**3-Butyl-4-thiocyanato-1*H*-isochromen-1-one (2t)**

The reaction was accomplished according to the general experimental procedure described above. Product **2t** was purified by flash column chromatography (silica gel, EtOAc/PE = 10/90). Yield: 48.7 mg, 94%, a white solid, mp. 57 – 58 ^o^C. ^1^H NMR (600 MHz, CDCl_3_) δ 8.4 – 8.3 (m, 1H), 8.0 (dd, *J* = 8.1, 1.1 Hz, 1H), 7.9 (ddt, *J* = 8.0, 7.1, 1.0 Hz, 1H), 7.6 (ddd, *J* = 8.1, 7.2, 1.0 Hz, 1H), 3.0 – 3.0 (m, 2H), 1.8 – 1.8 (m, 2H), 1.5 (dt, *J* = 14.8, 7.4 Hz, 2H), 1.0 – 1.0 (m, 3H). ^13^C NMR (151 MHz, CDCl_3_) δ 166.0, 160.3, 135.9, 135.5, 130.4, 129.1, 124.1, 120.3, 109.4, 99.6, 33.0, 29.5, 22.3, 13.7. HRMS (ESI) m/z: [M + Na]^+^ Calcd for C_14_H_13_NO_2_SNa 282.0559; Found 282.0565.

**3-Butyl-4-selenocyanato-1*H*-isochromen-1-one (3t)**

The reaction was accomplished according to the general experimental procedure described above. Product **3t** was purified by flash column chromatography (silica gel, EtOAc/PE = 20/80). Yield: 58.2 mg, 95%, a white solid, mp. 64 – 65 ^o^C. ^1^H NMR (400 MHz, CDCl_3_) δ 8.3 (dd, *J* = 8.0, 1.3 Hz, 1H), 8.0 – 7.9 (m, 1H), 7.9 (ddd, *J* = 8.1, 7.2, 1.4 Hz, 1H), 7.6 (ddd, *J* = 8.3, 7.3, 1.2 Hz, 1H), 3.1 – 3.0 (m, 2H), 1.9 – 1.7 (m, 2H), 1.5 (h, *J* = 7.4 Hz, 2H), 1.0 (t, *J* = 7.4 Hz, 3H). ^13^C NMR (101 MHz, CDCl_3_) δ 165.1, 160.8, 136.4, 135.9, 130.3, 129.0, 126.4, 120.3, 99.6, 99.4, 34.9, 29.9, 22.4, 13.8. ^77^Se NMR (76 MHz, CDCl_3_) δ 195.2. HRMS (ESI) m/z: [M + Na]^+^ Calcd for C_14_H_13_NO_2_SeNa 330.0004; Found 330.0009.

**7-Propyl-8-thiocyanato-5*H*-[1,3]dioxolo[4,5-*g*]isochromen-5-one (2u)**

The reaction was accomplished according to the general experimental procedure described above. Product **2u** was purified by flash column chromatography (silica gel, EtOAc/PE = 10/90). Yield: 1.02 g, 87%, a white solid, mp. 160 – 162 ^o^C. ^1^H NMR (600 MHz, CDCl_3_) δ 7.6 (s, 1H), 7.4 (s, 1H), 6.2 (s, 2H), 3.0 – 2.9 (m, 2H), 1.8 (h, *J* = 7.4 Hz, 2H), 1.0 (t, *J* = 7.4 Hz, 3H). ^13^C NMR (151 MHz, CDCl_3_) δ 164.8, 159.9, 154.8, 148.9, 133.5, 115.0, 109.4, 108.2, 103.4, 102.8, 99.5, 35.0, 21.0, 13.6. HRMS (ESI) m/z: [M + Na]^+^ Calcd for C_14_H_11_NO_4_SNa 312.0301; Found 312.0307.

**7-Propyl-8-selenocyanato-5*H*-[1,3]dioxolo[4,5-*g*]isochromen-5-one (3u)**

The reaction was accomplished according to the general experimental procedure described above. Product **2u** was purified by flash column chromatography (silica gel, EtOAc/PE = 20/80). Yield: 1.23 g, 90%, a white solid, mp. 168 – 170 ^o^C. ^1^H NMR (600 MHz, CDCl_3_) δ 7.6 (s, 1H), 7.3 (s, 1H), 6.1 (s, 2H), 3.0 – 2.9 (m, 2H), 1.8 – 1.7 (m, 2H), 1.0 (t, *J* = 7.4 Hz, 3H). ^13^C NMR (151 MHz, CDCl_3_) δ 163.9, 160.3, 154.7, 148.8, 134.4, 115.0, 108.0, 105.7, 102.8, 99.4, 99.4, 36.8, 21.3, 13.6. ^77^Se NMR (76 MHz, CDCl_3_) δ 204.4. HRMS (ESI) m/z: [M + Na]^+^ Calcd for C_14_H_11_NO_4_SeNa 359.9746; Found 359.9750.

**7-Propyl-8-((trifluoromethyl)thio)-5*H*-[1,3]dioxolo[4,5-*g*]isochromen-5-one (2v)**

The reaction was accomplished according to the reported experimental procedure.^1^ Product **2v** was purified by flash column chromatography (silica gel, EtOAc/PE = 5/95). Yield: 49.8 mg, 75%, a grey solid, mp. 149 – 150 ^o^C.^1^H NMR (400 MHz, CDCl_3_) δ 7.6 (s, 1H), 7.4 (s, 1H), 6.1 (s, 2H), 3.0 (s, 2H), 1.8 (h, *J* = 7.5 Hz, 2H), 1.0 (t, *J* = 7.4 Hz, 3H). ^13^C NMR (101 MHz, CDCl_3_) δ 166.7, 160.5, 154.4, 148.5, 135.8, 129.0 (q, *J* = 311.5 Hz), 114.8, 107.5, 104.2, 102.6, 100.3, 100.3, 34.6, 20.9, 13.7. ^19^F NMR (376 MHz, CDCl_3_) δ -42.6. HRMS (ESI) m/z: [M + Na]^+^ Calcd for C_14_H_11_F_3_O_4_SNa 355.0222; Found 355.0228.

**7-Propyl-8-((trifluoromethyl)selanyl)-5*H*-[1,3]dioxolo[4,5-*g*]isochromen-5-one (3v)**

The reaction was accomplished according to the reported experimental procedure.^1^ Product **3v** was purified by flash column chromatography (silica gel, EtOAc/PE = 5/95). Yield: 68.4 mg, 90%, a grey solid, mp. 160 – 161 ^o^C. ^1^H NMR (400 MHz, CDCl_3_) δ 7.6 (s, 1H), 7.5 (s, 1H), 6.1 (s, 2H), 3.1 – 3.0 (m, 2H), 1.8 (h, *J* = 7.4 Hz, 2H), 1.0 (t, *J* = 7.4 Hz, 3H). ^13^C NMR (151 MHz, CDCl_3_) δ = 165.2, 160.8, 154.4, 148.4, 136.2, 121.9 (q, *J* = 336.7 Hz), 114.8, 107.5, 106.3, 102.5, 100.7, 36.5, 21.2, 13.6. ^19^F NMR (376 MHz, CDCl_3_) δ = -35.6. ^77^Se NMR (76 MHz, CDCl_3_) δ 413.7. HRMS (ESI) m/z: [M + Na]^+^ Calcd for C_14_H_11_F_3_O_4_SeNa 402.9667; Found 402.9673.

**8-((2*H*-Tetrazol-5-yl)thio)-7-propyl-5*H*-[1,3]dioxolo[4,5-*g*]isochromen-5-one (2w)**

The reaction was accomplished according to the reported experimental procedure.^1^ Product **2w**, Yield: 62.5 mg, 94%, a white solid, mp. 210 – 211 ^o^C. ^1^H NMR (400 MHz, DMSO-*d*_6_) δ 7.6 (s, 1H), 7.2 (s, 1H), 6.2 (s, 2H), 3.4 (br, 1H), 2.9 – 2.8 (m, 2H), 1.7 (h, *J* = 7.4 Hz, 2H), 0.9 (t, *J* = 7.4 Hz, 3H). ^13^C NMR (101 MHz, DMSO-*d*_6_) δ 164.6, 160.4, 154.7, 154.0, 148.8, 135.1, 115.0, 107.1, 103.6, 103.4, 102.5, 34.5, 21.0, 13.9. HRMS (ESI) m/z: [M + Na]^+^ Calcd for C_14_H_12_N_4_O_4_SNa 355.0471; Found 355.0475.

**8-((2*H*-Tetrazol-5-yl)selanyl)-7-propyl-5*H*-[1,3]dioxolo[4,5-*g*]isochromen-5-one (3w)**

The reaction was accomplished according to the reported experimental procedure.^1^ Product **3w**, Yield: 72.8 mg, 96%, a grey solid, mp. 220– 221 ^o^C. ^1^H NMR (400 MHz, DMSO-*d*_6_) δ 7.5 (s, 1H), 7.3 (s, 1H), 6.2 (s, 2H), 3.4 (br, 1H), 3.0 – 2.9 (m, 2H), 1.7 – 1.6 (m, 2H), 0.9 (t, *J* = 7.4 Hz, 3H). ^13^C NMR (101 MHz, DMSO-*d*_6_) δ 163.2, 160.8, 154.6, 148.7, 146.3, 136.0, 114.9, 106.9, 105.8, 103.5, 102.3, 36.5, 21.2, 13.9. ^77^Se NMR (76 MHz, DMSO-*d*_6_) δ 205.4. HRMS (ESI) m/z: [M + Na]^+^ Calcd for C_14_H_12_N_4_O_4_SeNa 402.9916; Found 402.9920.

# III Control Experiments

**Supplementary Scheme 1.** Control experiments.

**Supplementary Figure 1.** GS-MS spetrum of BnCl_2_

To shed light on the possible reaction mechanism, several control experiments were carried out (Supplementary Scheme S1). It was found that with the introduction of 2 equivalents of TEMPO to the reaction mixture, the reaction was almost hampered and only trace amount of the desired products **2a**/**3a** could be detected. However, this result could not corroborate that this thio/selenocyantion reaction proceed through a radical process, as it has been reported that TEMPO was incompatible with the oxidative reaction system.^2^ Replacement of TEMPO with BHT led to the completely opposite result and the desired products **2a** or **3a** were obtained in 94% and 90% yields, respectively. In addition, when (1-cyclopropylvinyl)benzene **1x**^3^ was applied as a radical-clock substrate, no ring-opening products **2x** or **3x** was obtained. These results are consistent with our proposal that the reactive species generated in the reaction system might be consumed by TEMPO and the present reaction might involve an ionic pathway rather than a radical pathway. The intramolecular cyclization process was also investigated with benzyl *o*-alkynylbenzoate **1y** under standard reaction conditions. In addition to the corresponding C4 thio/selenocyanated products **2a** and **3a** being obtained in 92% and 94% yields, BnCl was also isolated in 65% and 70% yields, respectively. These results suggested that the removal of benzyl group should be assisted by the nucleophilic attack of chloride ion during the cyclization process. Finnally, it is worth noting that the acyclic dithiocyanate product **2a’** (in the yield of 20%) would be generated in the reaction when the dosage of PhICl_2_ and NH_4_SCN was adjusted to 2 equiv and 4 equiv respectively. It can be concluded that thiocyanagen *in situ* generated in the reaction system would attack the alkynyl and afford the bridged intermediate and SCN anion, then both the carbonyl group and SCN anion undergo nucleophilic attack at the bridged intermediate, generating the resulting cyclic compounds **2a** and acyclic dithiocyanate compound **2a’**.


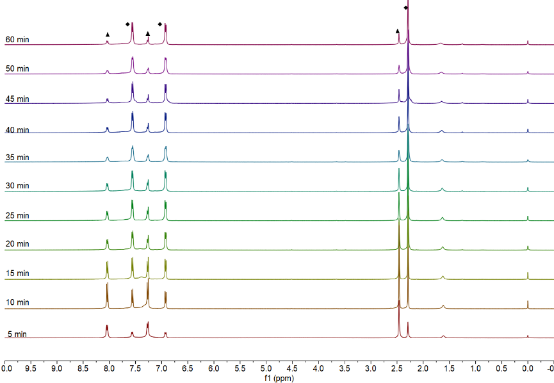


**Supplementary Figure 2.** ^1^H NMR study of reaction mechanism.

First, we measured the ^1^H NMR spectrum of a 1:1 mixture of **4** and NH_4_SCN (Supplementary Figure 2). At the initial stage, both **4** and **5** were clearly observed. The amount of **4** continued to decrease over the time period of 60 min (Figure S2), while that of **5** gradually increased and no byproducts were detected by ^1^H NMR analysis of the crude mixture. Based on those results, we speculate that there is no hypervalent iodine reagent intermediates generated in the reaction system.


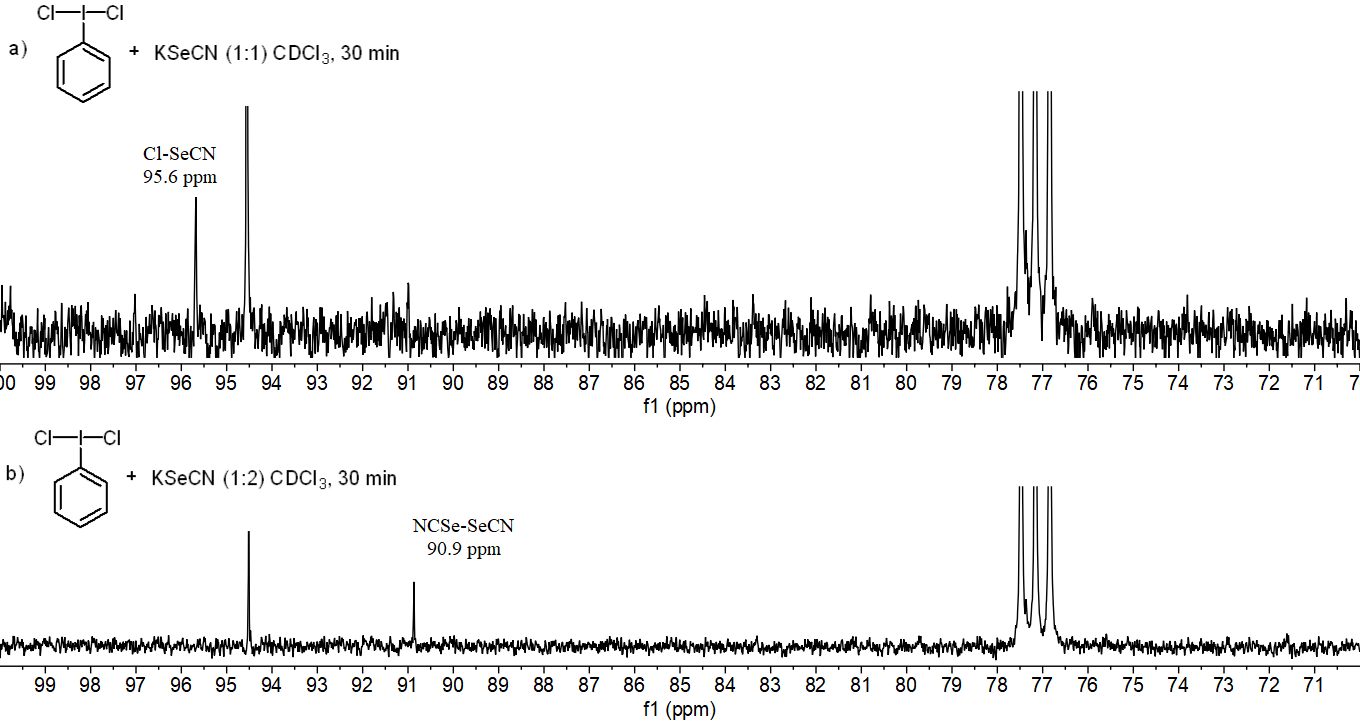


**Supplementary Figure 3.** ^13^C NMR study of selenocyanation mechanism.

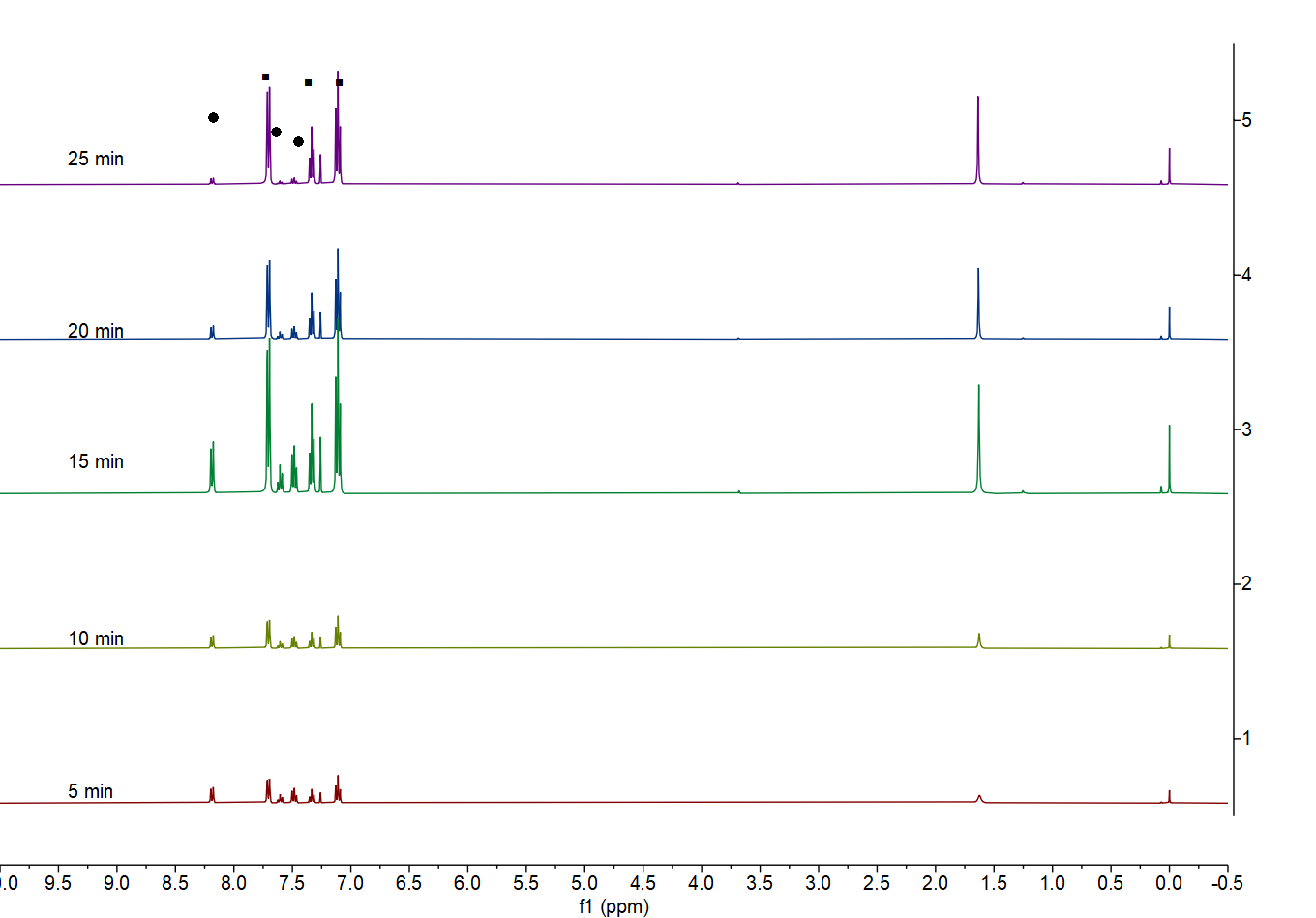


**Supplementary Figure 4.** ^1^H NMR study of selenocyanation mechanism

Following the similar procedures,^4^ we also carried out the control experiments to identify the formation of (SeCN)_2_ and Cl-SeCN intermediates: (1) the ^13^C NMR spectrum collected from the reaction mixture of 1 equiv of PhICl_2_ with 1 equiv of KSeCN in CDCl_3_ at rt for 30 min displays the peak of 95.6 ppm, which can be attributed to the carbon signal in Cl-SeCN; (2) the ^13^C NMR spectrum collected from the reaction mixture of 1 equiv of PhICl_2_ with 2 equiv of KSeCN in CDCl_3_ at rt for 30 min displays the peak of 90.9 ppm , which can be assigned to the carbon in NCSe-SeCN. These results imply that both the oxythiocyanation and oxyselenocyanation of *o*-1-(alkynyl)benzoates should follow the same mechanistic pathway. In order to further corroborate whether PhI(SeCN)_2_ species were indeed formed, the reaction of PhICl_2_ with KSeCN in CDCl_3_ was carried out respectively and ^1^H NMR analysis was implemented. The outcome revealed that only PhICl_2_ and PhI could be observed in the ^1^H NMR spectrum and PhI(SeCN)_2_ was not formed after the addition of KSeCN into PhICl_2_.

**Computational calculation**

All calculations were performed using density functional theory (DFT) by *Gaussian16* software package, with B3LYP exchange-correlation functional. The basis set of 3-21G for all atoms was used for structural optimizations and frequency calculations. The polarizable continuum model (PCM) with DCE as solvent was used unless specially mentioned. The contribution of Gibbs free energy involving the zero-point energy (ZPE) was calculated at the temperature of 323.15K and the pressure of 1 atm. Total energies (Gibbs free energy) in Hartree and Cartesian coordinates in Å are given for each structure as the following.


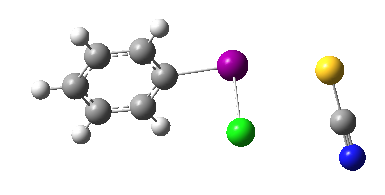

Total Energy = -8067.373524 Hartree

Cartesian coordinates:

C 2.30391300 0.69364100 -1.13313500

C 1.78476400 -0.13108700 -0.13149200

C 2.58770300 -0.64904300 0.88818400

C 3.95058800 -0.34964200 0.88666000

C 4.48904400 0.46598300 -0.11443900

C 3.66804000 0.98522900 -1.12194400

H 1.66486200 1.09706900 -1.90758800

H 2.16644800 -1.27475200 1.66397600

H 4.58607100 -0.75003300 1.66659200

H 5.54631800 0.70040200 -0.10734700

H 4.08530800 1.61722700 -1.89585200

Cl -0.66040400 1.61600800 1.20868100

C -3.84087300 0.64493700 -0.41855400

N -4.25774100 1.62242000 -0.92006300

I -0.27843900 -0.71006000 -0.22131800

S -3.24498300 -0.78412000 0.31695400


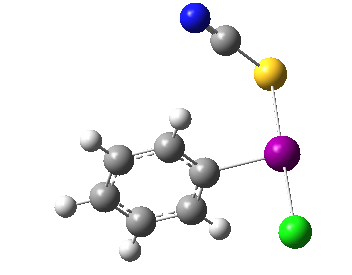

Total Energy = -8067.405902 Hartree

Cartesian coordinates:

C 0.99124500 -1.53187900 1.25326500

C 0.66723000 -0.80880700 0.10839500

C 1.46484300 -0.79963900 -1.03329300

C 2.64274400 -1.55246300 -1.01842100

C 2.99190600 -2.29034700 0.11517900

C 2.17040000 -2.28070700 1.24657000

H 0.35391300 -1.51951200 2.12675800

H 1.19362800 -0.22473400 -1.90781600

H 3.27839700 -1.55762000 -1.89481000

H 3.90439000 -2.87339800 0.11784500

H 2.44075500 -2.85162200 2.12590600

I -1.14482300 0.35024900 0.09566800

Cl -2.60448800 -1.87665300 -0.62213500

C 1.38263000 2.71896400 -0.49823100

N 2.11136700 2.77048800 -1.41464800

S 0.32095500 2.64016400 0.86248700


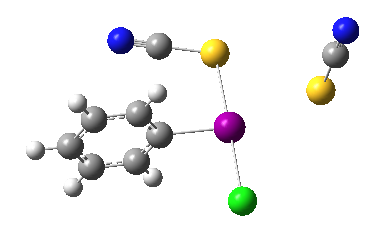

Total Energy = -8556.128158 Hartree

Cartesian coordinates:

C -2.54331500 -0.26990600 -1.04707200

C -1.71210700 -0.26904100 0.07224300

C -2.22092500 -0.14791300 1.36449100

C -3.60277100 -0.03213300 1.53720500

C -4.45101700 -0.03520200 0.42655300

C -3.92336400 -0.15299200 -0.86250300

H -2.13210500 -0.36287500 -2.04401900

H -1.56170300 -0.14725100 2.22302000

H -4.00988600 0.06070500 2.53659100

H -5.52147200 0.05566000 0.56510800

H -4.57930700 -0.15400000 -1.72441900

Cl 0.10891500 -3.28354000 -0.03226400

C -0.89772800 2.93286400 -0.09530100

N -1.97365300 3.35931800 0.09519700

C 4.11144100 0.37022600 0.85400600

N 4.54974400 0.43684800 1.94328400

I 0.42525900 -0.49321500 -0.20460000

S 0.69702800 2.32471200 -0.37458500

S 3.47923800 0.27277100 -0.74610900


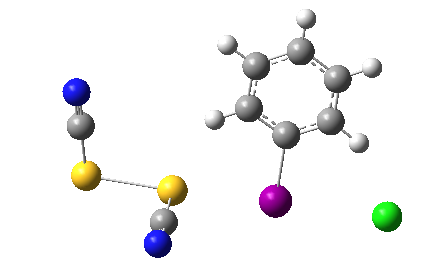

Total Energy = -8556.138935 Hartree

Cartesian coordinates:

C 0.26581600 1.91871100 0.08322200

C 1.36831500 1.07466700 -0.00339400

C 2.67327300 1.55432000 0.01893300

C 2.87388400 2.93486300 0.13381000

C 1.78463100 3.80480800 0.22348800

C 0.48393800 3.29608600 0.19773000

H -0.74071200 1.52428400 0.06061100

H 3.50757400 0.86408900 -0.04697800

H 3.88623000 3.32120300 0.15331700

H 1.94744700 4.87220000 0.31316200

H -0.36793500 3.96215500 0.26662000

Cl 4.34954300 -1.47031700 -0.15911400

C -2.14396200 -1.23314300 1.40064700

N -2.25564600 -1.47813700 2.53889400

C -4.70009600 1.05704600 -0.34967200

N -4.81985300 2.20220400 -0.12706900

I 1.05891300 -1.07604100 -0.17866200

S -1.98148700 -0.87193900 -0.29625400

S -4.54373500 -0.62999800 -0.68400100


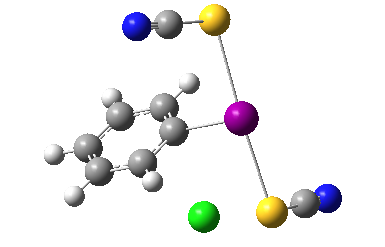

Total Energy = -8556.122248 Hartree

Cartesian coordinates:

C 0.47104900 1.80183500 0.93206400

C 0.49524500 1.12153500 -0.27730500

C 0.94305700 1.68673100 -1.46704700

C 1.40158500 3.00669400 -1.43248700

C 1.39610700 3.72177900 -0.23061500

C 0.93118700 3.12423500 0.94336600

H 0.08040100 1.32360600 1.82896200

H 0.94652900 1.12829300 -2.39318500

H 1.75639100 3.46911300 -2.34536600

H 1.75164700 4.74490100 -0.21218300

H 0.92196400 3.67804100 1.87422500

I -0.20725500 -0.92853100 -0.30595500

C -3.51790800 -0.07360700 -1.34614200

N -3.98572800 -0.04216800 -2.42112100

C 3.36711200 -1.44255500 0.32352600

N 4.01465700 -1.20623800 1.27221300

Cl -1.23954200 -0.20746700 3.18214600

S 2.44178500 -1.79724700 -1.08988500

S -2.84974500 -0.11187200 0.26094000


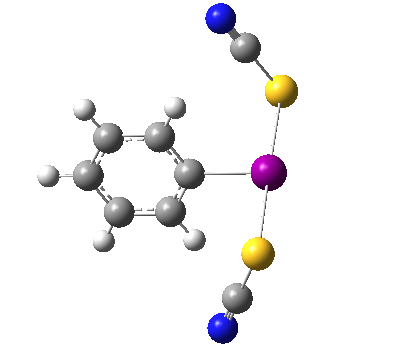

Total Energy = -8097.943876 Hartree

Cartesian coordinates:

C -0.72891000 1.68305800 -0.98313400

C -0.00014900 1.01830200 -0.00007900

C 0.72834800 1.68333700 0.98297900

C 0.72537300 3.08103500 0.97093600

C -0.00074400 3.77568400 -0.00004200

C -0.72655100 3.08075500 -0.97105000

H -1.29647100 1.14443000 -1.72893300

H 1.29623900 1.14493400 1.72869100

H 1.28941700 3.61752100 1.72345900

H -0.00097900 4.85853300 -0.00001600

H -1.29079600 3.61701700 -1.72358100

I 0.00022900 -1.13756800 -0.00013400

C 3.46017300 -0.39336600 0.19614700

N 4.00257900 0.14006800 1.08870400

C -3.46023000 -0.39424500 -0.19564700

N -4.00320800 0.13908200 -1.08792000

S -2.65203100 -1.16453300 1.12058600

S 2.65271700 -1.16376300 -1.12050200


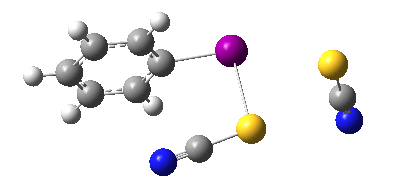

Total Energy = -8097.920731 Hartree

Cartesian coordinates:

C -2.49823800 -0.54259100 -1.06084600

C -1.65098900 -0.57113000 0.05146300

C -2.14810500 -0.49518300 1.35654200

C -3.52862300 -0.42308200 1.54729600

C -4.38992400 -0.41253800 0.44532900

C -3.87630100 -0.47029100 -0.85474300

H -2.09752700 -0.58687900 -2.06514700

H -1.47856800 -0.50363500 2.20676700

H -3.92653400 -0.37328800 2.55306100

H -5.46029000 -0.35385900 0.59908900

H -4.54344900 -0.45726800 -1.70751500

I 0.45663600 -0.86472300 -0.24966700

C -0.84769500 2.56698700 -0.05984200

N -1.94145600 2.95019500 0.13067600

C 3.97400900 -0.05213900 0.88410000

N 4.38989800 -0.00308100 1.98179100

S 3.37890700 -0.12300500 -0.72704800

S 0.74364100 1.99020800 -0.33526100


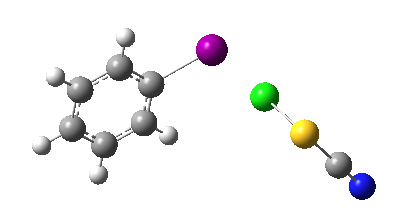

Total Energy = -8067.417827 Hartree

Cartesian coordinates:

C -2.25511200 0.90418000 1.25423400

C -2.04939300 0.18747100 0.07632500

C -2.66438500 0.55567200 -1.11951800

C -3.50933000 1.66944200 -1.13023900

C -3.72844000 2.39880900 0.04126600

C -3.10197300 2.01617600 1.23017800

H -1.76918700 0.60908500 2.17452500

H -2.49353400 -0.00810900 -2.02690200

H -3.99141900 1.96182600 -2.05526700

H -4.38340000 3.26140800 0.02752800

H -3.26694600 2.57814900 2.14148200

Cl 1.89130100 -0.11100000 -0.46903300

C 4.68204200 0.93171000 0.60570900

N 5.19963400 0.86657600 1.65424700

I -0.75507100 -1.53312100 0.10622700

S 3.94582600 1.04333000 -0.95283200

# IV Biological Experiments

**
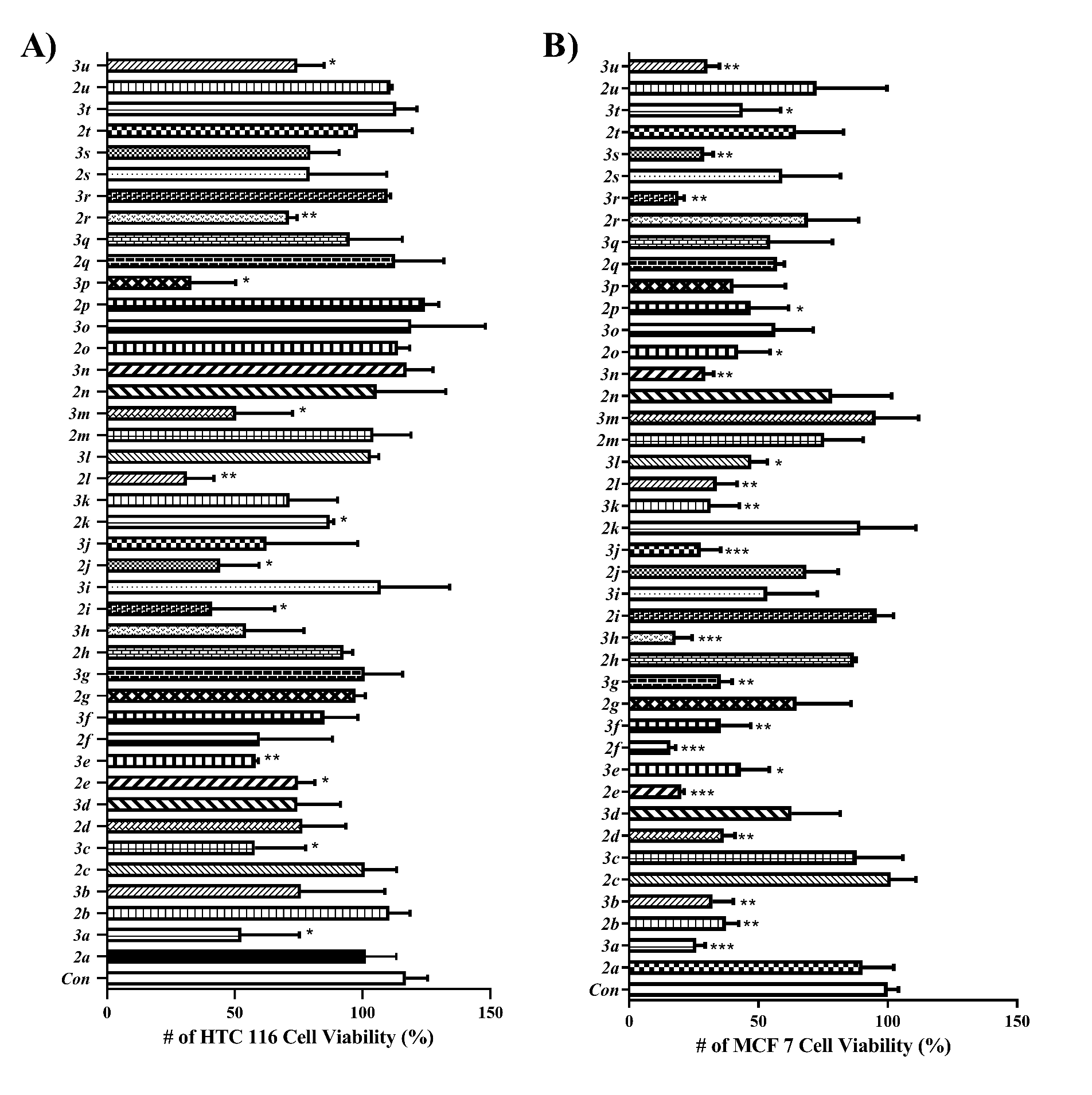
**

**Supplementary Figure 5.** Antitumor activity of synthesized 4-thio/selenocyanated isocoumarins against HCT 116 (A) and MCF 7 (B) cell lines, determined by a CCK-8 assay.

**Cell lines and culture conditions**

HCT 116 and MCF 7 cells were maintained in Dulbecco’s Modified Eagle’s Medium (DMEM) medium (Corning, Manassas, VA, United States) with 10% fetal bovine serum (Biological Industries, Cromwell, CT, United States) and 100 U/mL penicillin/streptomycin (Solarbio, Beijing, China) in thermostatic incubator with 5% CO_2_.

**Cell viability assay (CCK-8)**

All the tested 4-thio/selenocyanated isocoumarins were evaluated *in vitro* for their antitumor activity against HCT 116 and MCF 7 cell lines by cell counting kit-8 (CCK-8) assay (Biosharp, China) according to the manufacturer’s instructions. Briefly, exponentially growing cells were harvested and plated in 96-well plates at a concentration of 5 x 10^3^ cells per well. After 24 h incubation, the cells were respectively treated with target compounds at the concentration of 10 μM (the concentration of DMSO was kept below 0.1%, which was found to be non-toxic to the cells) for 48 h. Then, cells were further incubated with CCK-8 solution for 2 h at 37 ^o^C. The absorbance was measured at 450 nm and 650 nm (reference) with a microplate reader (TECAN, Switzerland).

Cell viability was calculated by the equation: Cell viability (%) = [A _Drug+_ – A _Black]_ / [A _Drug-_ – A _Black_] x 100%

A _Drug+_ : OD value of wells with cells, CCK-8 and drugs;

A _Drug-_ : OD value of wells with cells, CCK-8, but without drugs;

A _Black_: OD value of wells with culture medium and CCK-8, but without cells.

# V Reference

[1] Z. Liang, F. Wang, P. Chen, G. Liu, *Org. Lett.* **2015**, *17*, 2438-2441.

[2] a) P. Lucio Anelli, C. Biffi, F. Montanari, S. Quici, *J. Org. Chem.* **1987**, *52*, 2559-2562; b) C. I. Herrerías, T. Y. Zhang, C.-J. Li, *Tetrahedron Lett.* **2006**, *47*, 13-17.

[3] L.-H. Lu, S.-J. Zhou, W.-B. He, W. Xia, P. Chen, X. Yu, X. Xu, W.-M. He, *Org. Biomol. Chem.* **2018**, *16*, 9064-9068.

[4] S. Tao, J. Xiao, Y. Li, F. Sun, Y. Du, *Chin. J. Chem .* **2021**, *39*, 2536-2546.

# VI NMR Spectra of Product


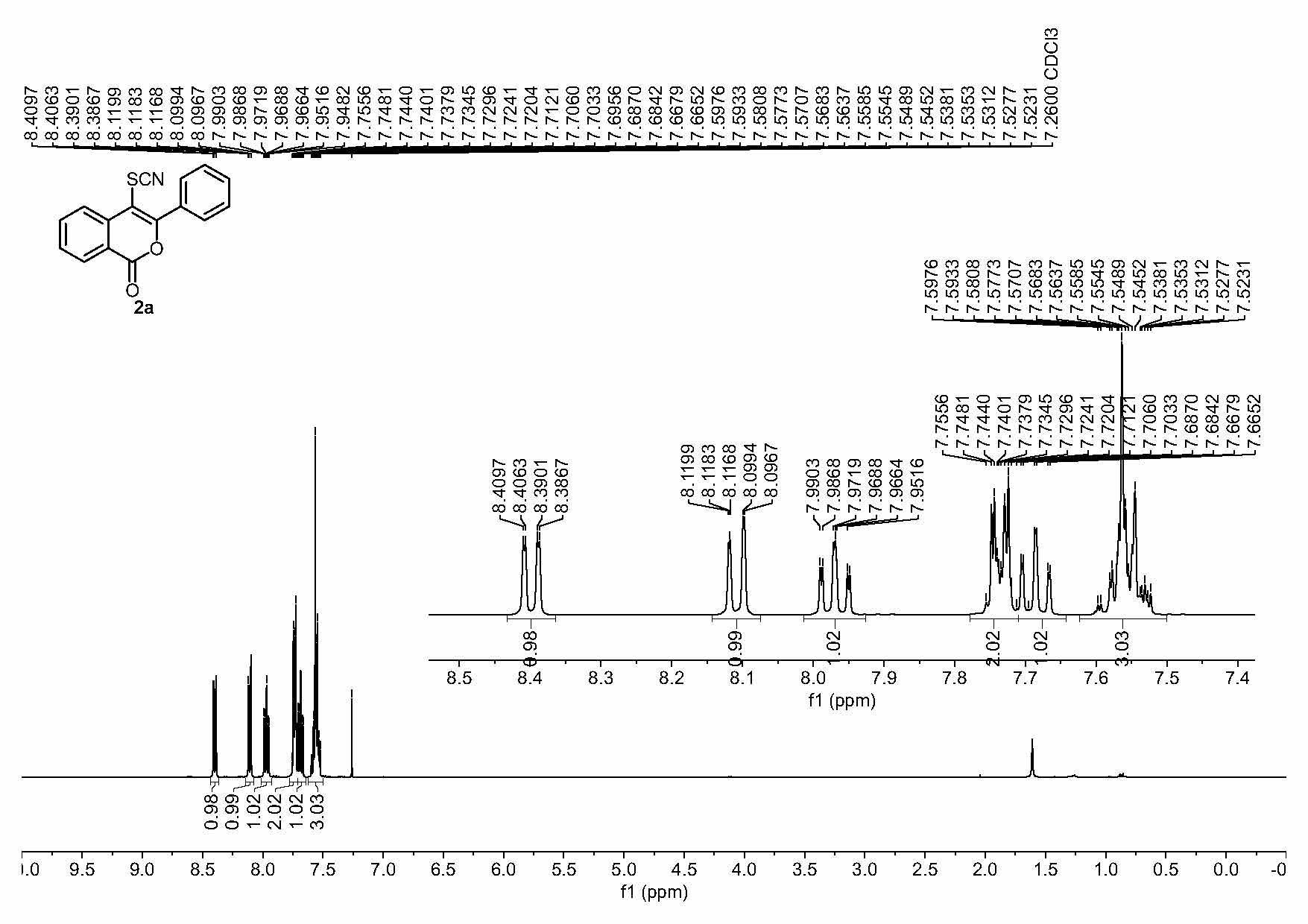


#
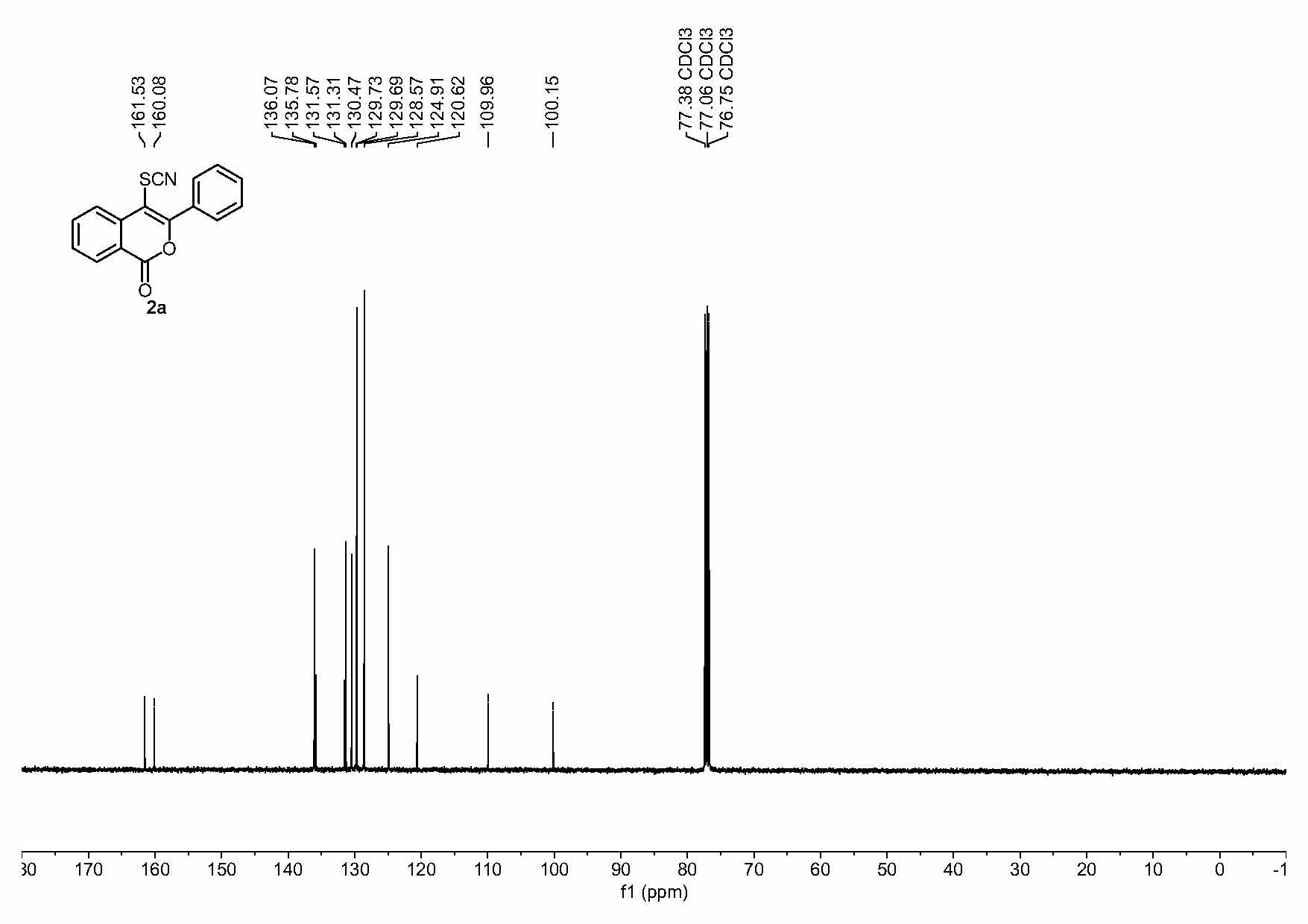

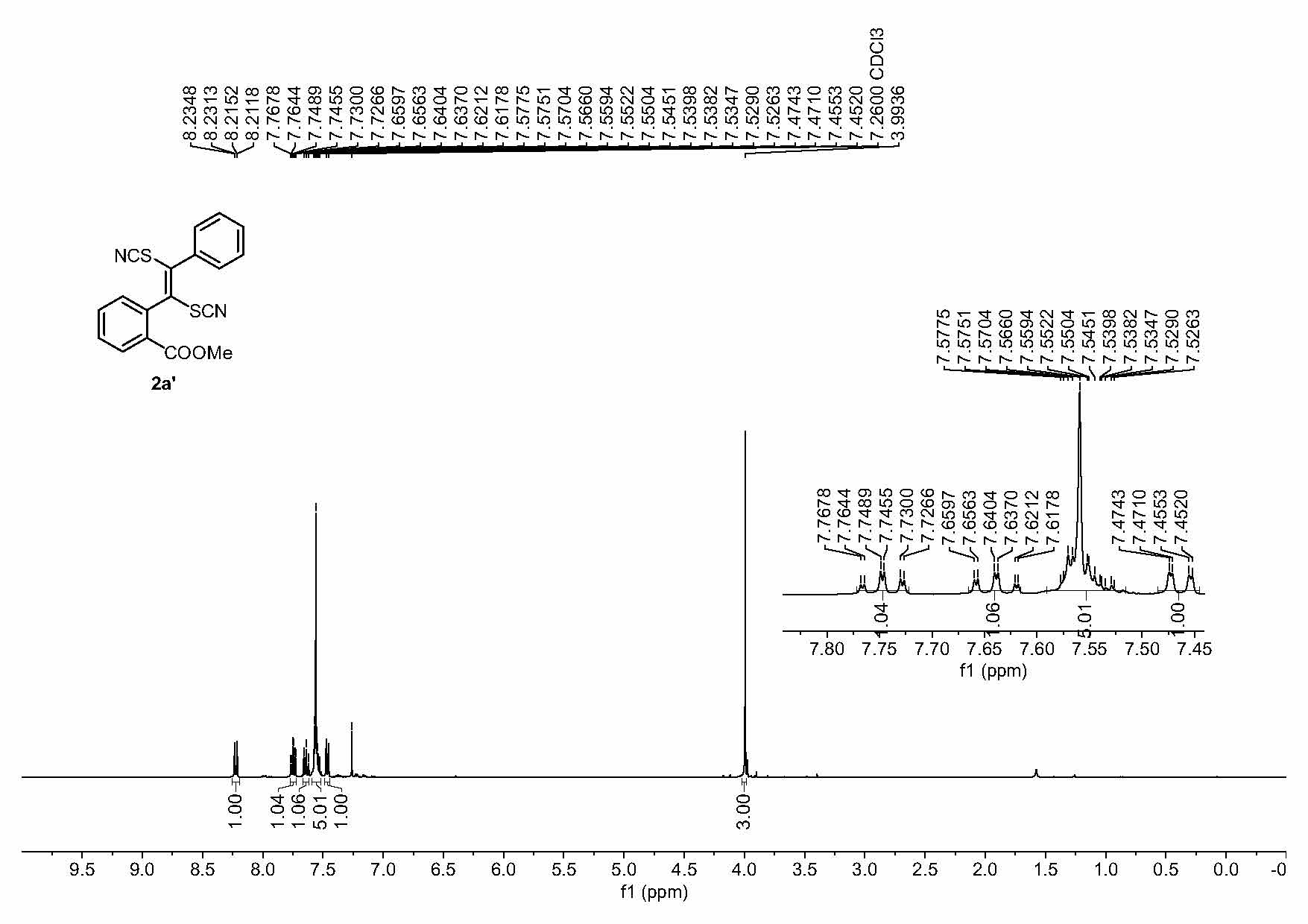

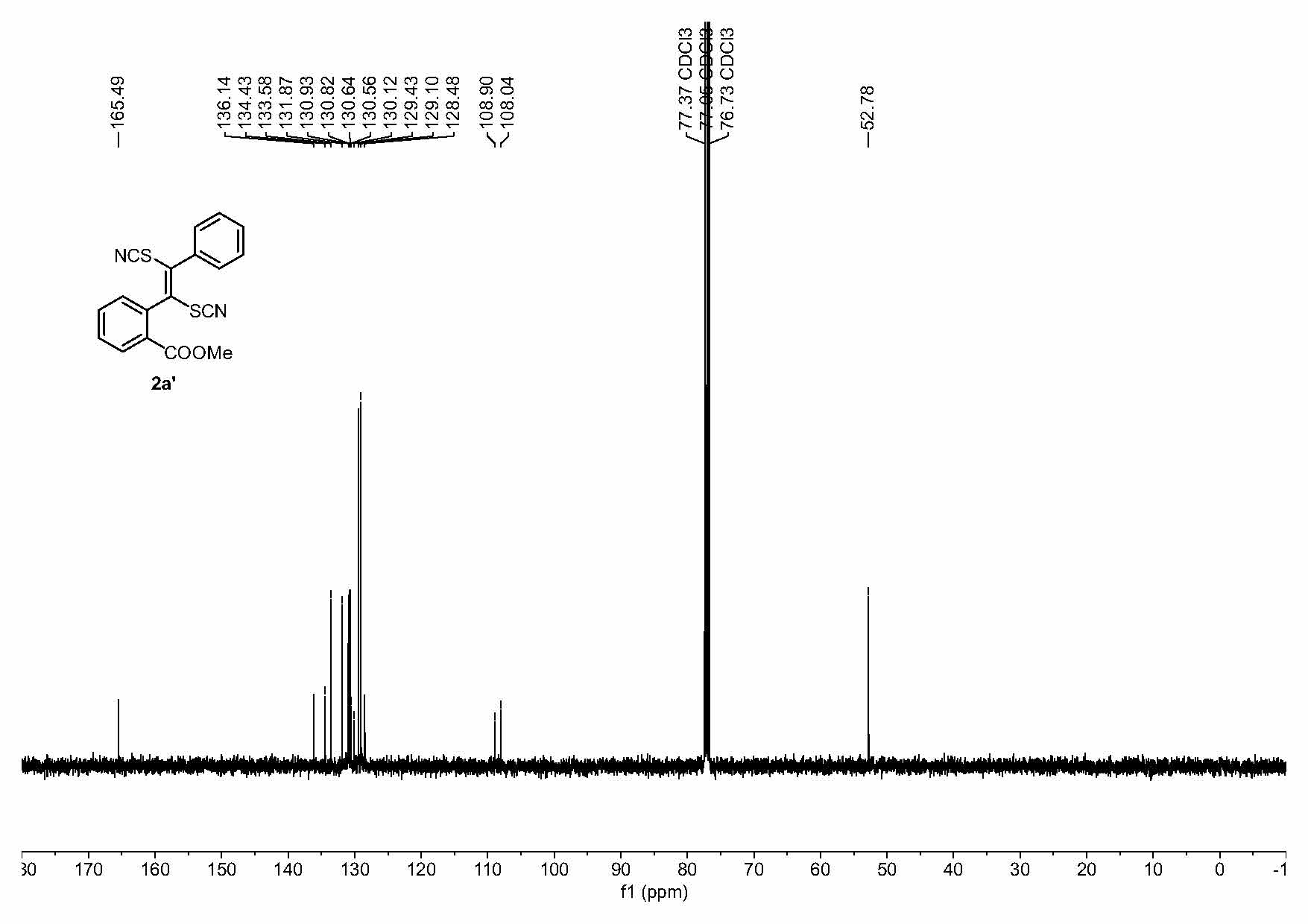

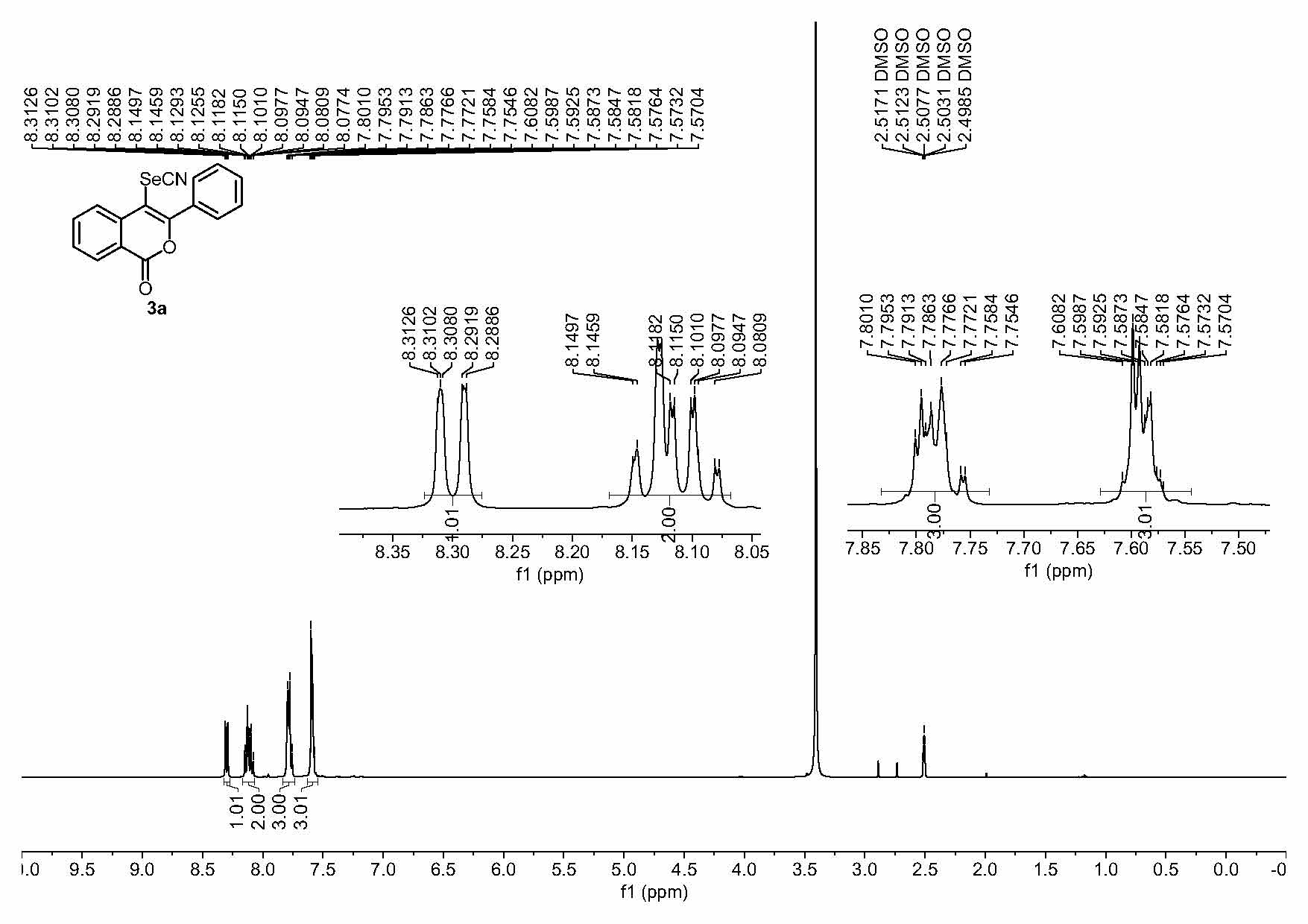

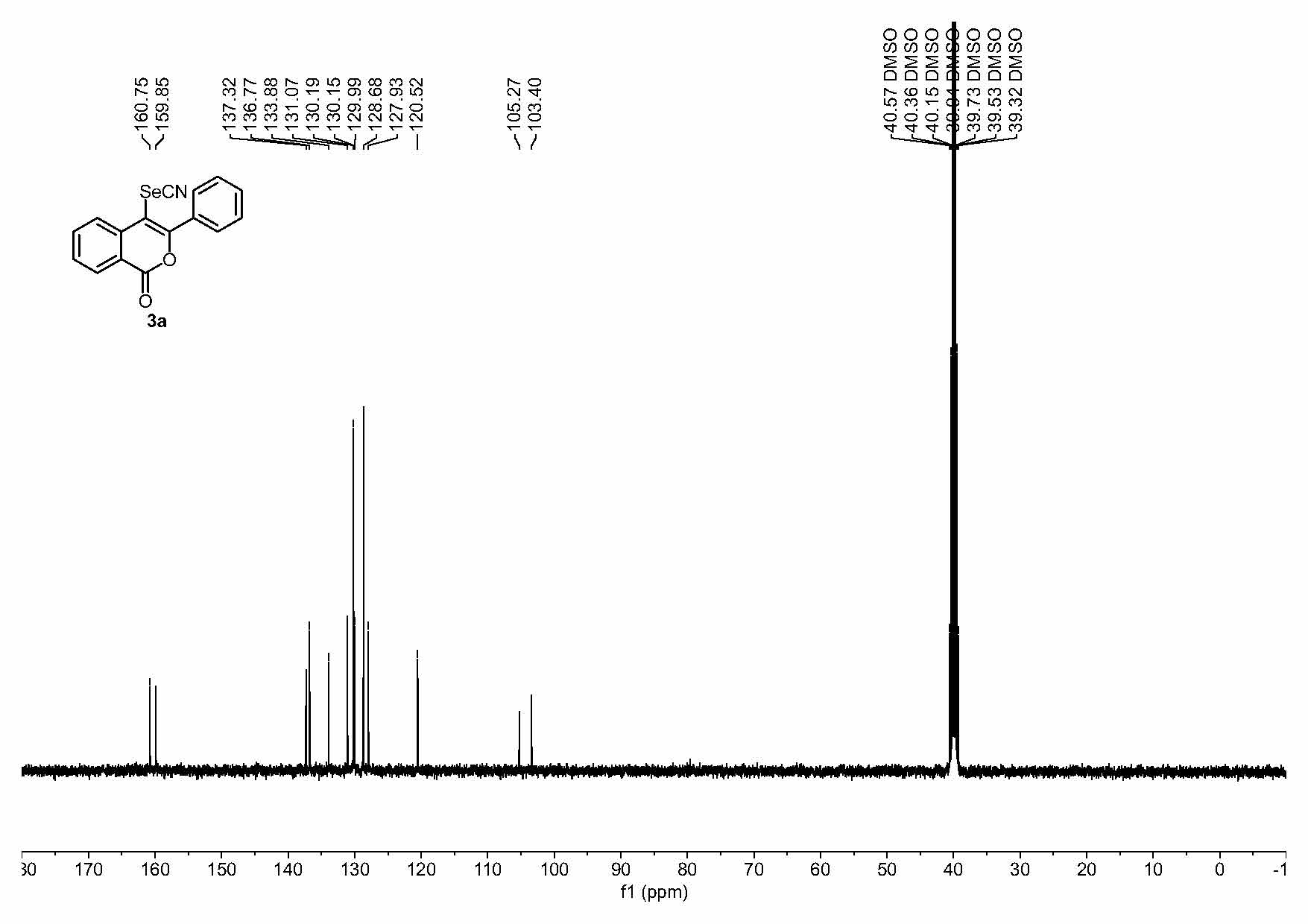

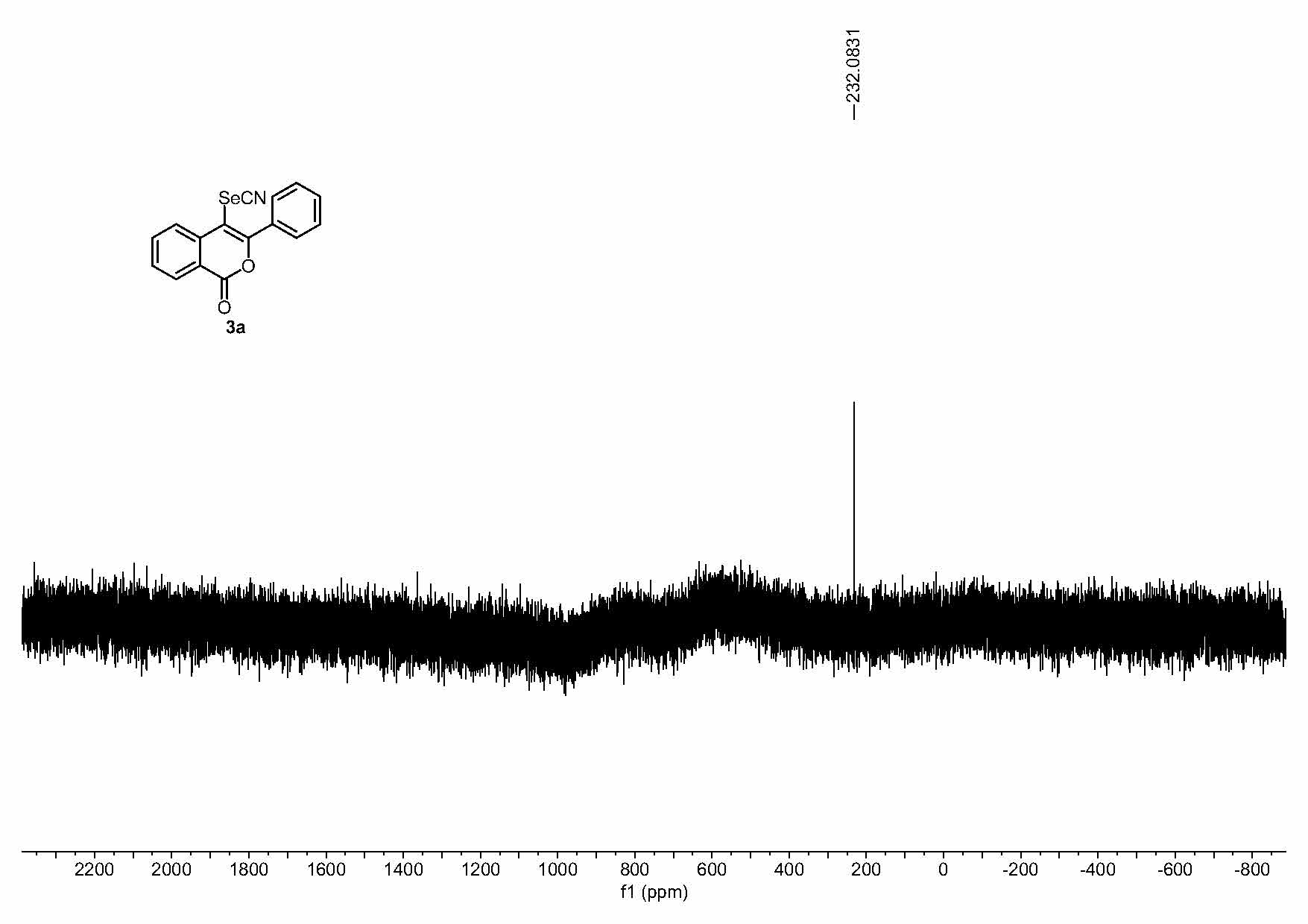

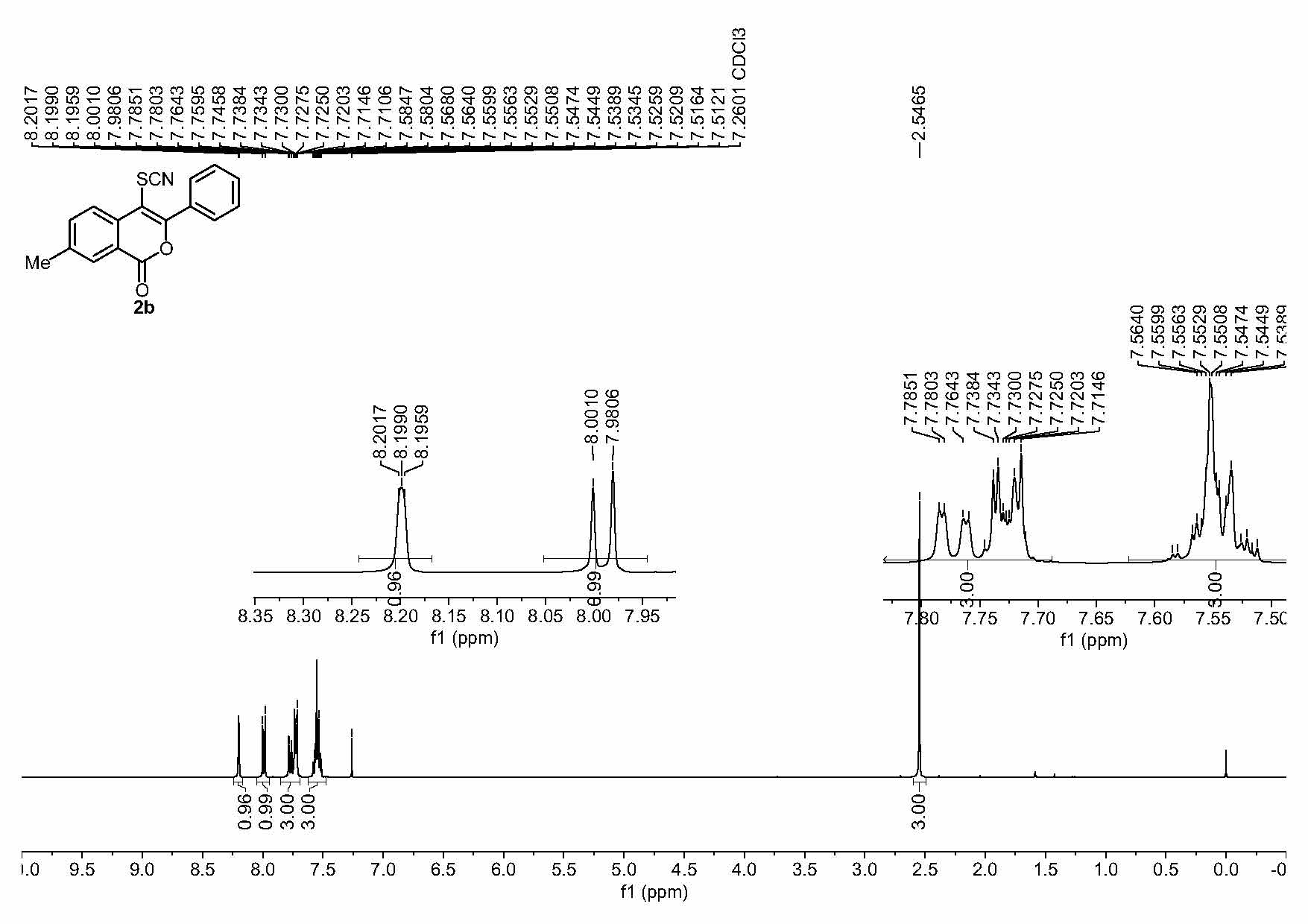

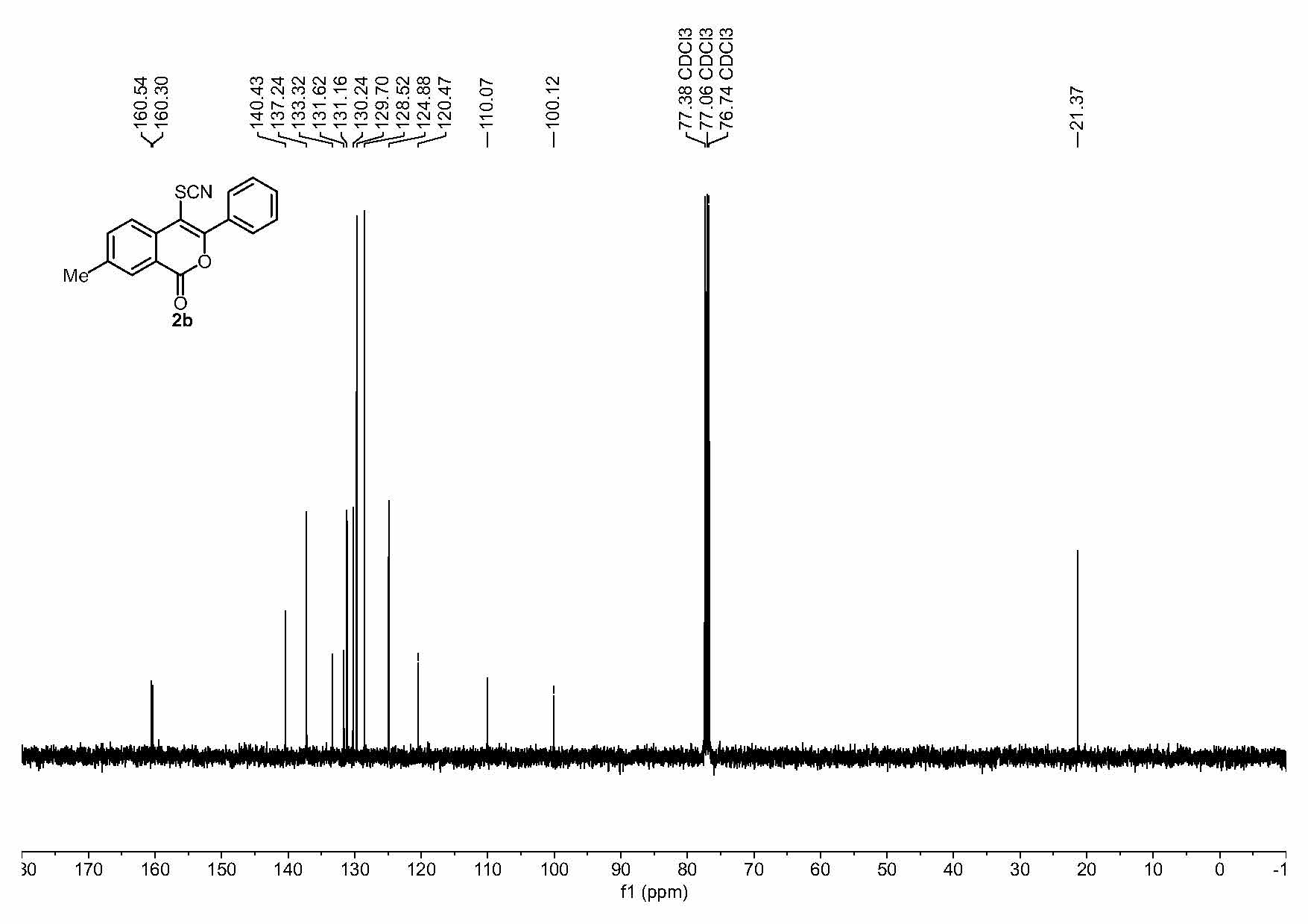

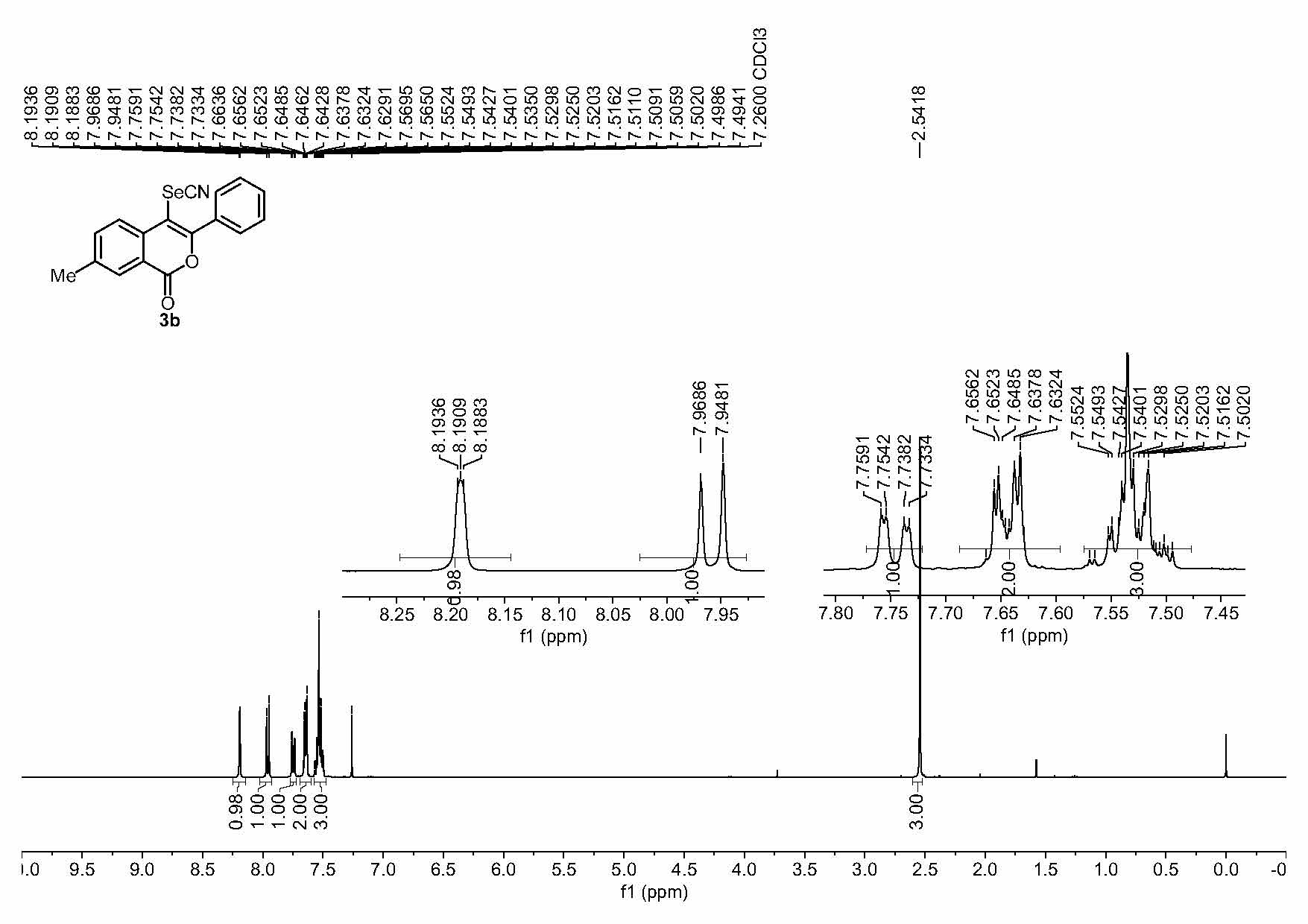

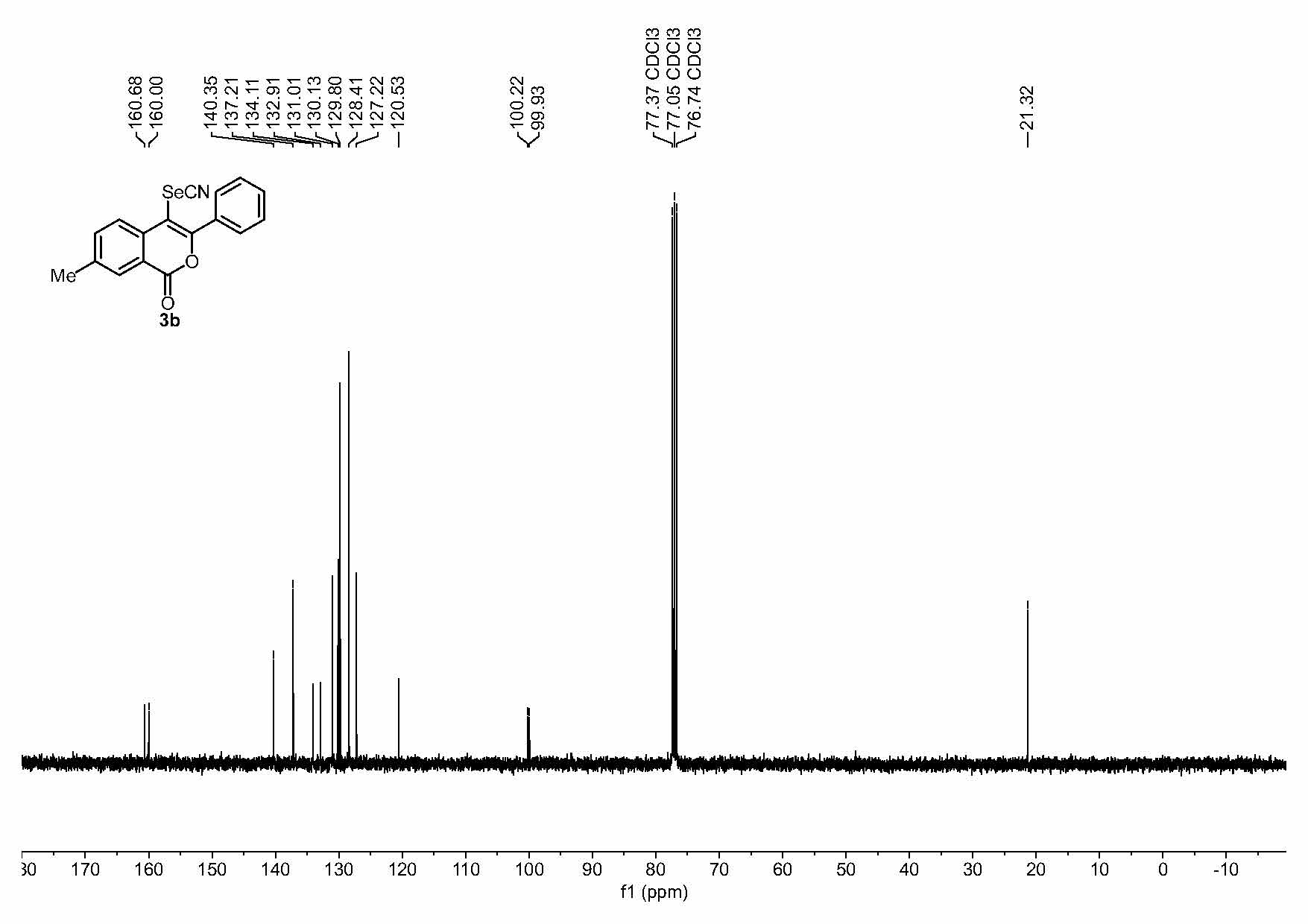

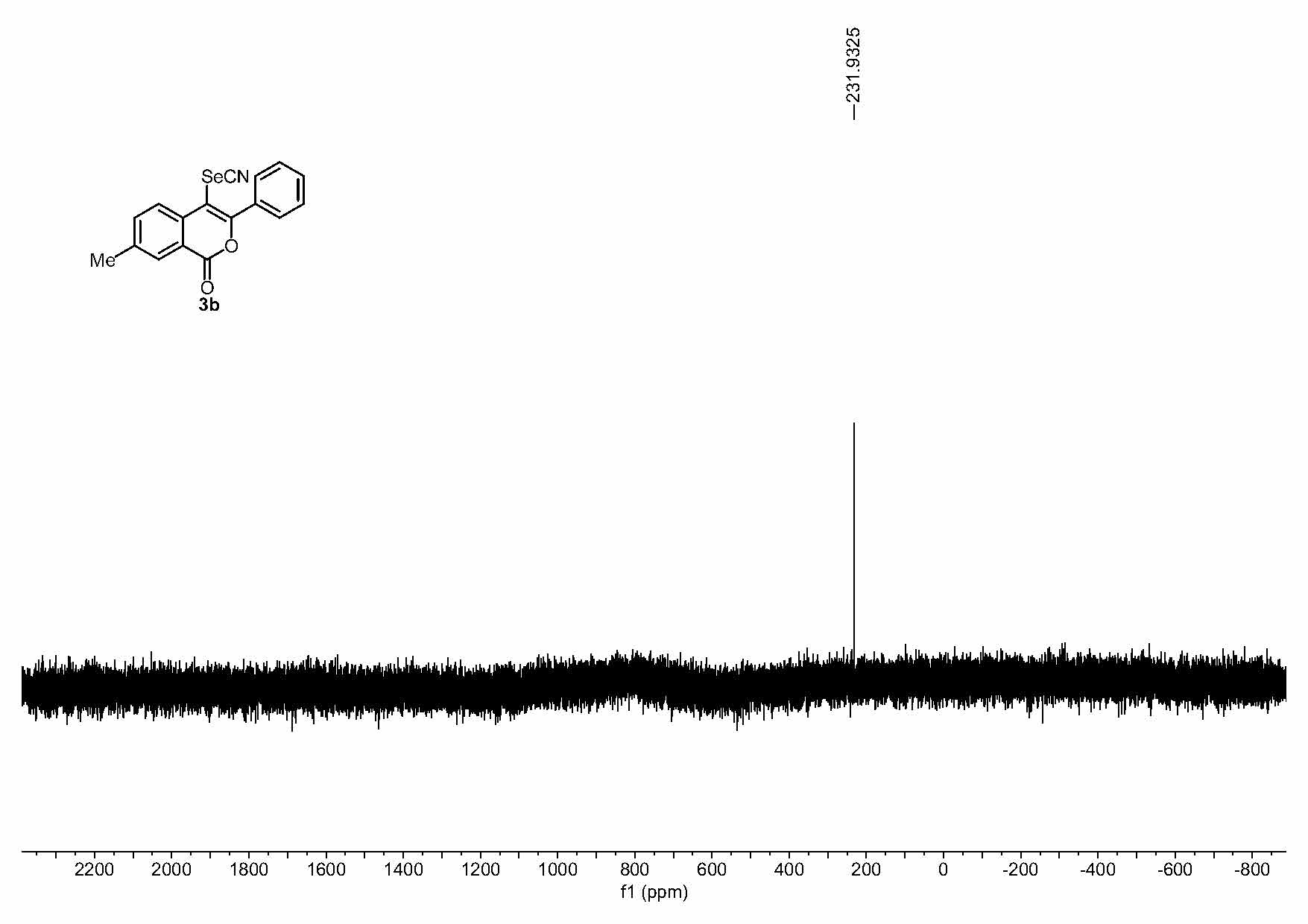

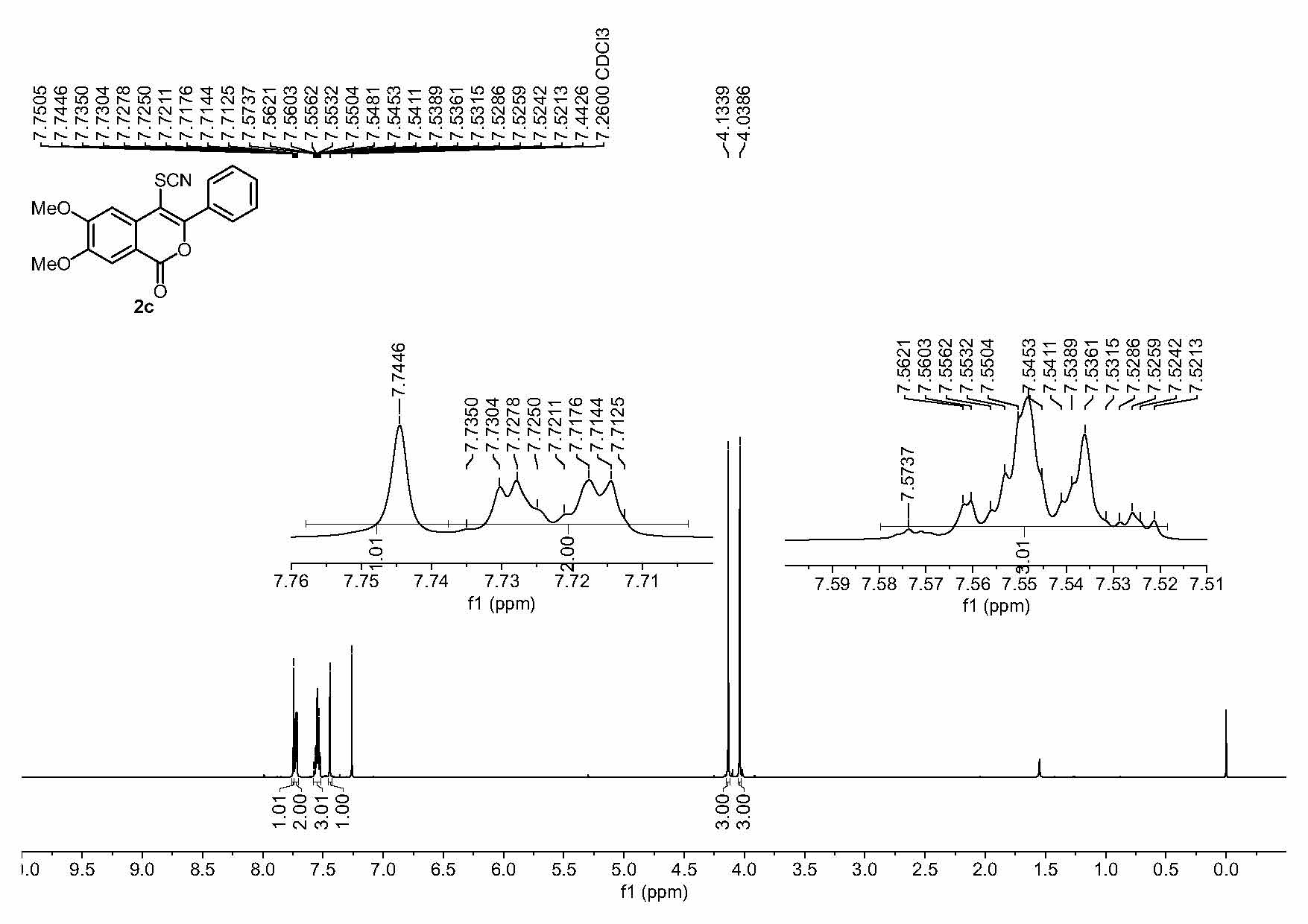

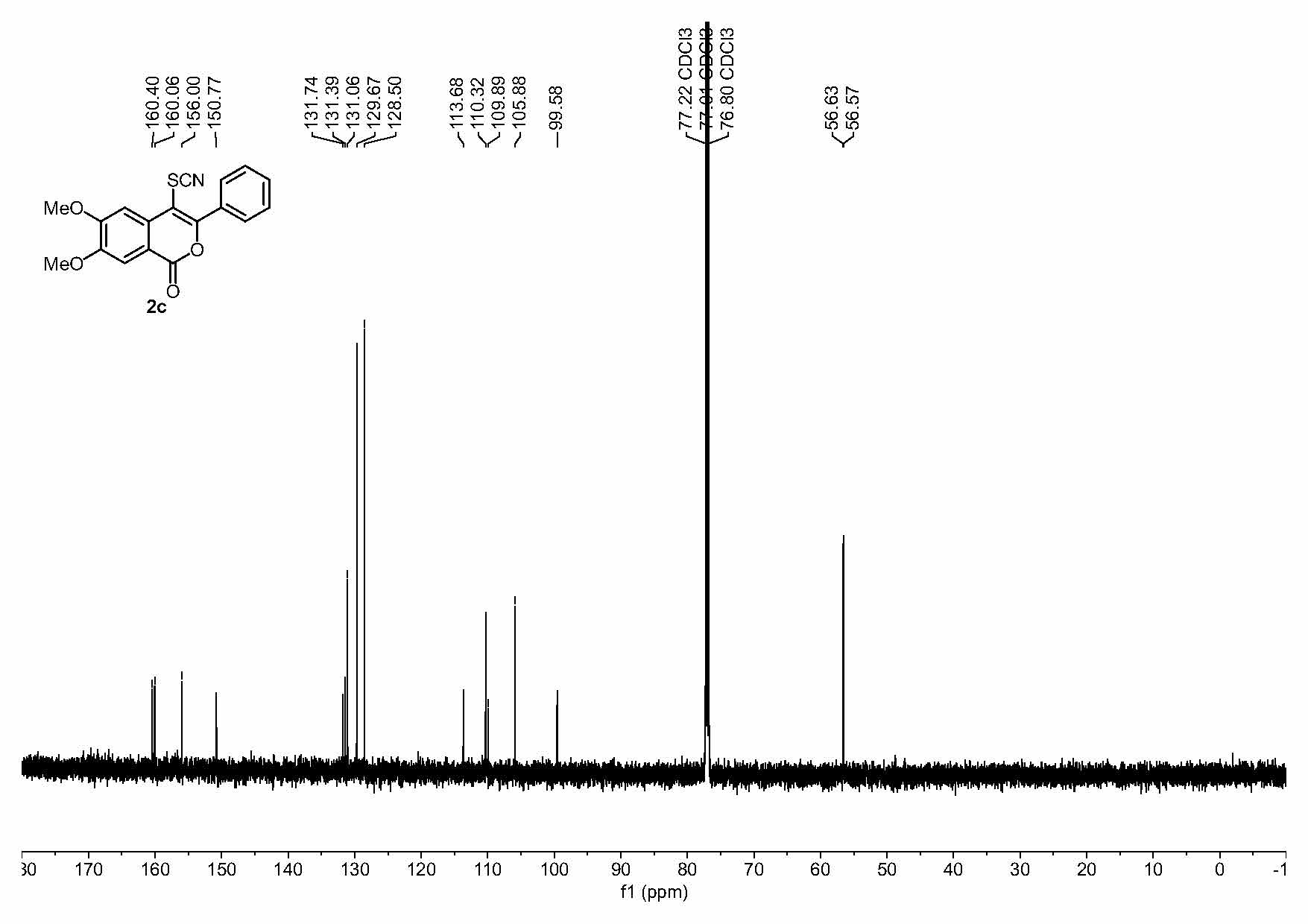

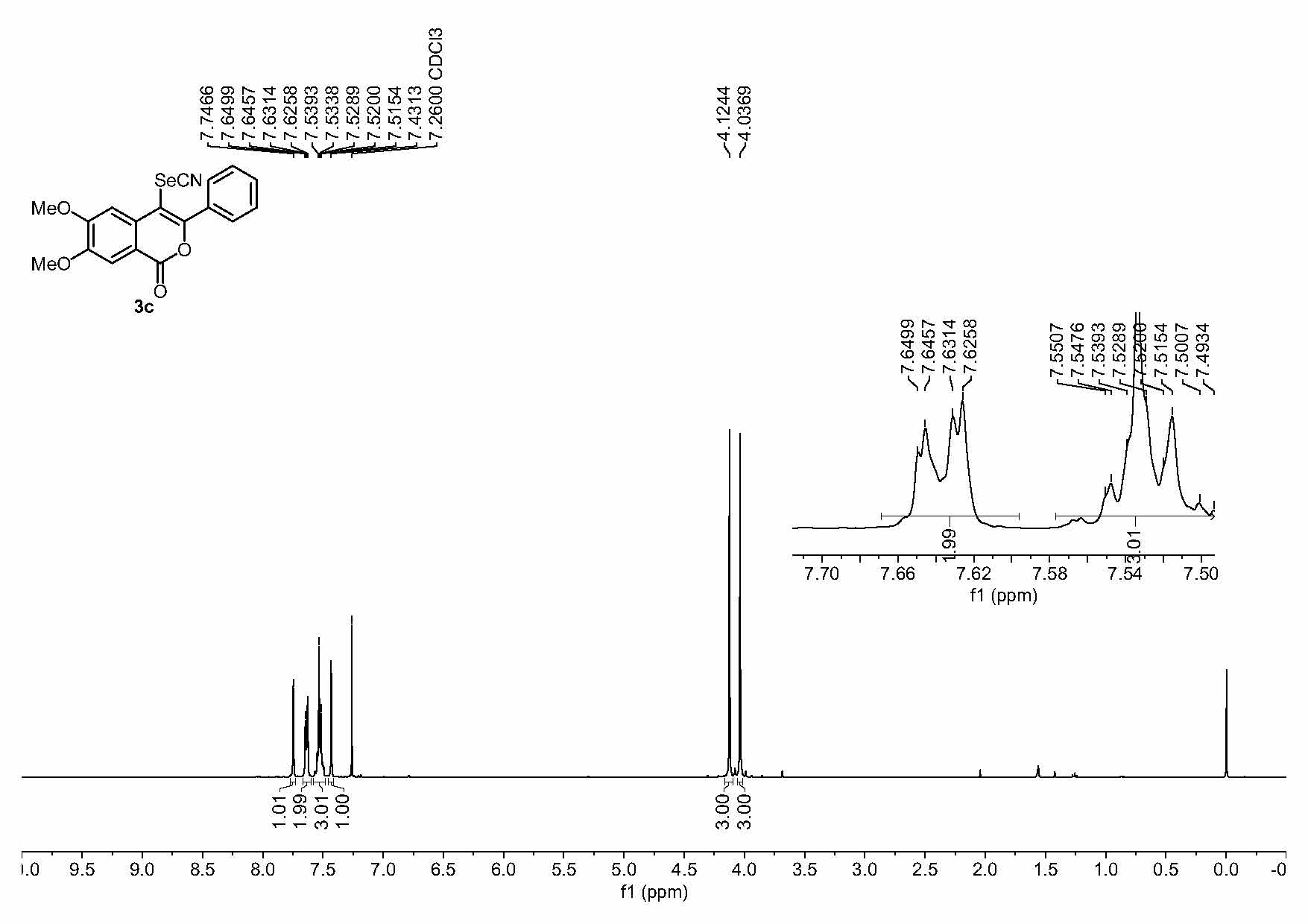

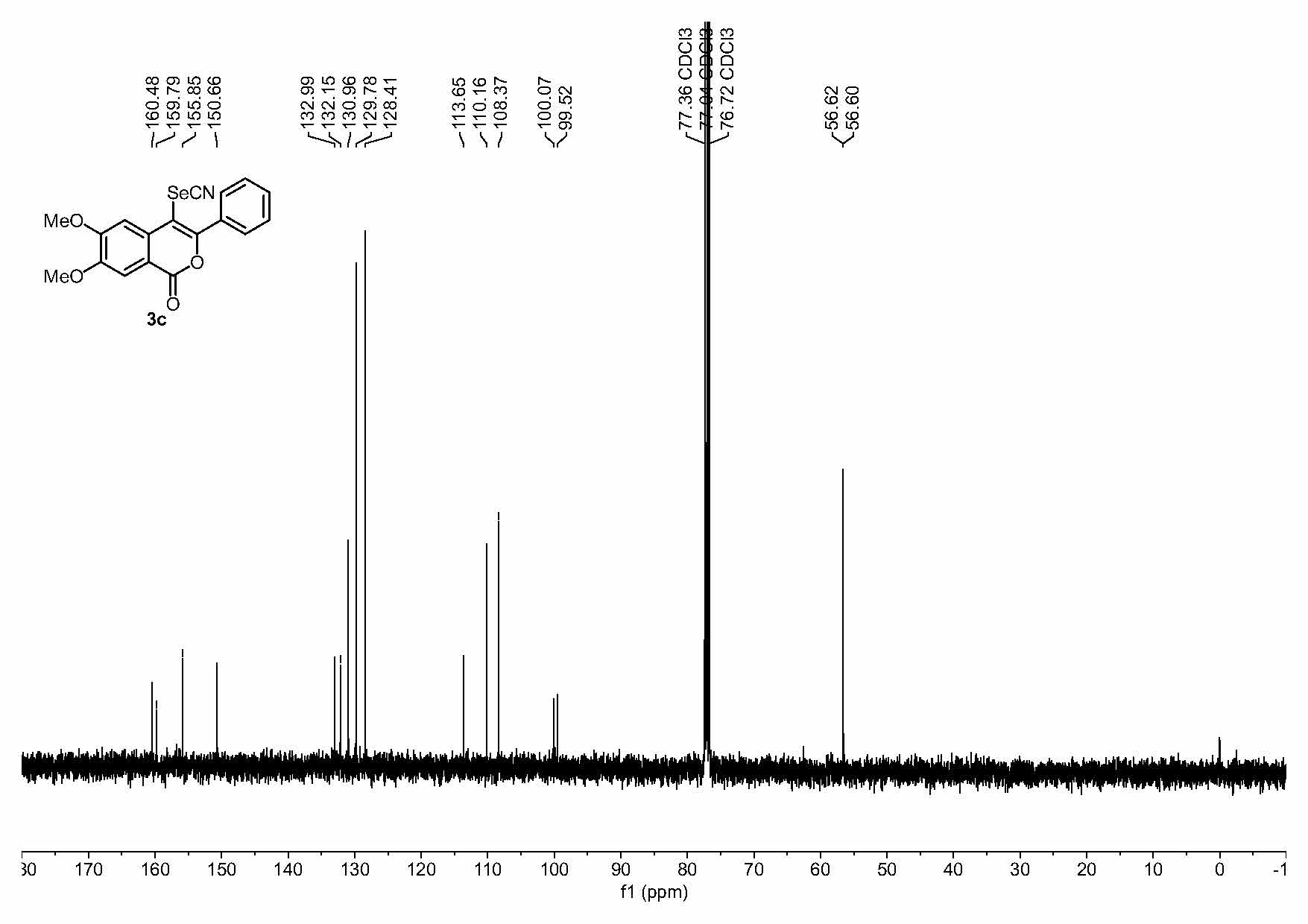

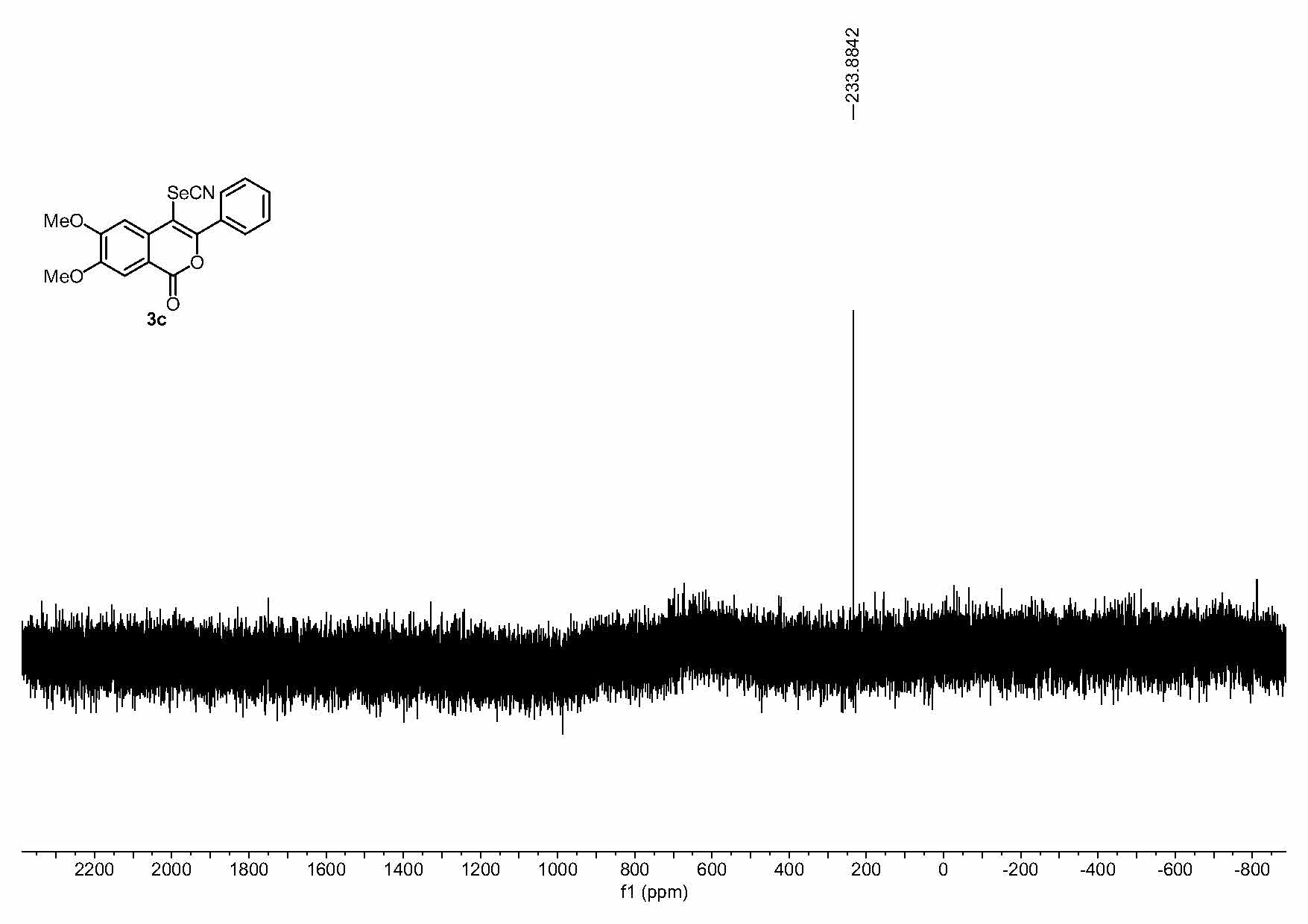

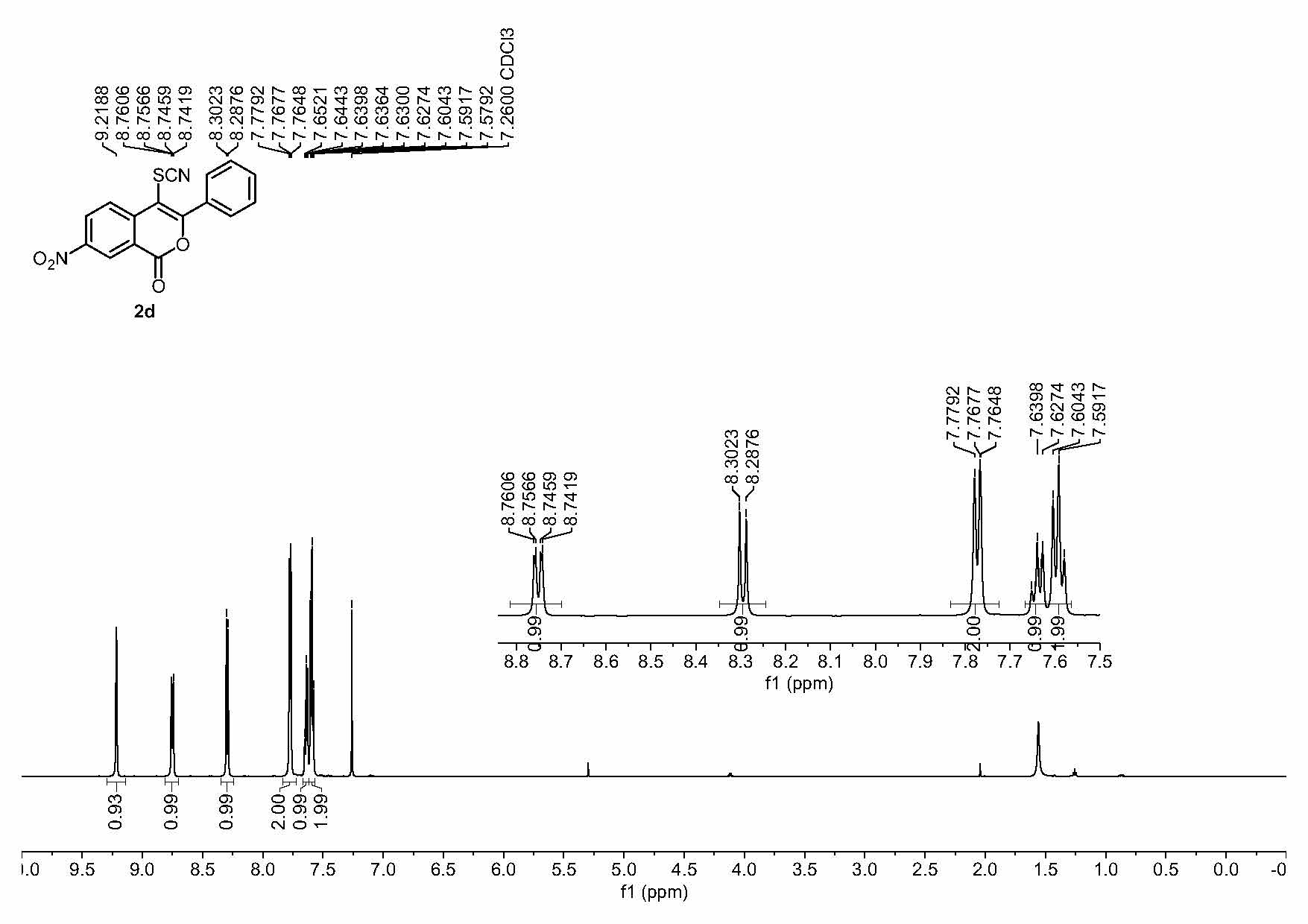

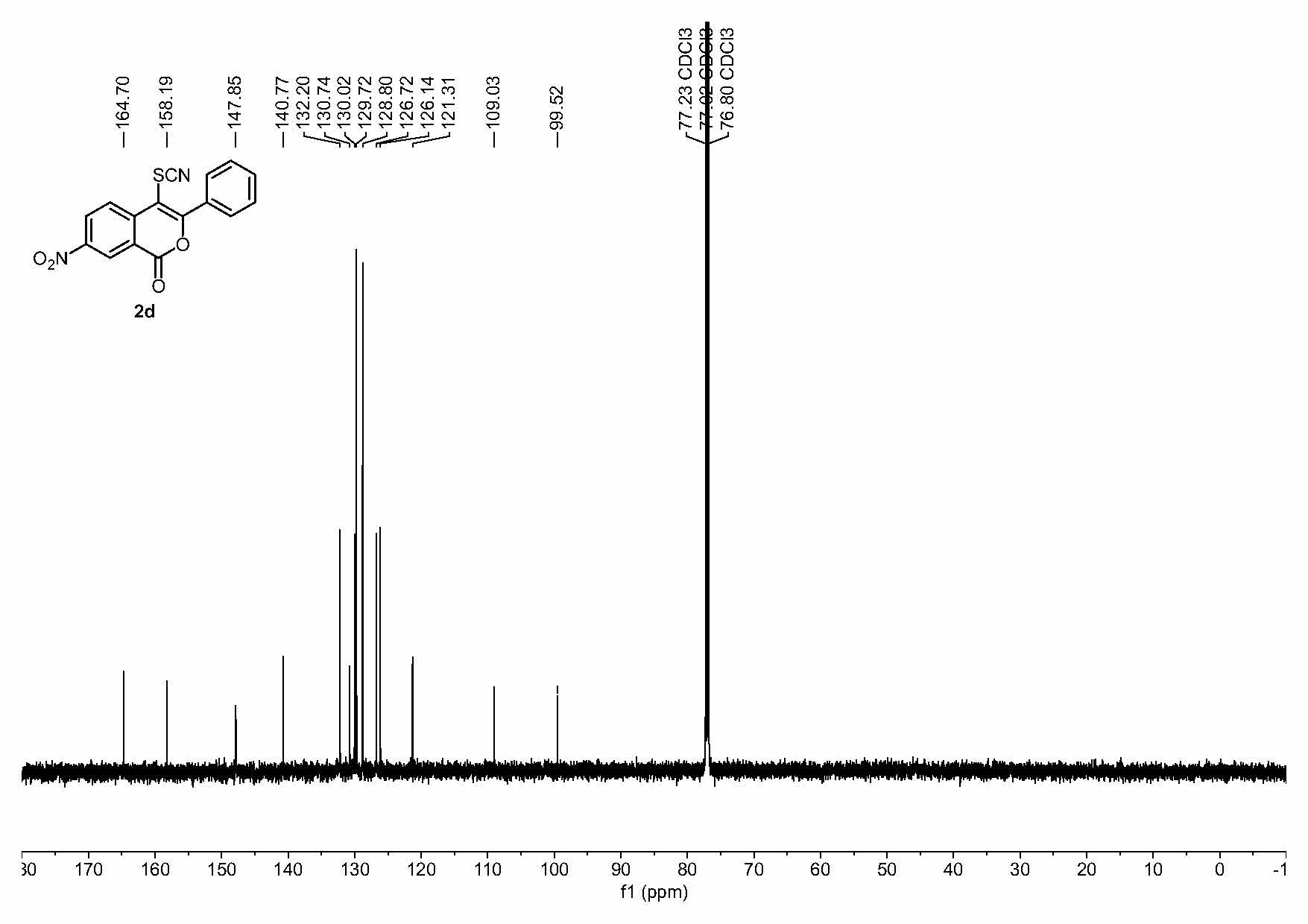

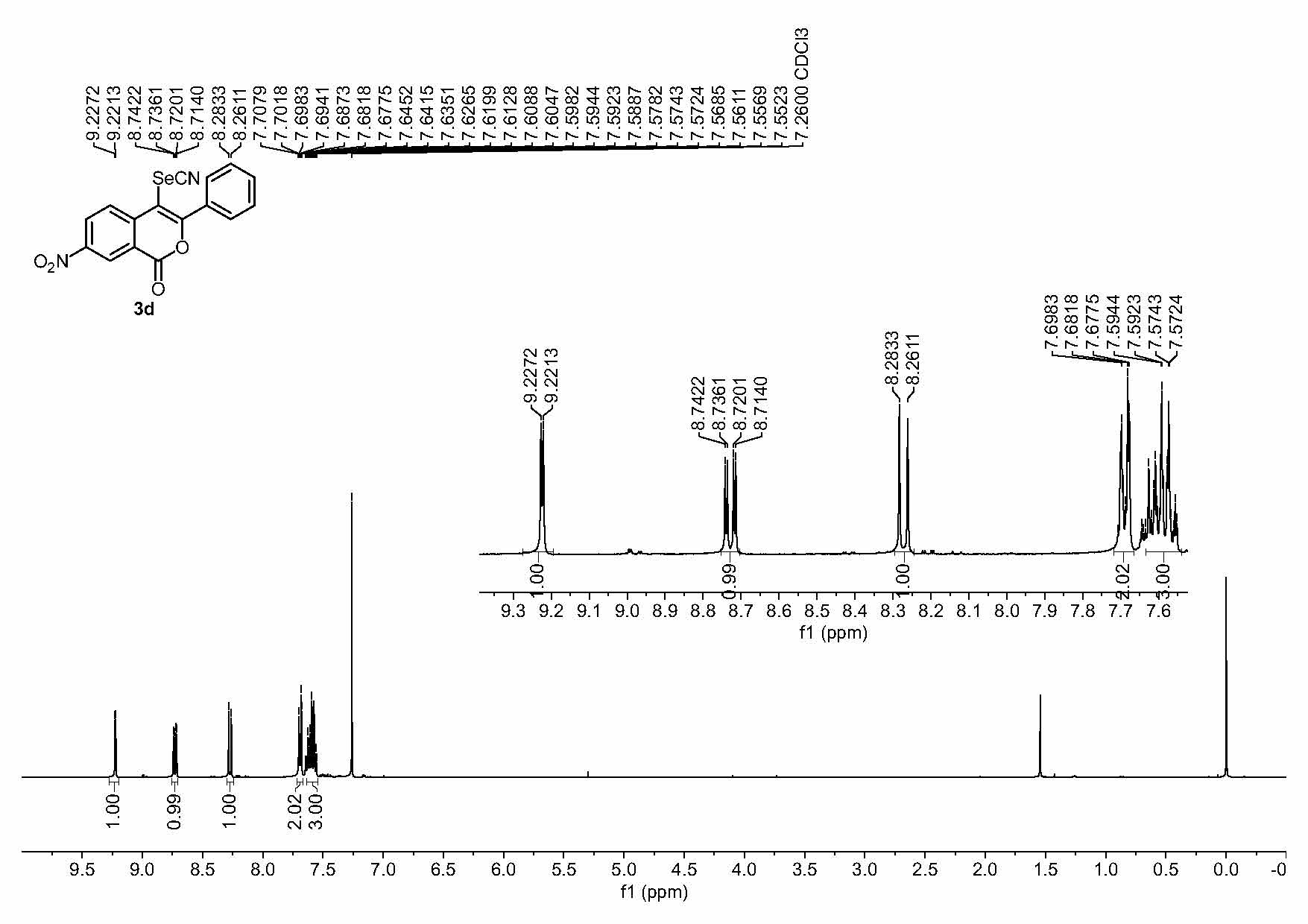

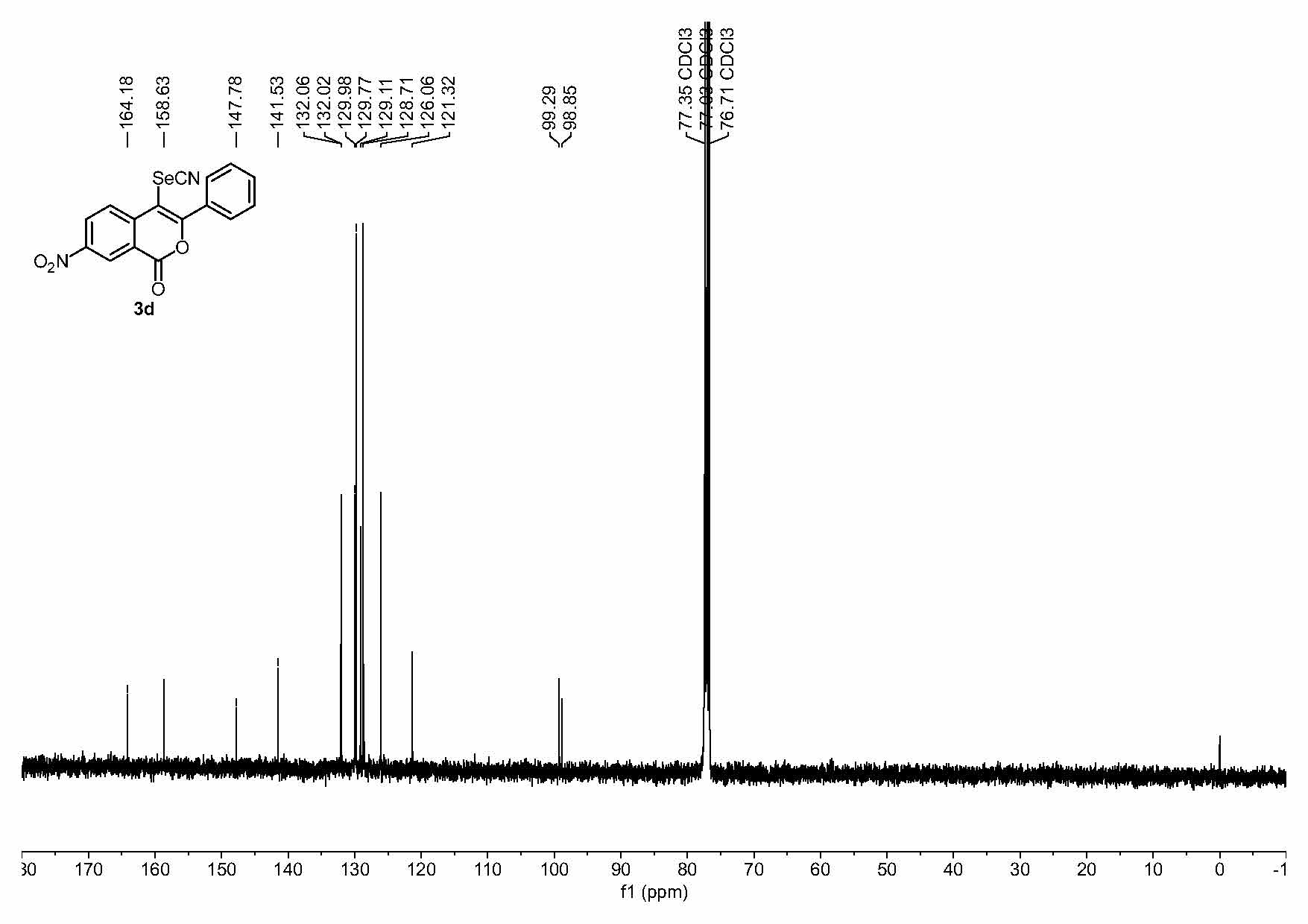

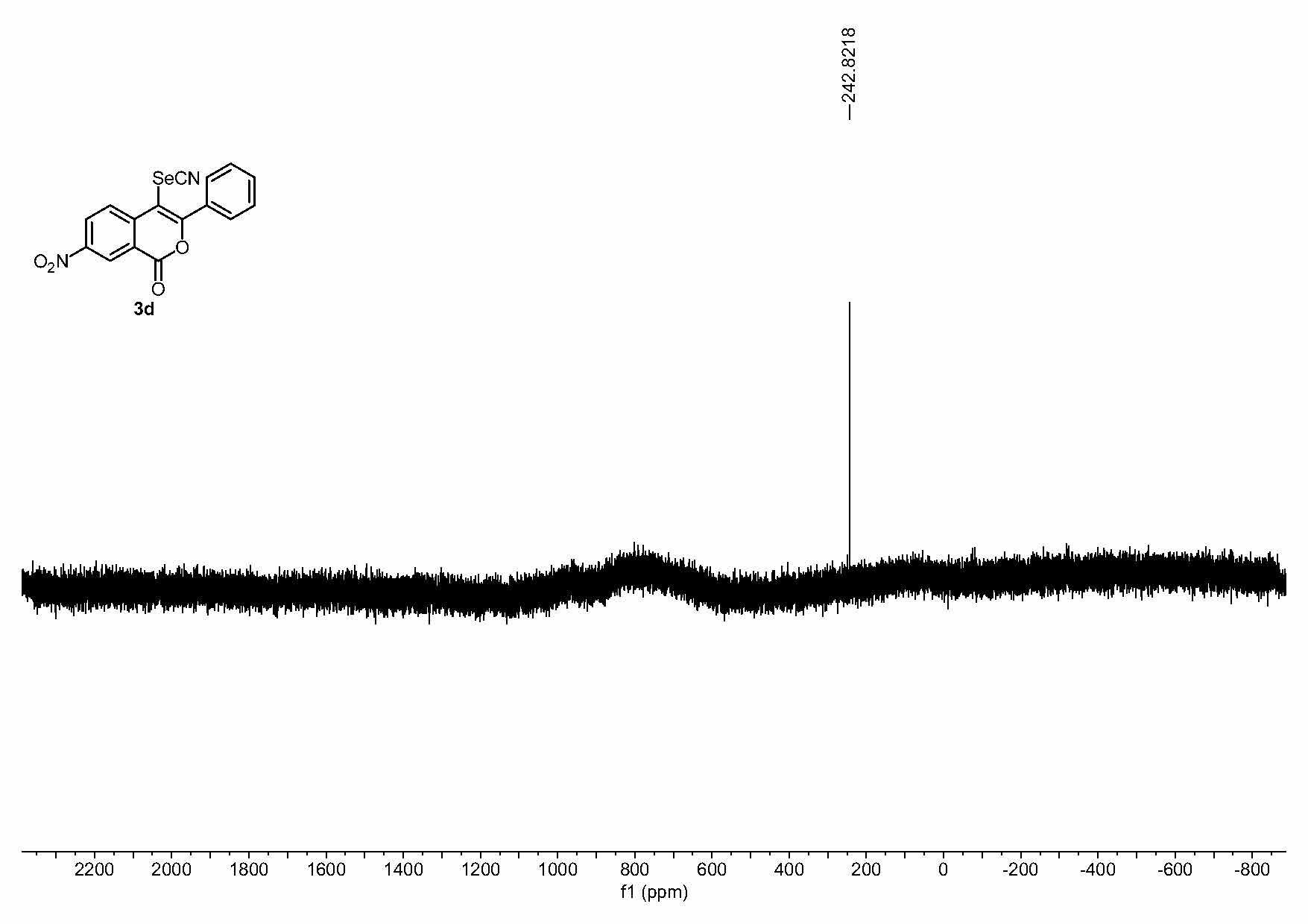

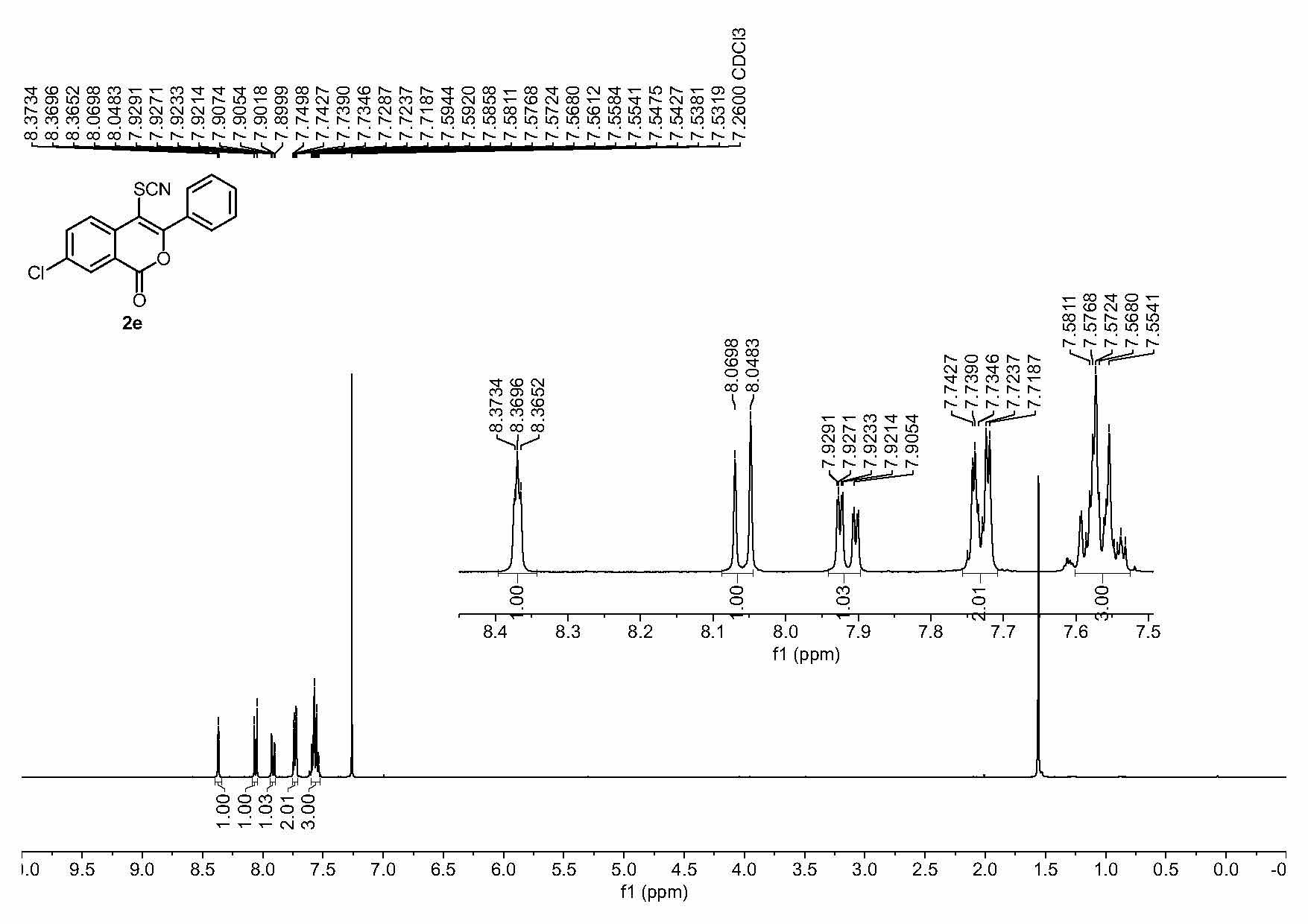

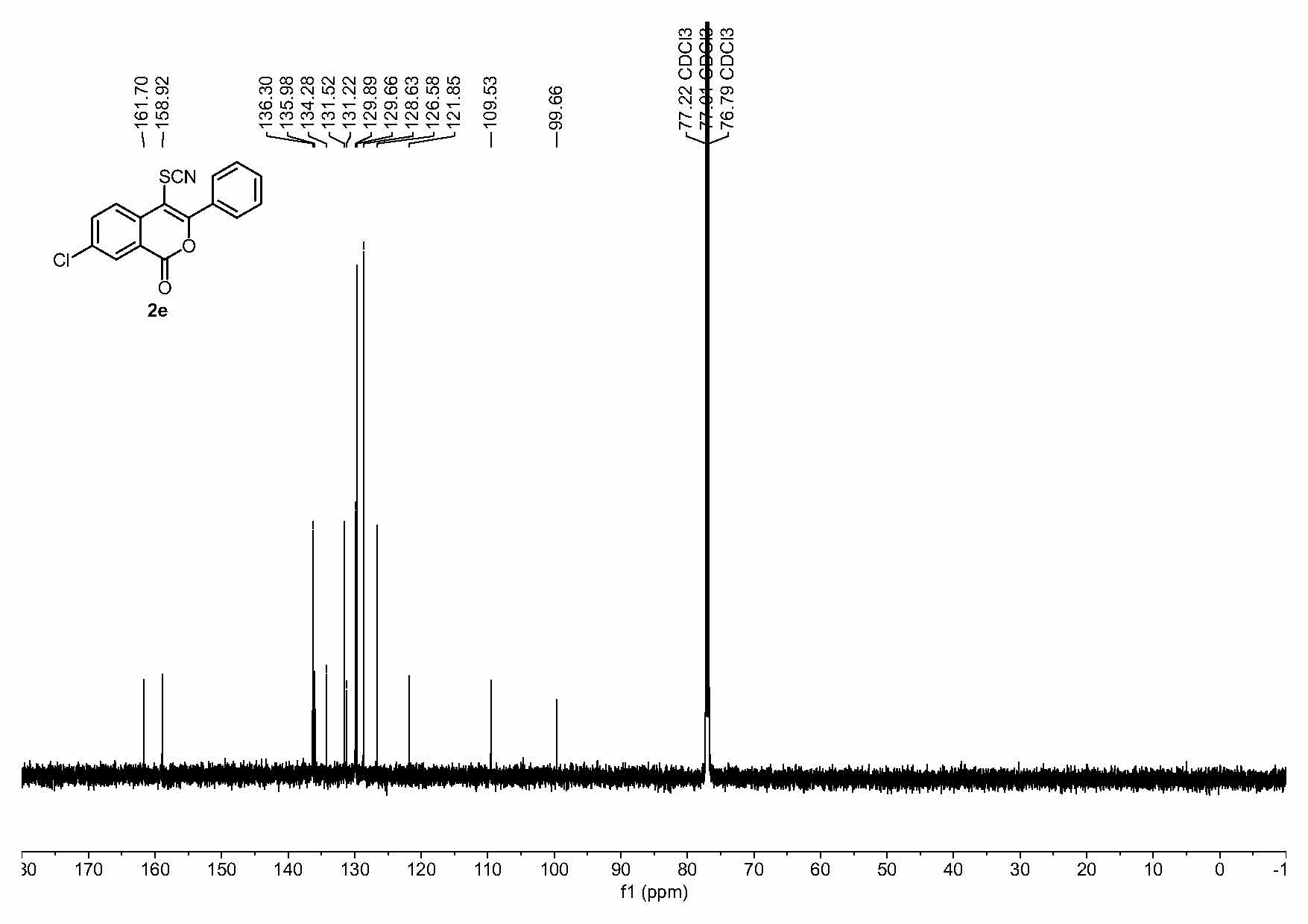

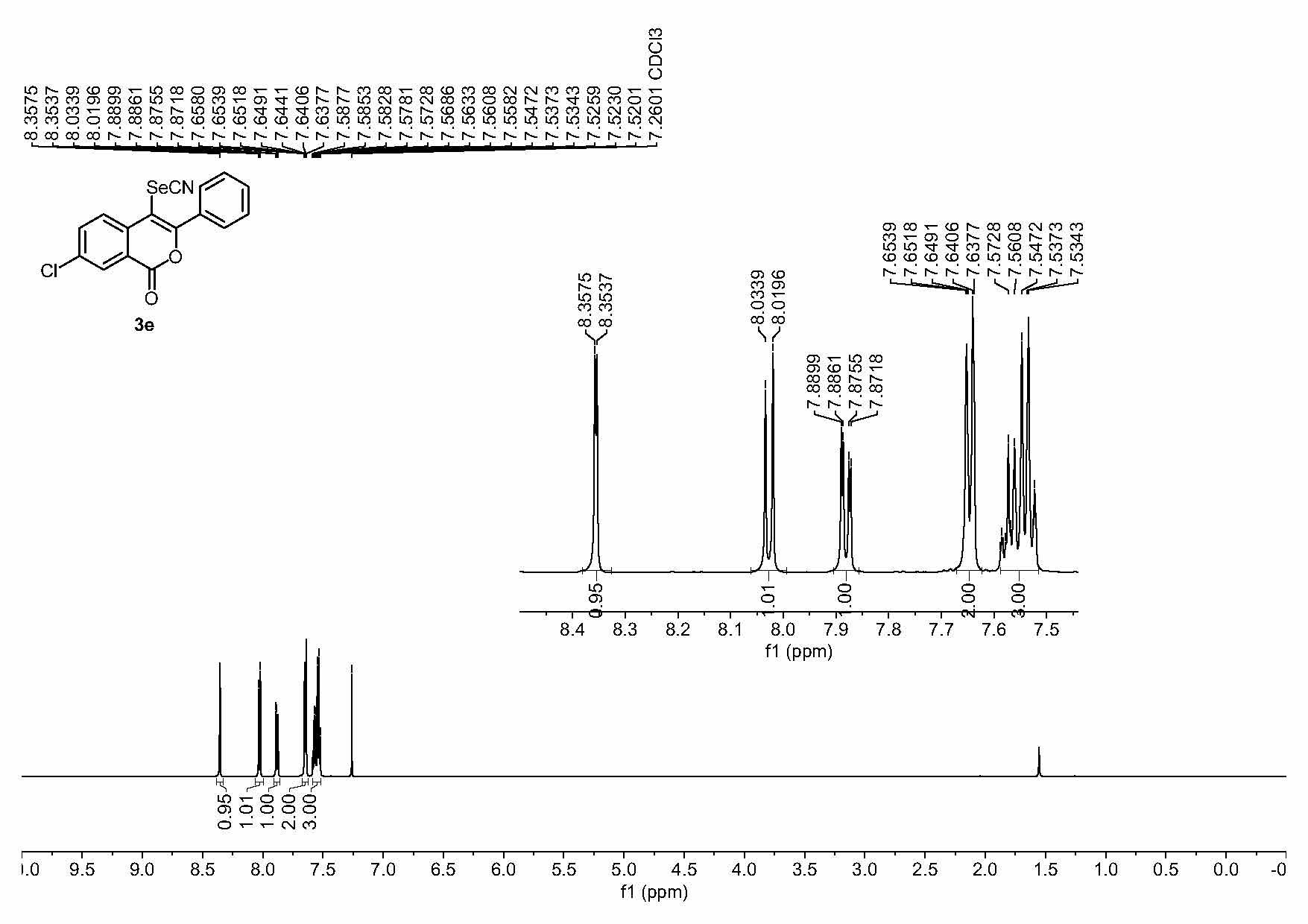

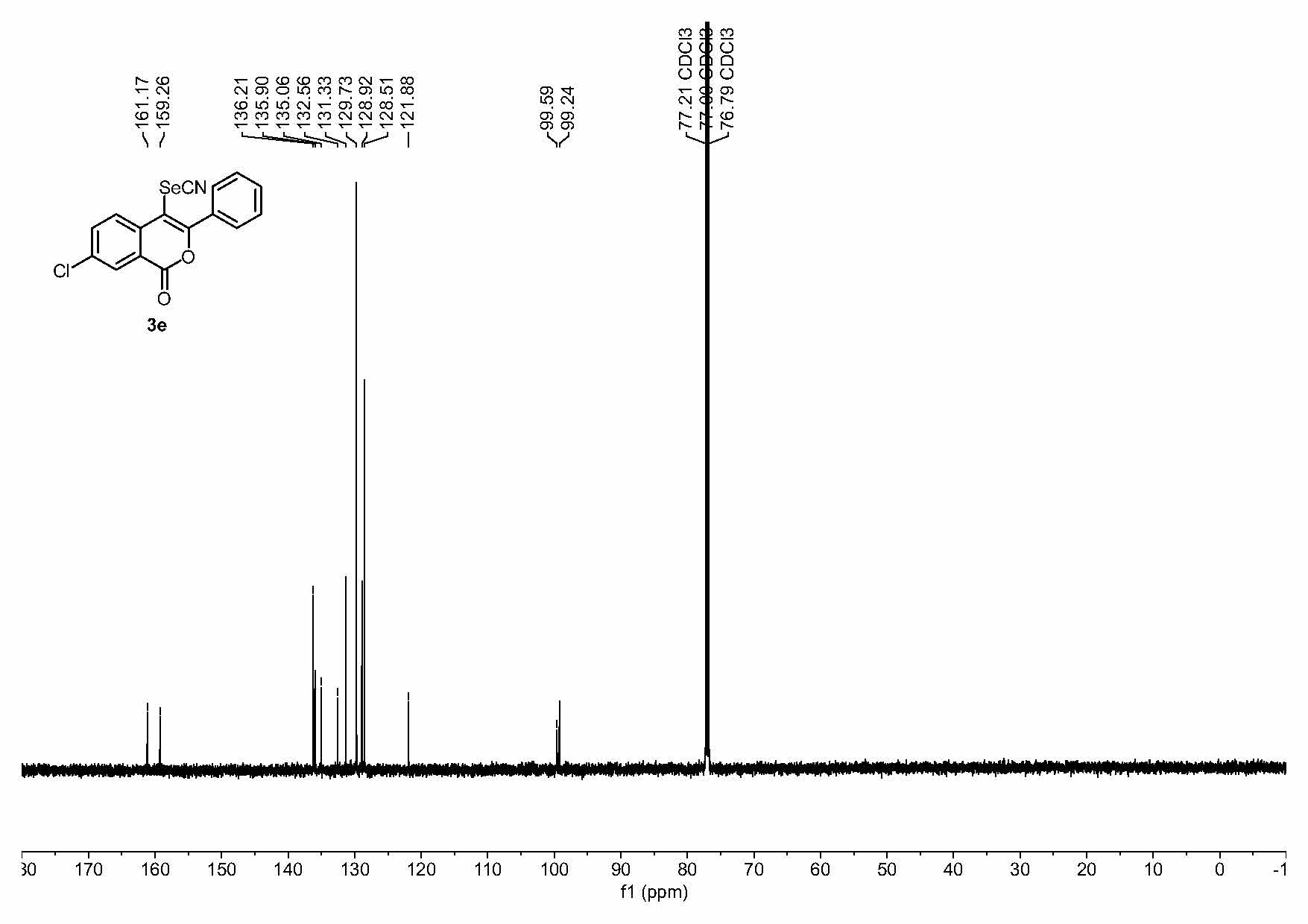

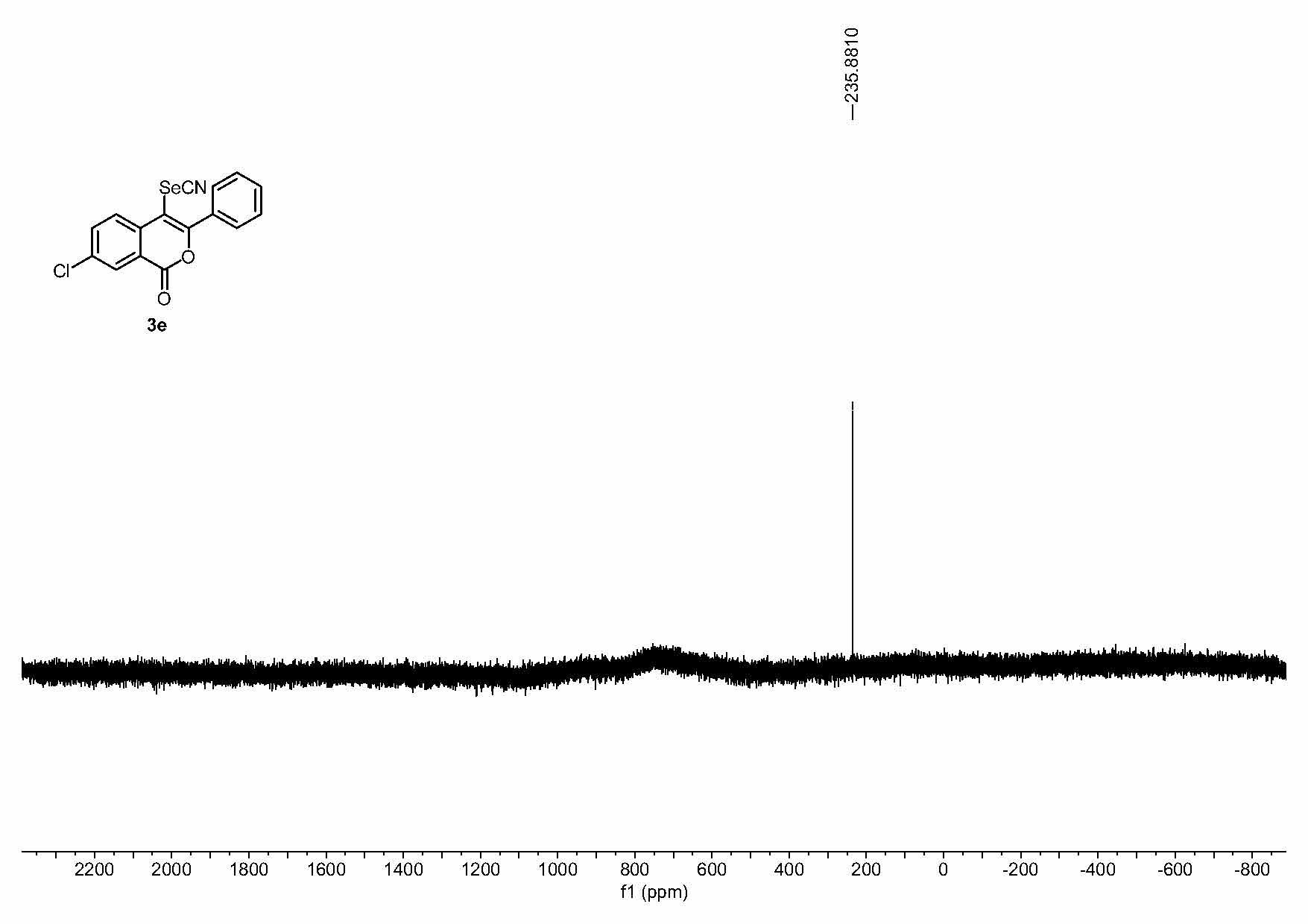

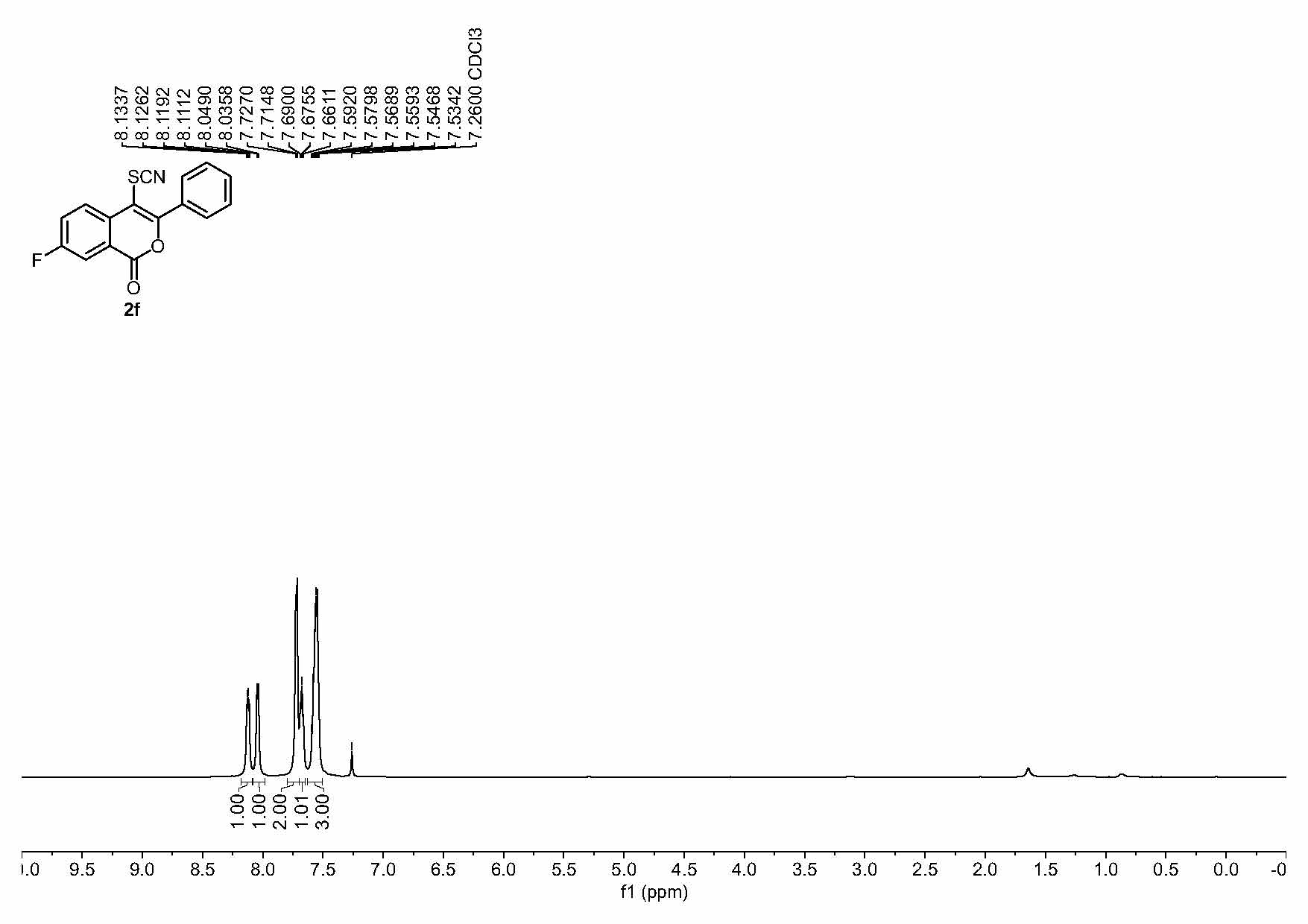

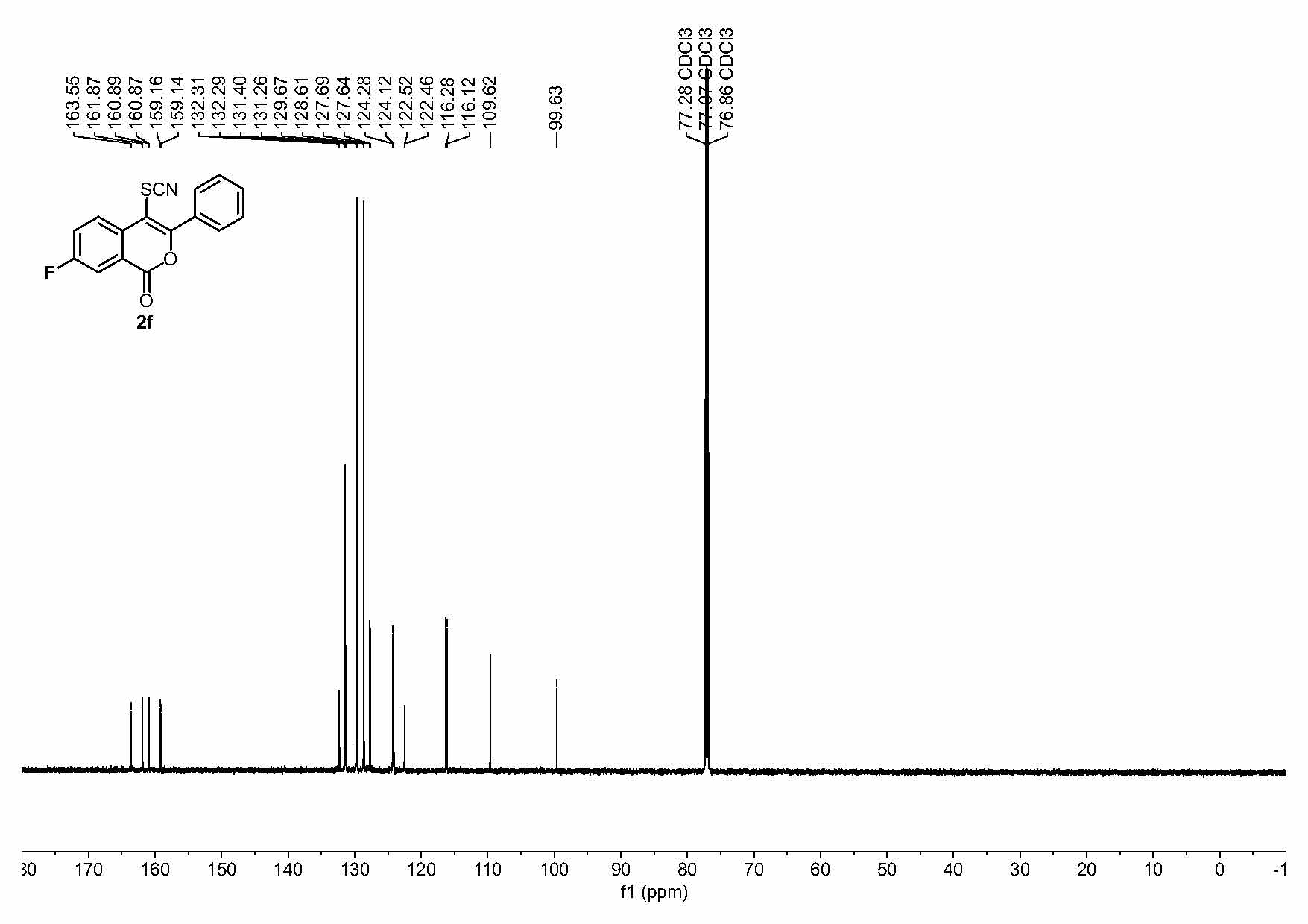

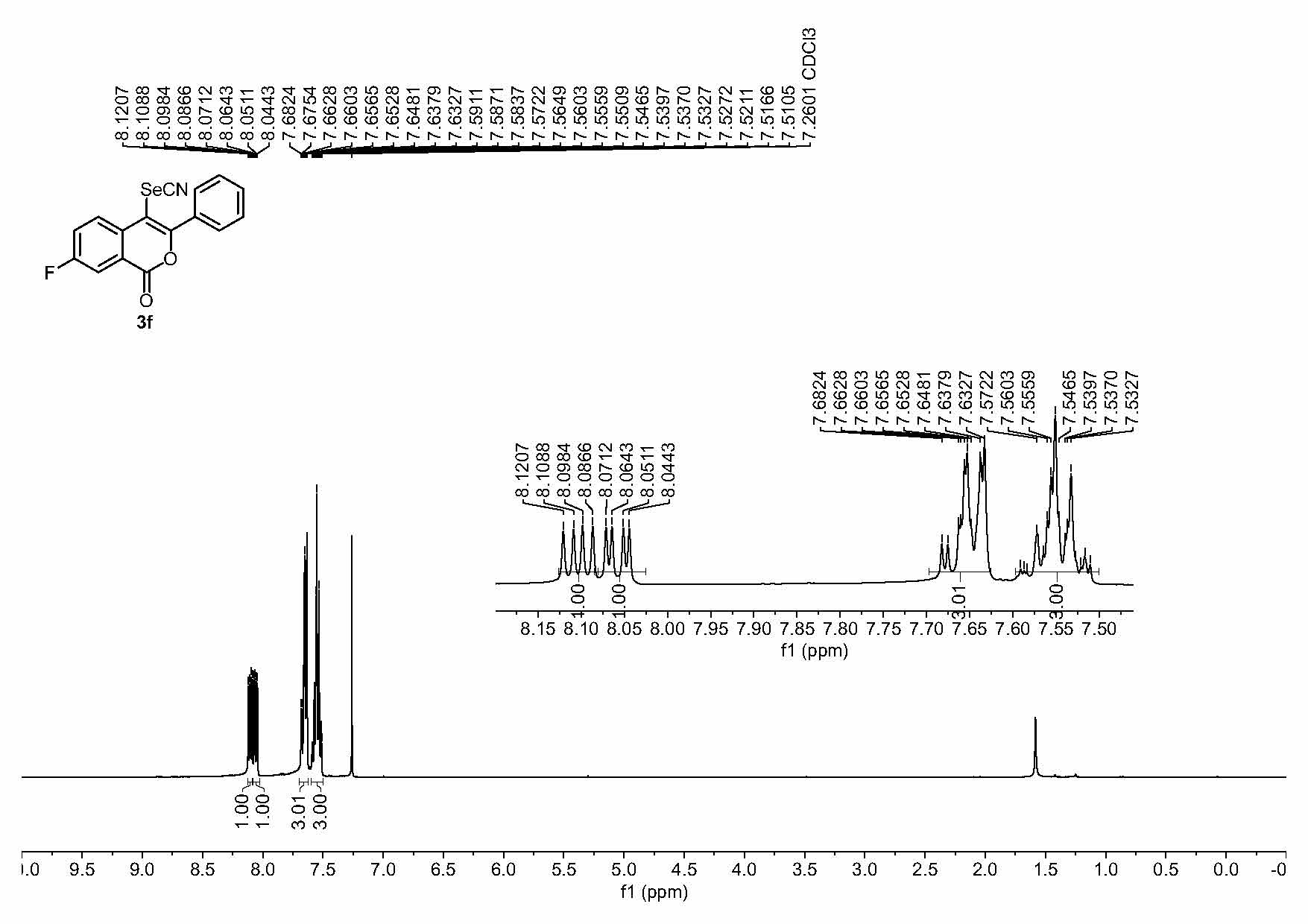

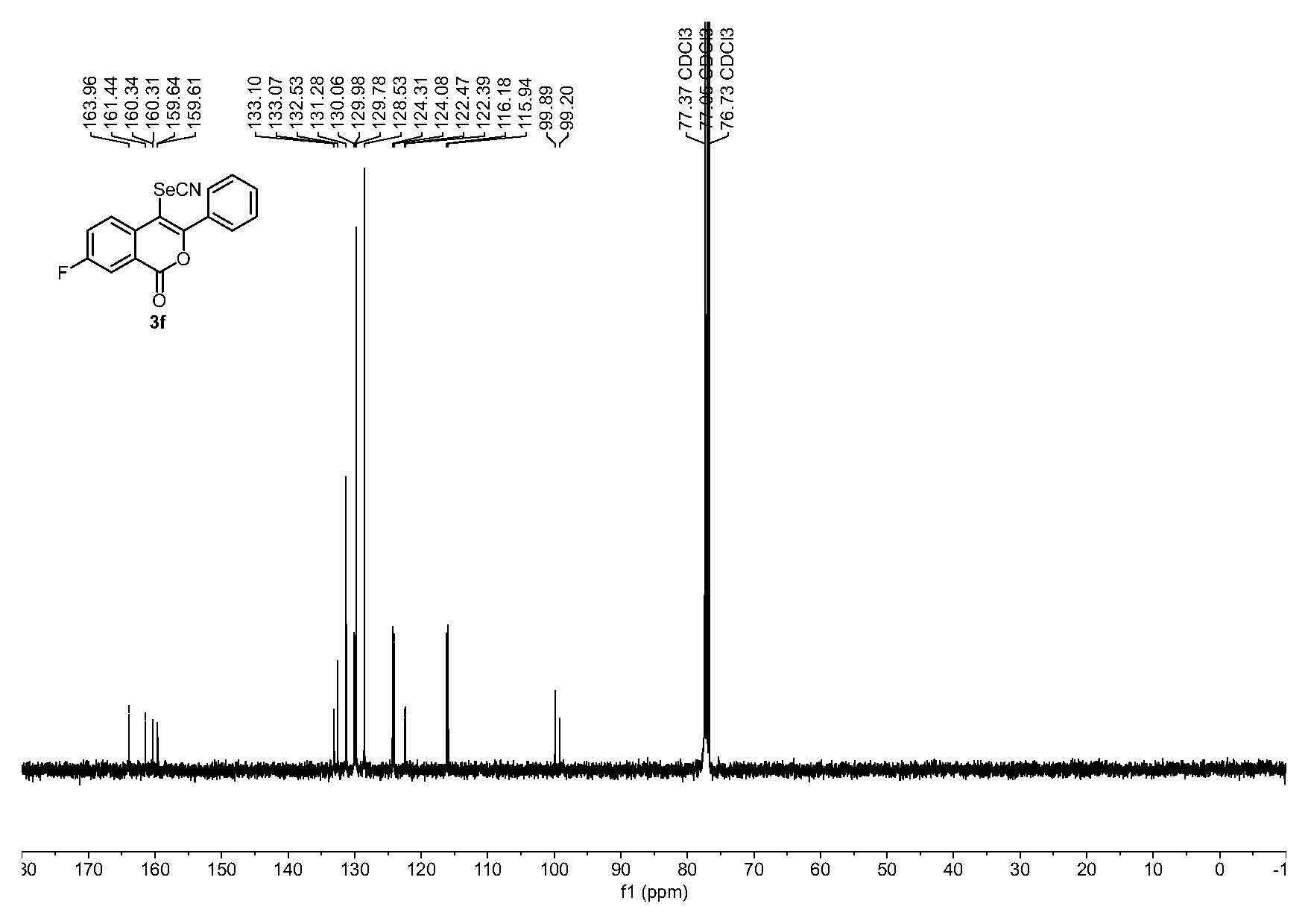

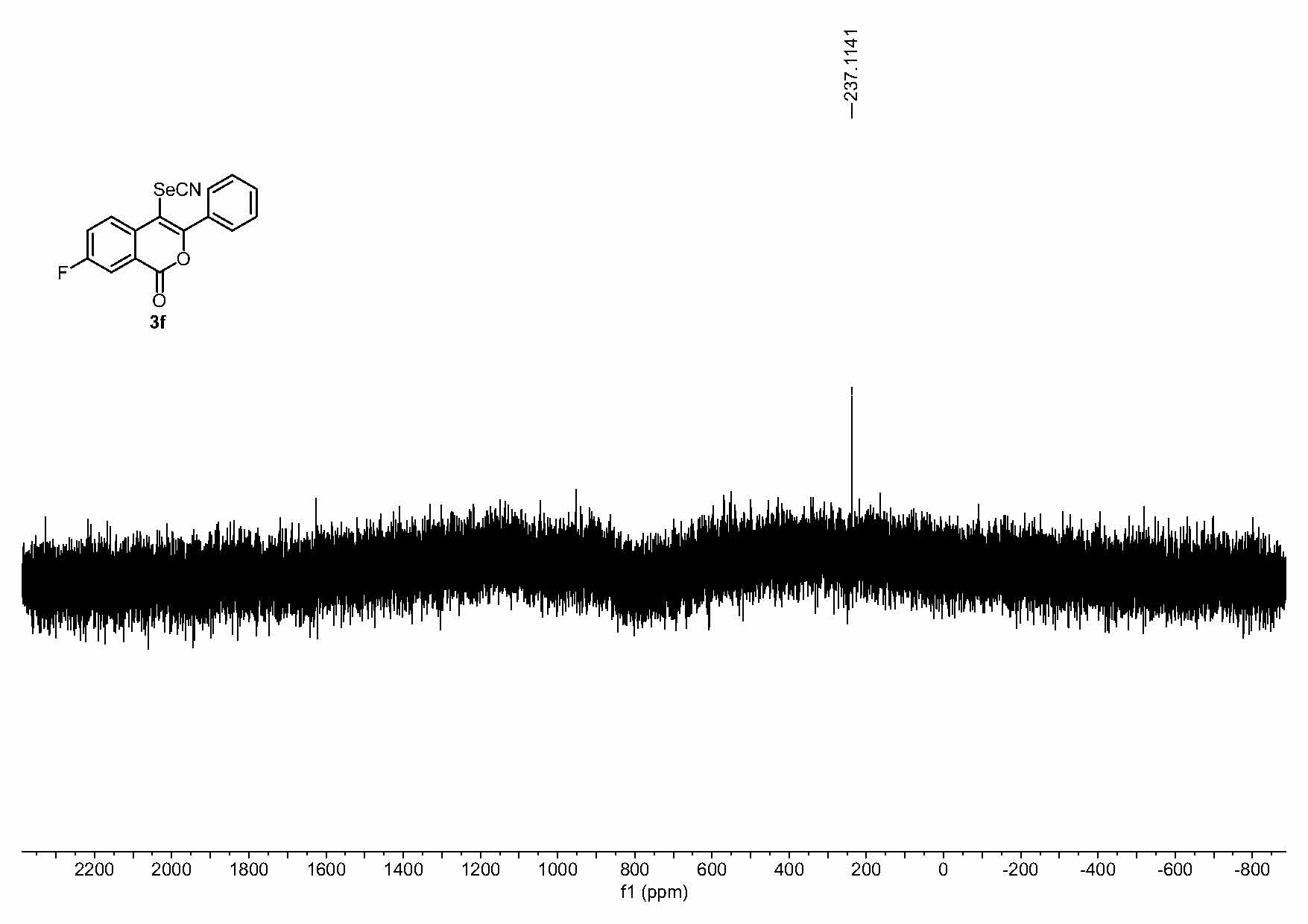

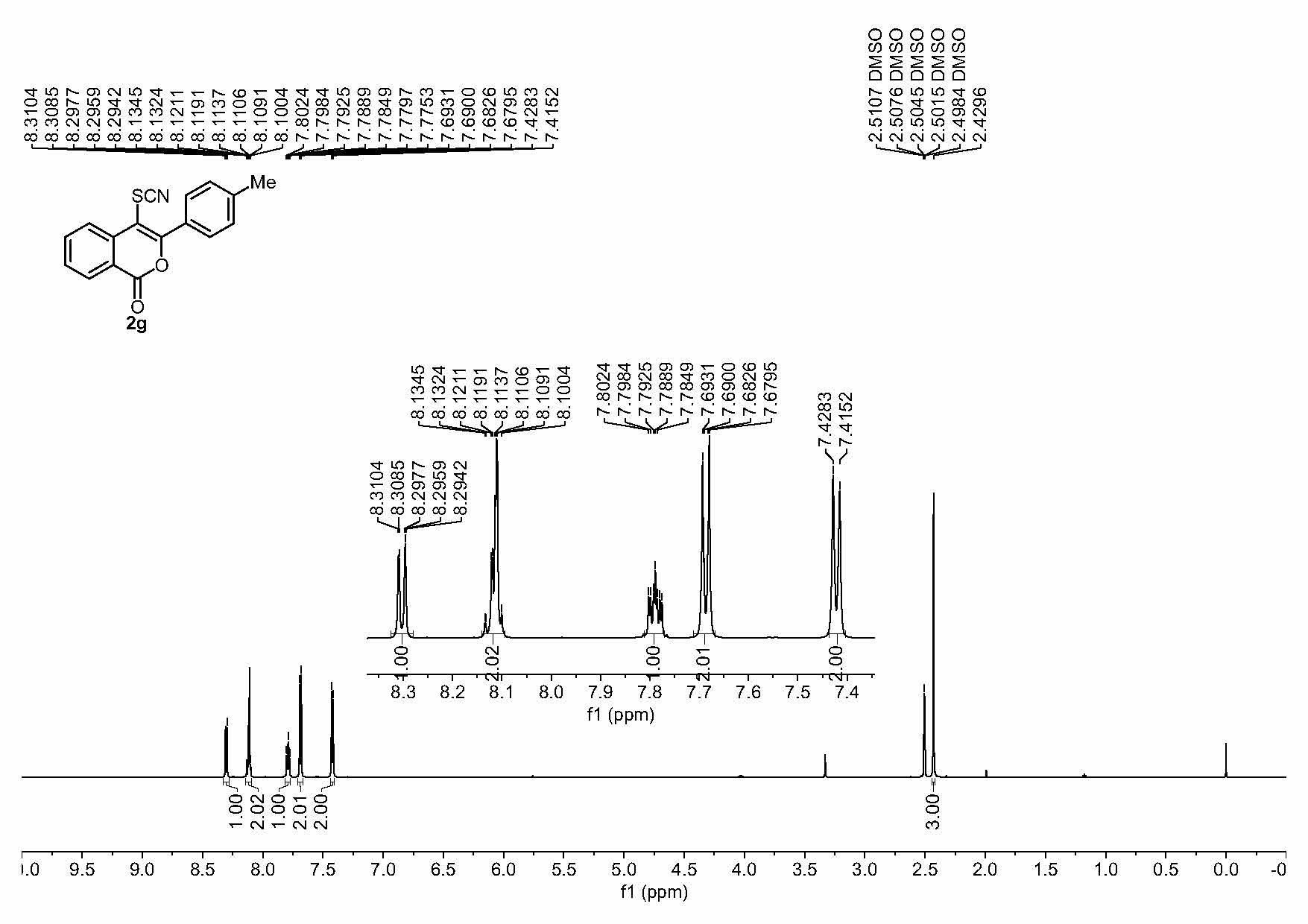

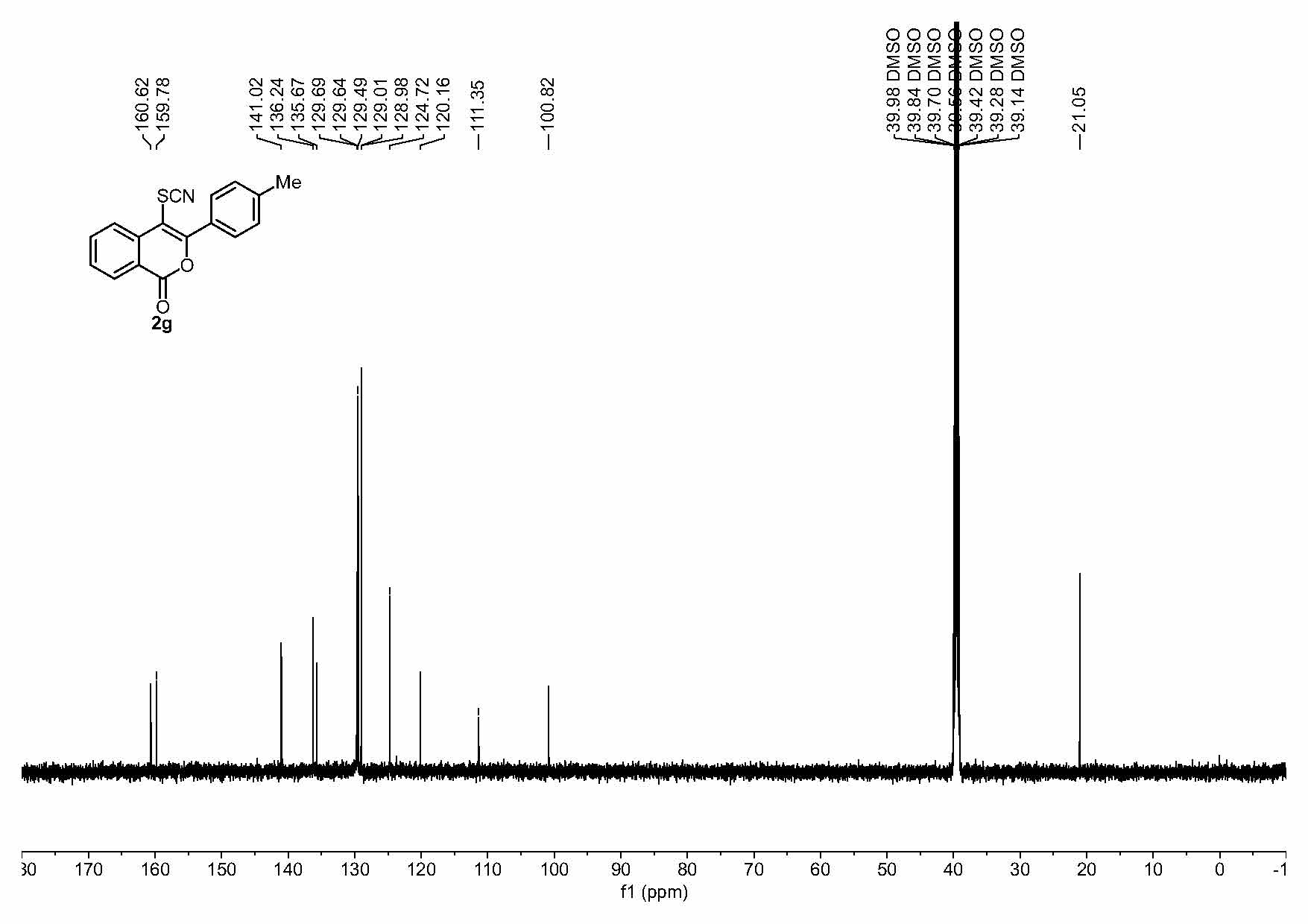

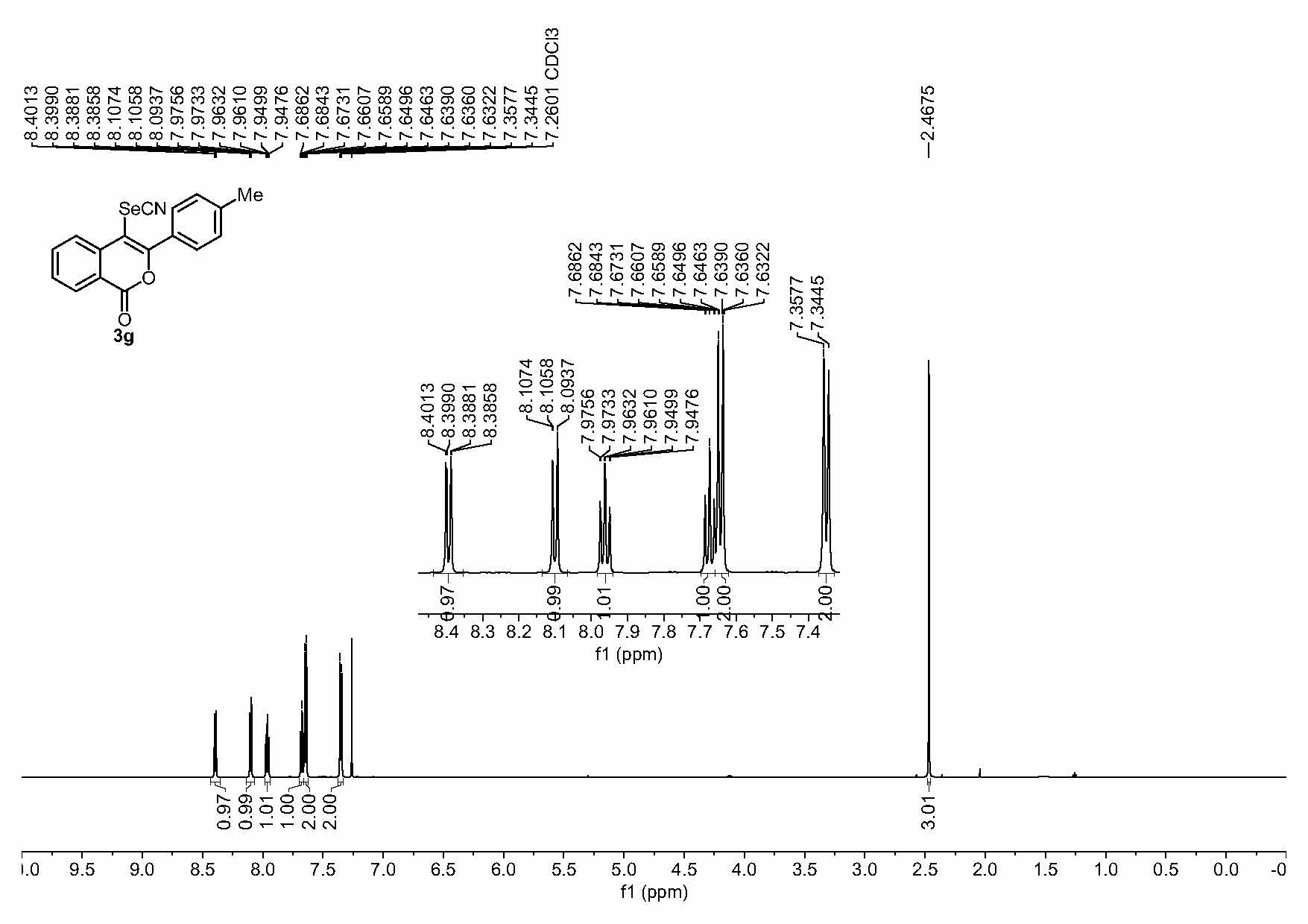

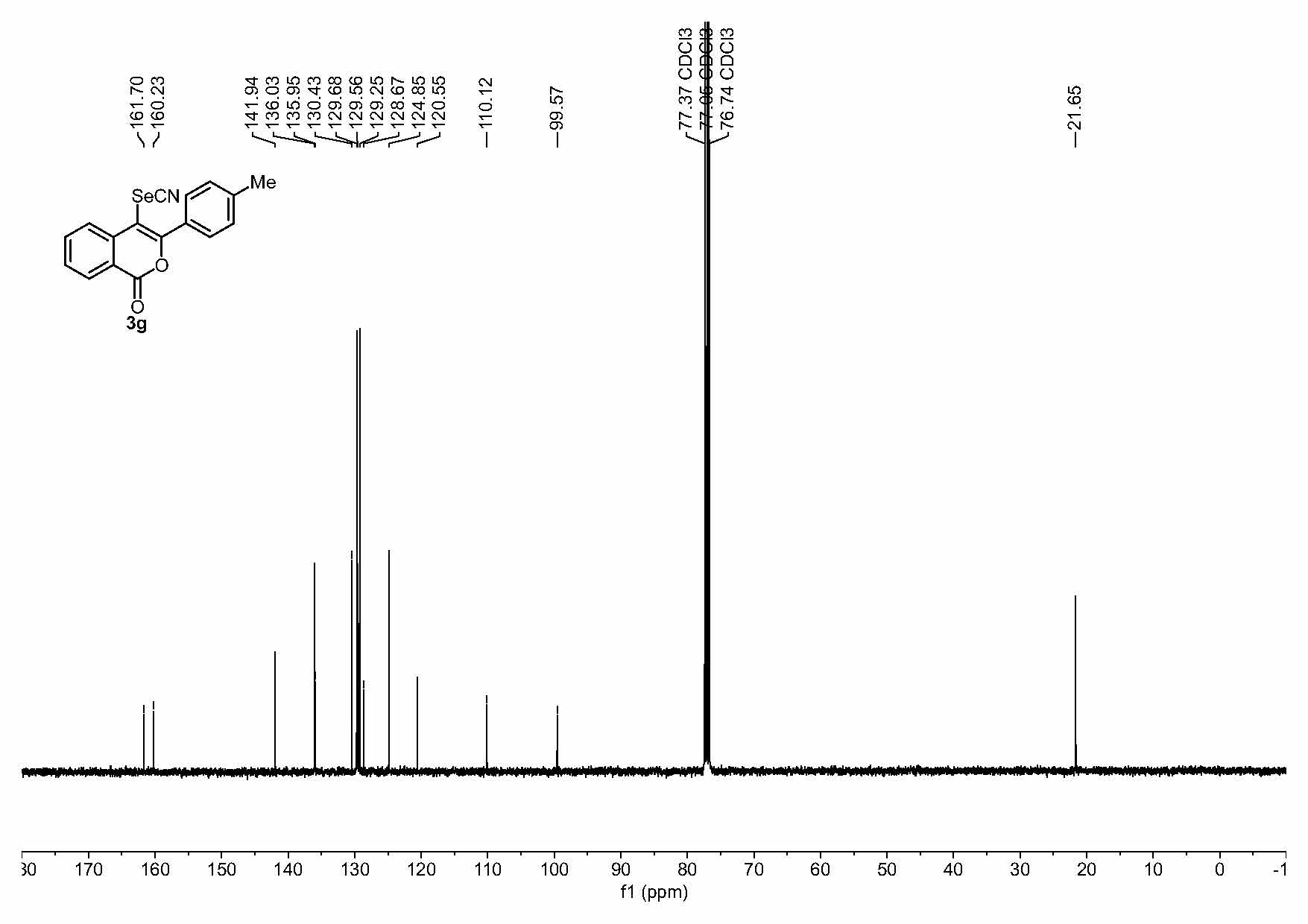

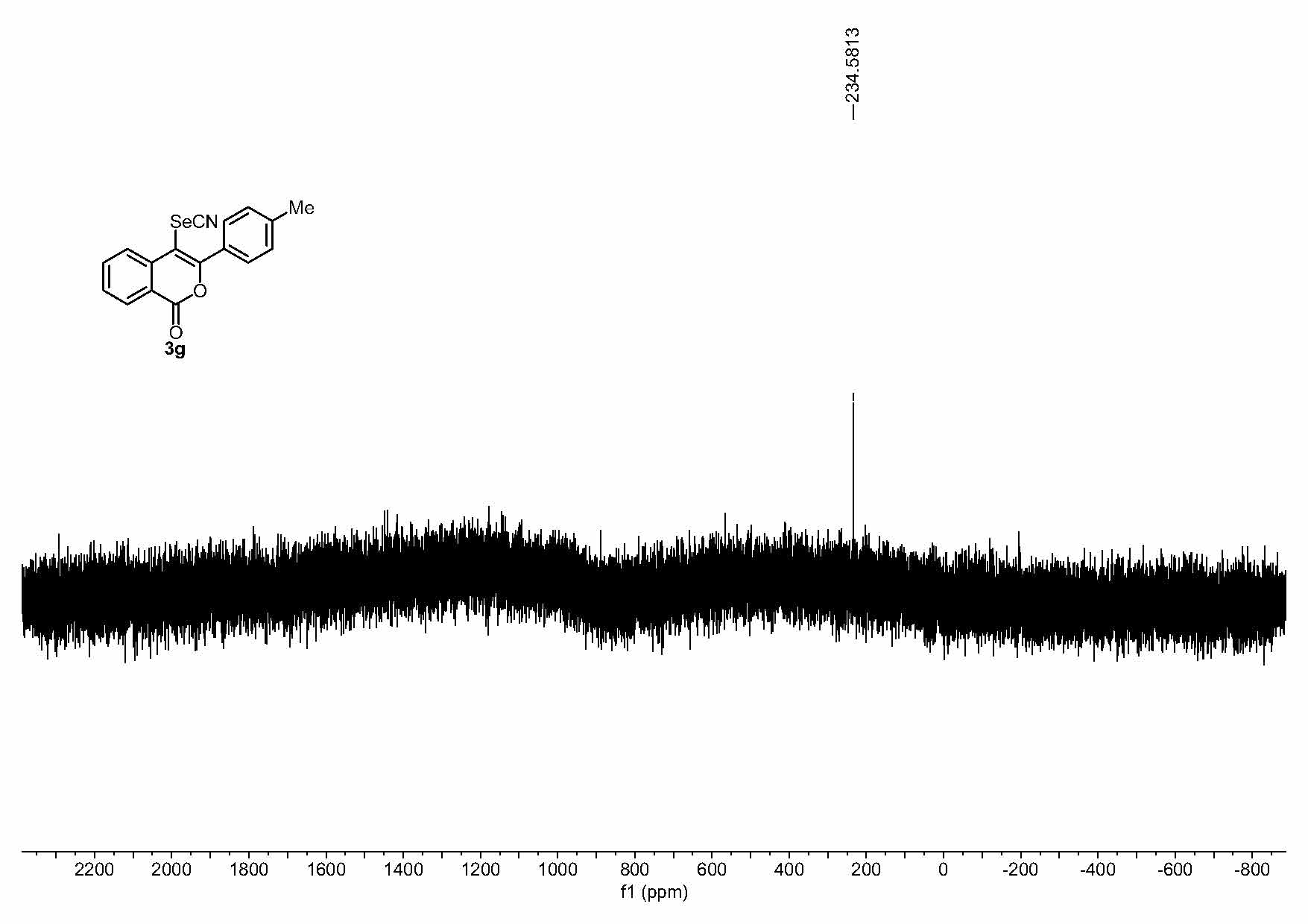

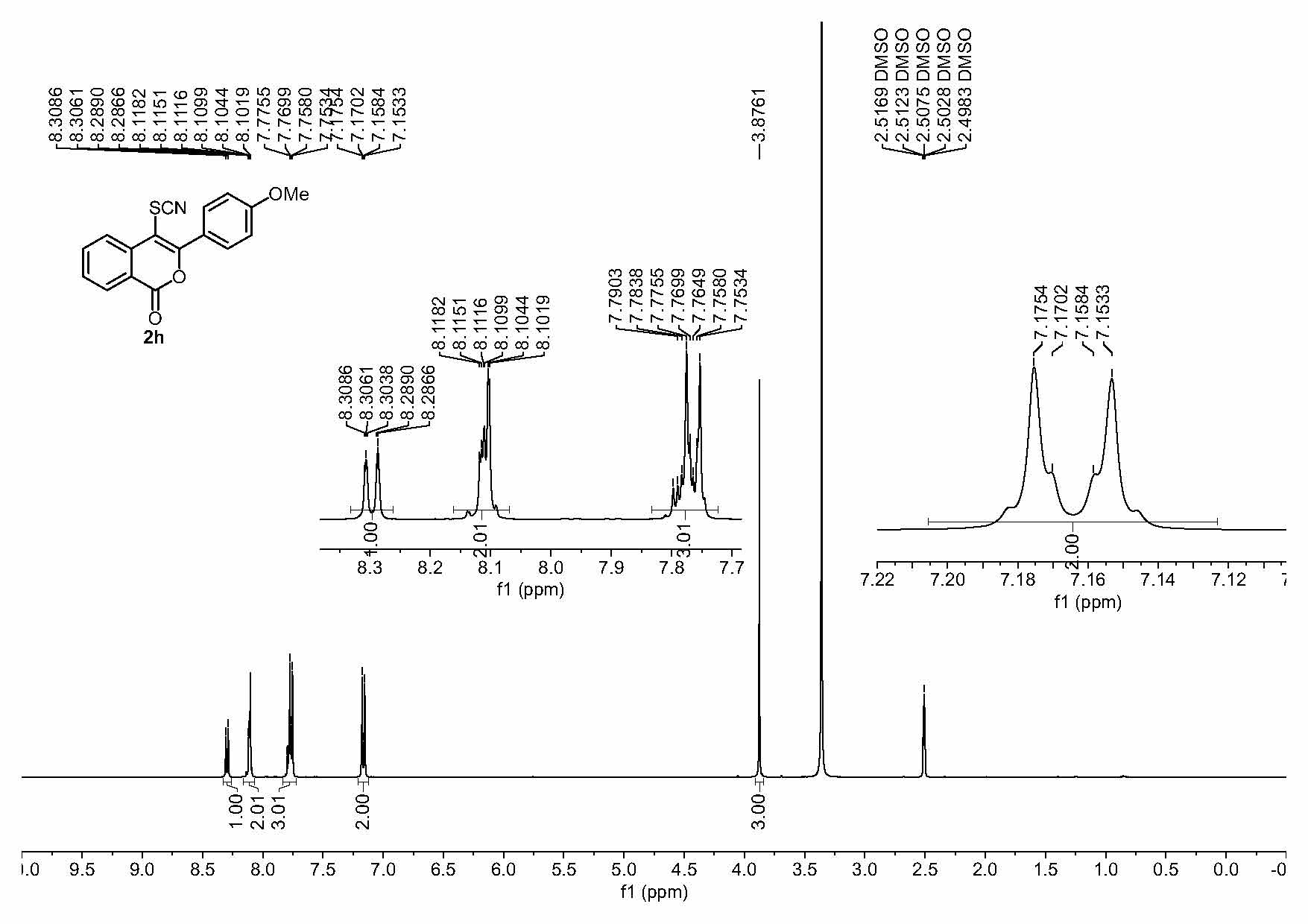

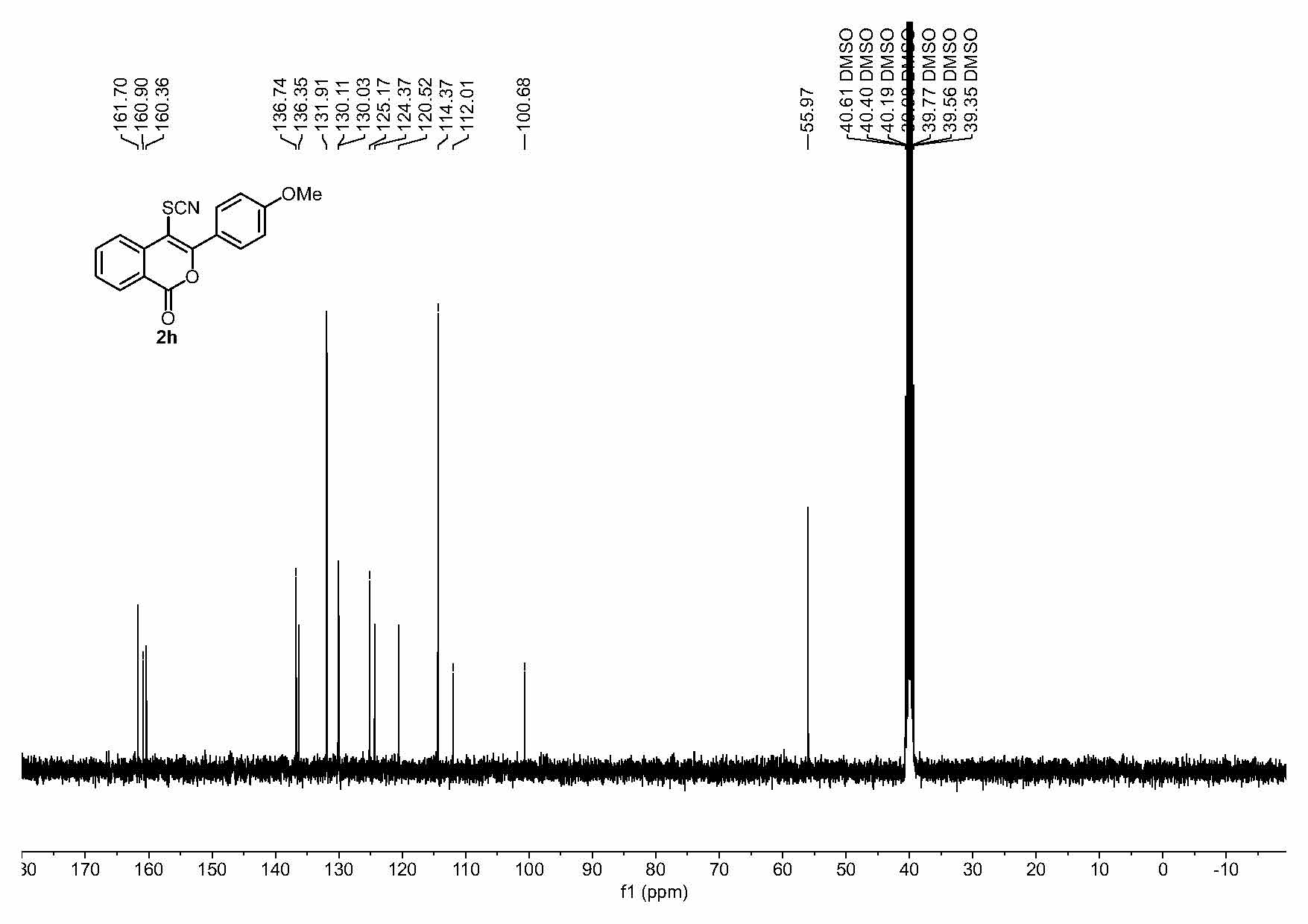

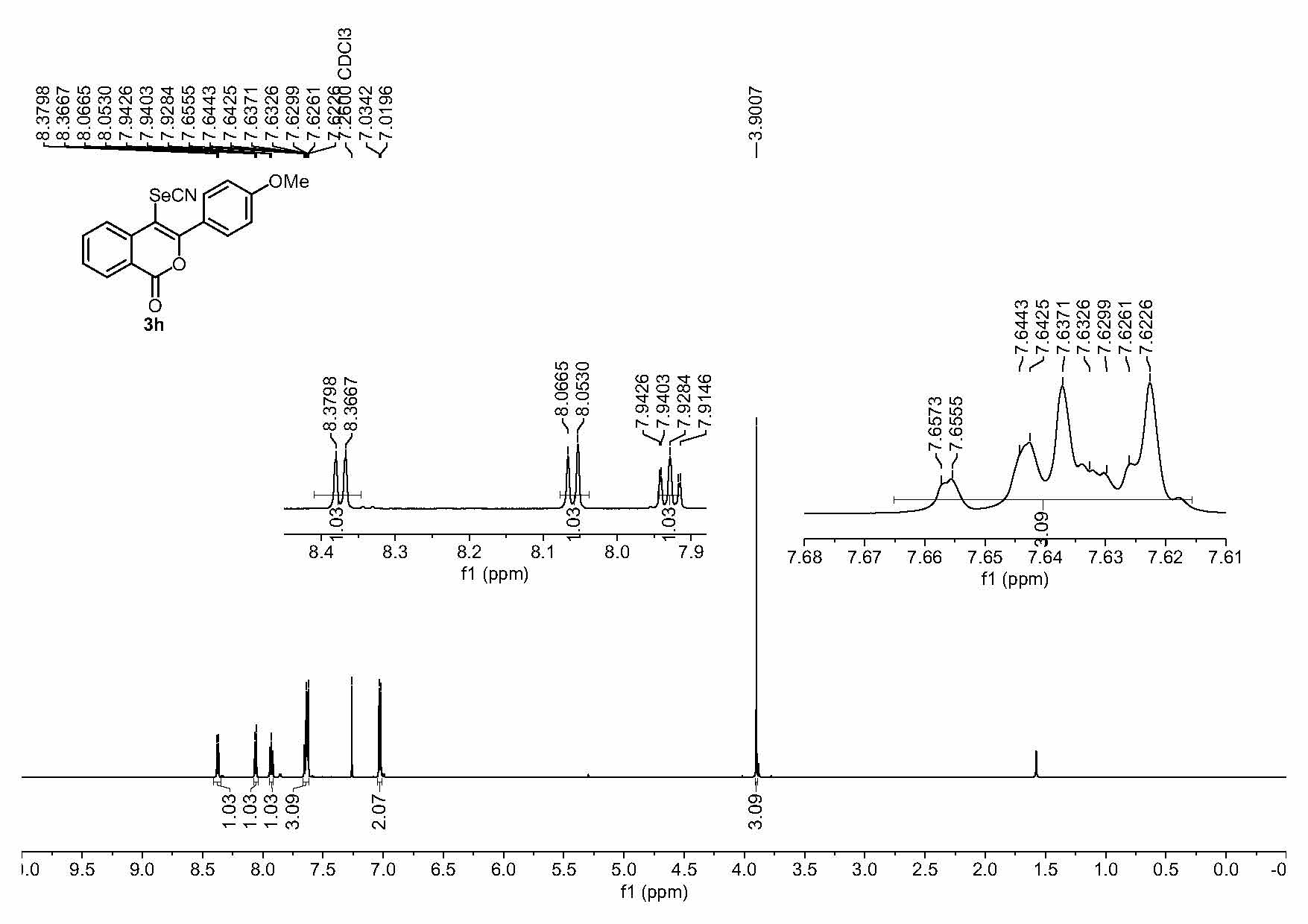

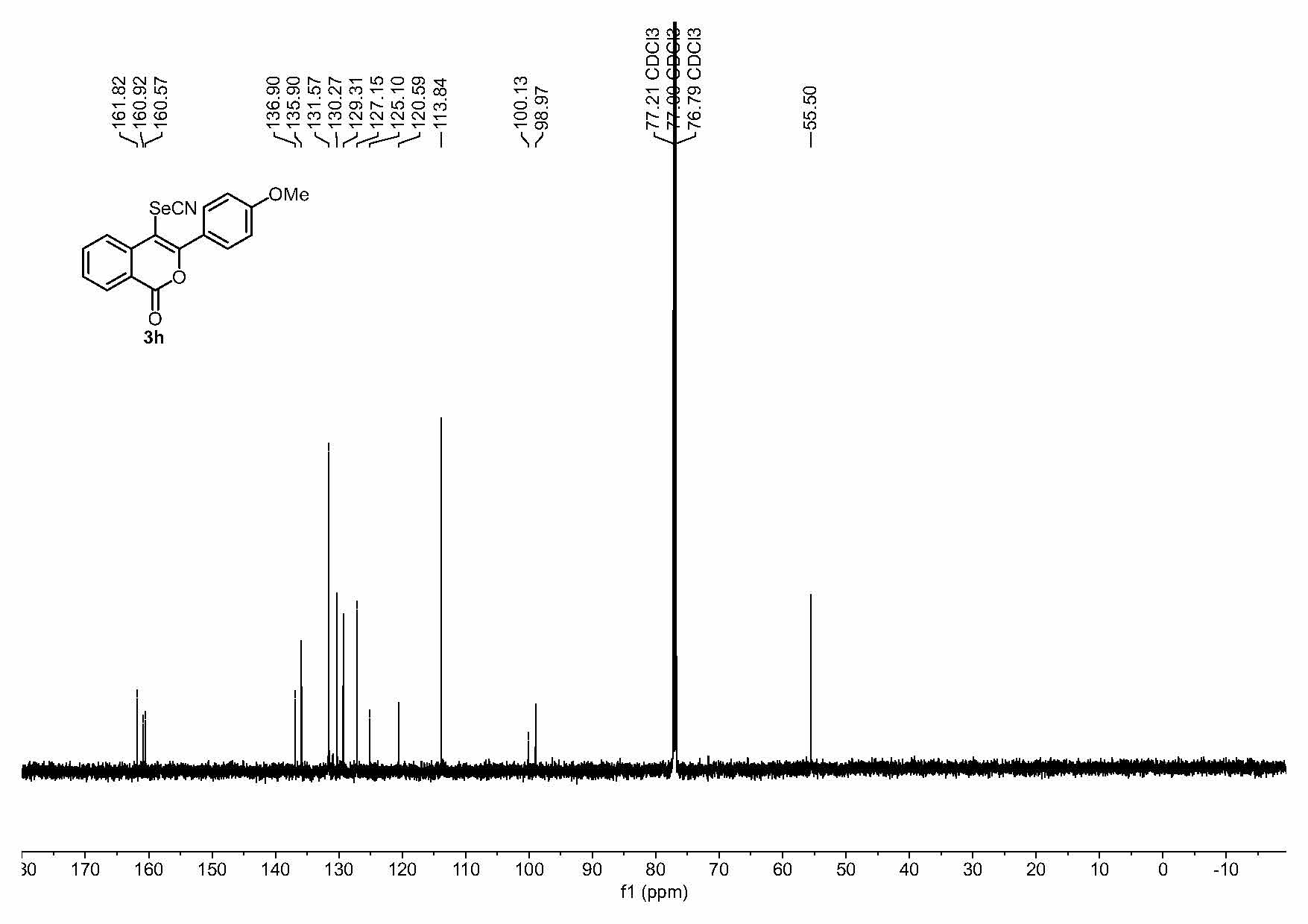

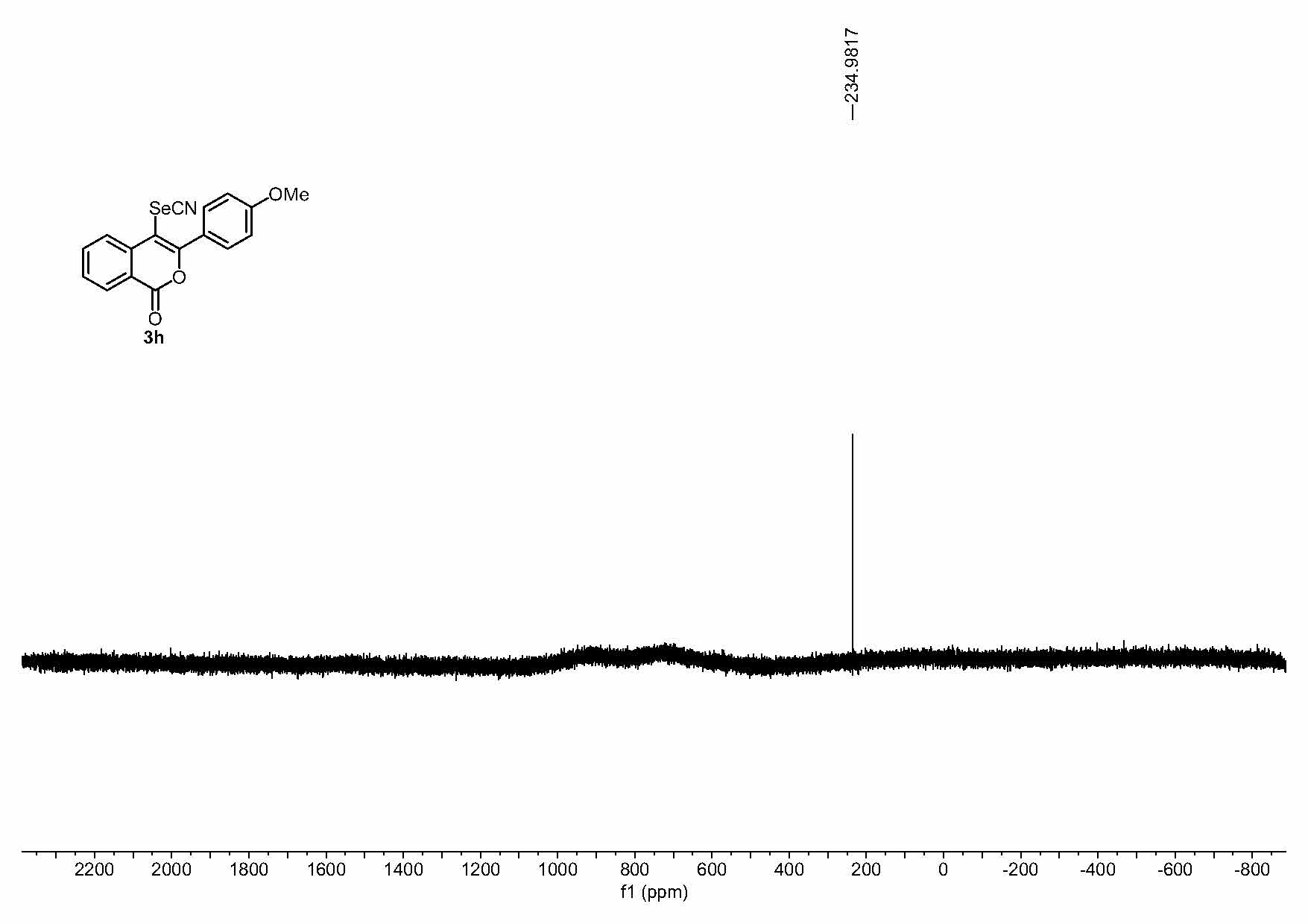

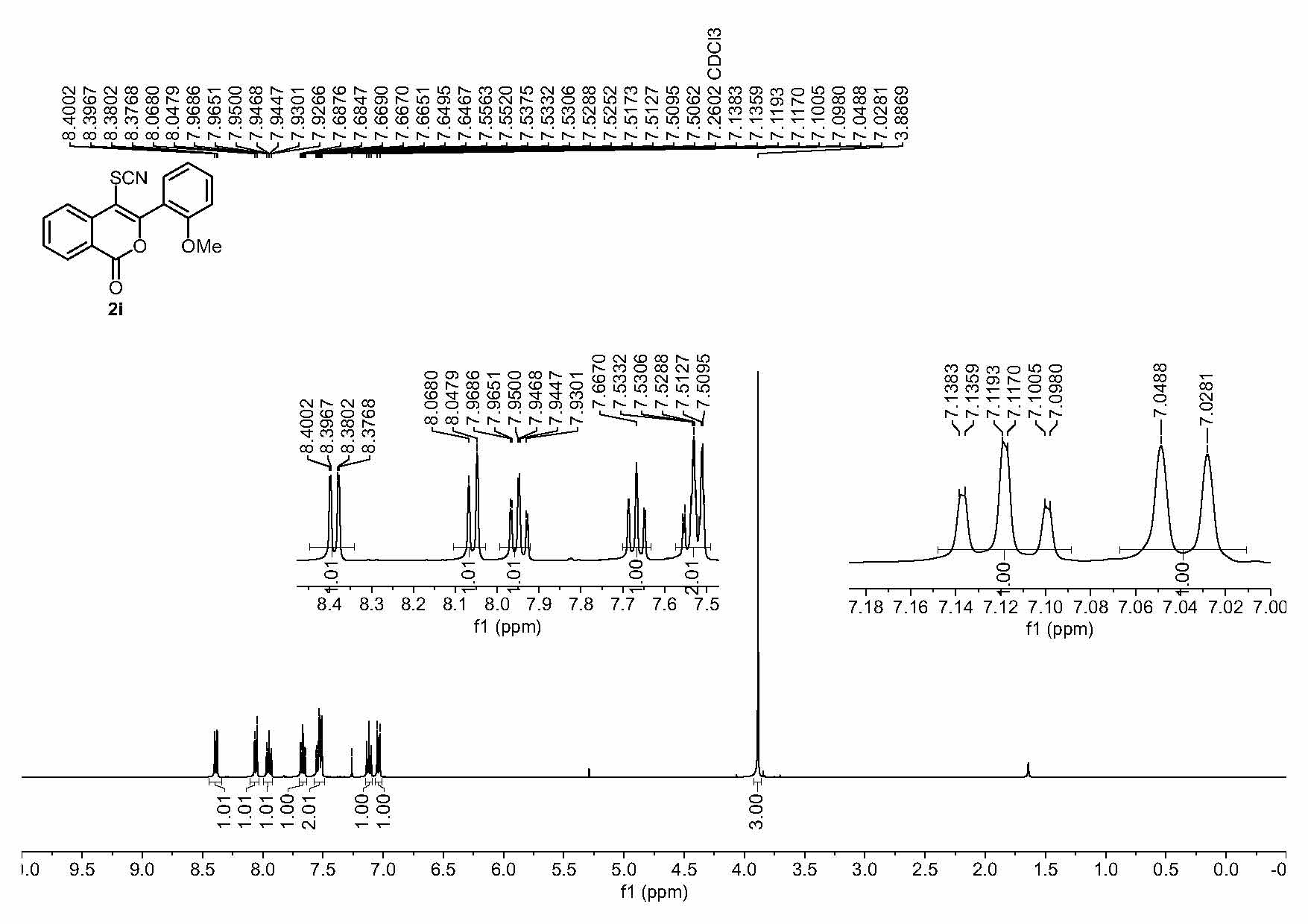

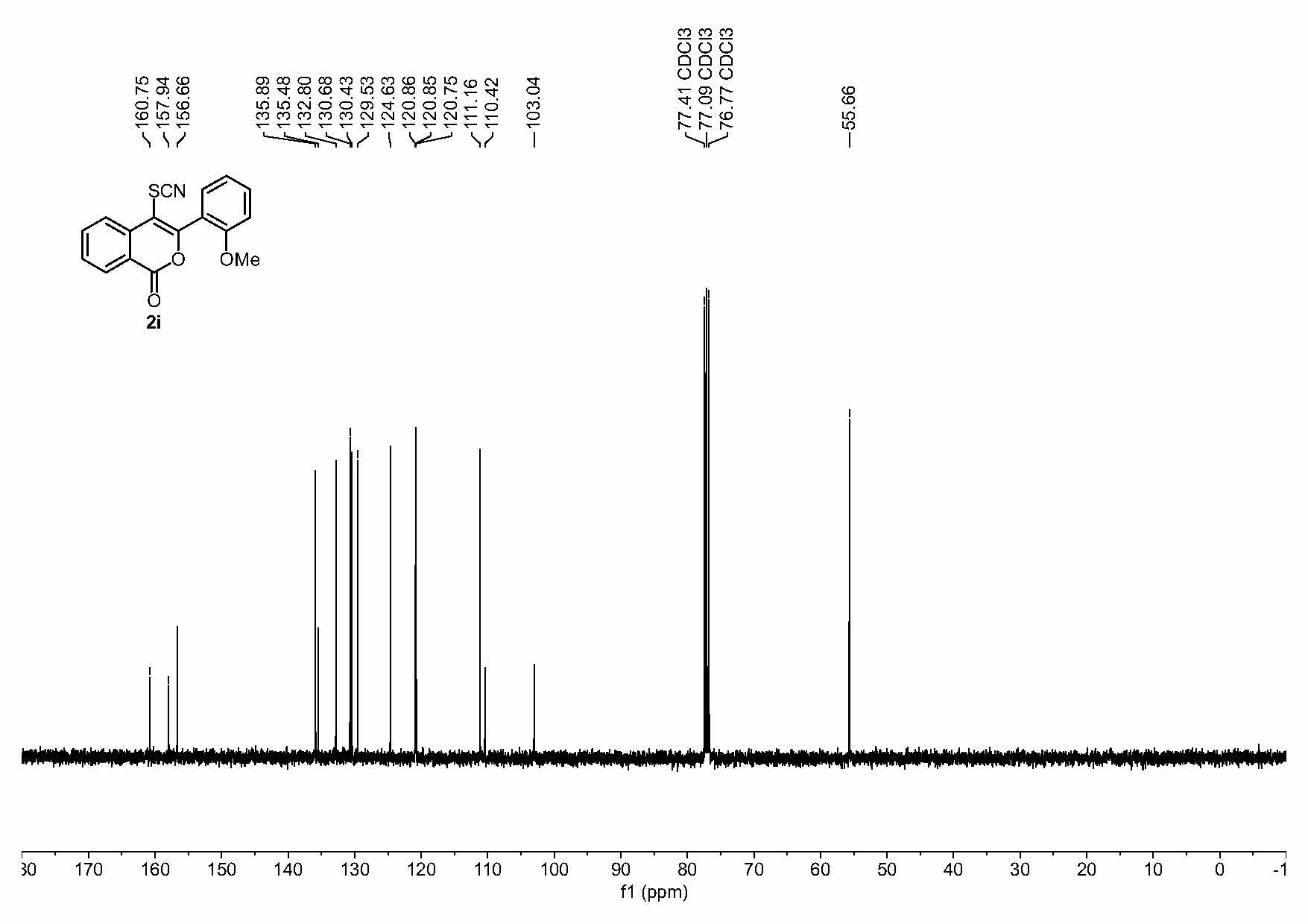

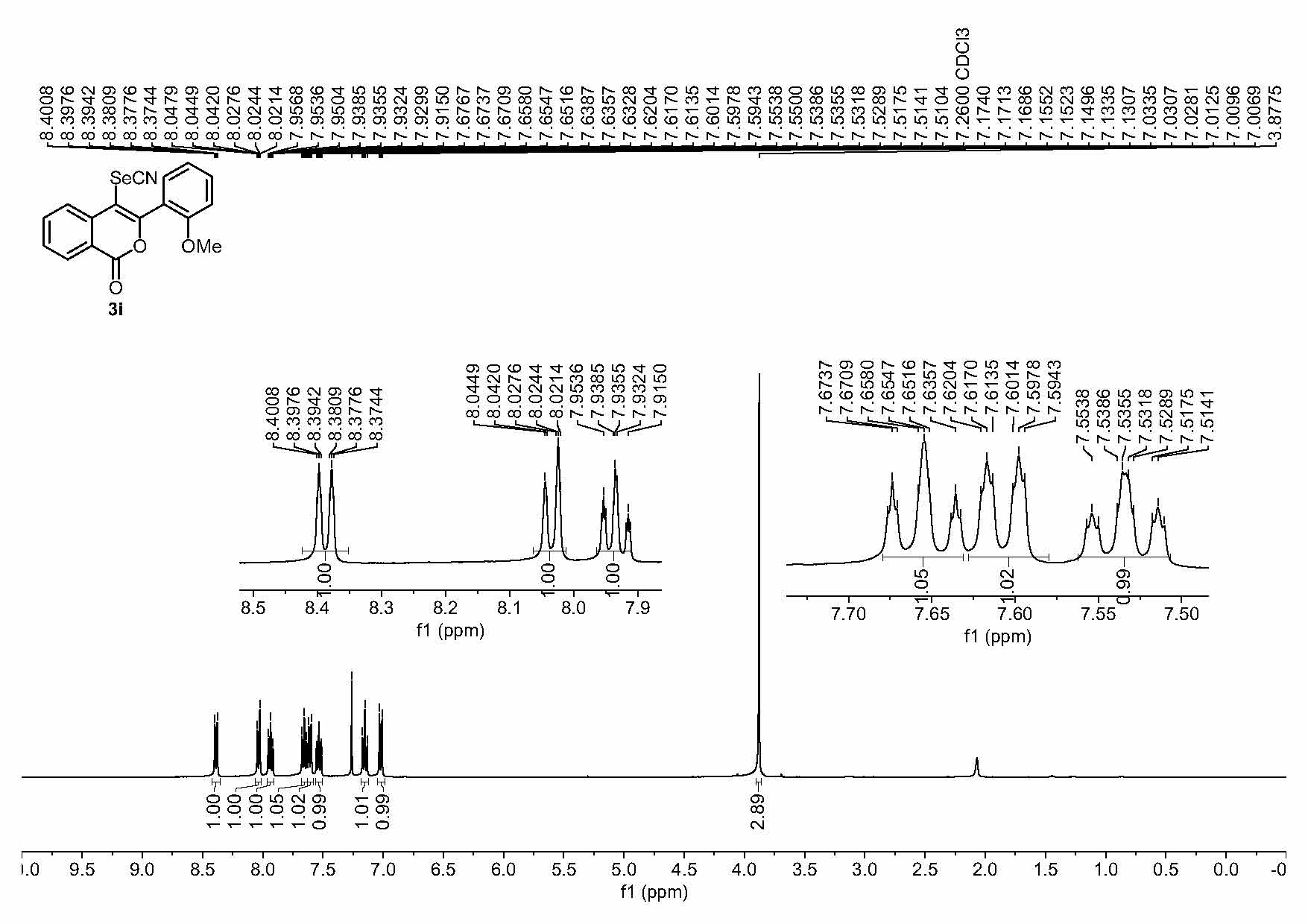

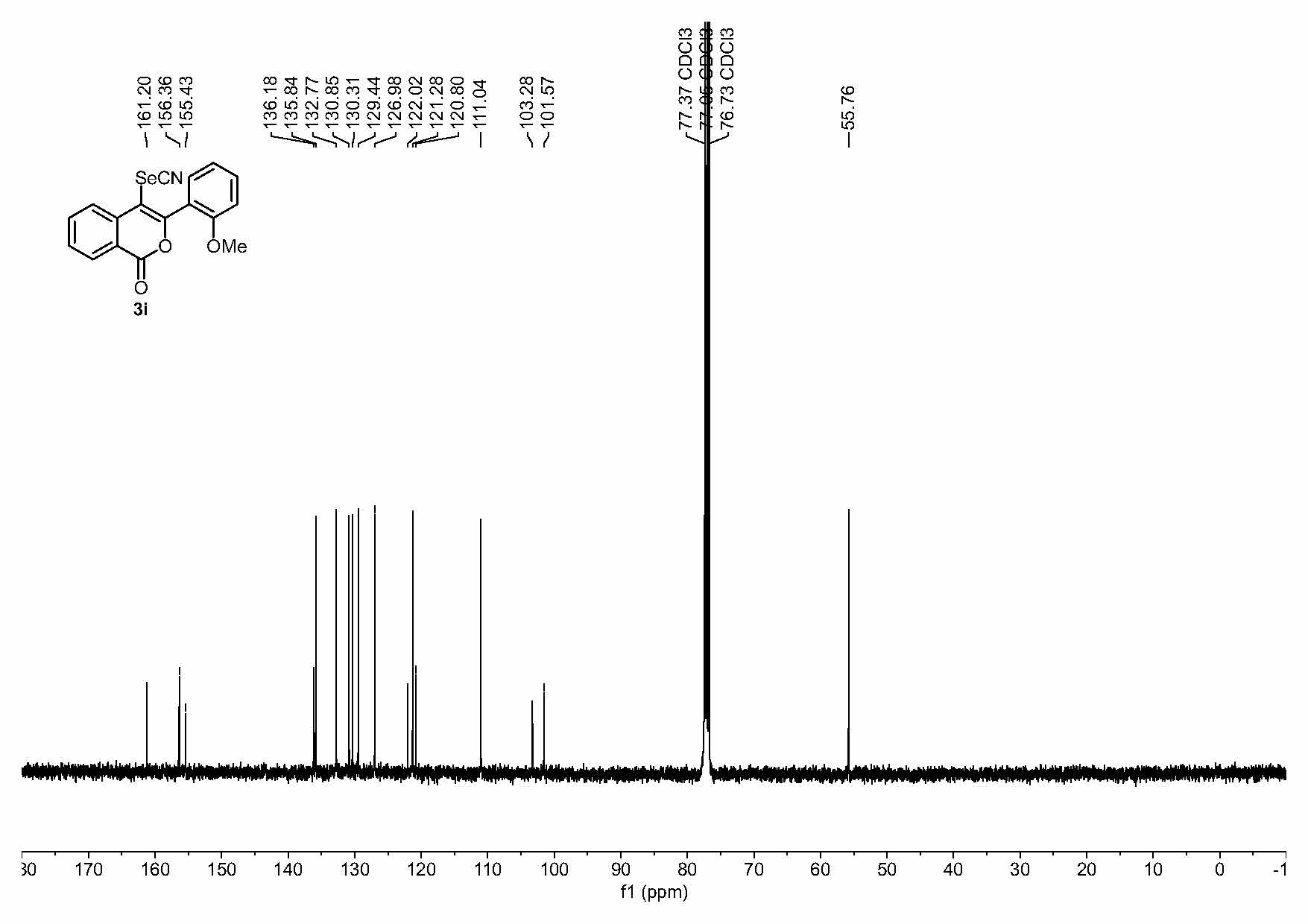

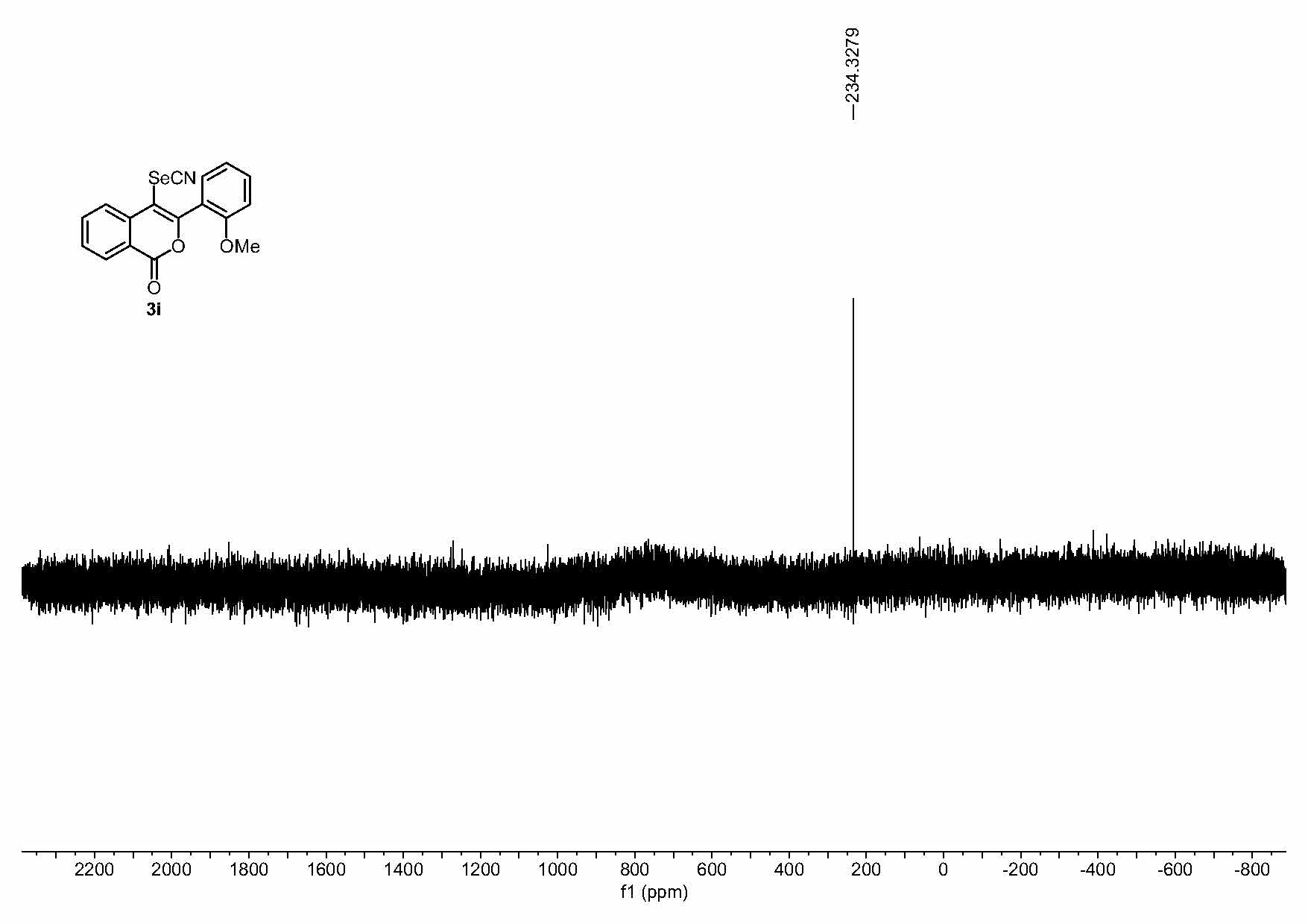

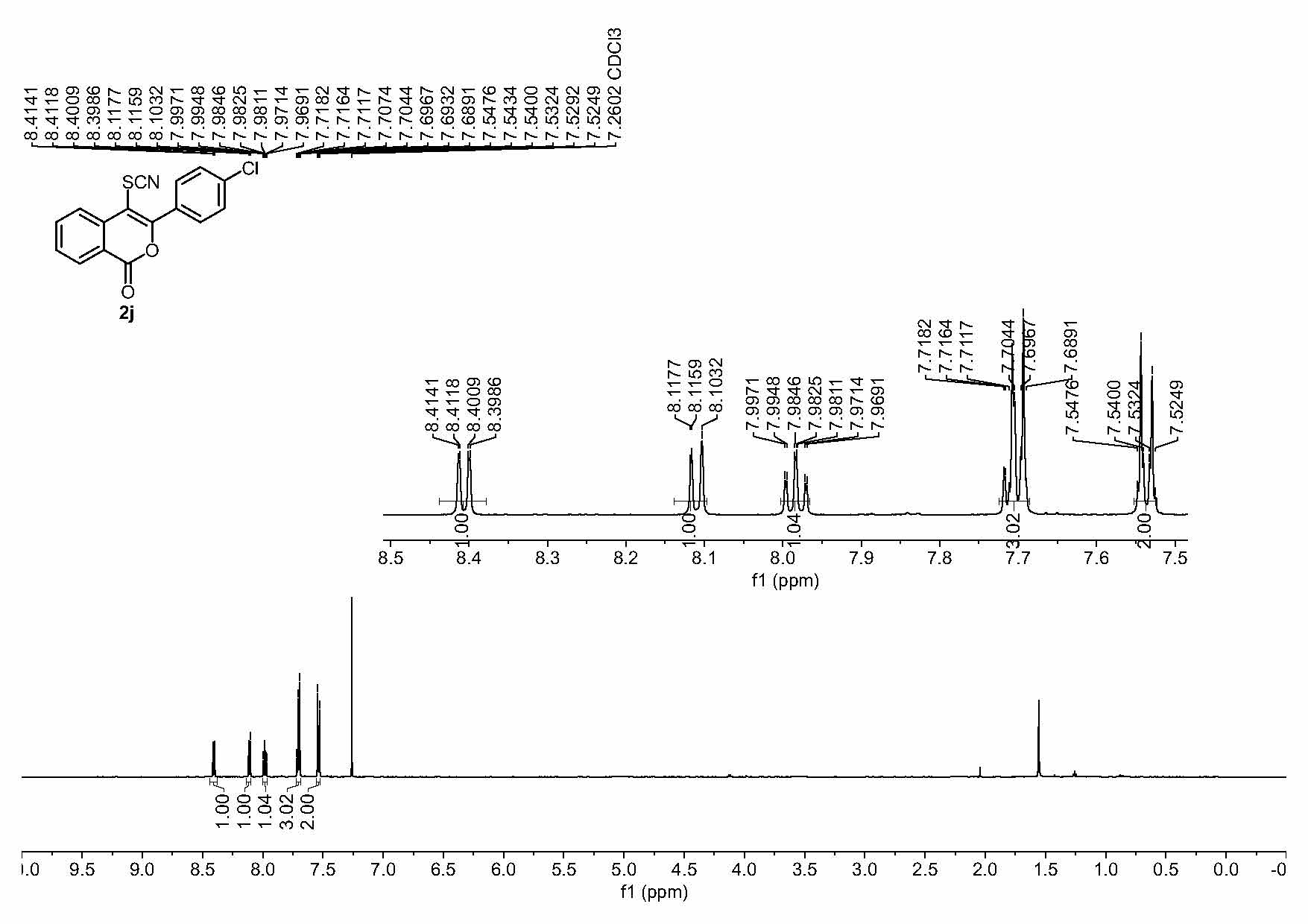

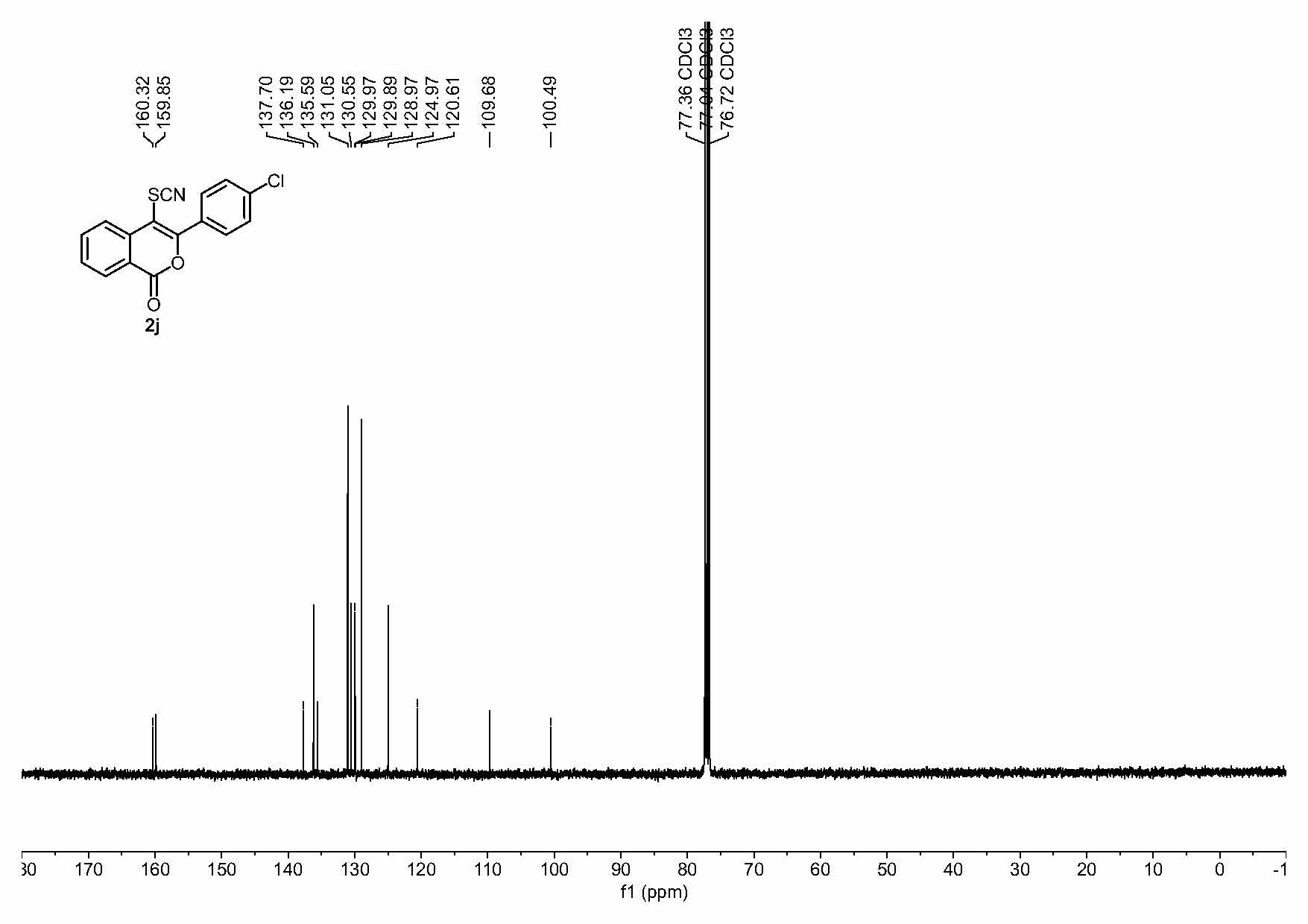

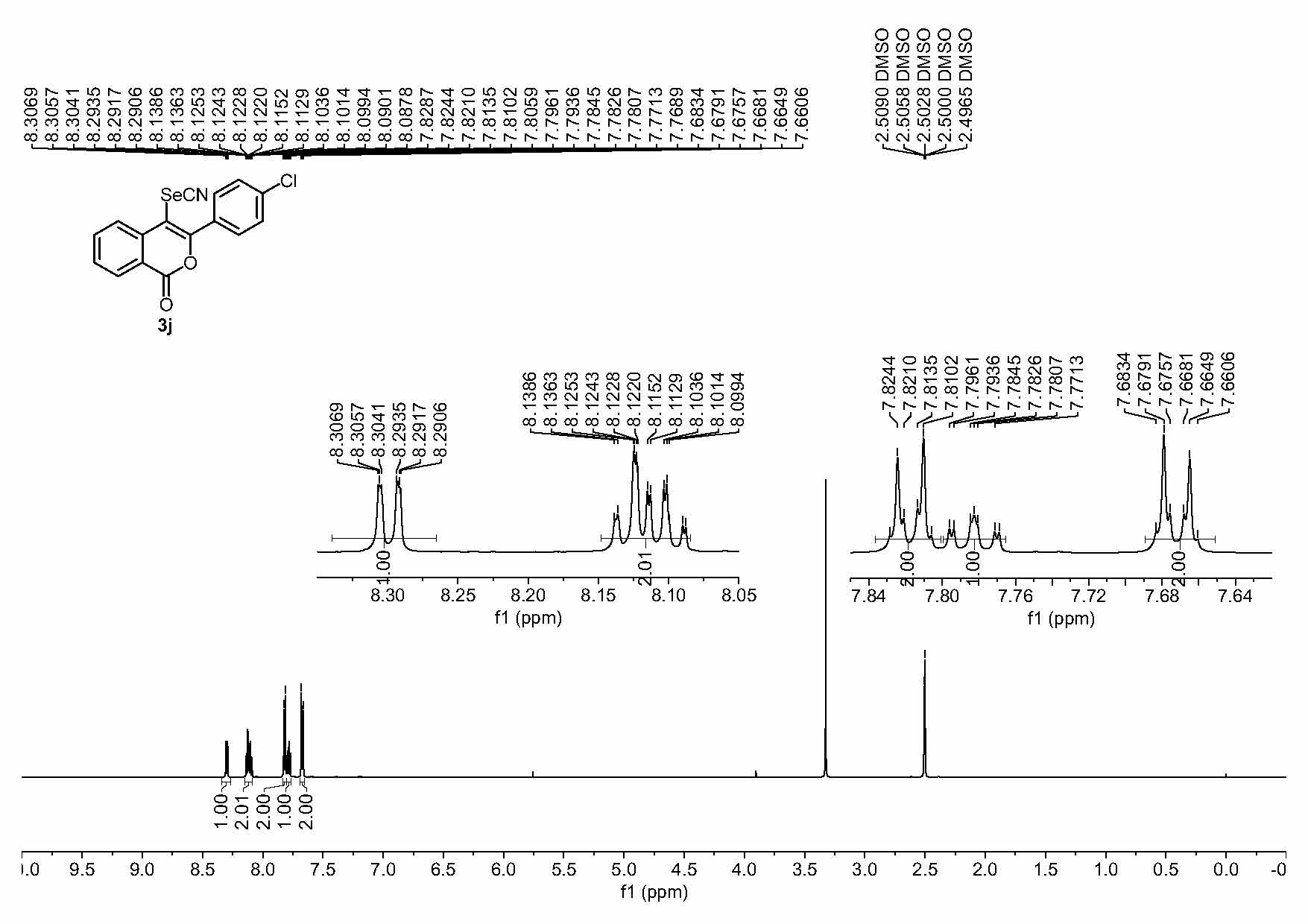

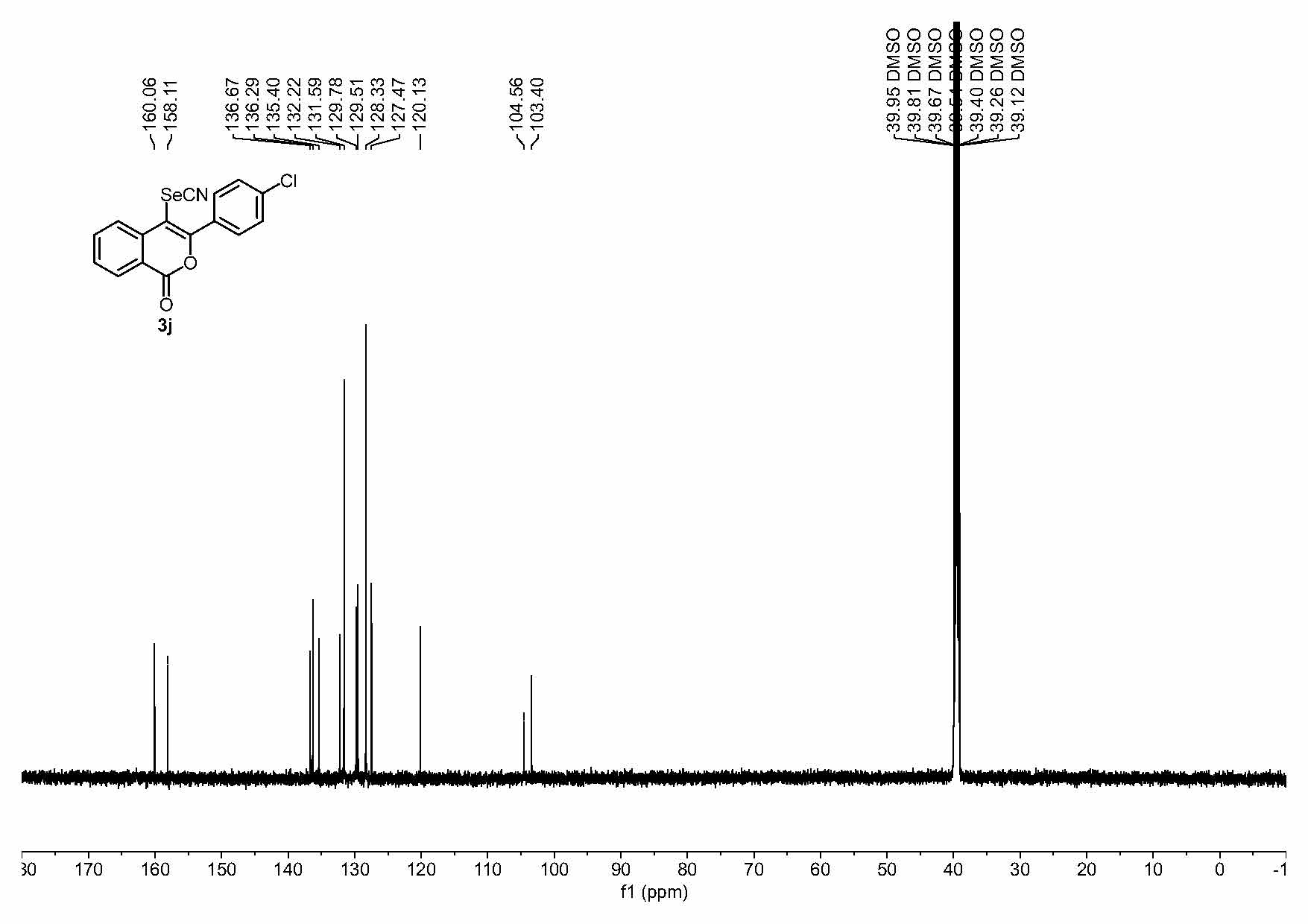

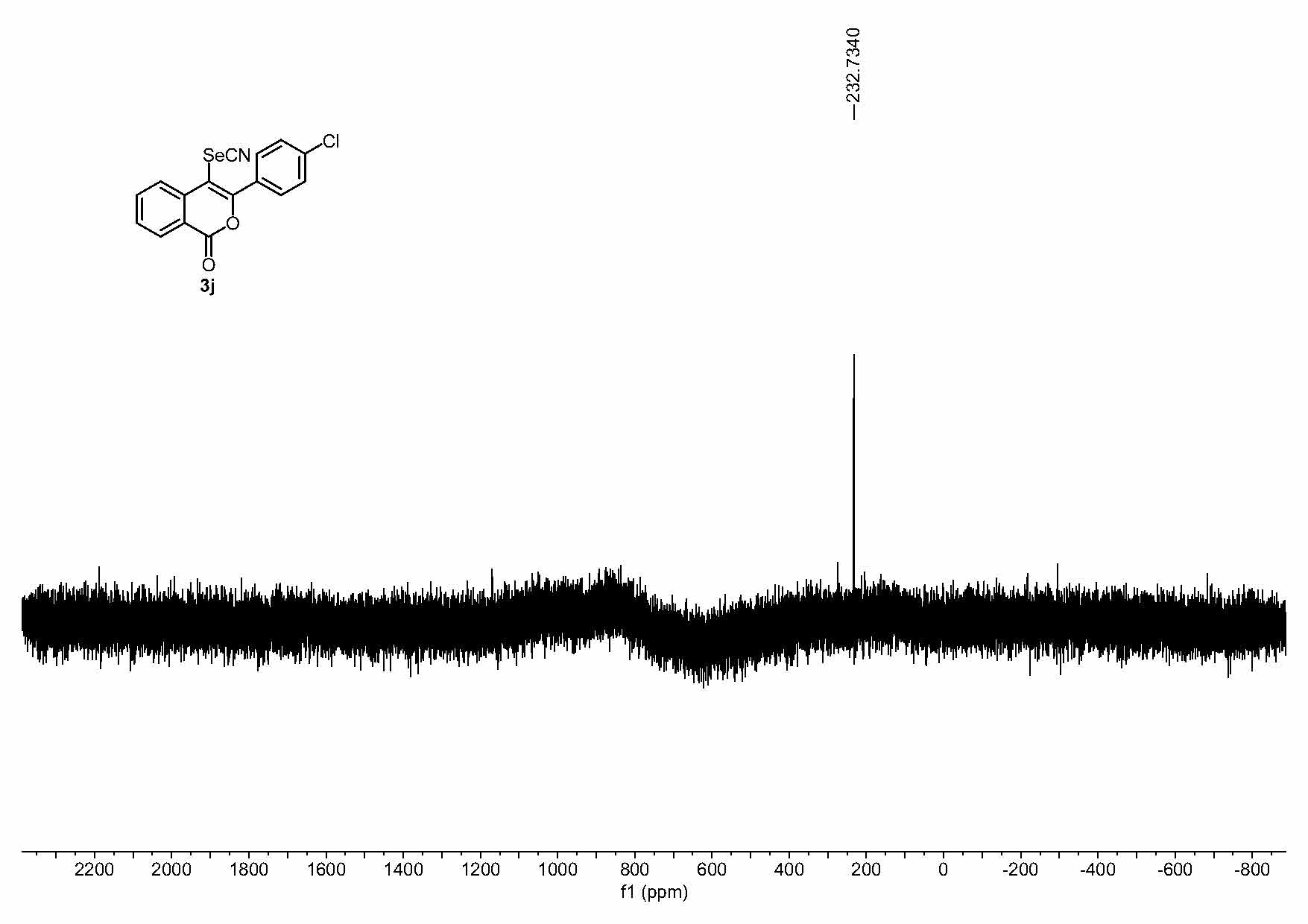

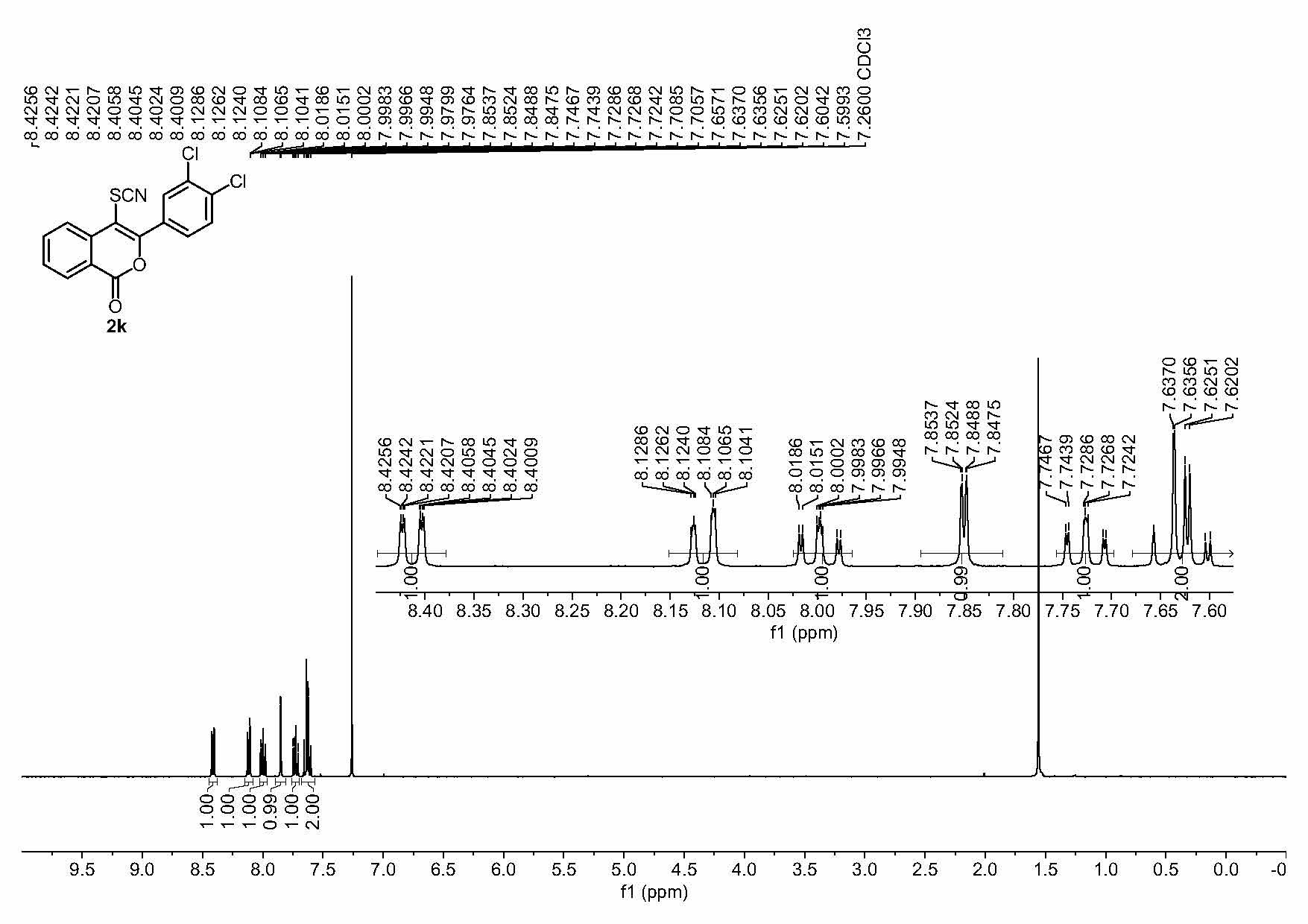

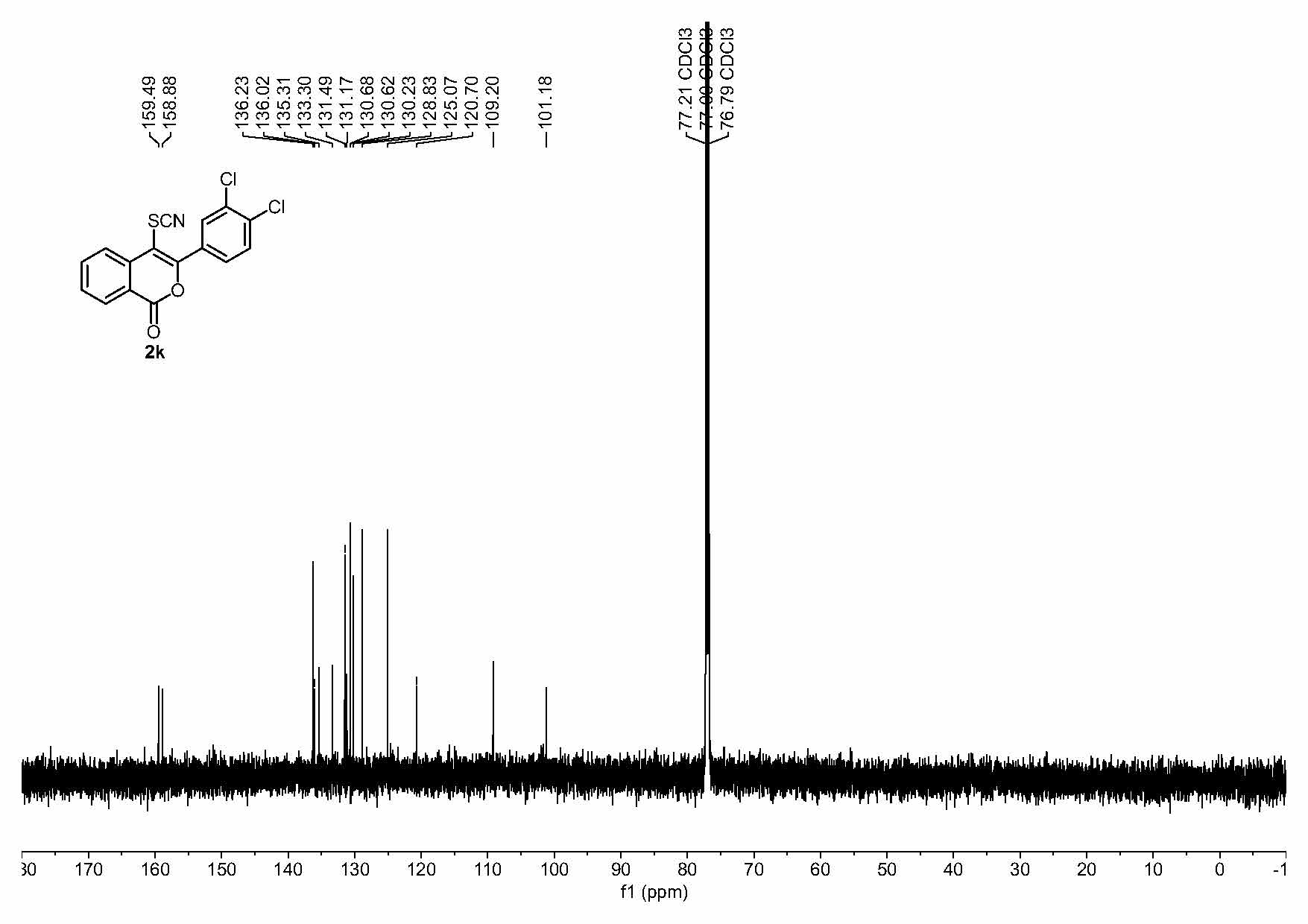

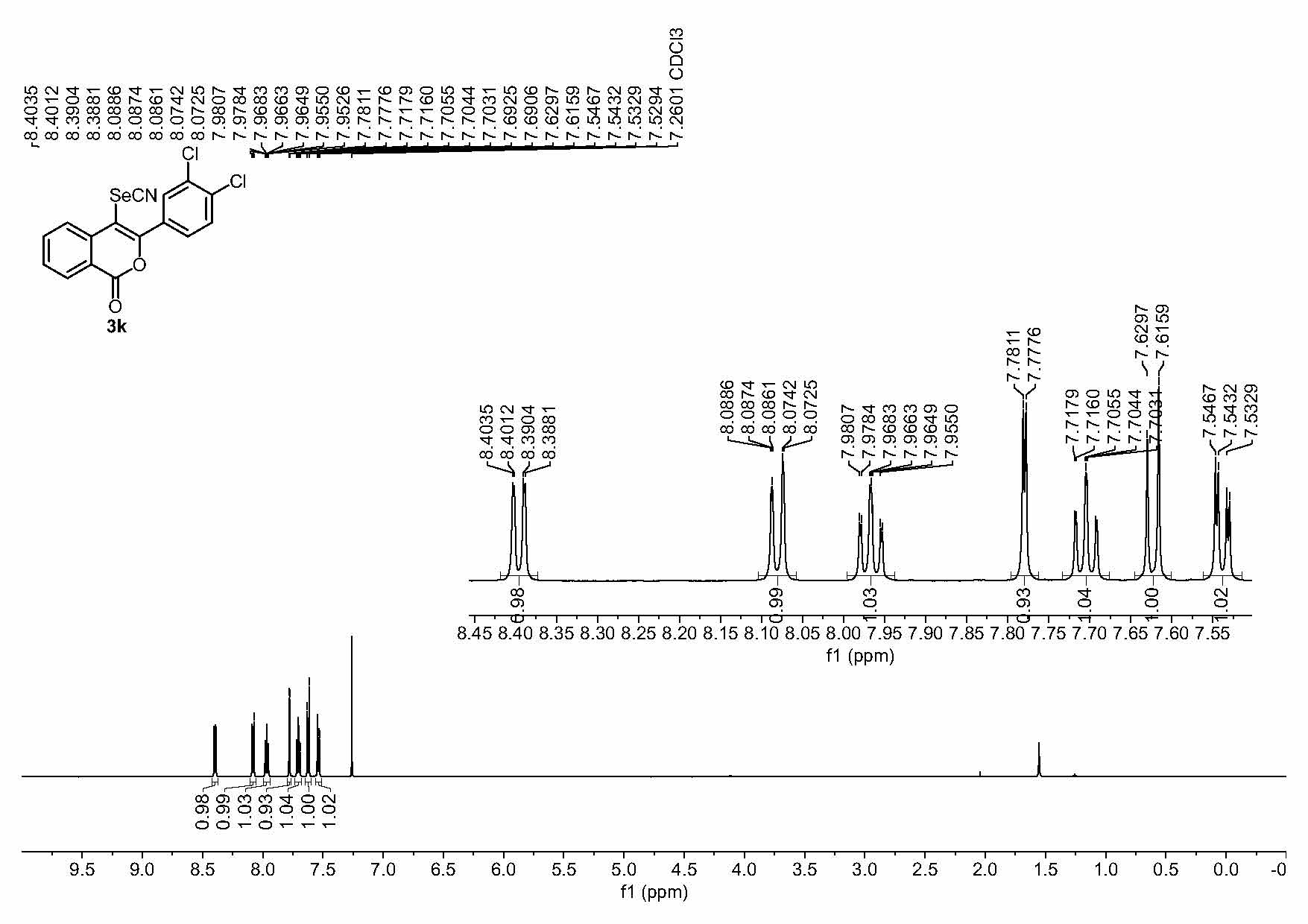

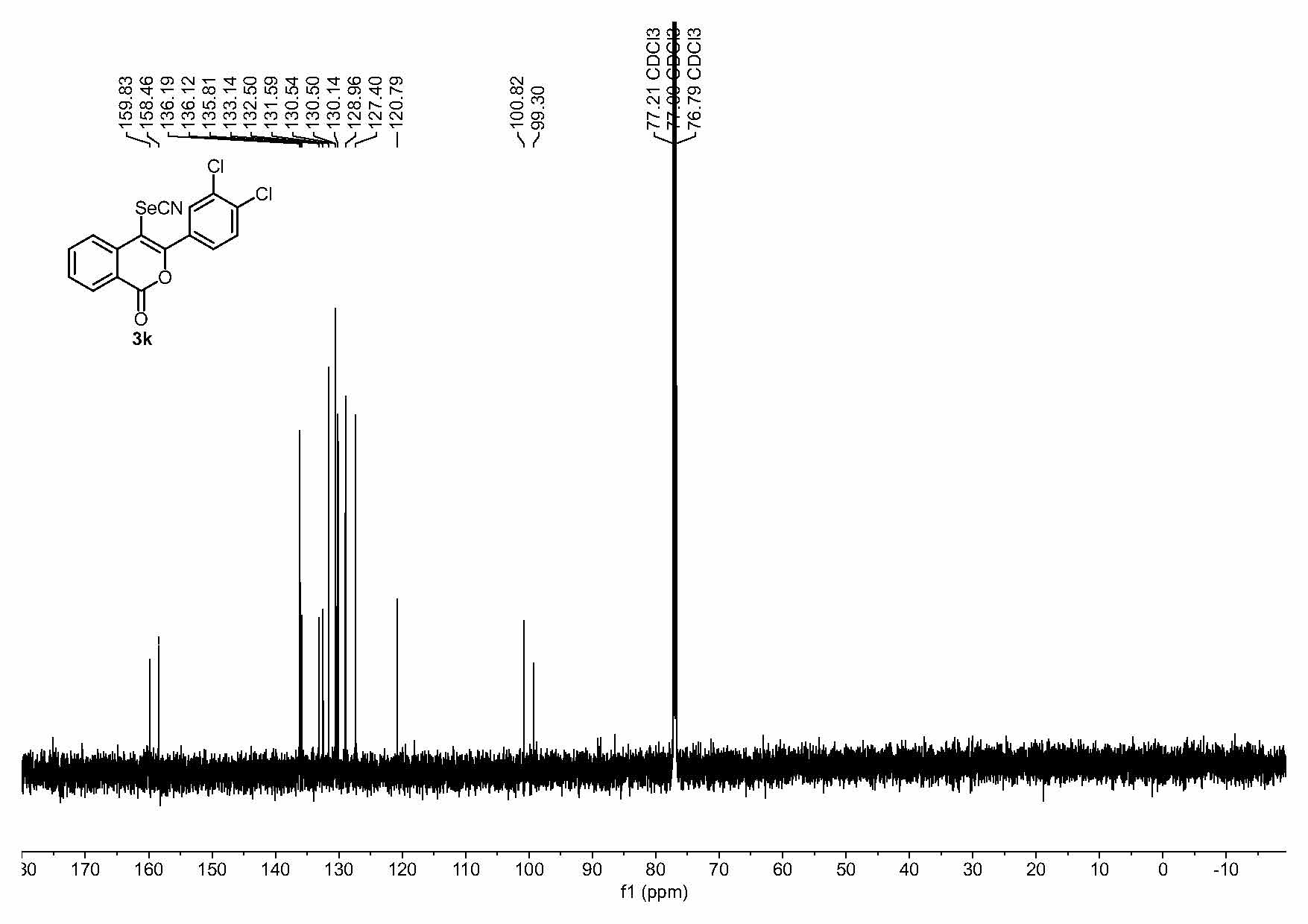

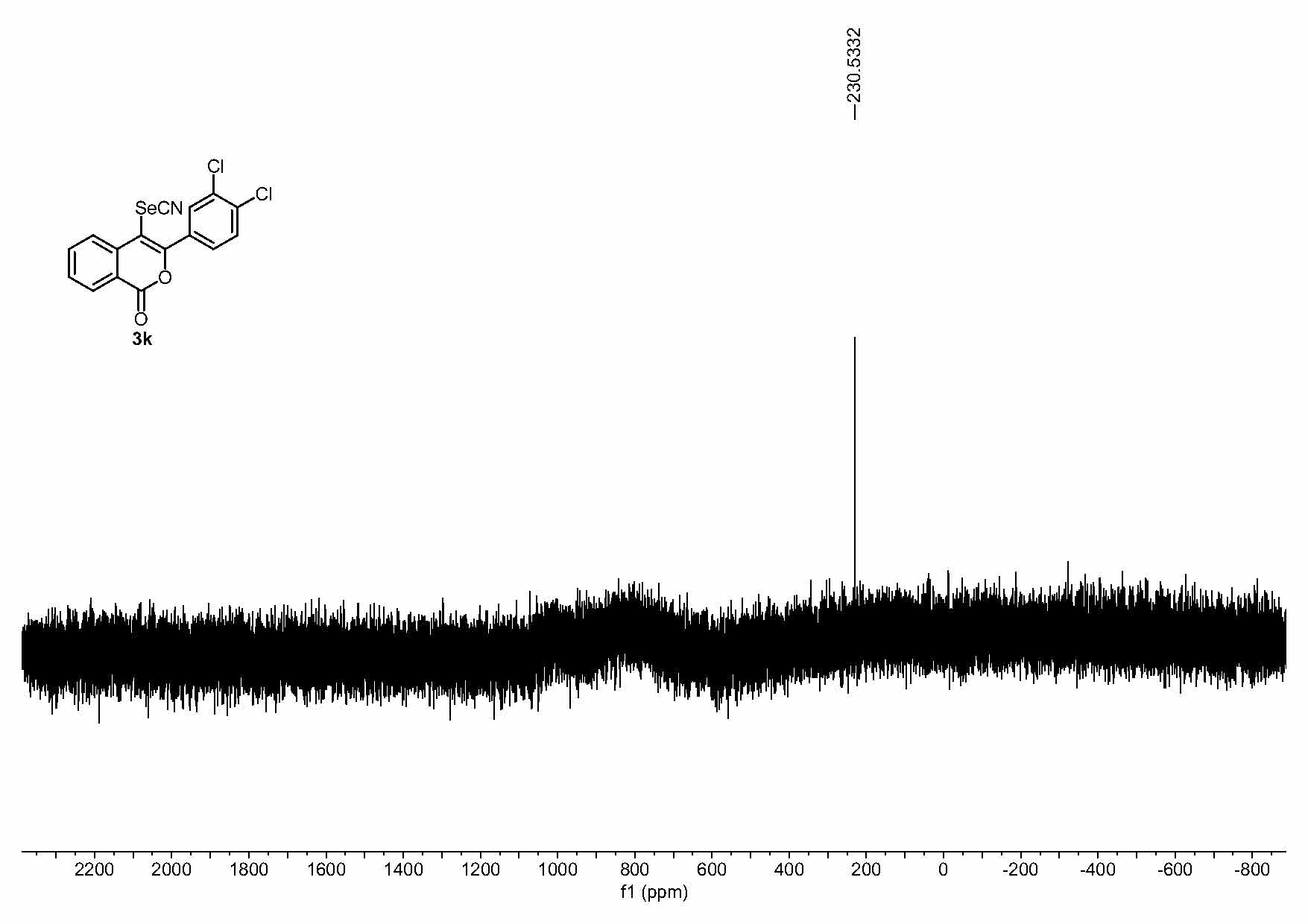

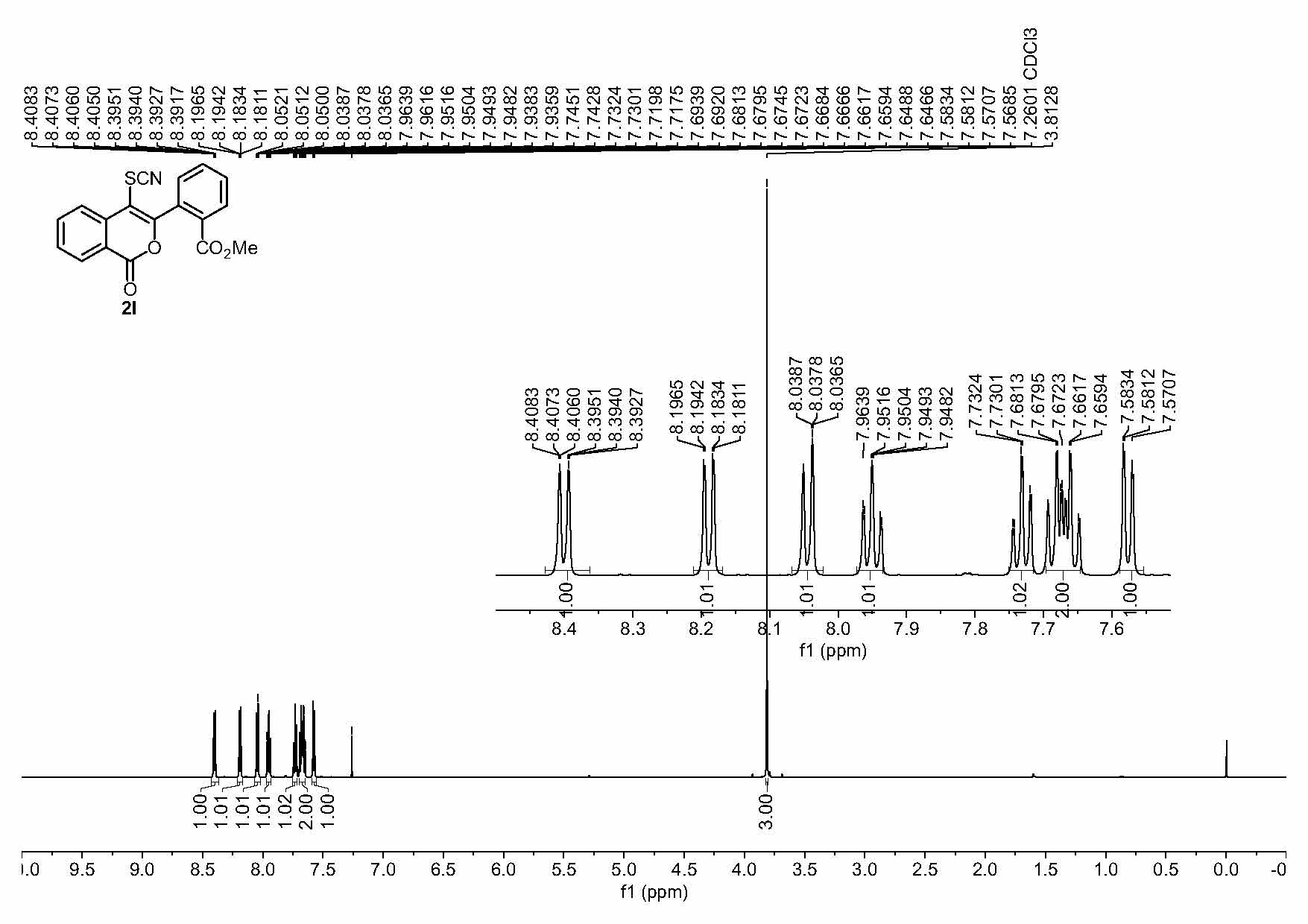

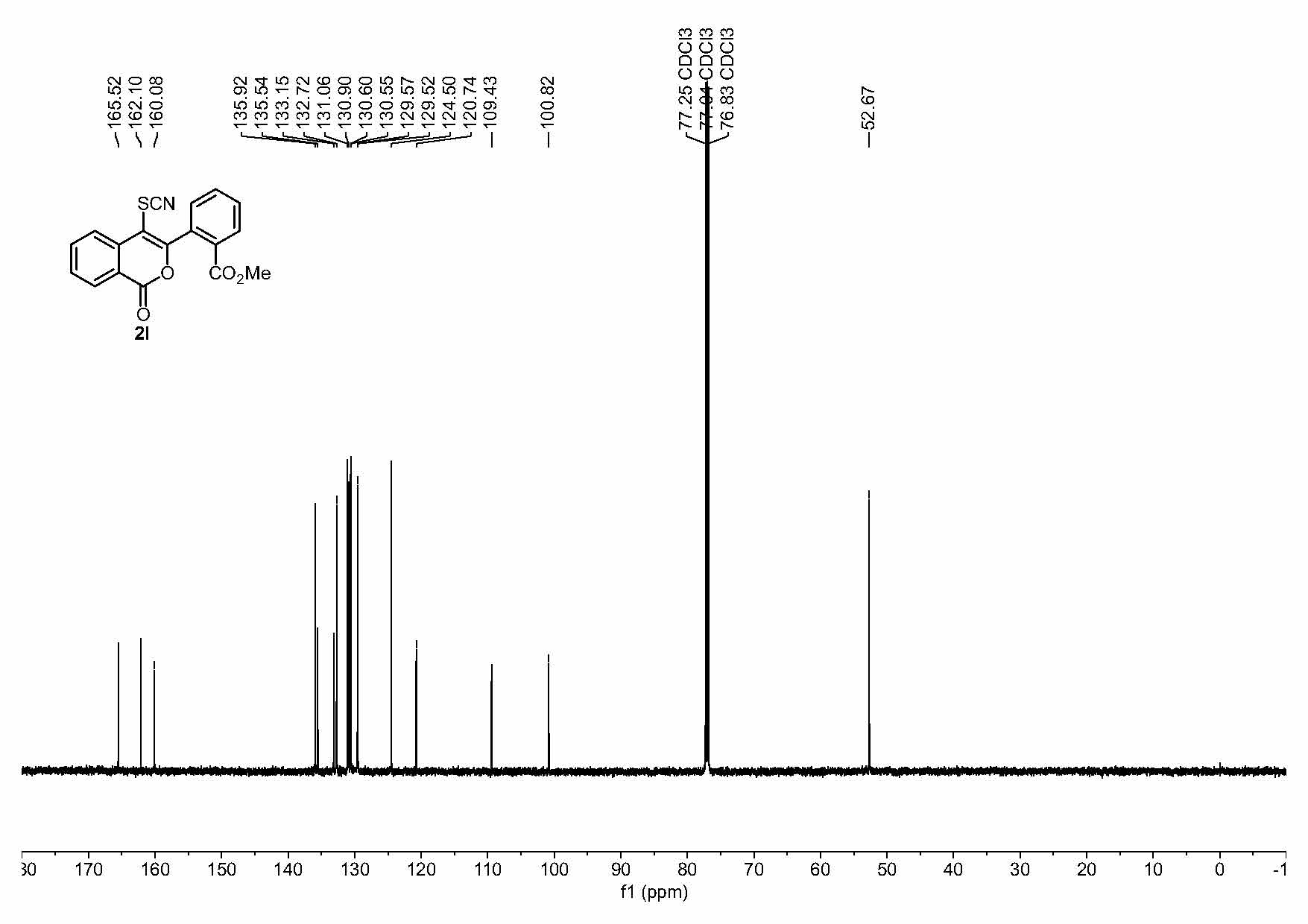

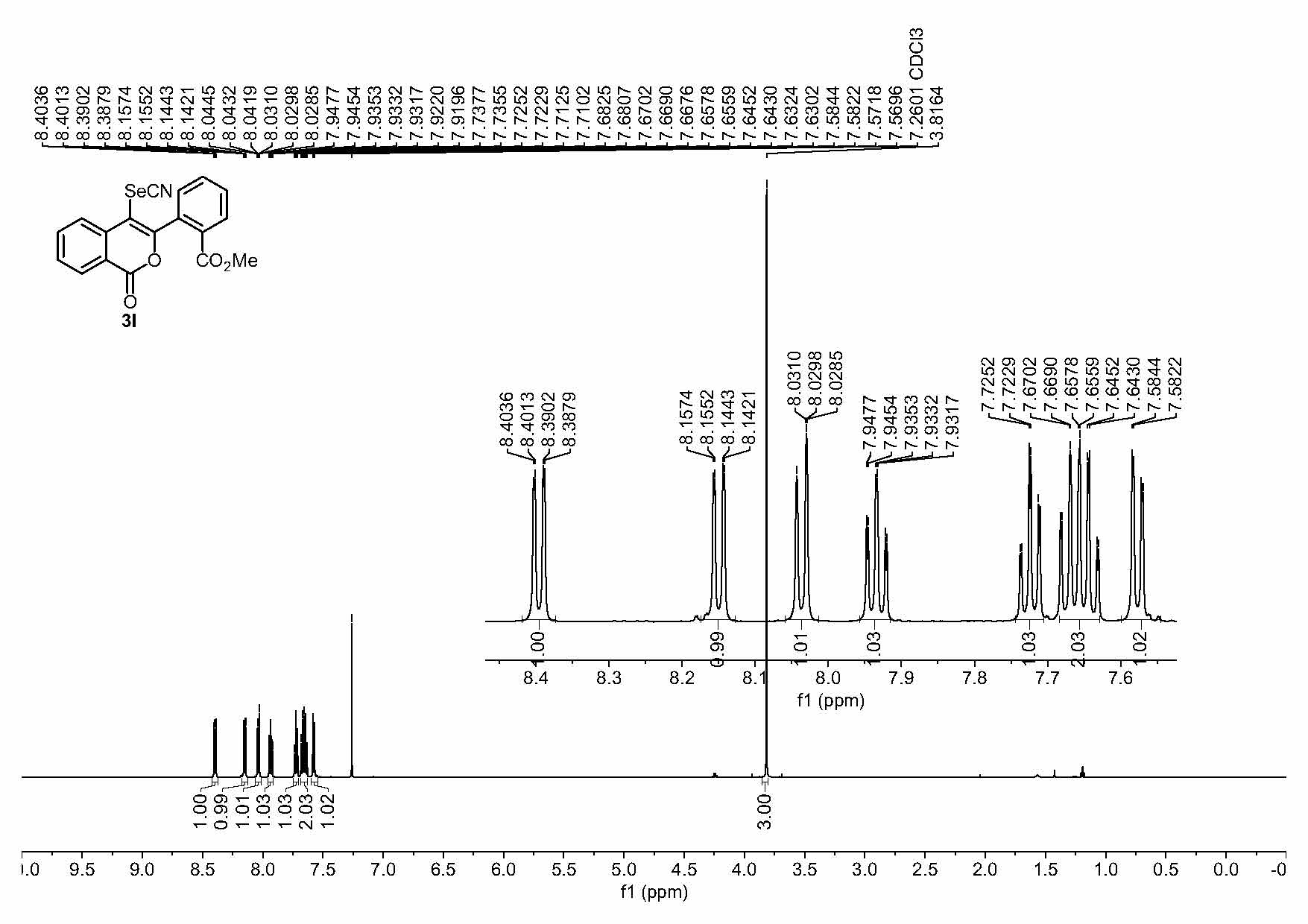

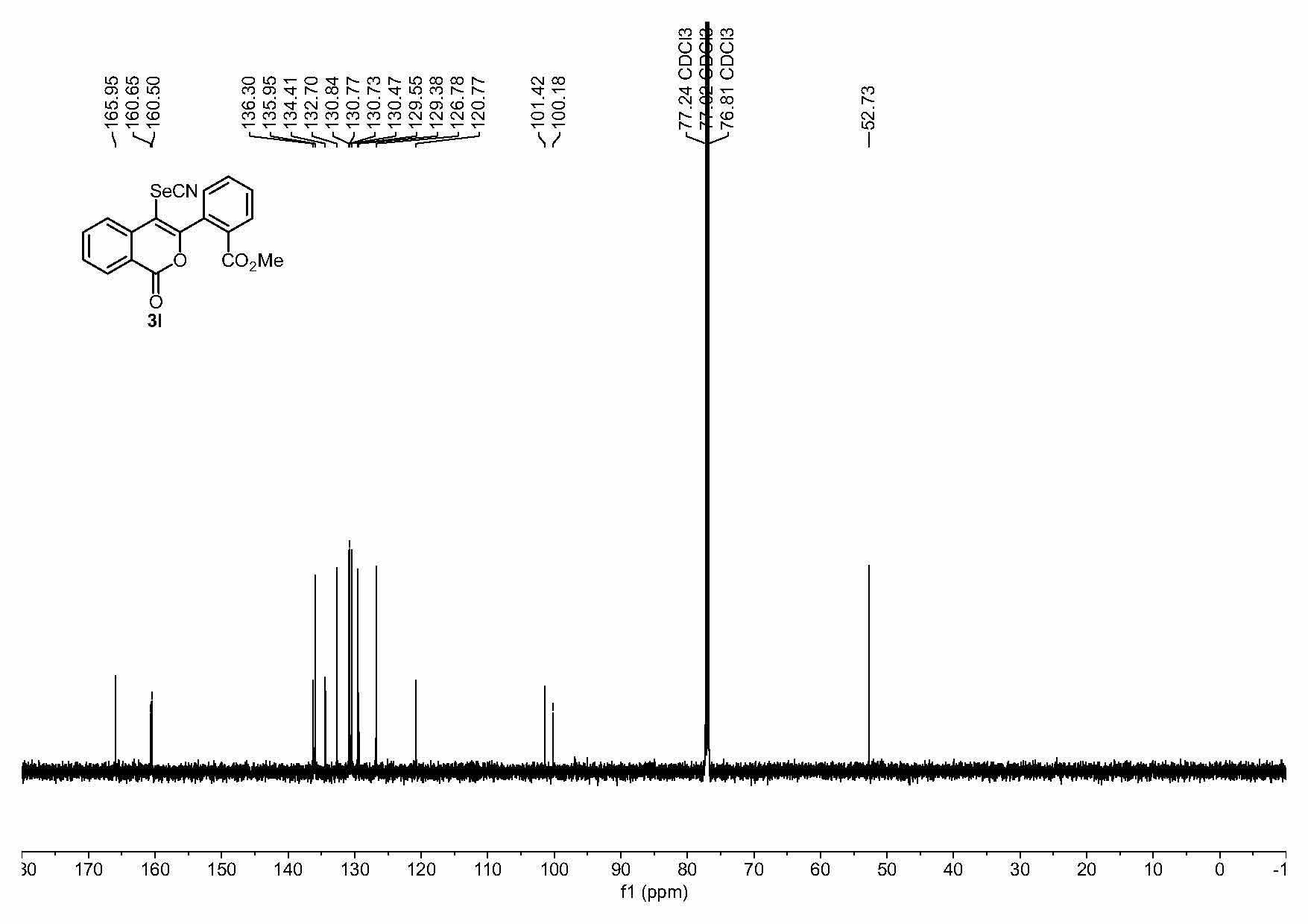

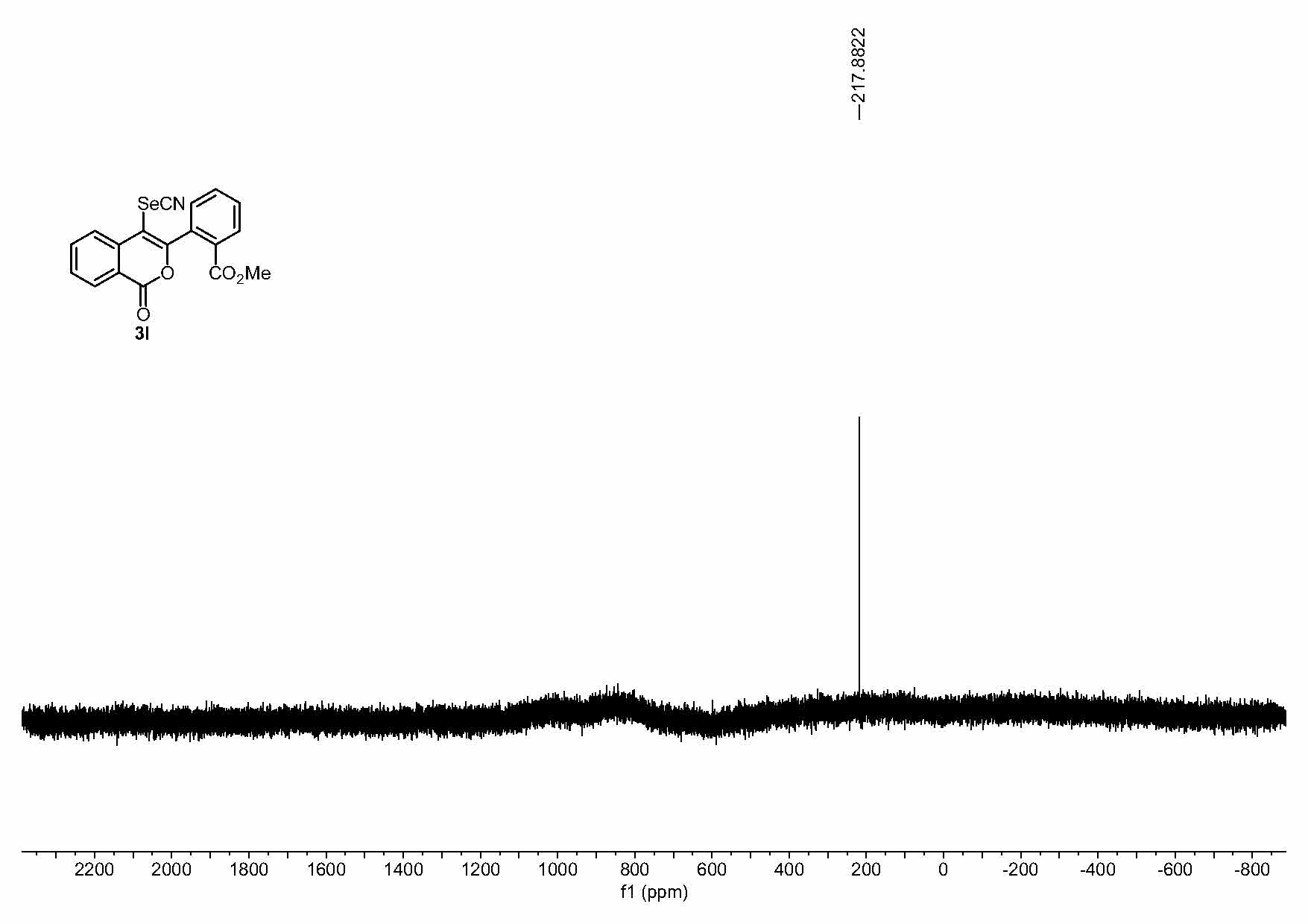

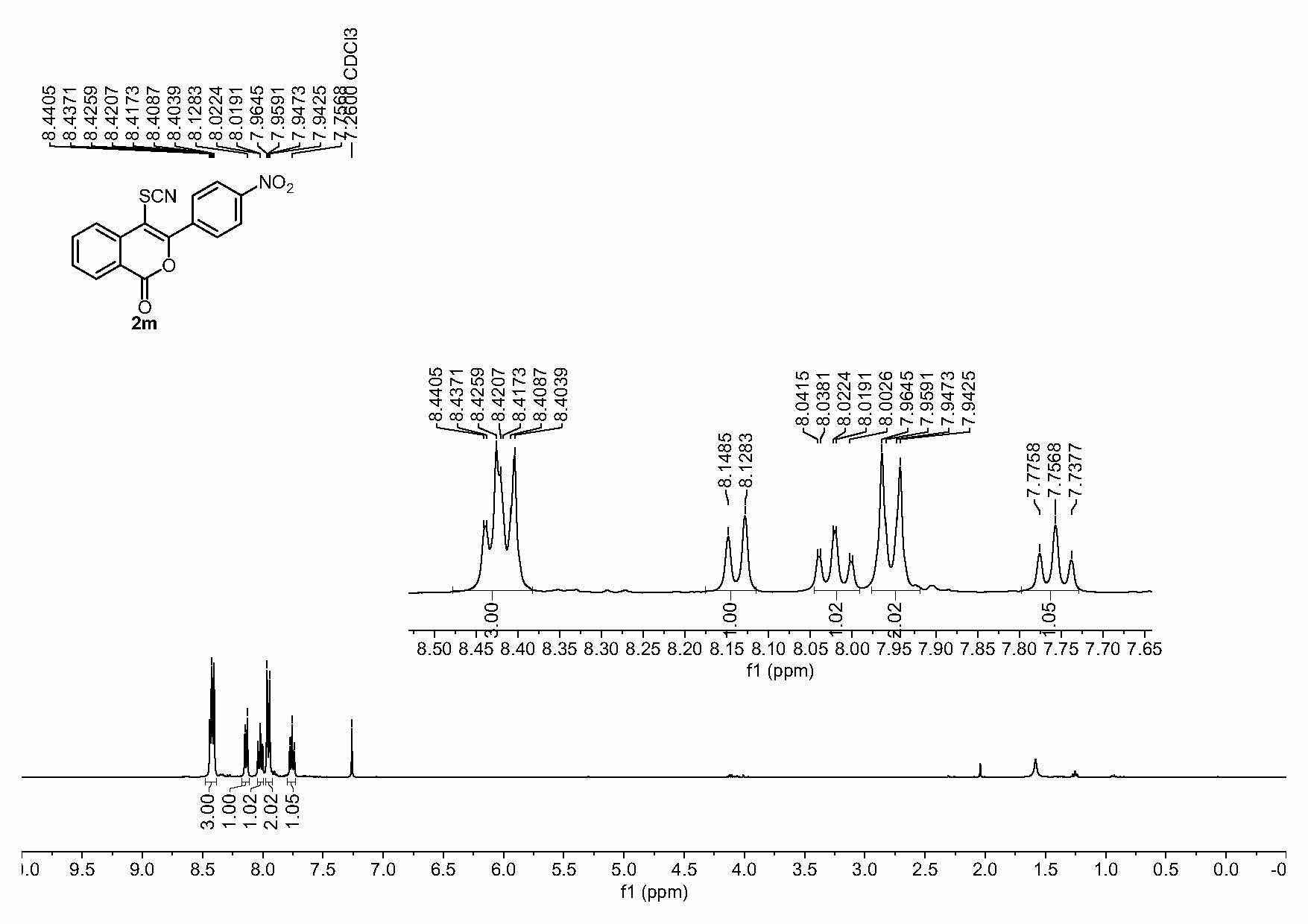

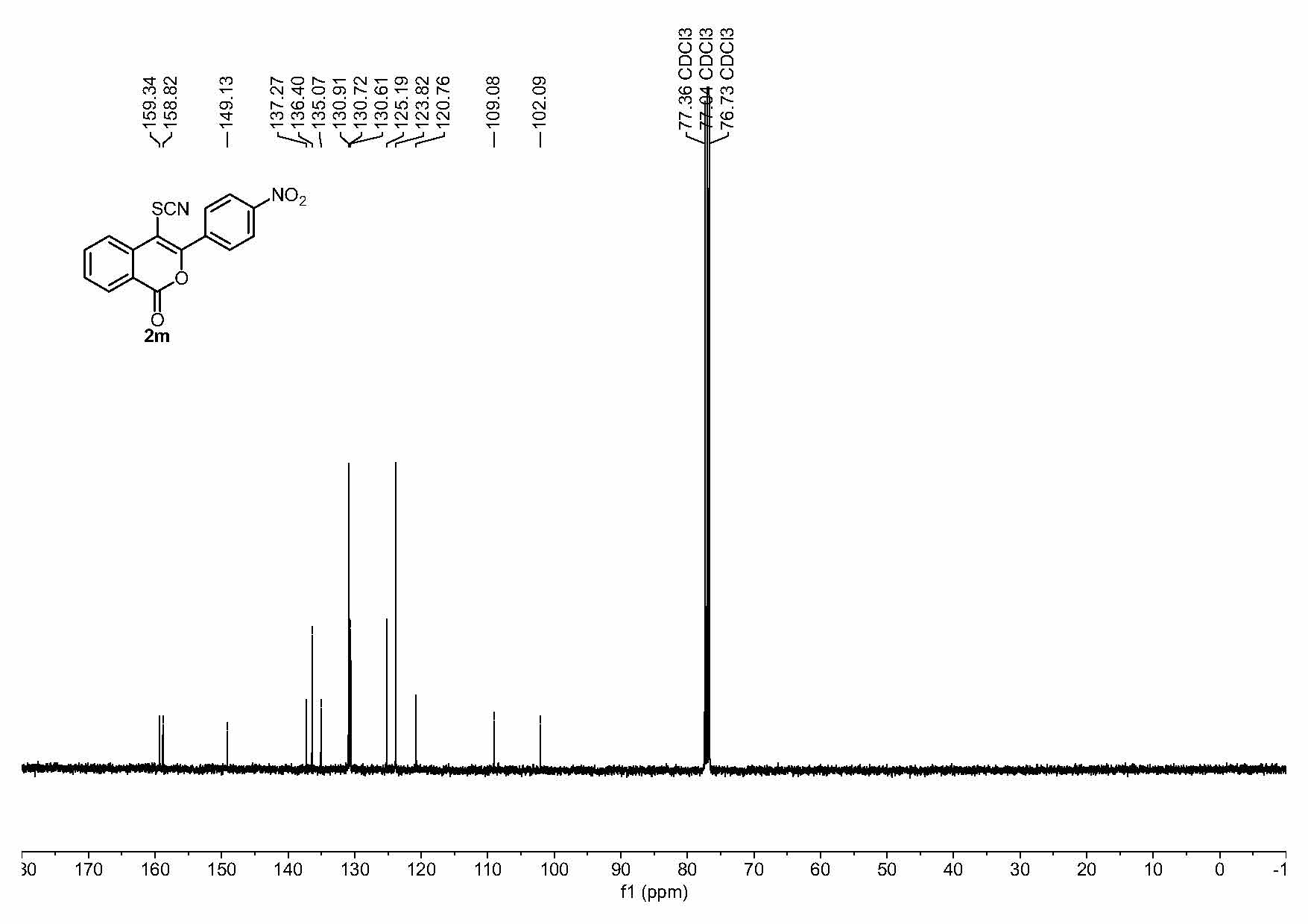

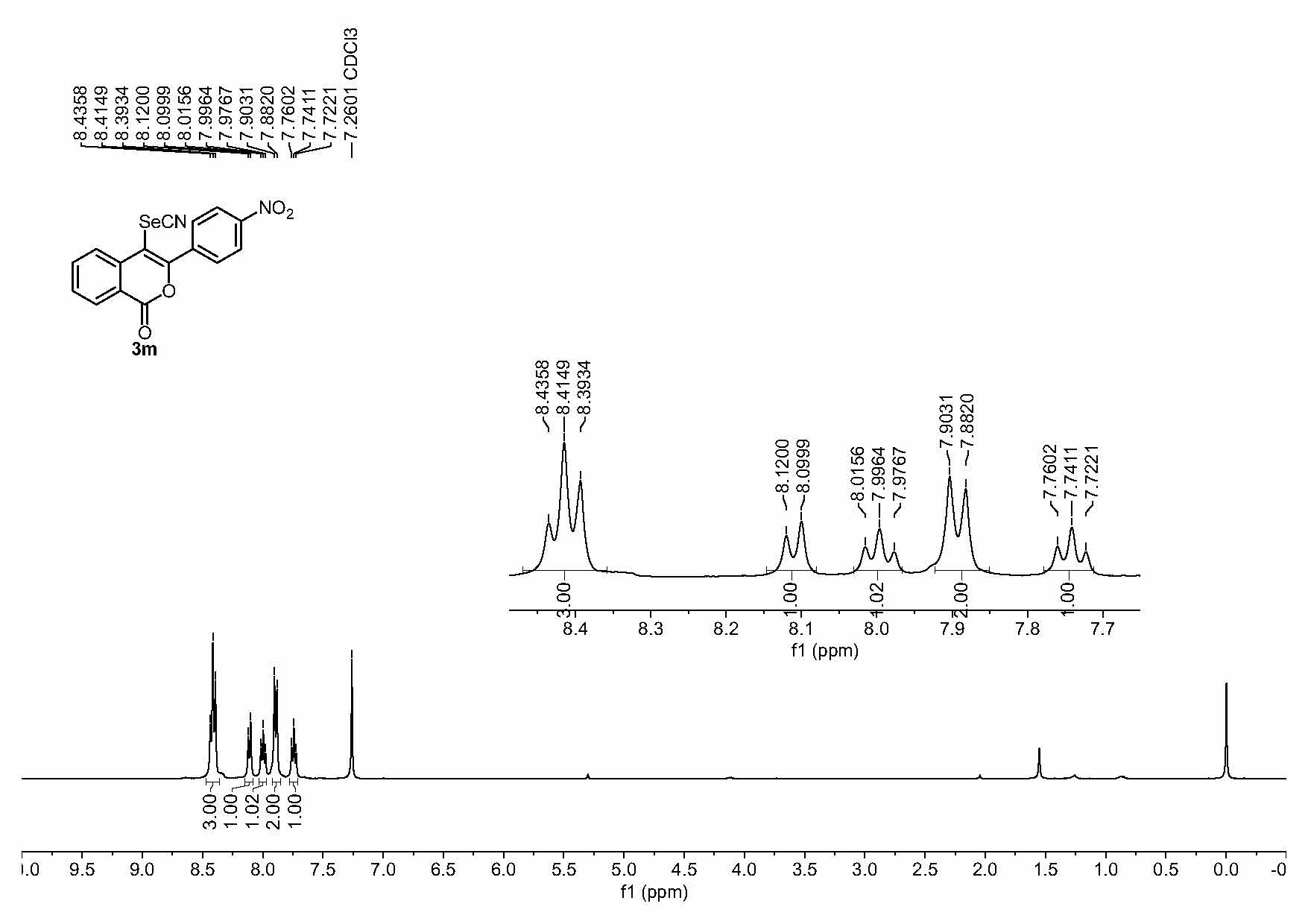

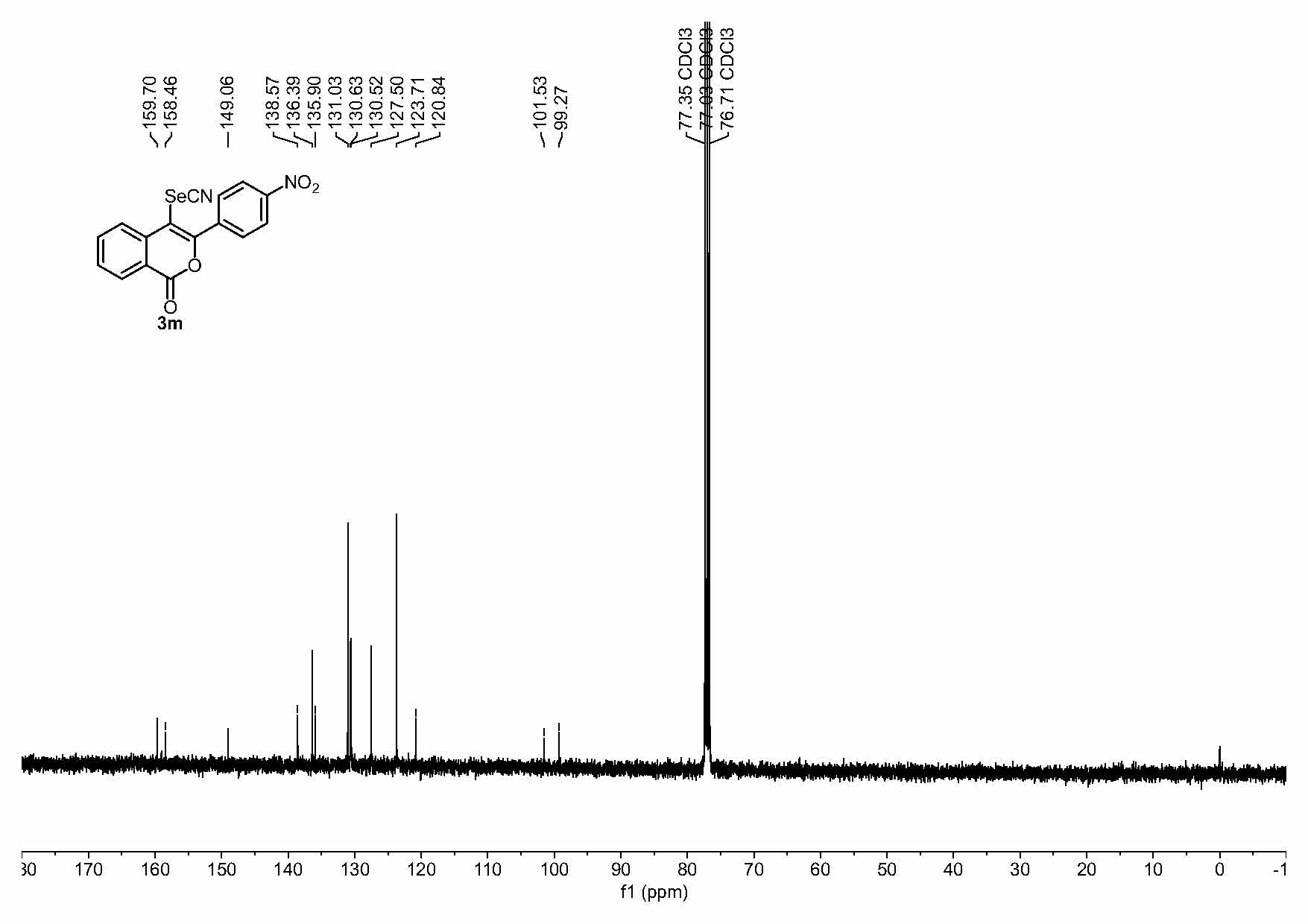

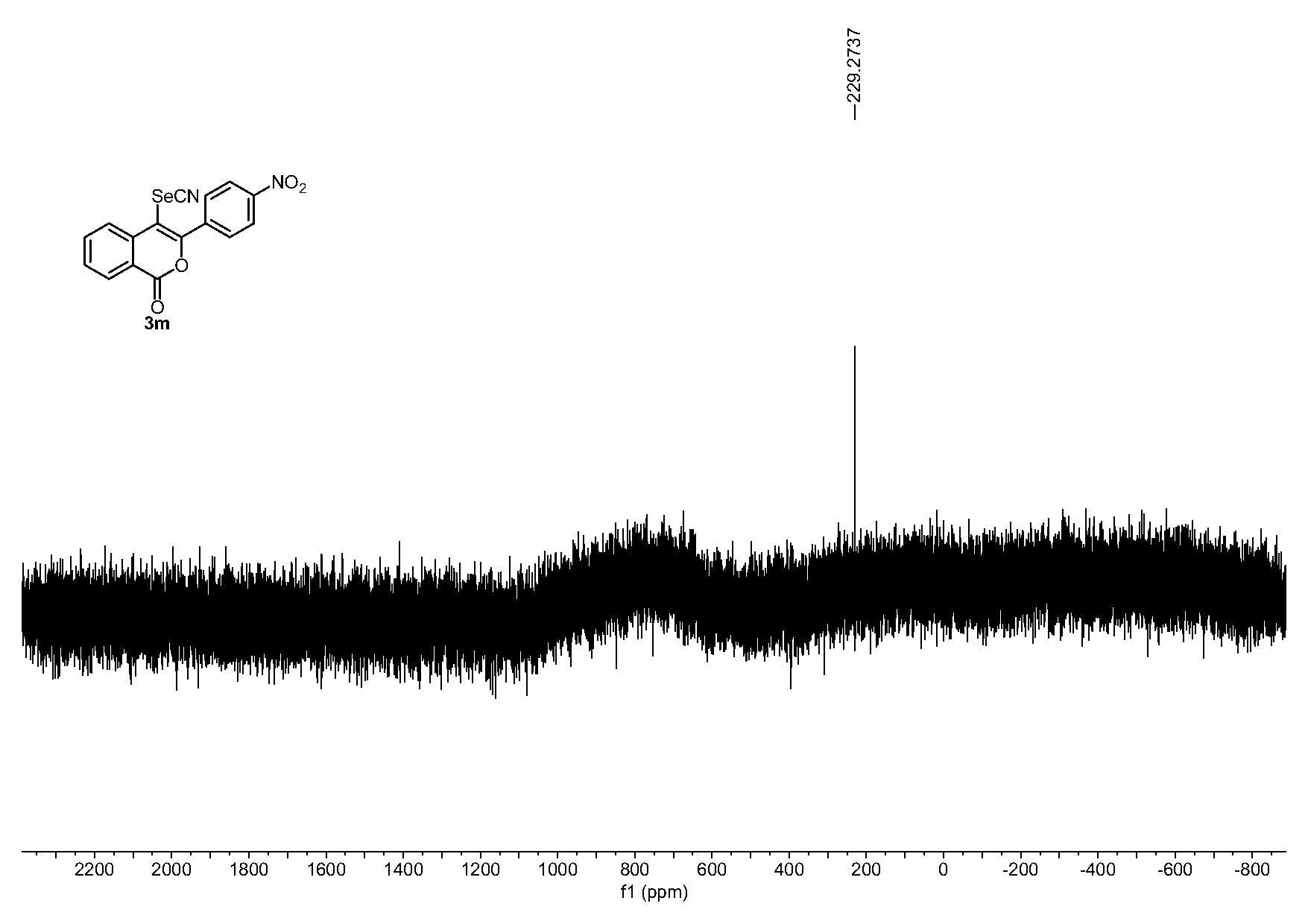

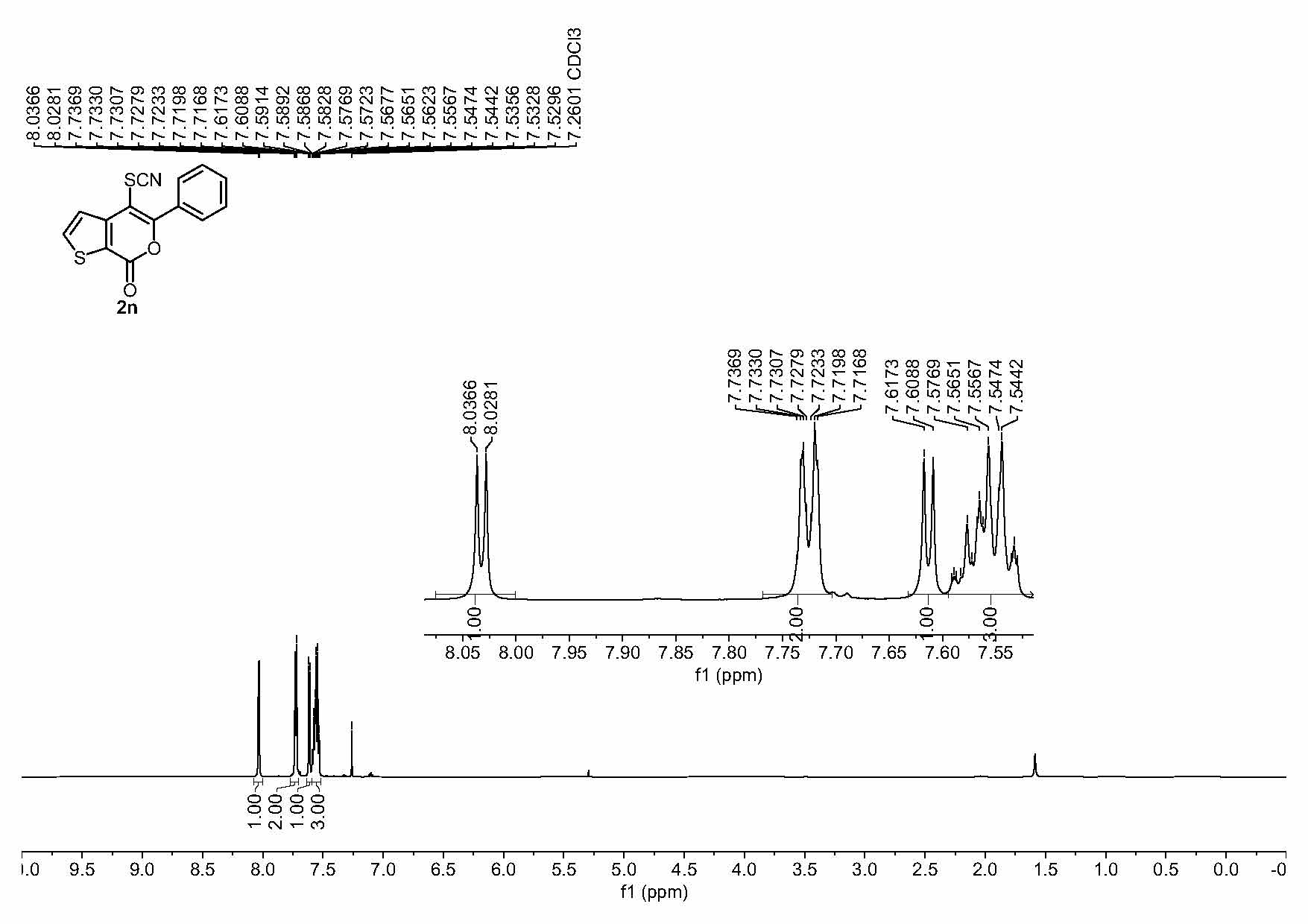

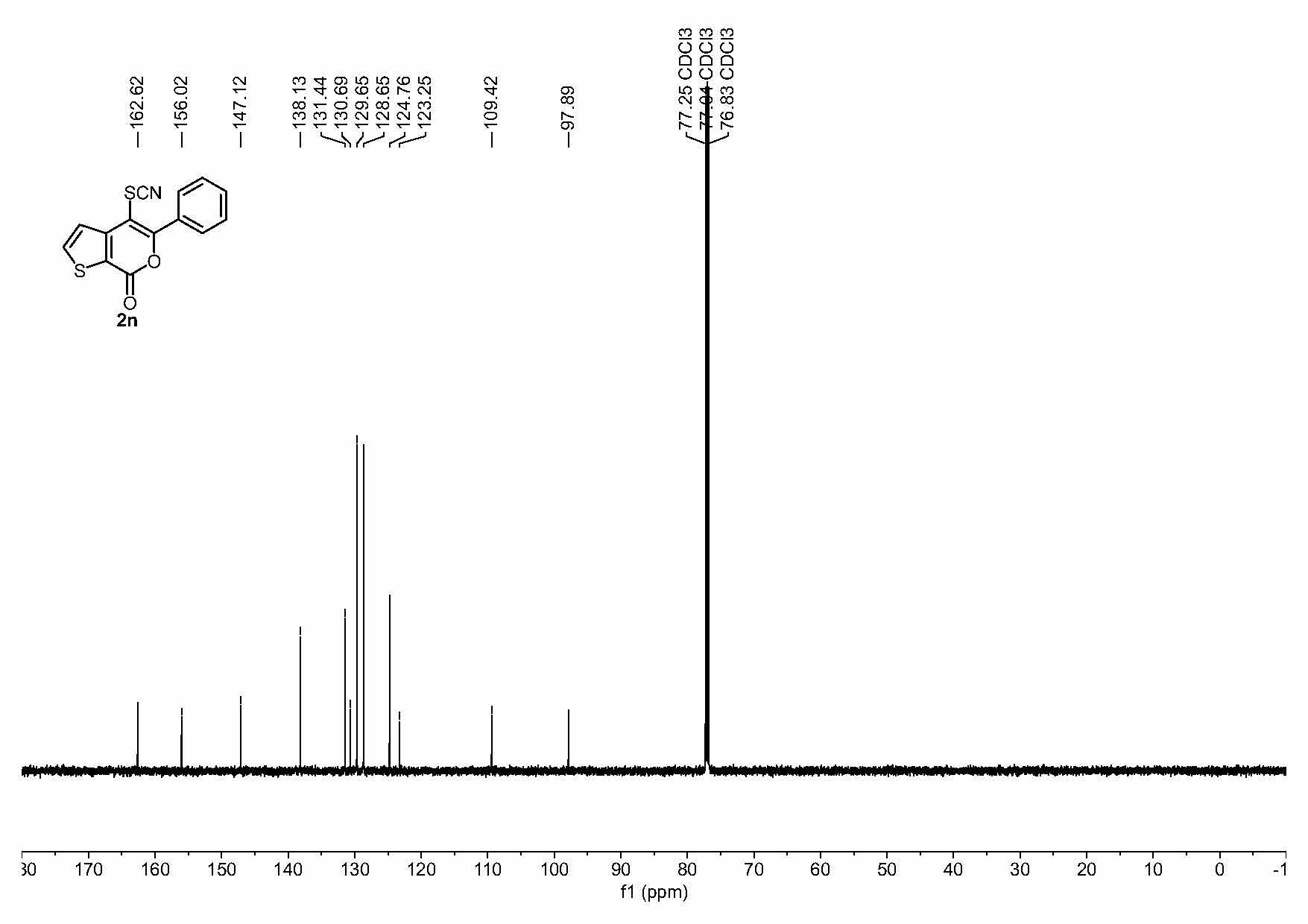

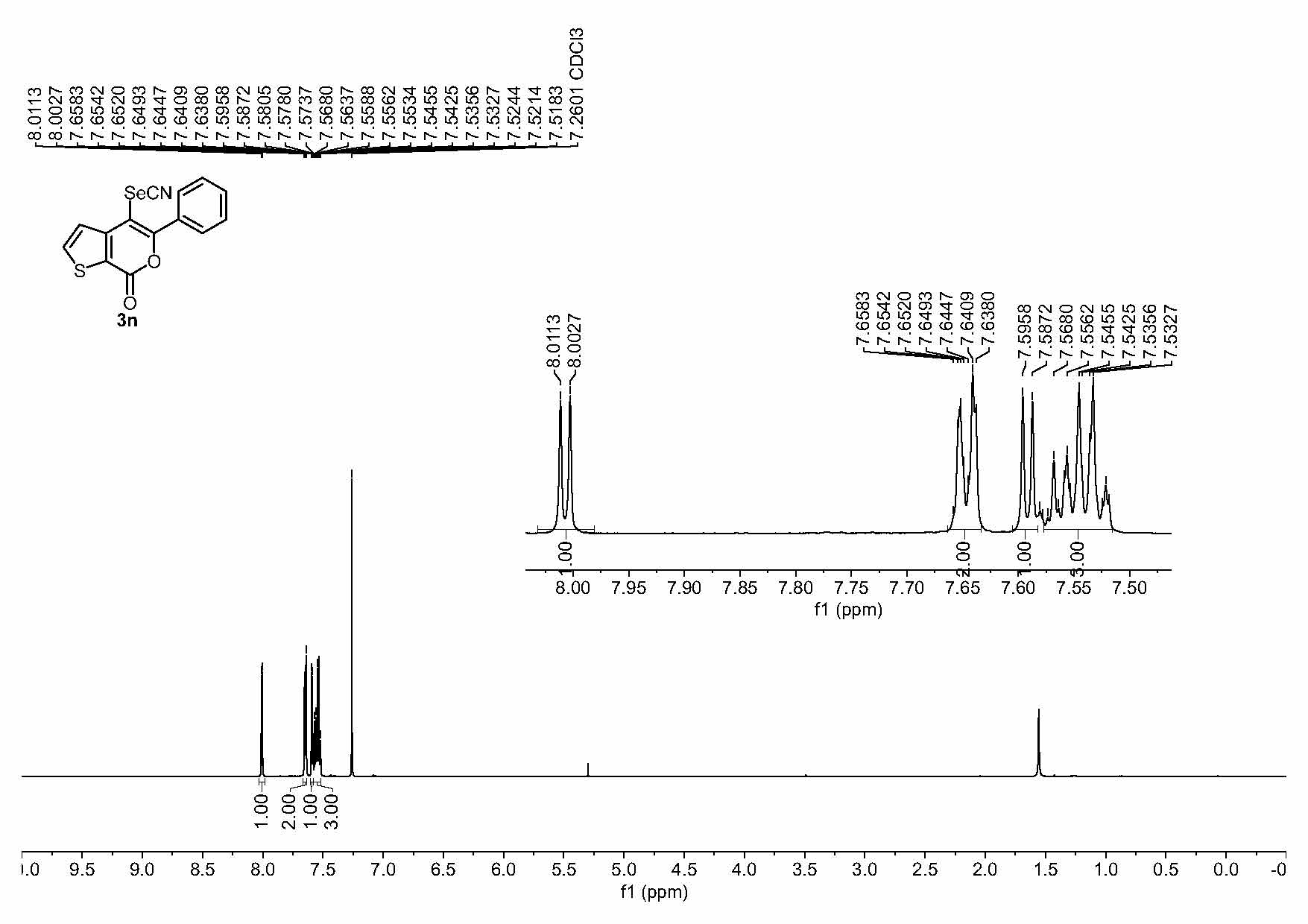

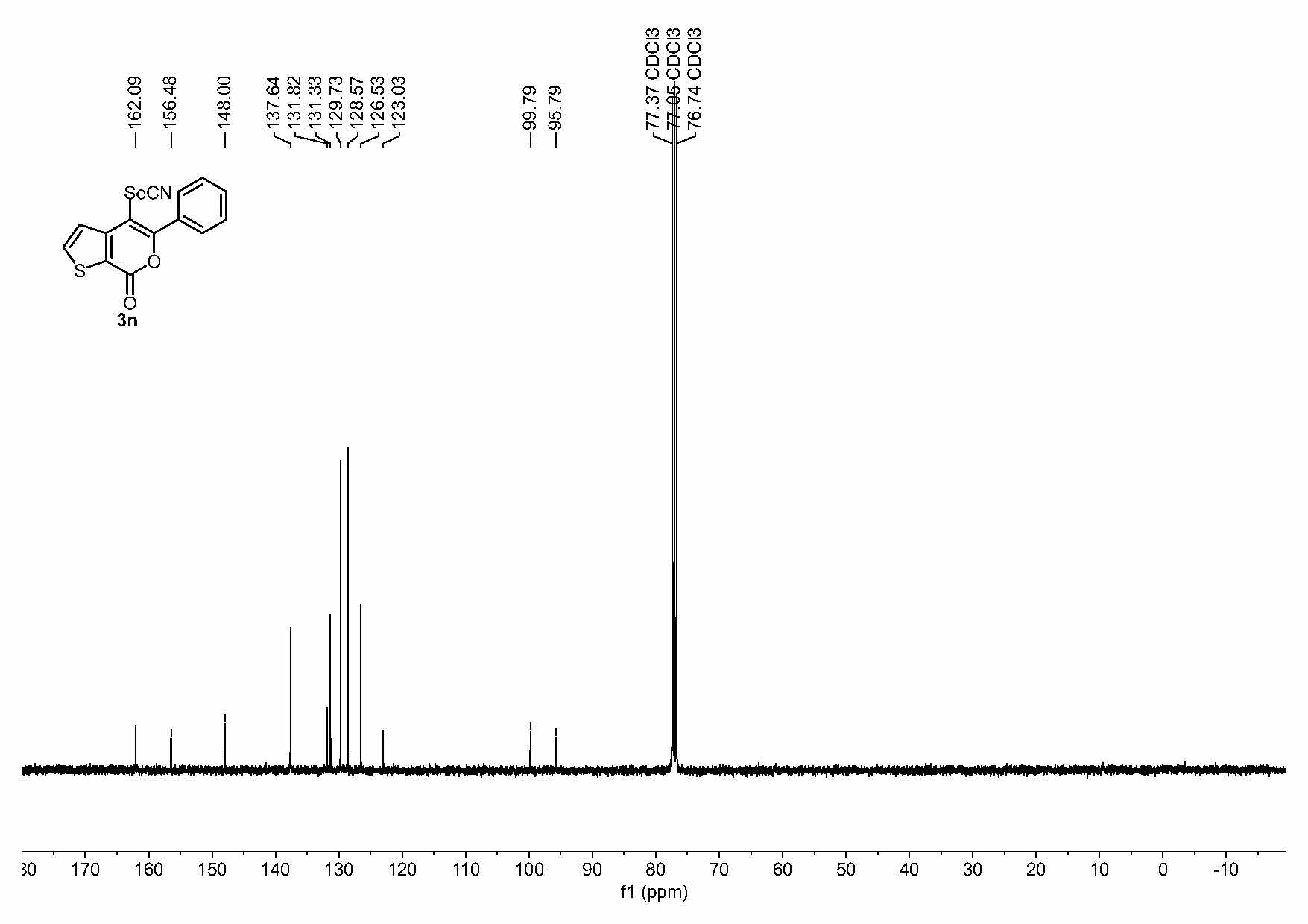

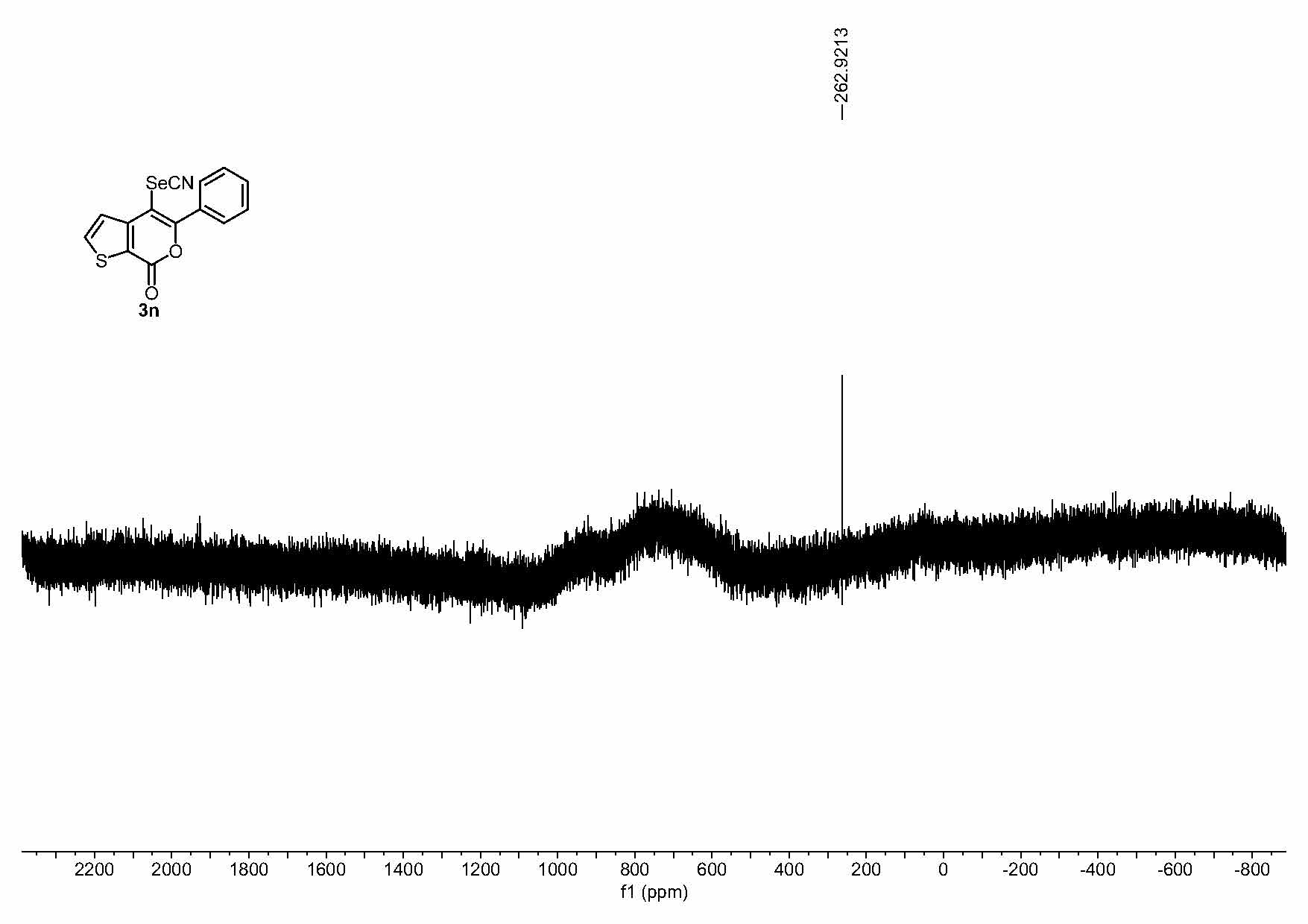

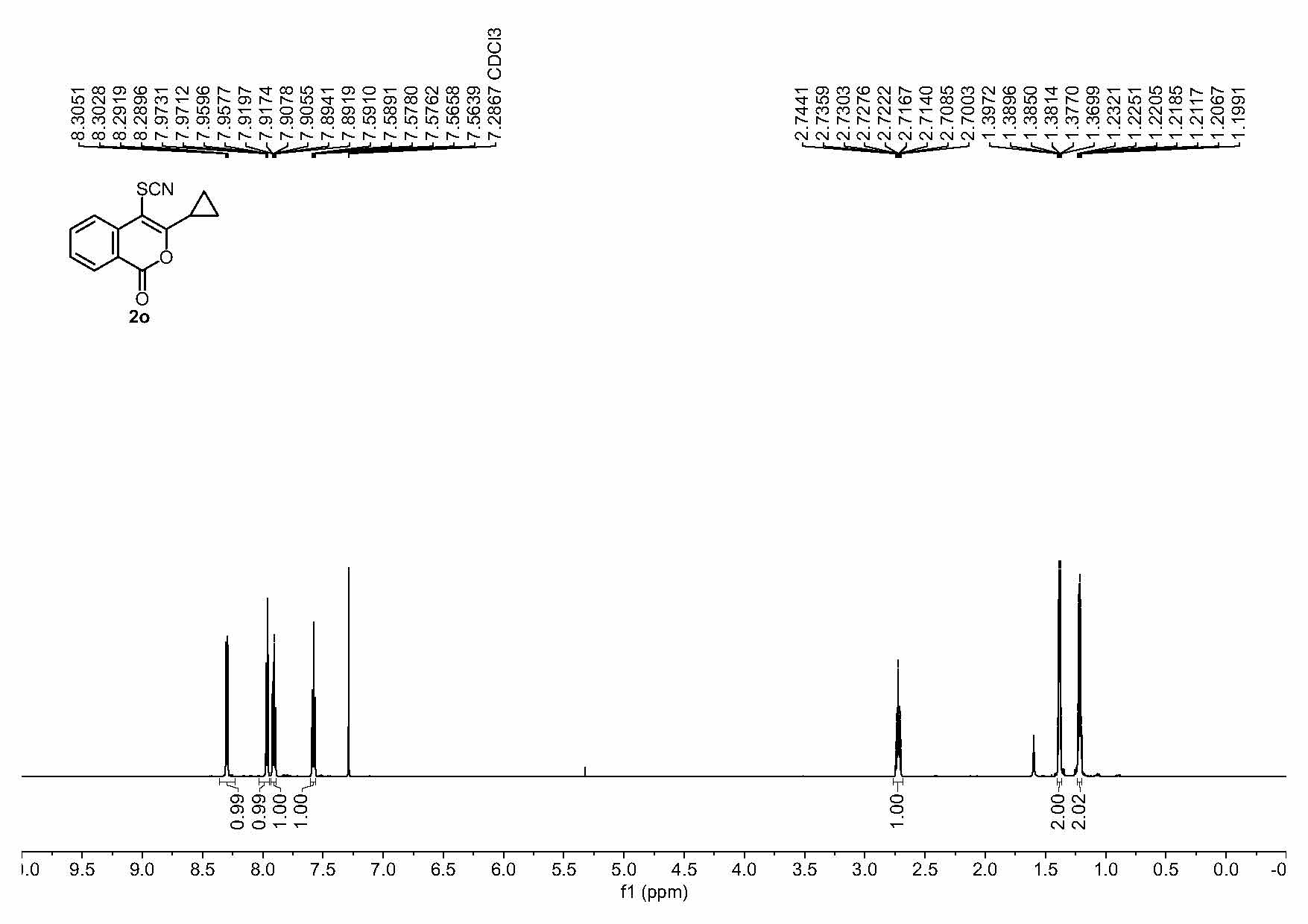

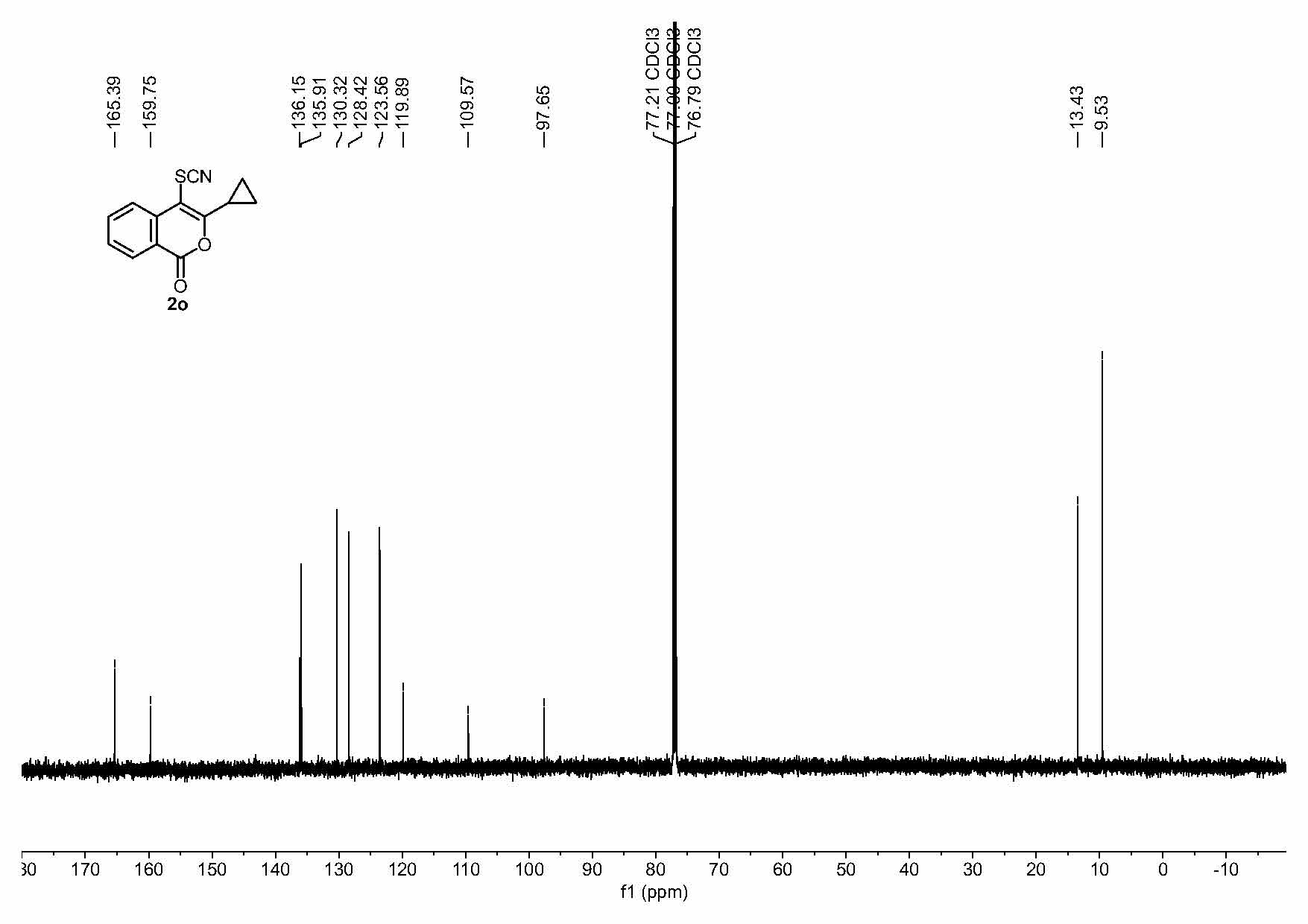

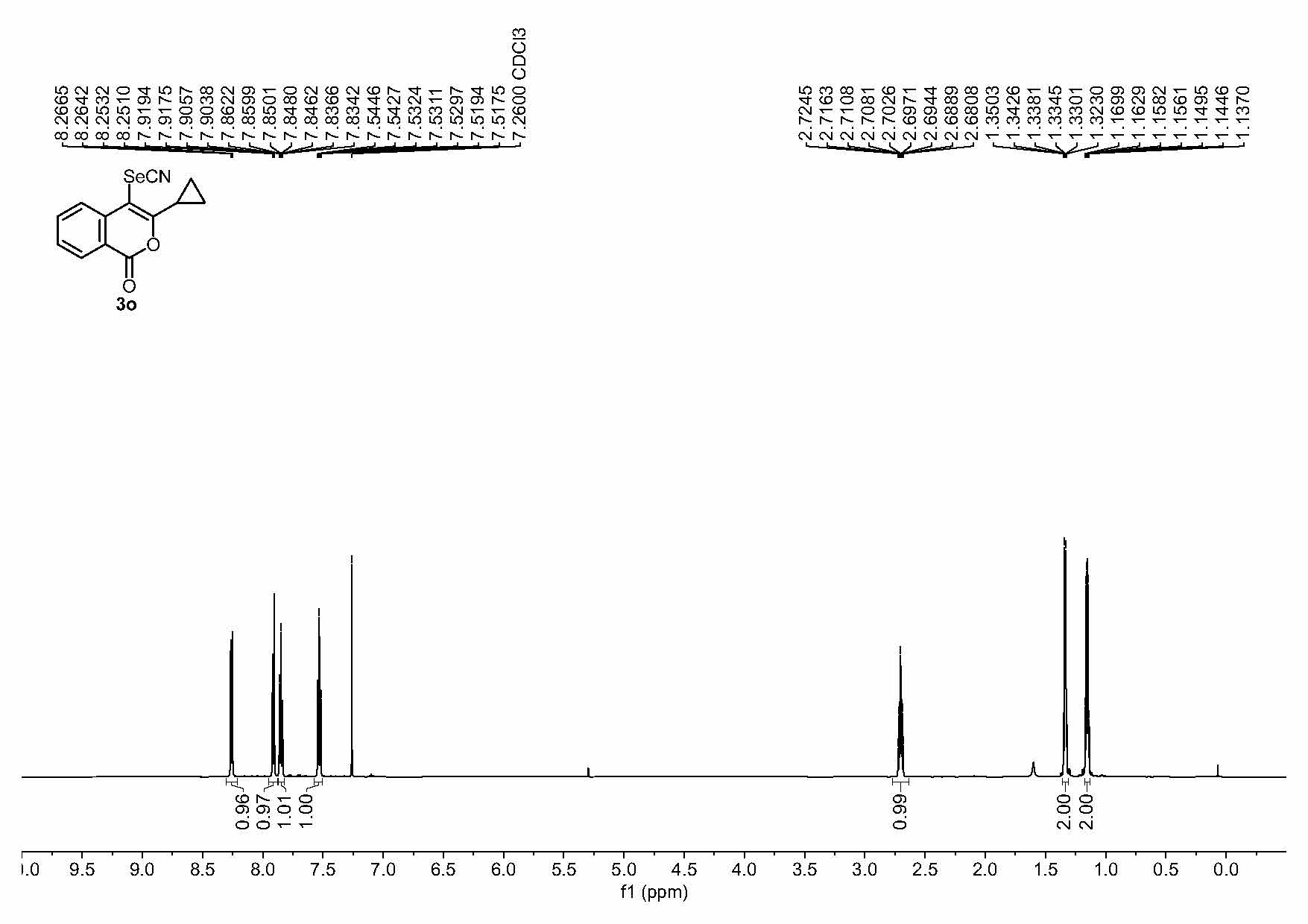

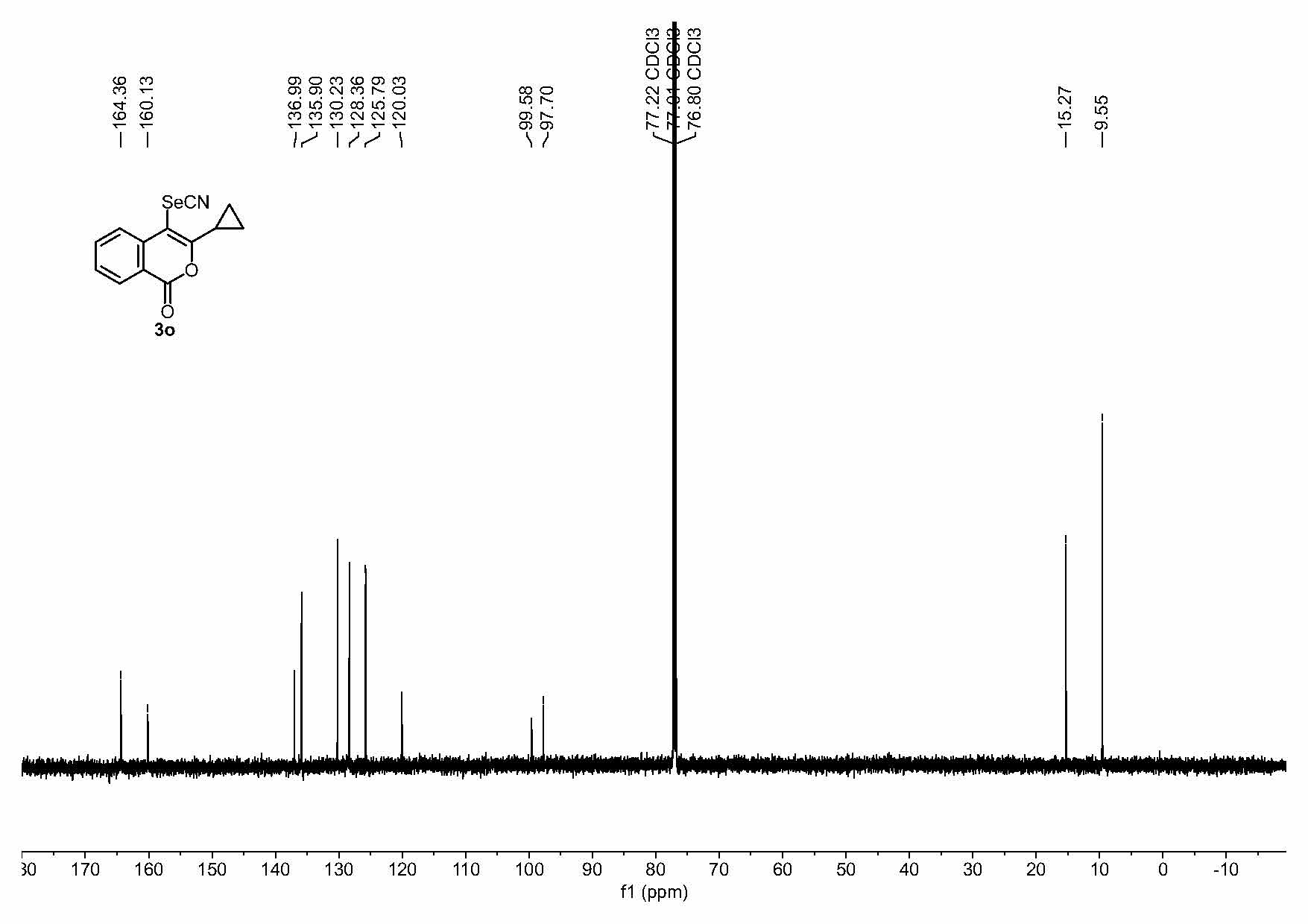

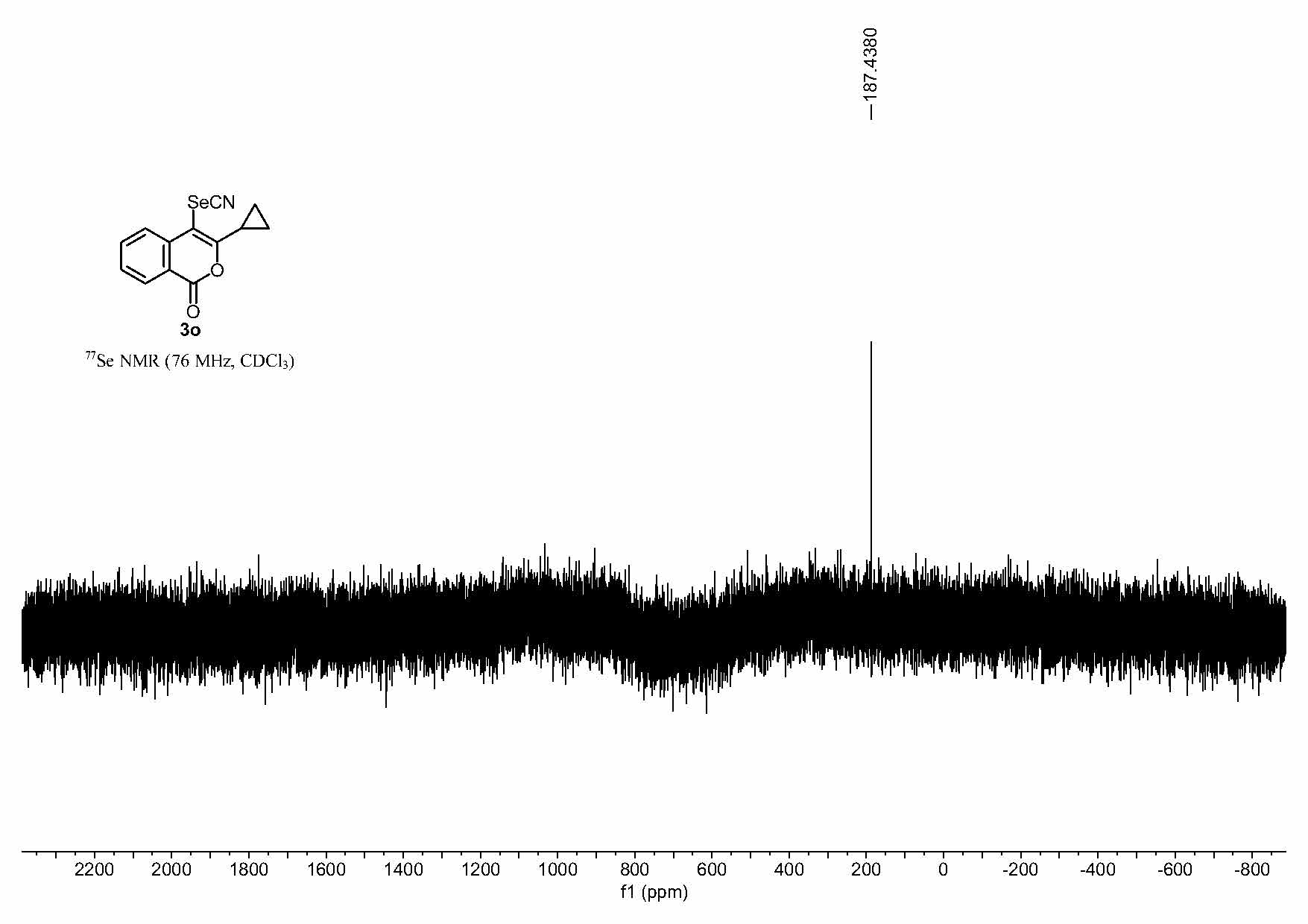

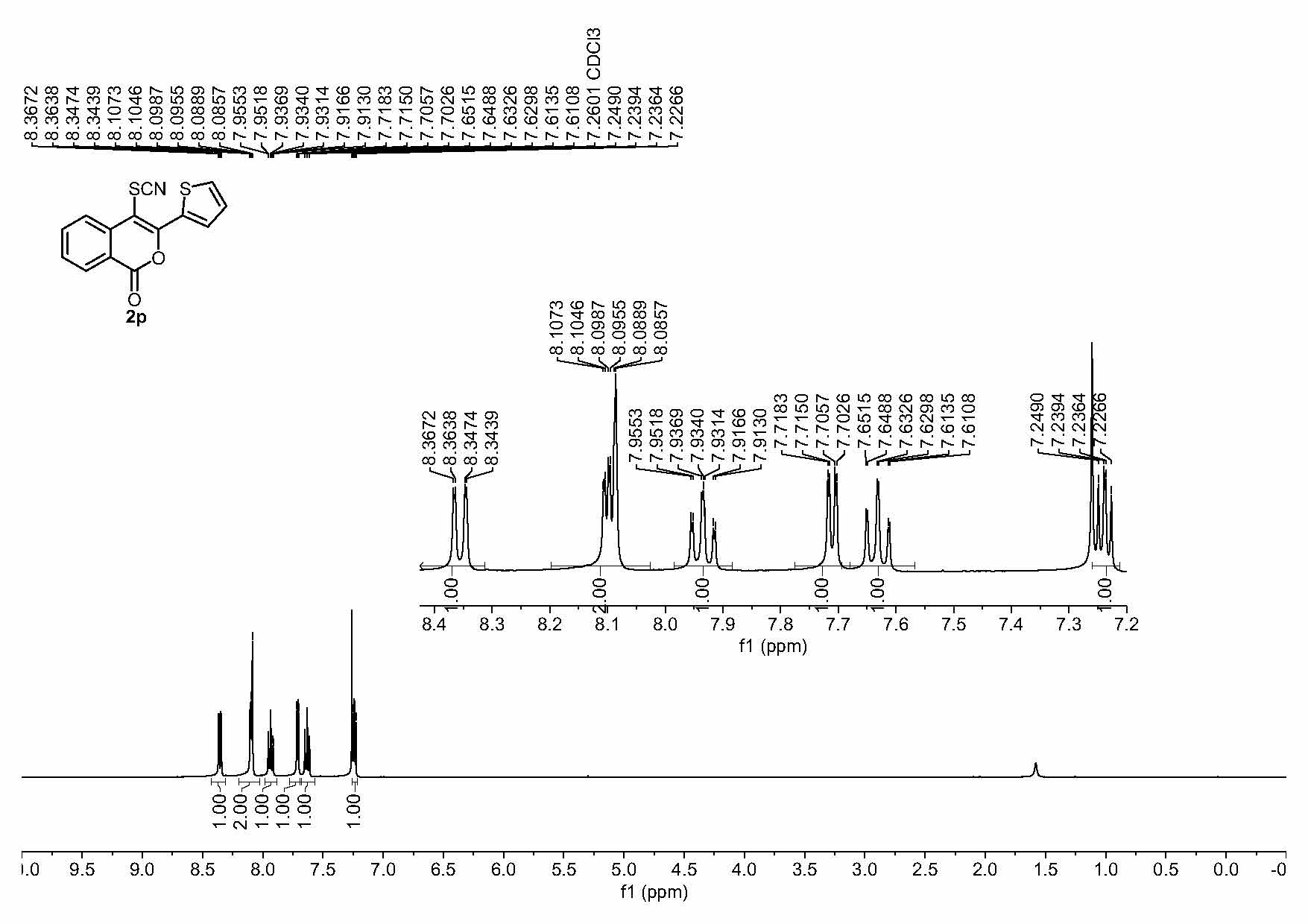

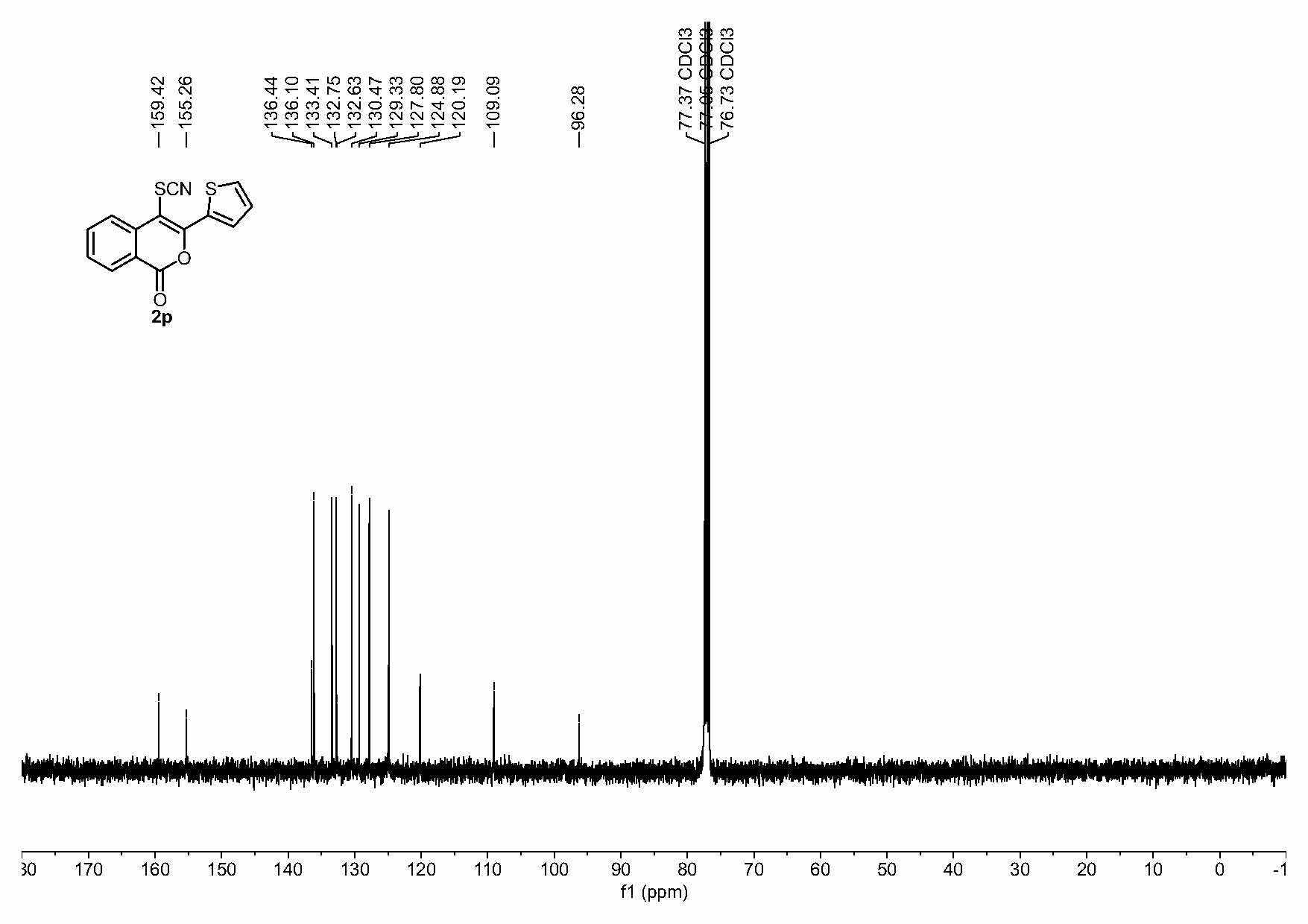

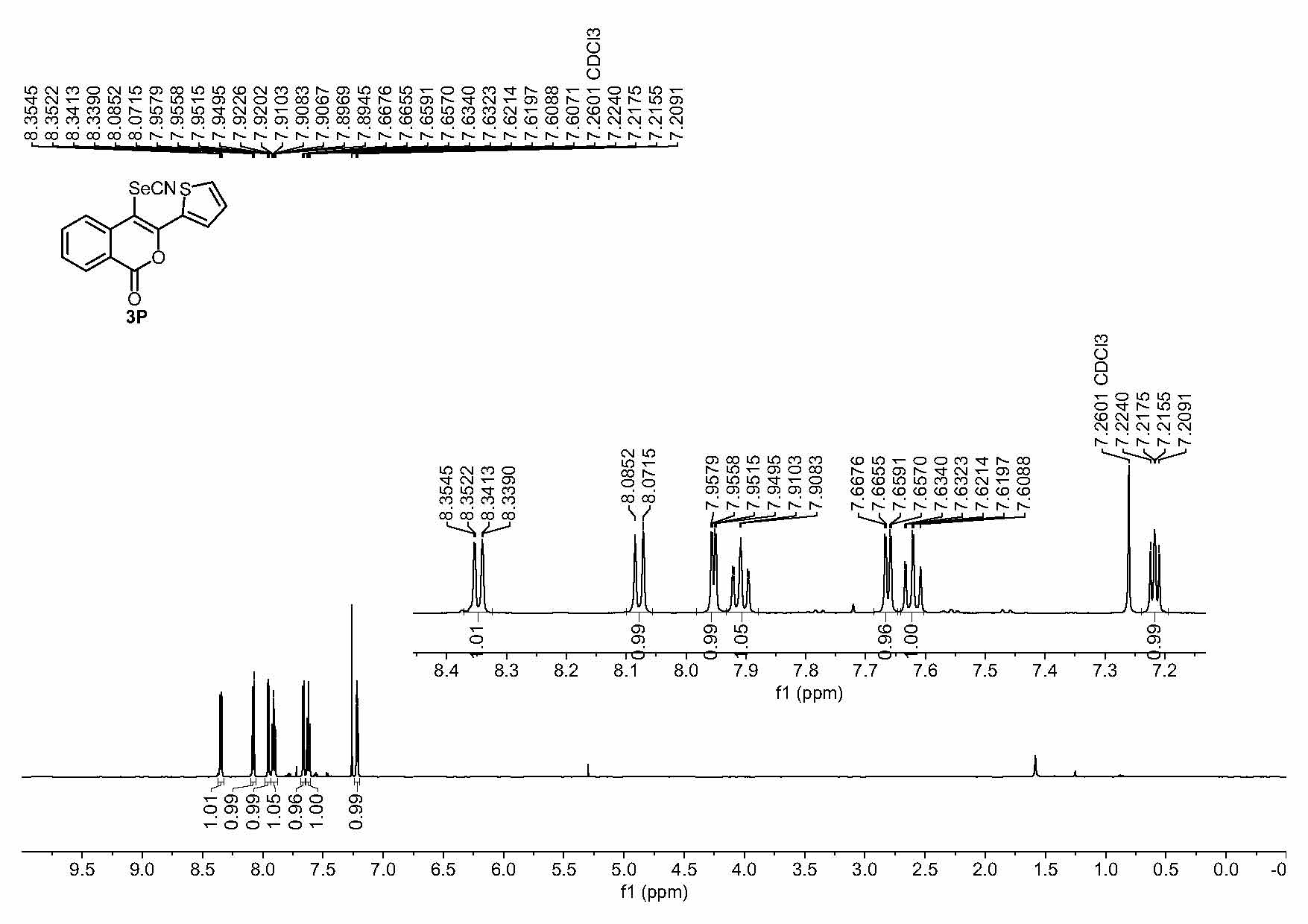

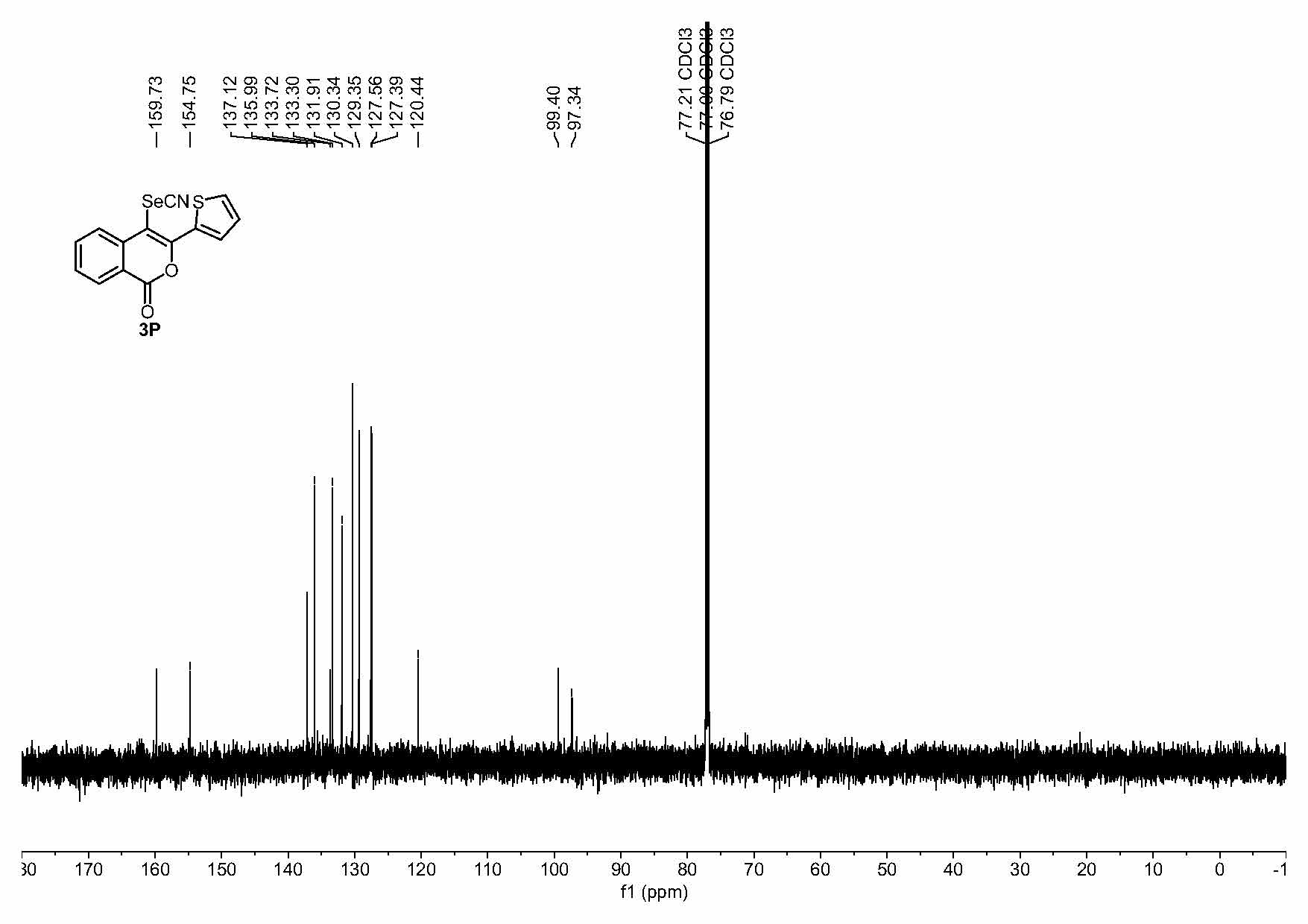

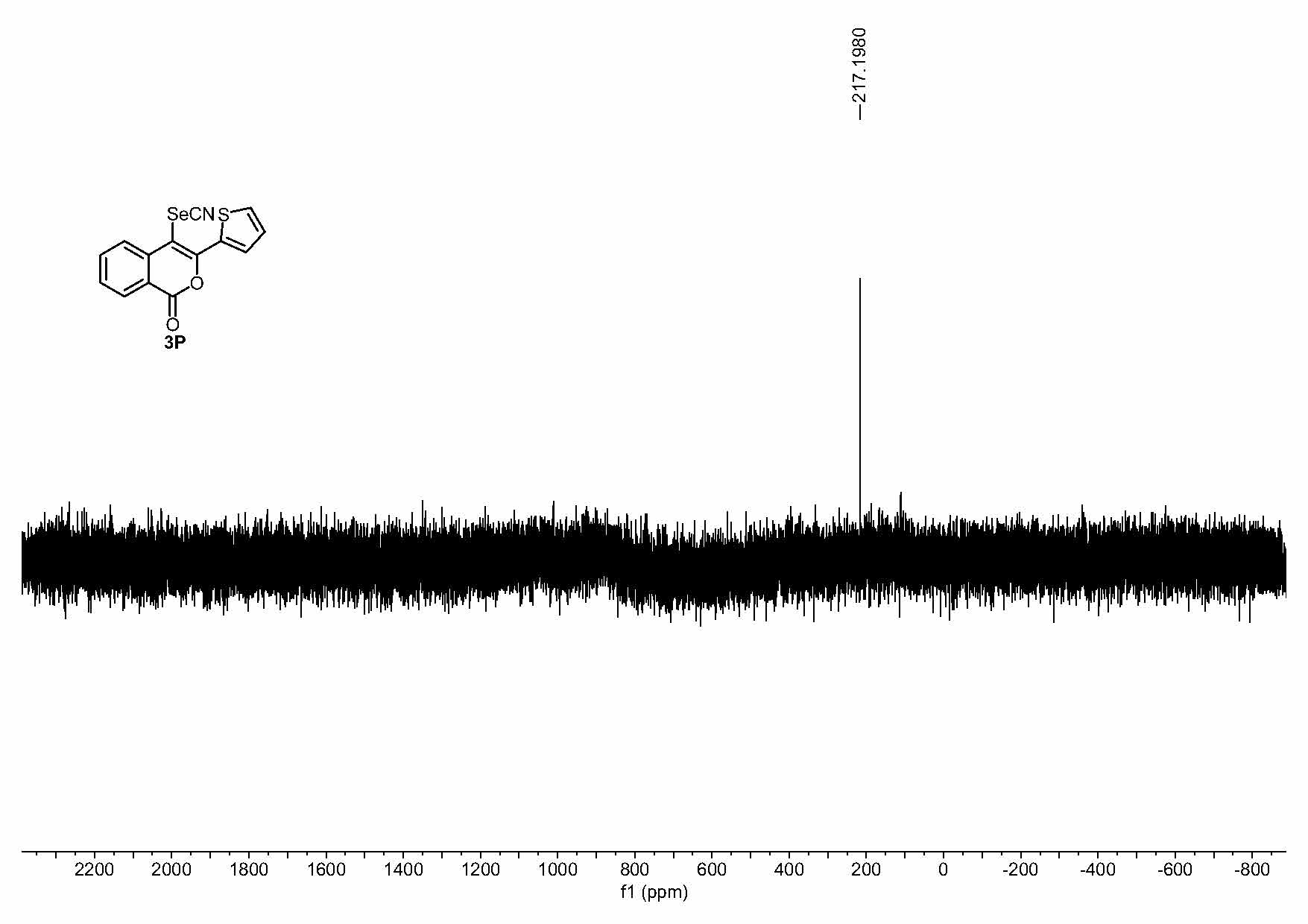

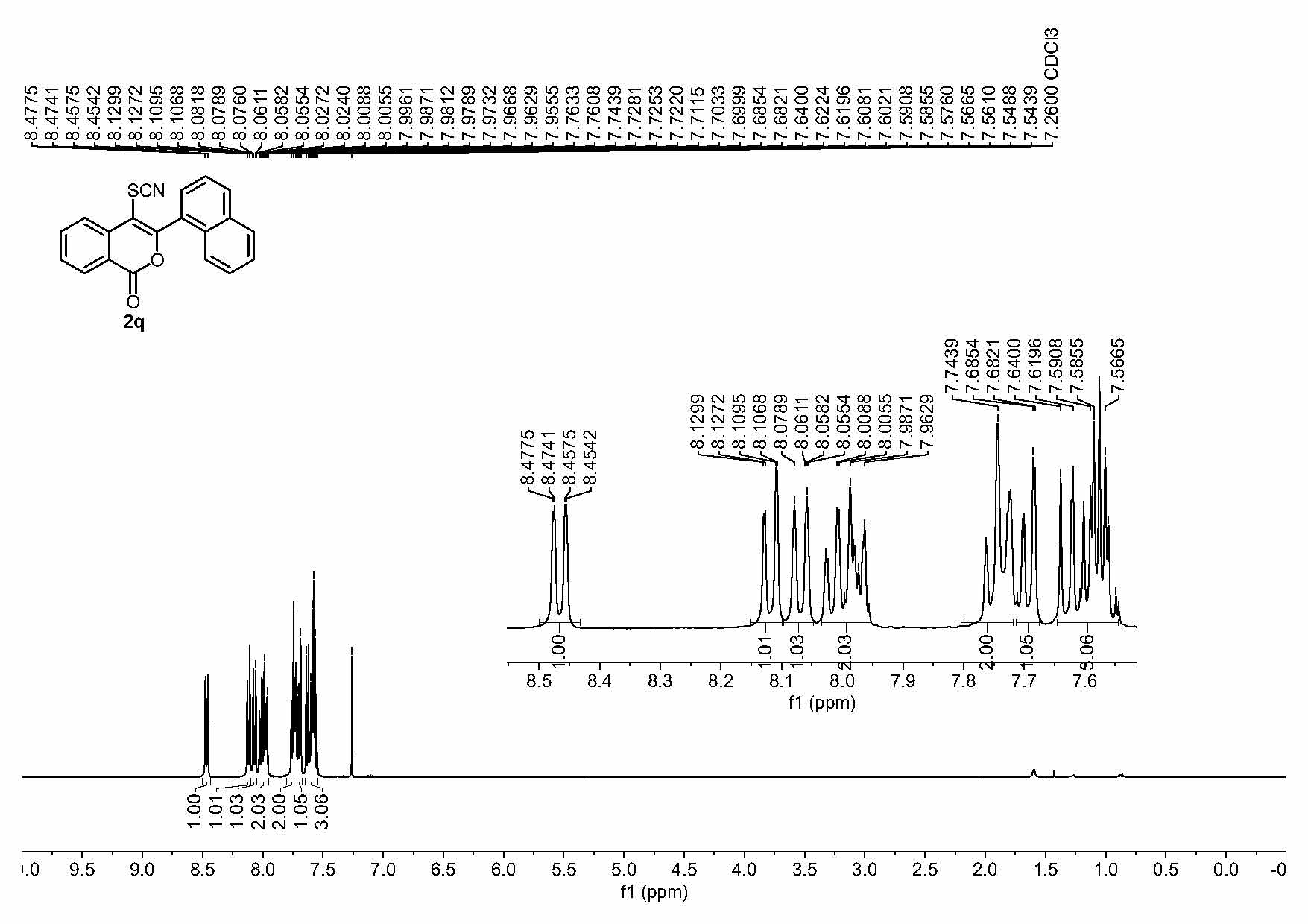

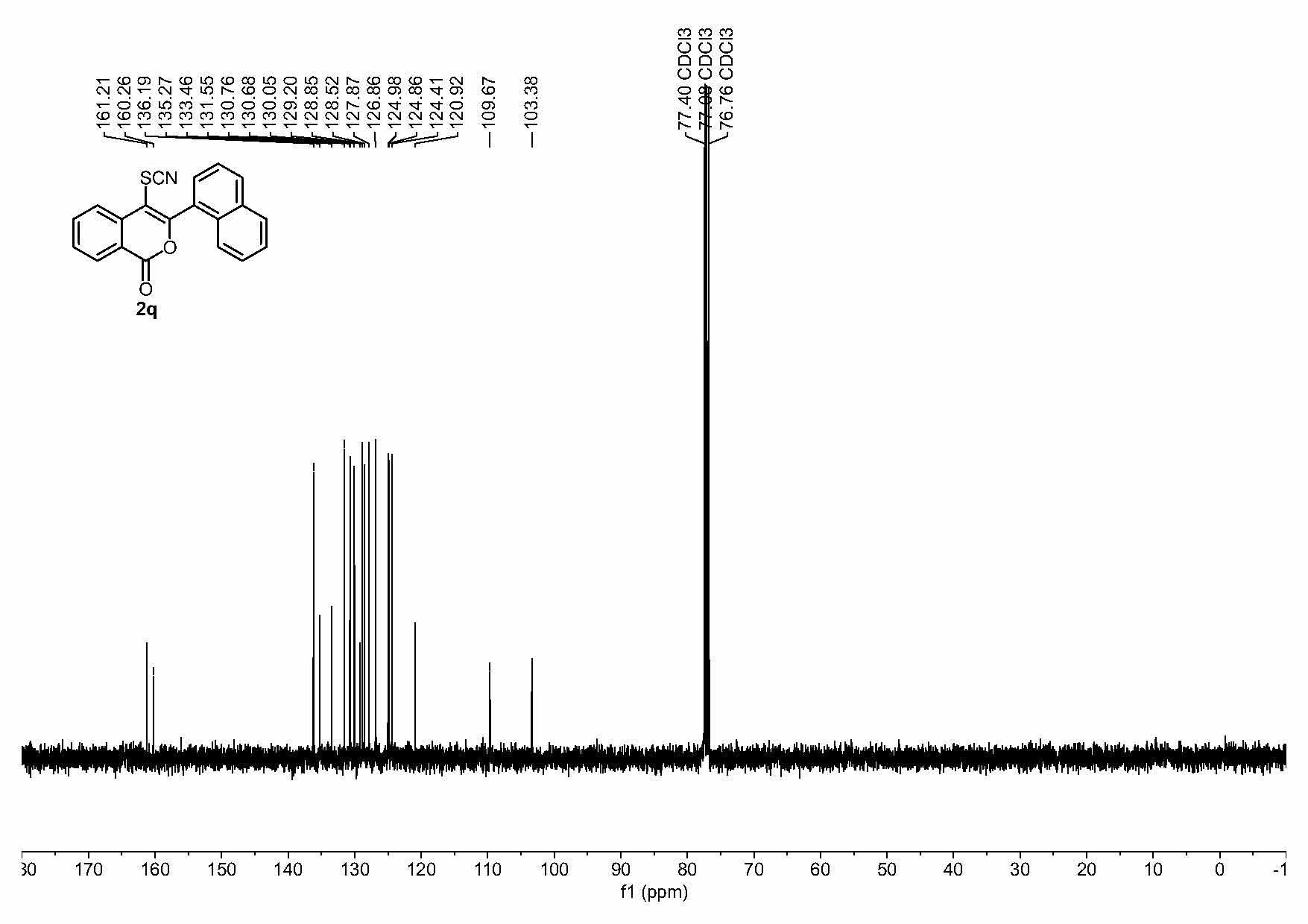

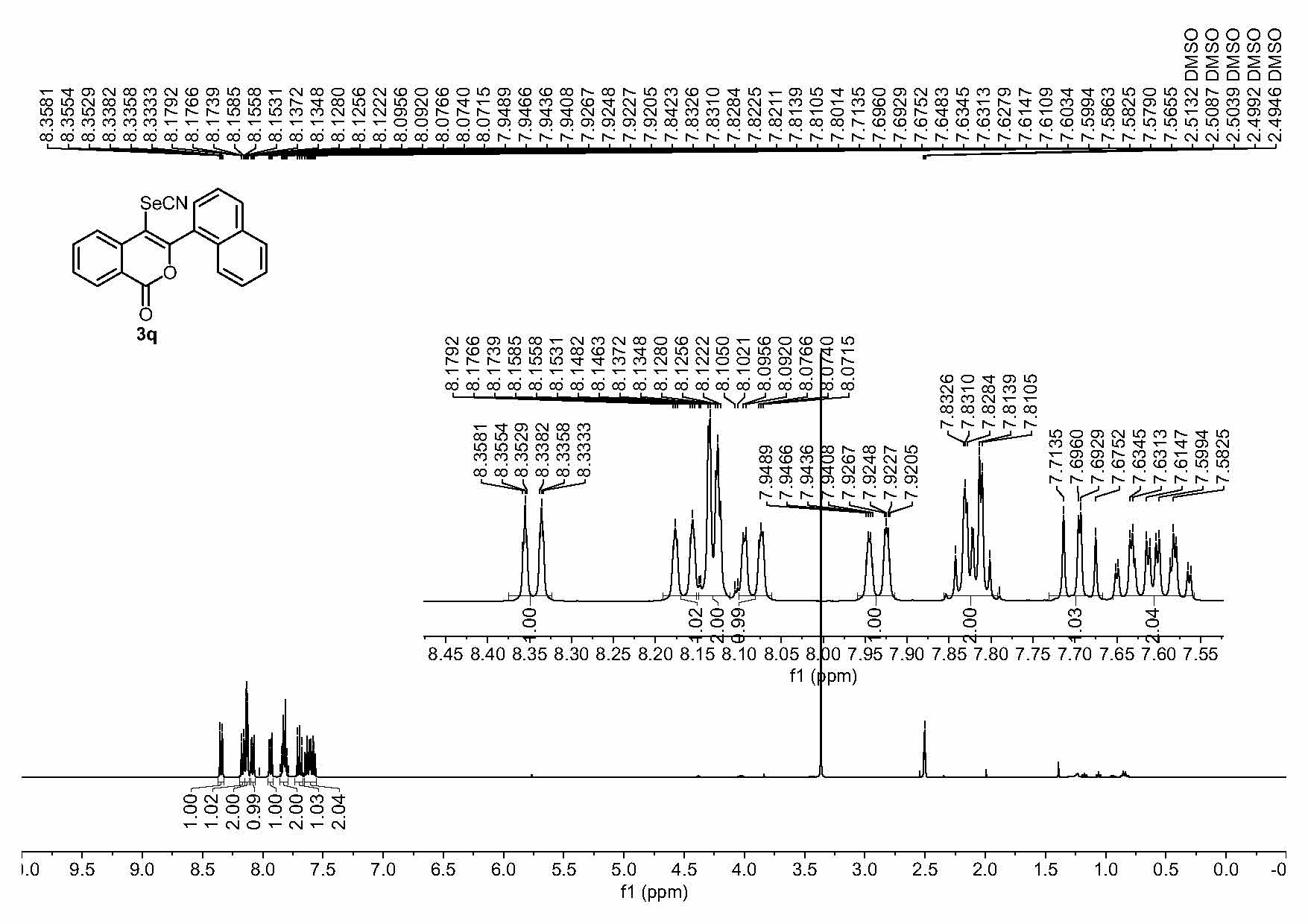

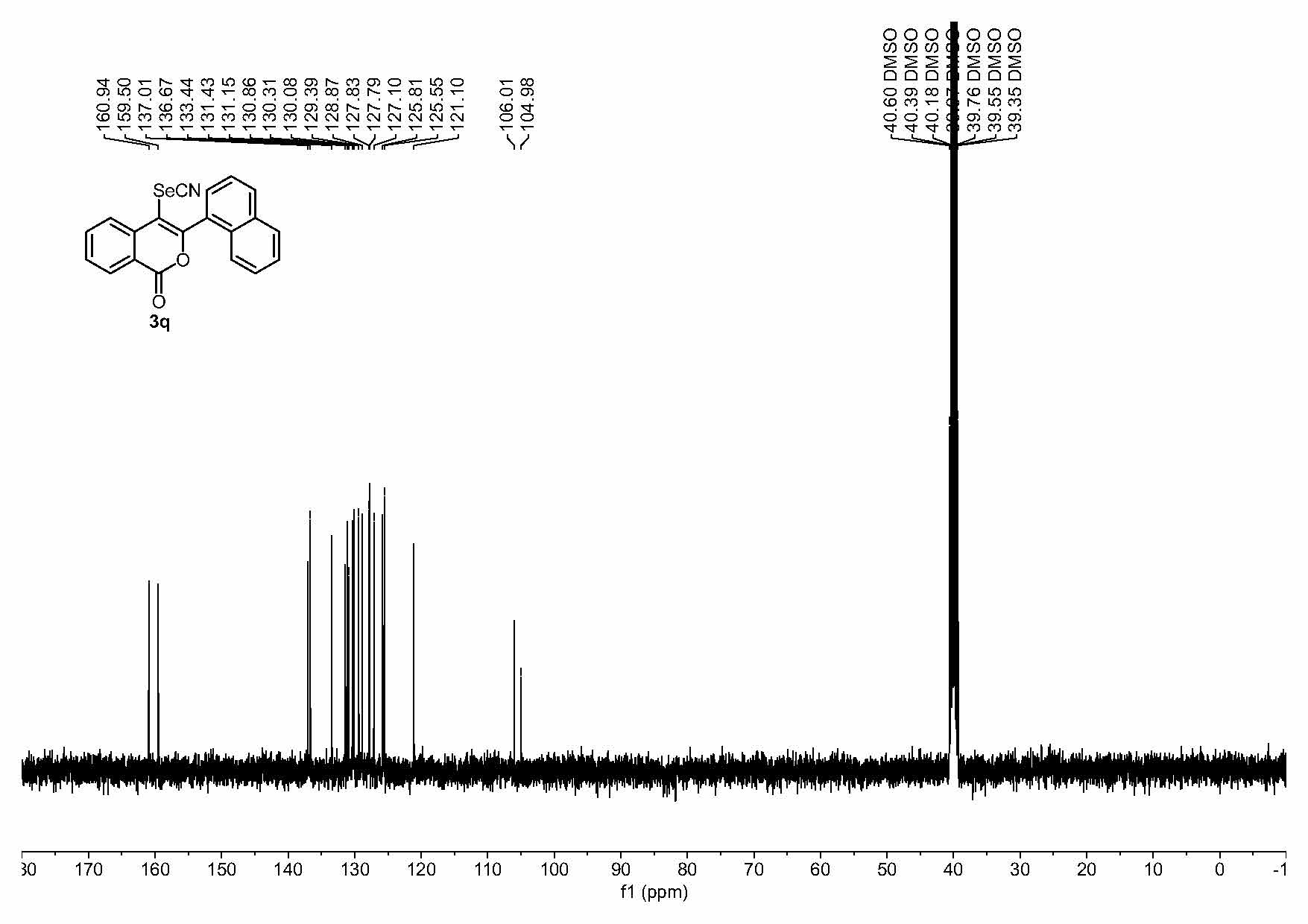

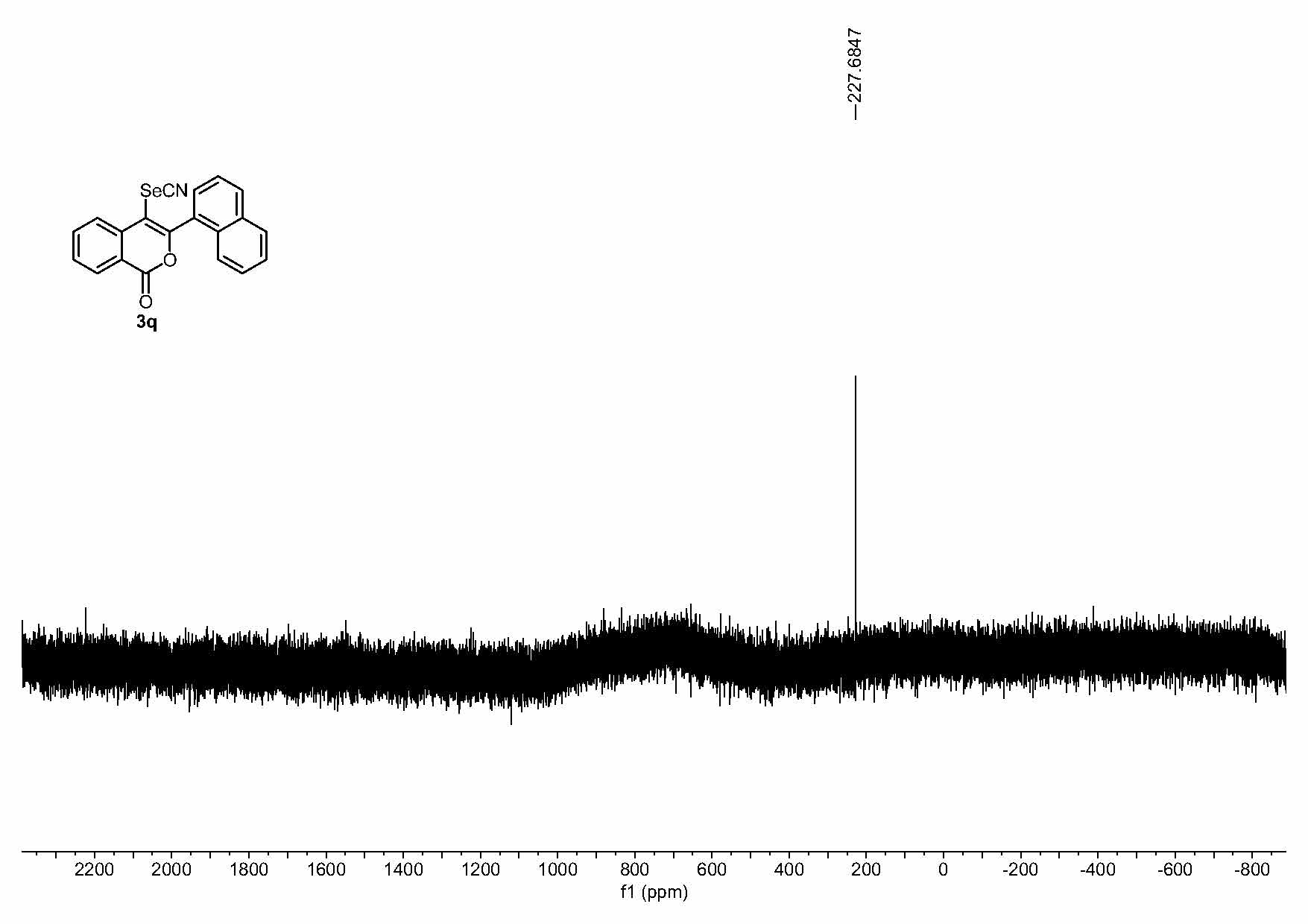

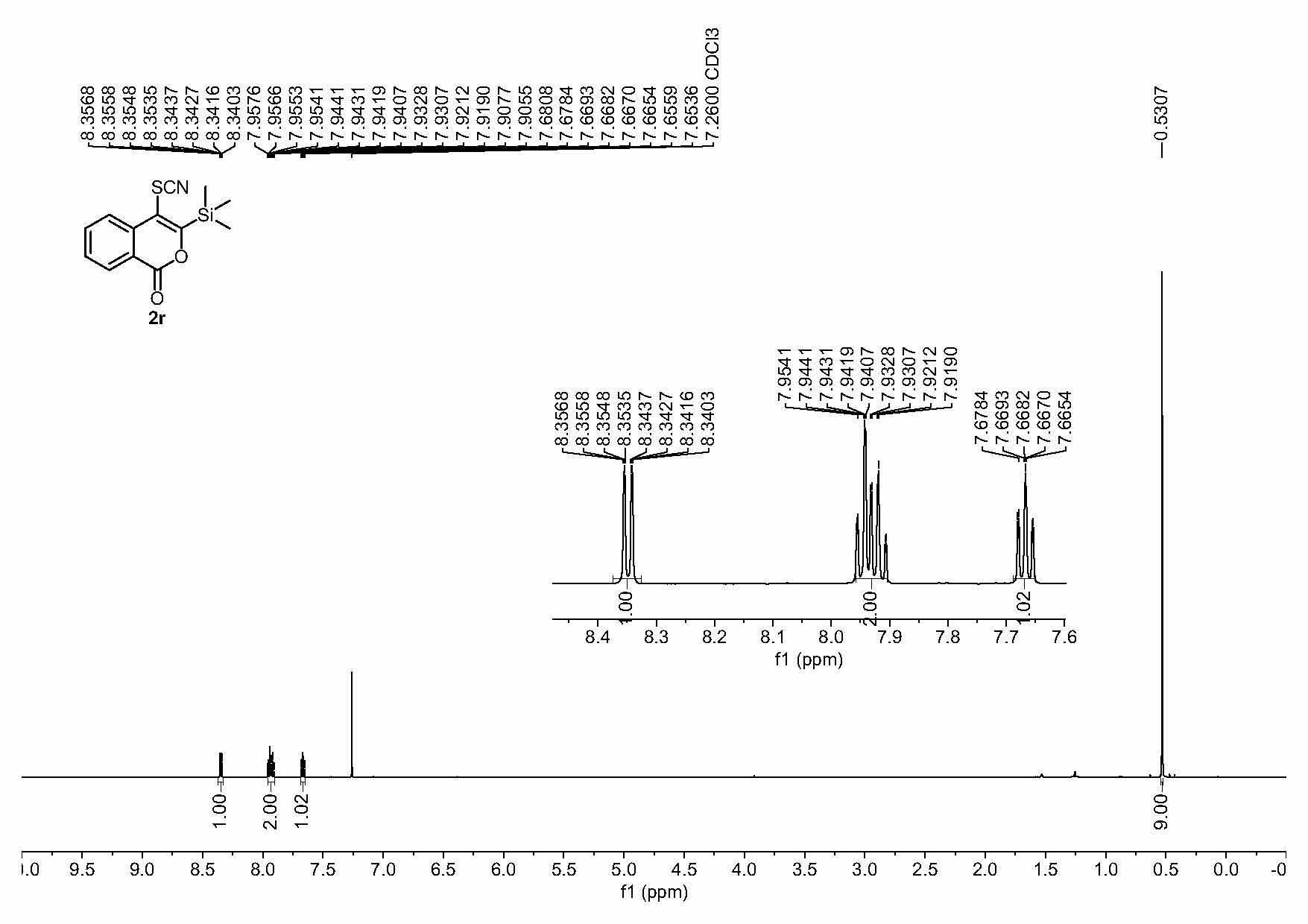


# VII X-Ray Structure and Data

Single crystals of C_17_H_11_NO_3_S **2h** were recrystallized from CHCl_3_ and n-hexane. A suitable crystal was selected for the determination of its structure *via* X-ray crystallography. The crystal was kept at 159.99 (10) K during data collection. Using Olex2, the structure was solved with the ShelXT structure solution program using Intrinsic Phasing and refined with the ShelXL refinement package using Least Squares minimisation. Compound **2h** crystallizes monoclinic, space group P2_1_/n (no. 14), *a* = 9.2559(2) Å, *b* = 8.9951(2) Å, *c* = 17.2761(4) Å, *β* = 103.136(2), *V* = 1400.73(6) Å^3^, *Z* = 4, *T* = 159.99(10) K, 59.99(1) = 0.243 mm^-1^, *Dcalc* = 1.467 g/cm^3^, 16068 reflections measured (4.616ares minimisation.ation.ined wit*R*_int_= 0.0270, R_sigma_ = 0.0206) which were used in all calculations. The final *R*_1_ was 0.0320 (I > 2σ(I)) and *wR*_2_ was 0.0881 (all data).

**Figure S3.** X-ray crystal structure of **2h** with 50% ellipsoid probability

| **Table S3 Crystal data and structure refinement for 2h.** | |
| --- | --- |
| Identification code | **2h** |
| Empirical formula | C_17_H_11_NO_3_S |
| Formula weight | 309.33 |
| Temperature/K | 159.99(10) |
| Crystal system | monoclinic |
| Space group | P2_1_/n |
| a/Å | 9.2559(2) |
| b/Å | 8.9951(2) |
| c/Å | 17.2761(4) |
| α/° | 90 |
| β/° | 103.136(2) |
| γ/° | 90 |
| Volume/Å^3^ | 1400.73(6) |
| Z | 4 |
| ρ_calc_g/cm^3^ | 1.467 |
| μ/mm^‑1^ | 0.243 |
| F(000) | 640.0 |
| Crystal size/mm^3^ | 0.15 × 0.15 × 0.1 |
| Radiation | Mo Kα (λ = 0.71073) |
| 2Θ range for data collection/° | 4.616 to 59.508 |
| Index ranges | -10 ≤ h ≤ 12, -9 ≤ k ≤ 12, -22 ≤ l ≤ 21 |
| Reflections collected | 16068 |
| Independent reflections | 3344 [R_int_ = 0.0270, R_sigma_ = 0.0206] |
| Data/restraints/parameters | 3344/0/201 |
| Goodness-of-fit on F^2^ | 1.071 |
| Final R indexes [I>=2σ (I)] | R_1_ = 0.0320, wR_2_ = 0.0851 |
| Final R indexes [all data] | R_1_ = 0.0359, wR_2_ = 0.0881 |
| Largest diff. peak/hole / e Å^-3^ | 0.34/-0.27 |

| **Table S4 Fractional Atomic Coordinates (×10^4^) and Equivalent Isotropic Displacement Parameters (Å^2^× a^3^) for 2h. U_eq_ is defined as 1/3 of of the trace of the orthogonalised U_IJ_ tensor.** | | | | | | | | | | |
| --- | --- | --- | --- | --- | --- | --- | --- | --- | --- | --- |
| **Atom** | | ***x*** | | ***y*** | | | ***z*** | | **U(eq)** | |
| S1 | | 2507.5(3) | | 8258.8(4) | | | 7480.3(2) | | 23.68(11) | |
| O2 | | 1088.3(9) | | 7205.4(10) | | | 5182.9(5) | | 21.83(19) | |
| O1 | | -929.2(10) | | 7570.3(11) | | | 4246.4(5) | | 27.4(2) | |
| O3 | | 7210.0(10) | | 4146.9(11) | | | 6191.2(6) | | 31.6(2) | |
| N1 | | 896.0(14) | | 6101.8(14) | | | 8150.7(7) | | 34.4(3) | |
| C6 | | -6.8(13) | | 8932.2(13) | | | 6270.2(7) | | 20.4(2) | |
| C5 | | -854.8(12) | | 8746.0(13) | | | 5488.7(7) | | 20.7(2) | |
| C8 | | 1943.6(12) | | 7398.2(13) | | | 5940.5(7) | | 19.9(2) | |
| C11 | | 3366.1(12) | | 6606.3(13) | | | 6026.0(7) | | 20.1(2) | |
| C15 | | 6050.9(13) | | 6521.1(14) | | | 6370.4(7) | | 23.4(3) | |
| C7 | | 1427.4(13) | | 8188.0(13) | | | 6483.9(7) | | 20.4(2) | |
| C16 | | 4725.9(13) | | 7300.3(14) | | | 6312.0(7) | | 22.5(2) | |
| C12 | | 3341.9(13) | | 5118.4(14) | | | 5783.7(7) | | 23.3(2) | |
| C9 | | -297.7(12) | | 7833.9(13) | | | 4921.5(7) | | 20.5(2) | |
| C4 | | -2242.3(13) | | 9432.2(14) | | | 5236.5(8) | | 25.3(3) | |
| C14 | | 6004.7(13) | | 5031.6(14) | | | 6150.1(7) | | 23.0(3) | |
| C10 | | 1535.1(13) | | 6970.2(14) | | | 7872.1(7) | | 23.8(3) | |
| C13 | | 4642.5(14) | | 4336.5(14) | | | 5851.4(7) | | 25.1(3) | |
| C3 | | -2783.4(14) | | 10311.3(16) | | | 5759.9(8) | | 30.1(3) | |
| C1 | | -590.3(14) | | 9819.4(15) | | | 6792.9(7) | | 26.4(3) | |
| C2 | | -1954.6(15) | | 10502.1(16) | | | 6534.5(8) | | 30.8(3) | |
| C17 | | 8638.0(15) | | 4776.2(19) | | | 6486.5(10) | | 40.8(4) | |
| **Table S5 Anisotropic Displacement Parameters (Å^2^×10^3^) for 2h. The Anisotropic displacement factor exponent takes the form: -2π^2^[h^2^a*^2^U_11_+2hka*b*U_12_+…].** | | | | | | | | | | |
| **Atom** | **U_11_** | | **U_22_** | | **U_33_** | **U_23_** | | **U_13_** | | **U_12_** |
| S1 | 23.27(16) | | 30.75(19) | | 16.05(16) | -2.23(10) | | 2.49(11) | | -5.07(11) |
| O2 | 21.9(4) | | 27.0(5) | | 15.7(4) | -2.7(3) | | 2.4(3) | | 1.4(3) |
| O1 | 26.9(4) | | 33.4(5) | | 19.5(4) | -4.1(3) | | 0.1(4) | | 0.0(4) |
| O3 | 26.6(5) | | 30.5(5) | | 35.6(5) | -3.8(4) | | 2.8(4) | | 7.8(4) |
| N1 | 43.7(7) | | 32.6(7) | | 23.9(6) | 1.5(5) | | 1.1(5) | | -10.0(5) |
| C6 | 22.9(5) | | 20.2(6) | | 19.3(5) | 0.8(4) | | 7.0(4) | | -1.8(4) |
| C5 | 21.7(5) | | 20.8(6) | | 20.4(5) | 1.9(4) | | 6.2(4) | | -2.1(4) |
| C8 | 21.1(5) | | 21.3(6) | | 16.7(5) | 0.6(4) | | 2.9(4) | | -2.7(4) |
| C11 | 21.2(5) | | 22.9(6) | | 16.6(5) | 1.1(4) | | 4.8(4) | | -0.2(4) |
| C15 | 21.1(5) | | 26.7(6) | | 21.9(6) | -1.3(5) | | 3.5(4) | | -1.5(4) |
| C7 | 20.5(5) | | 23.6(6) | | 16.5(5) | -0.1(4) | | 2.8(4) | | -2.9(4) |
| C16 | 24.0(5) | | 21.3(6) | | 22.3(6) | -1.5(4) | | 5.5(5) | | -1.3(4) |
| C12 | 24.7(6) | | 23.1(6) | | 21.7(6) | -1.0(4) | | 4.0(5) | | -3.8(4) |
| C9 | 21.2(5) | | 21.1(6) | | 19.2(5) | 1.6(4) | | 4.2(4) | | -2.9(4) |
| C4 | 23.9(6) | | 27.1(7) | | 24.3(6) | 3.8(5) | | 4.4(5) | | -0.6(5) |
| C14 | 25.3(6) | | 25.6(6) | | 18.5(5) | 2.2(4) | | 5.6(5) | | 4.1(5) |
| C10 | 26.4(6) | | 25.9(6) | | 16.6(5) | -2.9(4) | | -0.2(5) | | 0.1(5) |
| C13 | 31.5(6) | | 19.3(6) | | 24.5(6) | -1.0(4) | | 6.6(5) | | -0.3(5) |
| C3 | 26.7(6) | | 30.8(7) | | 33.6(7) | 4.2(5) | | 8.6(5) | | 5.9(5) |
| C1 | 29.9(6) | | 28.6(7) | | 21.6(6) | -1.8(5) | | 7.5(5) | | 0.7(5) |
| C2 | 35.8(7) | | 30.1(7) | | 30.1(7) | -1.5(5) | | 14.7(6) | | 6.1(5) |
| C17 | 24.5(6) | | 48.0(9) | | 46.4(9) | -13.3(7) | | 0.7(6) | | 7.4(6) |

| **Table S6 Bond Lengths for 2h.** | | | | | | |
| --- | --- | --- | --- | --- | --- | --- |
| **Atom** | **Atom** | **Length/Å** |  | **Atom** | **Atom** | **Length/Å** |
| S1 | C7 | 1.7846(12) |  | C5 | C4 | 1.4021(16) |
| S1 | C10 | 1.6996(13) |  | C8 | C11 | 1.4746(16) |
| O2 | C8 | 1.3780(13) |  | C8 | C7 | 1.3484(17) |
| O2 | C9 | 1.3806(14) |  | C11 | C16 | 1.3912(16) |
| O1 | C9 | 1.2035(14) |  | C11 | C12 | 1.4010(17) |
| O3 | C14 | 1.3590(14) |  | C15 | C16 | 1.3962(16) |
| O3 | C17 | 1.4217(17) |  | C15 | C14 | 1.3909(19) |
| N1 | C10 | 1.1495(18) |  | C12 | C13 | 1.3760(17) |
| C6 | C5 | 1.4091(16) |  | C4 | C3 | 1.3783(19) |
| C6 | C7 | 1.4573(16) |  | C14 | C13 | 1.3969(17) |
| C6 | C1 | 1.4020(17) |  | C3 | C2 | 1.394(2) |
| C5 | C9 | 1.4589(17) |  | C1 | C2 | 1.3838(18) |

| **Table S7 Bond Angles for 2h.** | | | | | | | | |
| --- | --- | --- | --- | --- | --- | --- | --- | --- |
| **Atom** | **Atom** | **Atom** | **Angle/˚** |  | **Atom** | **Atom** | **Atom** | **Angle/˚** |
| C10 | S1 | C7 | 97.38(5) |  | C8 | C7 | S1 | 118.74(9) |
| C8 | O2 | C9 | 123.34(9) |  | C8 | C7 | C6 | 120.81(10) |
| C14 | O3 | C17 | 118.08(11) |  | C11 | C16 | C15 | 120.62(11) |
| C5 | C6 | C7 | 117.72(10) |  | C13 | C12 | C11 | 120.57(11) |
| C1 | C6 | C5 | 118.20(11) |  | O2 | C9 | C5 | 117.04(10) |
| C1 | C6 | C7 | 124.08(11) |  | O1 | C9 | O2 | 116.54(11) |
| C6 | C5 | C9 | 120.36(10) |  | O1 | C9 | C5 | 126.42(11) |
| C4 | C5 | C6 | 121.12(11) |  | C3 | C4 | C5 | 119.50(12) |
| C4 | C5 | C9 | 118.52(11) |  | O3 | C14 | C15 | 125.17(11) |
| O2 | C8 | C11 | 109.96(10) |  | O3 | C14 | C13 | 114.74(11) |
| C7 | C8 | O2 | 120.64(10) |  | C15 | C14 | C13 | 120.09(11) |
| C7 | C8 | C11 | 129.38(10) |  | N1 | C10 | S1 | 178.70(11) |
| C16 | C11 | C8 | 122.27(11) |  | C12 | C13 | C14 | 120.09(12) |
| C16 | C11 | C12 | 119.12(11) |  | C4 | C3 | C2 | 119.85(12) |
| C12 | C11 | C8 | 118.57(10) |  | C2 | C1 | C6 | 120.11(12) |
| C14 | C15 | C16 | 119.47(11) |  | C1 | C2 | C3 | 121.20(12) |
| C6 | C7 | S1 | 120.42(9) |  |  |  |  |  |

| **Table S8 Torsion Angles for 2h.** | | | | | | | | | | | | | | |
| --- | --- | --- | --- | --- | --- | --- | --- | --- | --- | --- | --- | --- | --- | --- |
| **A** | **B** | | **C** | **D** | **Angle/6** | |  | **A** | | **B** | **C** | **D** | **Angle/6** | |
| O2 | C8 | | C11 | C16 | 128.04(12) | |  | C7 | | C6 | C1 | C2 | 179.04(12) | |
| O2 | C8 | | C11 | C12 | -49.68(14) | |  | C7 | | C8 | C11 | C16 | -53.65(18) | |
| O2 | C8 | | C7 | S1 | 174.26(8) | |  | C7 | | C8 | C11 | C12 | 128.63(14) | |
| O2 | C8 | | C7 | C6 | -3.80(18) | |  | C16 | | C11 | C12 | C13 | 2.12(18) | |
| O3 | C14 | | C13 | C12 | 179.81(11) | |  | C16 | | C15 | C14 | O3 | -178.85(11) | |
| C6 | C5 | | C9 | O2 | -1.35(16) | |  | C16 | | C15 | C14 | C13 | 1.84(18) | |
| C6 | C5 | | C9 | O1 | 178.46(12) | |  | C12 | | C11 | C16 | C15 | -1.07(18) | |
| C6 | C5 | | C4 | C3 | 0.40(19) | |  | C9 | | O2 | C8 | C11 | -179.06(10) | |
| C6 | C1 | | C2 | C3 | 0.9(2) | |  | C9 | | O2 | C8 | C7 | 2.45(17) | |
| C5 | C6 | | C7 | S1 | -175.49(9) | |  | C9 | | C5 | C4 | C3 | -179.33(11) | |
| C5 | C6 | | C7 | C8 | 2.54(17) | |  | C4 | | C5 | C9 | O2 | 178.39(10) | |
| C5 | C6 | | C1 | C2 | -0.89(18) | |  | C4 | | C5 | C9 | O1 | -1.80(19) | |
| C5 | C4 | | C3 | C2 | -0.4(2) | |  | C4 | | C3 | C2 | C1 | -0.2(2) | |
| C8 | O2 | | C9 | O1 | -179.67(10) | |  | C14 | | C15 | C16 | C11 | -0.89(18) | |
| C8 | O2 | | C9 | C5 | 0.16(16) | |  | C10 | | S1 | C7 | C6 | 77.49(10) | |
| C8 | C11 | | C16 | C15 | -178.78(11) | |  | C10 | | S1 | C7 | C8 | -100.58(10) | |
| C8 | C11 | | C12 | C13 | 179.91(11) | |  | C1 | | C6 | C5 | C9 | 180.00(11) | |
| C11 | C8 | | C7 | S1 | -3.89(18) | |  | C1 | | C6 | C5 | C4 | 0.27(18) | |
| C11 | C8 | | C7 | C6 | 178.05(11) | |  | C1 | | C6 | C7 | S1 | 4.58(17) | |
| C11 | C12 | | C13 | C14 | -1.18(18) | |  | C1 | | C6 | C7 | C8 | -177.39(12) | |
| C15 | C14 | | C13 | C12 | -0.82(18) | |  | C17 | | O3 | C14 | C15 | 0.44(19) | |
| C7 | C6 | | C5 | C9 | 0.06(16) | |  | C17 | | O3 | C14 | C13 | 179.77(12) | |
| C7 | C6 | | C5 | C4 | -179.67(11) | |  |  | |  |  |  |  | |
| **Table S9 Hydrogen Atom Coordinates (Å×10^4^) and Isotropic Displacement Parameters (Å^2^× a^3^) for 2h.** | | | | | | | | | | | | | | |
| **Atom** | | ***x*** | | | | ***y*** | | | ***z*** | | | | | **U(eq)** |
| H15 | | 6957.16 | | | | 6994.29 | | | 6555.13 | | | | | 28 |
| H16 | | 4752.59 | | | | 8292.57 | | | 6465.85 | | | | | 27 |
| H12 | | 2439.06 | | | | 4655.16 | | | 5575.26 | | | | | 28 |
| H4 | | -2793.48 | | | | 9295.15 | | | 4719.95 | | | | | 30 |
| H13 | | 4614.49 | | | | 3343.91 | | | 5698.09 | | | | | 30 |
| H3 | | -3699.66 | | | | 10776.2 | | | 5596.54 | | | | | 36 |
| H1 | | -59.57 | | | | 9949.24 | | | 7314.16 | | | | | 32 |
| H2 | | -2325.08 | | | | 11099.11 | | | 6884.07 | | | | | 37 |
| H17A | | 8780.85 | | | | 5593.04 | | | 6154.1 | | | | | 61 |
| H17B | | 9383.97 | | | | 4035.24 | | | 6485.45 | | | | | 61 |
| H17C | | 8713.29 | | | | 5125.31 | | | 7019.29 | | | | | 61 |

Single crystals of C_17_H_11_NO_3_Se **3h** were recrystallized from CHCl_3_ and n-hexane. A suitable crystal was selected for the determination of its structure *via* X-ray crystallography. The crystal was kept at 159.99 (10) K during data collection. Using Olex2, the structure was solved with the ShelXT structure solution program using Intrinsic Phasing and refined with the ShelXL refinement package using Least Squares minimisation. Compound **3h** crystallizes monoclinic, space group P2_1_/n (no. 14), *a* = 9.2710(2) Å, *b* = 9.0200(2) Å, *c* = 17.4122(5) Å, *β* = 103.294(3), *V* = 1417.07(6) Å^3^, *Z* = 4, *T* = 159.98(10) K, 59MoKα) = 2.662 mm^-1^, *Dcalc* = 1.670 g/cm^3^, 9250 reflections measured (4.6Squares minimisation.ation.ined *R*_int_ = 0.0250, R_sigma_ = 0.0309) which were used in all calculations. The final *R*_1_ was 0.0315 (I > 2ere used *wR*_2_ was 0.0832 (all data).

**Figure S4.** X-ray crystal structure of **3h** with 50% ellipsoid probability

| **Table S10 Crystal data and structure refinement for 3h.** | |
| --- | --- |
| Identification code | **3h** |
| Empirical formula | C_17_H_11_NO_3_Se |
| Formula weight | 356.23 |
| Temperature/K | 159.98(10) |
| Crystal system | monoclinic |
| Space group | P2_1_/n |
| a/Å | 9.2710(2) |
| b/Å | 9.0200(2) |
| c/Å | 17.4122(5) |
| α/° | 90 |
| β/° | 103.294(3) |
| γ/° | 90 |
| Volume/Å^3^ | 1417.07(6) |
| Z | 4 |
| ρ_calc_g/cm^3^ | 1.670 |
| μ/mm^‑1^ | 2.662 |
| F(000) | 712.0 |
| Crystal size/mm^3^ | 0.15 × 0.15 × 0.1 |
| Radiation | MoKα (λ = 0.71073) |
| 2Θ range for data collection/° | 4.6 to 59.346 |
| Index ranges | -10 ≤ h ≤ 11, -11 ≤ k ≤ 8, -19 ≤ l ≤ 21 |
| Reflections collected | 9250 |
| Independent reflections | 3337 [R_int_ = 0.0250, R_sigma_ = 0.0309] |
| Data/restraints/parameters | 3337/0/200 |
| Goodness-of-fit on F^2^ | 1.030 |
| Final R indexes [I>=2σ (I)] | R_1_ = 0.0315, wR_2_ = 0.0798 |
| Final R indexes [all data] | R_1_ = 0.0402, wR_2_ = 0.0832 |
| Largest diff. peak/hole / e Å^-3^ | 0.79/-0.43 |

| **Table S11 Fractional Atomic Coordinates (×10^4^) and Equivalent Isotropic Displacement Parameters (Å^2^×10^3^) for 3h. U_eq_ is defined as 1/3 of of the trace of the orthogonalised U_IJ_ tensor.** | | | | |
| --- | --- | --- | --- | --- |
| **Atom** | ***x*** | ***y*** | ***z*** | **U(eq)** |
| Se1 | 7397.8(2) | 1724.5(2) | 2478.7(2) | 22.10(9) |
| O2 | 8949.6(15) | 2767.1(15) | 4835.0(8) | 21.3(3) |
| O1 | 10973.5(15) | 2397.1(15) | 5758.8(9) | 25.7(3) |
| O3 | 2865.6(17) | 5892.6(15) | 3830.1(10) | 32.0(4) |
| N1 | 9214(2) | 3977(2) | 1855.6(12) | 33.4(5) |
| C7 | 8577(2) | 1793(2) | 3542.8(13) | 20.2(4) |
| C8 | 8080(2) | 2578(2) | 4080.1(12) | 18.8(4) |
| C6 | 10020(2) | 1059(2) | 3744.3(12) | 19.1(4) |
| C5 | 10875(2) | 1240(2) | 4519.2(12) | 19.3(4) |
| C9 | 10331(2) | 2139(2) | 5087.6(13) | 20.3(4) |
| C12 | 6710(2) | 4863(2) | 4243.4(12) | 22.9(4) |
| C11 | 6669(2) | 3378.0(19) | 4005.9(12) | 18.6(4) |
| C10 | 8534(2) | 3108(2) | 2099.7(13) | 22.9(4) |
| C16 | 5301(2) | 2708(2) | 3720.5(12) | 21.4(4) |
| C14 | 4057(2) | 4990(2) | 3869.8(12) | 22.7(4) |
| C4 | 12269(2) | 563(2) | 4763.0(14) | 24.8(5) |
| C1 | 10602(2) | 192(2) | 3224.6(13) | 25.1(4) |
| C13 | 5422(2) | 5657(2) | 4173.0(13) | 24.0(5) |
| C15 | 3989(2) | 3506(2) | 3653.7(13) | 23.3(4) |
| C3 | 12803(2) | -297(2) | 4240.3(14) | 29.8(5) |
| C2 | 11968(3) | -475(2) | 3473.7(15) | 29.8(5) |
| C17 | 1434(3) | 5275(3) | 3549.3(18) | 42.9(7) |

| **Table S12 Anisotropic Displacement Parameters (Å^2^×10^3^) for 3h. The Anisotropic displacement factor exponent takes the form: -2π^2^[h^2^a*^2^U_11_+2hka*b*U_12_+…].** | | | | | | |
| --- | --- | --- | --- | --- | --- | --- |
| **Atom** | **U_11_** | **U_22_** | **U_33_** | **U_23_** | **U_13_** | **U_12_** |
| Se1 | 20.55(13) | 29.11(14) | 14.54(13) | -1.89(7) | -0.30(9) | -4.68(7) |
| O2 | 19.4(7) | 26.3(7) | 15.2(8) | -3.0(5) | -2.0(6) | 1.2(5) |
| O1 | 22.9(7) | 33.5(8) | 16.2(8) | -3.4(6) | -4.7(6) | -0.4(6) |
| O3 | 26.4(8) | 33.0(9) | 33.0(10) | -4.6(6) | -0.5(7) | 9.7(6) |
| N1 | 40.6(12) | 30.8(10) | 24.3(11) | 0.0(8) | -1.9(9) | -8.2(8) |
| C7 | 19.1(10) | 22.9(10) | 17.1(11) | -0.6(7) | 0.9(8) | -3.2(7) |
| C8 | 20.5(9) | 20.5(10) | 13.5(10) | 0.5(7) | 0.2(8) | -3.7(7) |
| C6 | 19.6(10) | 17.6(9) | 19.4(11) | 0.2(7) | 3.1(8) | -2.0(7) |
| C5 | 19.9(9) | 19.4(9) | 18.2(10) | 1.9(8) | 3.2(8) | -3.0(7) |
| C9 | 18.8(10) | 19.7(9) | 21.0(11) | 1.8(8) | 1.7(9) | -3.1(7) |
| C12 | 24.9(10) | 22.9(10) | 19.4(11) | -1.0(8) | 2.0(9) | -3.5(8) |
| C11 | 20.1(10) | 21.7(10) | 13.3(10) | 1.9(7) | 2.3(8) | 0.5(7) |
| C10 | 24.0(10) | 24.2(10) | 16.5(11) | -3.2(8) | -3.5(9) | -0.9(8) |
| C16 | 22.8(10) | 21.1(9) | 19.6(11) | -1.8(8) | 3.1(8) | -0.9(7) |
| C14 | 23.5(10) | 27.4(11) | 16.8(11) | 2.4(8) | 4.1(9) | 6.1(8) |
| C4 | 22.1(11) | 25.1(10) | 25.2(12) | 3.3(8) | 1.2(9) | 0.4(7) |
| C1 | 26.0(11) | 27.7(10) | 20.5(11) | -1.9(8) | 3.4(9) | 0.9(8) |
| C13 | 30.9(12) | 19.5(10) | 21.0(12) | -1.3(7) | 4.5(9) | 0.7(7) |
| C15 | 19.6(10) | 28.4(11) | 20.3(11) | -1.3(8) | 1.1(9) | -1.3(7) |
| C3 | 24.1(11) | 30.0(11) | 35.3(14) | 5.3(10) | 7.0(10) | 7.0(8) |
| C2 | 31.9(12) | 30.0(11) | 29.4(13) | -1.7(9) | 10.8(11) | 6.1(9) |
| C17 | 24.9(12) | 53.3(15) | 44.7(17) | -14.7(13) | -4.2(12) | 13.0(11) |

| **Table S13 Bond Lengths for 3h.** | | | | | | |
| --- | --- | --- | --- | --- | --- | --- |
| **Atom** | **Atom** | **Length/Å** |  | **Atom** | **Atom** | **Length/Å** |
| Se1 | C7 | 1.924(2) |  | C6 | C1 | 1.395(3) |
| Se1 | C10 | 1.850(2) |  | C5 | C9 | 1.457(3) |
| O2 | C8 | 1.385(2) |  | C5 | C4 | 1.404(3) |
| O2 | C9 | 1.376(2) |  | C12 | C11 | 1.399(3) |
| O1 | C9 | 1.206(3) |  | C12 | C13 | 1.374(3) |
| O3 | C14 | 1.361(2) |  | C11 | C16 | 1.389(3) |
| O3 | C17 | 1.419(3) |  | C16 | C15 | 1.395(3) |
| N1 | C10 | 1.147(3) |  | C14 | C13 | 1.391(3) |
| C7 | C8 | 1.337(3) |  | C14 | C15 | 1.388(3) |
| C7 | C6 | 1.461(3) |  | C4 | C3 | 1.372(3) |
| C8 | C11 | 1.473(3) |  | C1 | C2 | 1.379(3) |
| C6 | C5 | 1.408(3) |  | C3 | C2 | 1.390(3) |

| **Table S14 Bond Angles for 3h.** | | | | | | | | |
| --- | --- | --- | --- | --- | --- | --- | --- | --- |
| **Atom** | **Atom** | **Atom** | **Angle/˚** |  | **Atom** | **Atom** | **Atom** | **Angle/˚** |
| C10 | Se1 | C7 | 94.45(8) |  | O1 | C9 | C5 | 126.47(19) |
| C9 | O2 | C8 | 123.25(16) |  | C13 | C12 | C11 | 120.60(19) |
| C14 | O3 | C17 | 117.82(17) |  | C12 | C11 | C8 | 118.64(17) |
| C8 | C7 | Se1 | 118.68(15) |  | C16 | C11 | C8 | 122.50(17) |
| C8 | C7 | C6 | 121.06(19) |  | C16 | C11 | C12 | 118.86(18) |
| C6 | C7 | Se1 | 120.18(15) |  | N1 | C10 | Se1 | 178.7(2) |
| O2 | C8 | C11 | 109.53(16) |  | C11 | C16 | C15 | 120.79(18) |
| C7 | C8 | O2 | 120.66(18) |  | O3 | C14 | C13 | 114.69(18) |
| C7 | C8 | C11 | 129.80(18) |  | O3 | C14 | C15 | 125.23(18) |
| C5 | C6 | C7 | 117.37(18) |  | C15 | C14 | C13 | 120.07(18) |
| C1 | C6 | C7 | 124.59(19) |  | C3 | C4 | C5 | 119.5(2) |
| C1 | C6 | C5 | 118.04(18) |  | C2 | C1 | C6 | 120.3(2) |
| C6 | C5 | C9 | 120.60(18) |  | C12 | C13 | C14 | 120.27(18) |
| C4 | C5 | C6 | 121.01(19) |  | C14 | C15 | C16 | 119.36(19) |
| C4 | C5 | C9 | 118.40(19) |  | C4 | C3 | C2 | 119.8(2) |
| O2 | C9 | C5 | 117.01(18) |  | C1 | C2 | C3 | 121.3(2) |
| O1 | C9 | O2 | 116.52(19) |  |  |  |  |  |

| **Table S15 Hydrogen Atom Coordinates (År e^4^) and Isotropic Displacement Parameters (Å^2^× a^3^) for 3h.** | | | | |
| --- | --- | --- | --- | --- |
| **Atom** | ***x*** | ***y*** | ***z*** | **U(eq)** |
| H12 | 7617.14 | 5315.07 | 4450.83 | 27 |
| H16 | 5259.76 | 1715.75 | 3572.08 | 26 |
| H4 | 12826.87 | 695.75 | 5275.22 | 30 |
| H1 | 10066.16 | 62.69 | 2707.7 | 30 |
| H13 | 5463.11 | 6645.75 | 4328.72 | 29 |
| H15 | 3077.11 | 3048.14 | 3466.32 | 28 |
| H3 | 13719.85 | -759.5 | 4398.27 | 36 |
| H2 | 12338.29 | -1055.69 | 3121.57 | 36 |
| H17A | 1339.1 | 4921.97 | 3019.77 | 64 |
| H17B | 697.03 | 6019.37 | 3554.94 | 64 |
| H17C | 1298.01 | 4464.43 | 3883.05 | 64 |
